# Synthesis, Biological Activity, and Molecular-Docking Studies of New Brassinosteroid Analogs

**DOI:** 10.3390/ijms251810158

**Published:** 2024-09-21

**Authors:** María Nuñez, Yaowei Wang, Eugenia Russinova, Ana Estévez-Braun, Angel Amesty, Andrés F. Olea, Marco Mellado, Katy Díaz, Luis Espinoza Catalán

**Affiliations:** 1 Departamento de Química, Universidad Técnica Federico Santa María, Avenida España 1680, Valparaíso 2340000, Chile; maria.nunezg@usm.cl; 2 Department of Plant Biotechnology and Bioinformatics, Ghent University, 9052 Ghent, Belgium; yaoweiwang@126.com (Y.W.); eugenia.russinova@psb.vib-ugent.be (E.R.); 3 Center for Plant Systems Biology, VIB, 9052 Ghent, Belgium; 4 Departamento de Química Orgánica, Instituto Universitario de Bio-Orgánica Antonio González, Universidad de La Laguna, Avda. Astrofísico Francisco Sánchez 2, 38206 La Laguna, Tenerife, Spain; aestebra@ull.edu.es (A.E.-B.); aarnesty@ull.edu.es (A.A.); 5 Grupo QBAB, Instituto de Ciencias Aplicadas, Facultad de Ingeniería, Universidad Autónoma de Chile, El Llano Subercaseaux 2801, Santiago 8900000, Chile; andres.olea@uautonoma.cl; 6 Facultad de Medicina y Ciencias de la Salud, Universidad Central de Chile, Santiago 8330507, Chile; marco.mellado@ucentral.cl

**Keywords:** brassinosteroid analogs, plant growth promotion, synthesis, molecular docking

## Abstract

Much work has been dedicated to the quest to determine the structure–activity relationship in synthetic brassinosteroid (BR) analogs. Recently, it has been reported that analogs with phenyl or benzoate groups in the alkyl chain present activities comparable to those shown by natural BRs, depending on the nature of the substituent in the aromatic ring. However, as it is well known that the activity depends on the structure of the whole molecule, in this work, we have synthesized a series of compounds with the same substituted benzoate in the alkyl chain and a hydroxyl group at C3. The main goal was to compare the activities with analogs with -OH at C2 and C3. Additionally, a molecular-docking study and molecular dynamics simulations were performed to establish a correlation between the experimental and theoretical results. The synthesis of eight new BR analogs was described. All the analogs were fully characterized by spectroscopical methods. The bioactivity of these analogs was assessed using the rice lamina inclination test (RLIT) and the inhibition of the root and hypocotyl elongation of *Arabidopsis thaliana*. The results of the RLIT indicate that at the lowest tested concentration (1 × 10^−8^ M), in the BR analogs in which the aromatic ring was substituted at the para position with methoxy, the I and CN substituents were more active than brassinolide (50–72%) and 2–3 times more active than those analogs in which the substituent group was F, Cl or Br atoms. However, at the highest concentrations, brassinolide was the most active compound, and the structure–activity relationship changed. On the other hand, the results of the *A. thaliana* root sensitivity assay show that brassinolide and the analogs with I and CN as substituents on the benzoyl group were the most active compounds. These results are in line with those obtained via the RLIT. A comparison of these results with those obtained for similar analogs that had a hydroxyl group at C2 indicates the importance of considering the whole structure. The molecular-docking results indicate that all the analogs adopted a brassinolide-like orientation, while the stabilizing effect of the benzoate group on the interactions with the receptor complex provided energy binding values ranging between −10.17 and −13.17 kcal mol^−1^, where the analog with a nitrile group was the compound that achieved better contact with the amino acids present in the active site.

## 1. Introduction

Brassinosteroids (BRs) are a group of polyhydroxylated steroidal phytohormones required for the development, growth, and productivity of plants [1,2]. Additionally, it has been shown that BRs also have a positive impact on plant responses to various biotic and abiotic stresses [3,4].

**Figure 1:**
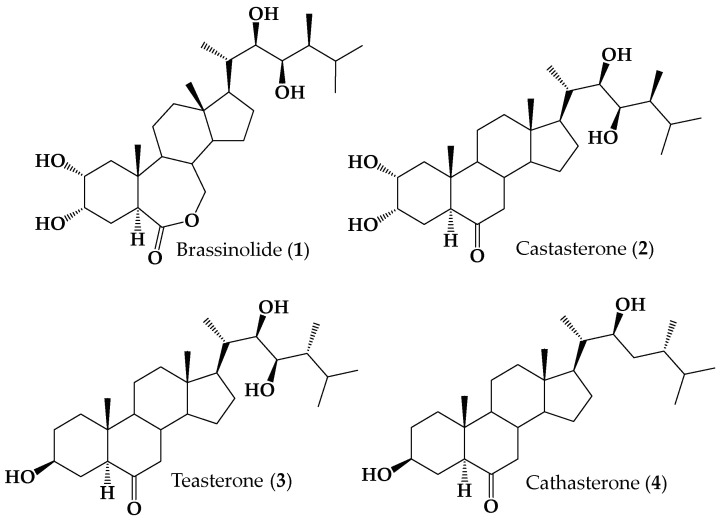
Structures of naturally occurring brassinosteroids **1**–**4**.

The low abundance of BRs in natural sources and their powerful biological activities have prompted considerable efforts to synthesize natural BRs and a host of new derivatives. However, as can be seen in Figure 1, the chemical structures of natural BRs are quite complex (i.e., they possess a significant number of chiral centers and functional groups).

Therefore, the total synthesis of natural BRs seems to be difficult and too expensive for agricultural applications, even though several groups have tried to achieve it [2,5,6,7,8,9]. Thus, partial syntheses of new analogs have been developed with the aim of establishing the structure–activity relationships (SARs) [10,11,12]. To achieve this goal, a family of structurally related compounds should be synthesized and tested for their bioactivity. For the synthesis of these compounds, it has become common to use, as the starting substances, available steroids with organic functions in appropriate positions, which can be easily and conveniently modified by direct chemical reactions [2]. One major challenge during the synthesis of natural BRs and their analogs is the assembly of the side alkyl chain, which requires at least three chiral centers, depending on the side chain and brassinosteroid structures. This construction is carried out by chain functionalization, which requires various strategies and synthesis steps and the use of chiral catalysts. As a result, the yields of the analogs are usually low [9]. Considering these synthesis-related difficulties, several groups have decided to introduce important structural modifications into the side chain; for example, the alkyl chain length [13,14,15,16,17,18], different oxygenated functions [19,20,21,22], spirostanic and furostanic moieties [23,24,25,26,27,28,29], cyclic substituents [10], methyl esters and/or carboxylic acids [30,31,32,33], nitrogen-containing side chains [34], and aromatic substituents [35].

**Figure 2:**
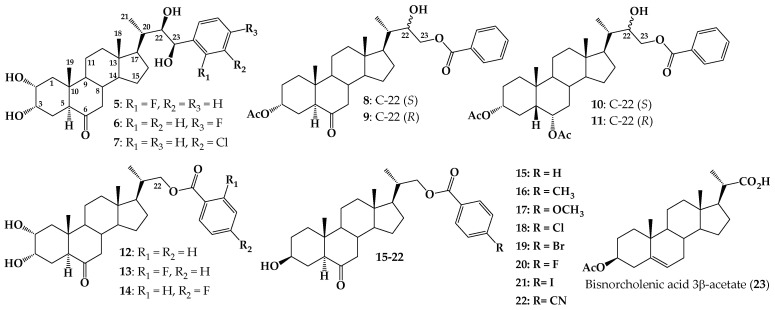
Structure of BR analogs with aryl ring at C-23 (**5**–**7**), 24-norcholane with benzoates at C-23 (compounds **8**–**11**), 23,24-dinorcholan type analogs with 2α,3α-dihydroxy and 22-benzoate having a fluorine substituent (compounds **12**–**14**) and new analogs 23,24-bisnorcholenic with 4-substituted benzoate at C-22, and different substituents at ˝*para”* position of aromatic ring (compounds **15**–**22**) and 23, 24-bisnorcholenic acid 3β-acetate (**23**).

In the latter group, some interesting BR analogs with an aromatic ring attached to the side chain (aryl, phenyl, or benzoate esters) have been synthesized (see Figure 2) and their bioactivity assessed using different bioassays [32,36,37].

For example, analogs **5**–**7** exhibit activity on ethylene production or inhibitory effect on *Arabidopsis* root growth [36,38]. BR analogs **8** and **9** were tested using the Rice Lamina Inclination Test (RLIT) and root elongation in *Arabidopsis thaliana*. Interestingly, analog **8** was more active than brassinolide (**1**) at all tested concentrations. Results from a molecular docking study suggest that this behavior could be explained in terms of hydrophobic interactions of the ligand with the receptor brassinosteroid-insensitive 1-leucine-rich repeat (BRI1-LRR) after formation of the complex BRI1–BRs-associated kinase 1 (BAK1) hydrogen bonding of the ligand with BAK1 [16]. Similar results were reported for diastereoisomers **10** and **11**, which were obtained as a mixture (ratio 1.0/0.44). RLIT measurements indicate that, at the lowest tested concentration (1 × 10^−8^ M), this mixture is notably more active than **1** [17]. Finally, evaluation of compounds **12**–**14** by RLIT and Bean Second Internode (BSI) bioassays, indicate that substitution of benzoate group at C-22 with F atom at “*ortho*” or “*para*” positions enhances the activities as compared with the analog **12**, which does not have a fluorine atom in the aromatic ring.

These preliminary studies suggest that the incorporation of a benzoate group at the C-22 and/or C-23 positions of the side chain could generate an increase in the biological activity of these compounds [37]. To test this hypothesis, and to evaluate the effect on the biological activity of different substituents in the aromatic ring, we have synthesized a new series of analogues 23,24-bisnorcholenic type with benzoate function at C-22 position of the side chain (compounds **15**–**22**, Figure 2). The bioactivity of these compounds was assessed by using RLIT, *Arabidopsis* root, and hypocotyl sensitivity bioassays, as a function of electronic and size parameters of the *p*-substituted benzoyl group. Finally, some tested analogs were subjected to a molecular docking study using Glide v9.7 and Maestro program.

The results reported herein are useful to establish new structural requirements for BR analogs' biological activity.

## 2. Results and Discussion

### 2.1. Chemical Synthesis

**Figure 3:**
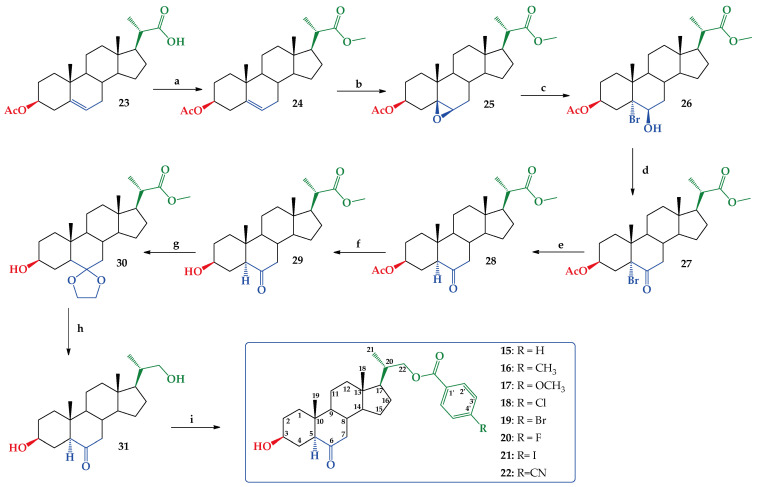
Synthesis of compounds **24**–**31** and new BR analogs **15**–**22**. Conditions **a**. CH_2_N_2_/ether, r.t. 6 h. 99.8%. **b**. KMnO_4_/Fe_2_(SO_4_)_3_, CH_2_Cl_2_, *t*-BuOH/H_2_O, r.t. 2 h. 92.7%. **c**. HBr (48% *w*/*w*)/CH_2_Cl_2_ r.t. 4 h. 47.7%. **d**. Jones CH_2_Cl_2_/(CH_3_)_2_CO r.t. 2 h. 100%. **e**. Zn/CH_3_CO_2_H, reflux. 4 h. 74.1%. f. NaHCO_3_, MeOH/H_2_O reflux 6 h. 90.5%. **g**. HOCH_2_CH_2_OH/TsOH, Dean–Stark apparatus, C_6_H_6_ reflux 2.5 h. 75.9%. **h**. (1) LiAlH_4_ 2M/THF, 1.5 h. (2) HCl (aq) 5%, 1 h. 79.9%. **i**. *p*-RC_6_H_4_COCl, CH_2_Cl_2_/py, DMAP, 3 h r.t. **15** (47.4%), **16** (32.6%), **17** (40.9%), **18** (41.3%), **19** (25.7%), **20** (46.0%), **21** (38.1%) and **22** (44.0%).

Synthesis of new BR analogs (compounds **15**–**22**, Figure 2) was performed using the commercially available (20*S*)-3β-acetoxy-5-pregnen-20-carboxylic acid (**23**) as starting material. Acid **23** was converted quantitatively (99.8% yield) in its methyl ester **24** by esterification with diazomethane/ether [39] (Figure 3). The melting point and the obtained spectroscopic data (IR and ^1^H NMR) were consistent with those reported [39]. The existing NMR spectroscopic information was complemented with ^13^C, ^13^C DEPT-135, 2D HSQC and 2D HMBC experiments (Figures S1–S5, Supplementary Material). Then, a reaction of **24** with a KMnO_4_ and Fe_2_(SO_4_)_3_ in a mixture of CH_2_Cl_2_, tert-butyl alcohol and H_2_O, produces epoxide **25** with 92.7% yield (Figure 3). This reaction is a highly diasteroselective β-epoxidation of the C5–C6 double bond (only traces of the α-epoxide could be detected by ^1^H NMR). Similar results have been reported in the synthesis of other steroidal epoxides with similar structures [40,41]. The β-epoxide stereochemistry was established by lD selective NOESY, which demonstrates the “α” spatial orientation of H-6, and therefore the “β” orientation of epoxide ring. The full structural determination was accompanied by other 1D and 2D NMR experiments (Figures S6–S12, Supplementary Material).

The regioselective opening of β-epoxide in compound **25** led to bromohydrin **26** as a unique product with a 47.7% yield (Figure 3). This reaction was carried out following a procedure described for the opening of epoxide rings in similar steroidal structures, and the stereochemistry at carbons C-5 and C-6 was consistent with reported data [40,41]. Additionally, the signal at δ = 4.20 ppm (1H, bt) assigned to H-6α, shows scalar coupling with *J* values expected for H atoms with equatorial-equatorial orientations (*J* = 2.9 Hz) for H7α and H-7β, which suggests that OH group in C-6 must have an axial orientation (Figures S13–S18, Supplementary Material). Subsequently, Jones oxidation [42] of **26** produces α-bromo ketone **27** quantitatively, according to a described procedure (Figures S19–S24, Supplementary Material) [41] (Figure 3). Reduction of **27** with Zn dust in refluxing glacial acetic acid leads to ketone **28** with a 74.1% yield [40,41]. The spatial orientation of H-5 was established as H-5α from the ^1^H NMR spectroscopic data. Thus, the signal at δ = 2.25 ppm (1H, dd) assigned to H-5α, shows scalar coupling with *J* values expected for hydrogens with axial-equatorial orientations (*J* = 14.0 and 4.0 Hz) for H4β and H-4α, required for the H-5 to have this “alpha” orientation (Figures S25–S30, Supplementary Material). Ketone **29** was obtained from **28** by selective saponification of the acetate group in C-3 with a 90.5% yield (Figures S31–S36, Supplementary Material) [39,43]. Protection of keto groups in **29** with ethylene glycol/TsOH produces the expected dioxolane derivative **30** with a 75.9% yield (Figures S37–S42, Supplementary Material) [44,45]. Reduction of **30** with LiAlH_4_, 2M/THF solution, and later acidification (HCl(aq) 5% *w*/*v*) produced compound **31** with 79.9% yield (Figures S43–S48, Supplementary Material) [44,45]. Finally, selective benzoylation of primary alcohol at C-22 with *p*-substituted benzoyl chlorides produces new BR analogs **15**–**22** with different yields (Figure 3) [14]. The low yields obtained in the latter step of the scheme reaction shown in Figure 3 might be due to the stoichiometric control used in benzoylation of compound **31**, to avoid dibenzoylation. All new analogs **15**–**22** were fully characterized by 1D and 2D NMR spectroscopic techniques (Figures S49–S104, Supplementary Material).

### 2.2. Biological Activity

Different bioassays have been proposed to detect and quantify BR’s bioactivity [2,11]. However, it has been observed that different plant responses can be obtained for the same compound when it is submitted to different bioactivity tests [11,12,16]. This is a very important point to keep in mind, especially when obtained data is compared with previous works or when it is intended to propose a SAR that rationalizes observed activity with BRs structure [12]. Thus, the bioactivity of new BR analogs was evaluated by using different bioassays, namely RLIT, inhibition of root and hypocotyl elongation bioassays [37].

#### 2.2.1. Rice Lamina Inclination Test

The rice lamina inclination test is a highly specific and sensitive assay that has become a standard method to detect and evaluate BR activity. In this bioassay, BR induces lamina joint inclination and angle inclination values vary in a concentration-dependent manner [46,47]. The data obtained herein for new BR analogs at different concentrations are shown in Table S1 and listed in Table 1.

The data show that the bioactivity of brassinolide increases with increasing concentration, i.e., the bending angle is 2–3 times higher at 1 μM, and it is the limiting value obtained for all tested compounds. On the other hand, the bioactivity of synthetic analogs does not change (**15**, **16**, **20**) or increase (**17**, **19**, **21**, **22**, **31**) with a greater concentration. This effect has been previously observed [16,17,37] and it could be attributed to additional processes affecting the activity of exogenously applied BRs, i.e., solubility, diffusion and local plant control of BR concentration. Somehow, these processes must be involved, but there are no experimental data to understand how BR activity is related to these factors.

Interestingly, at the lowest tested concentration (1 × 10^−8^ M) compounds **17**, **21** and **22** exhibit higher bioactivity than brassinolide. At this concentration, a relation between experimental activities and the chemical structure of the side chain in the new BRs gives some interesting results. Compounds in which the aromatic ring is substituted at “*para*” position with methoxy, I and CN substituent (**17**, **21** and **22**) are the most active, whereas those analogs in which the atom at “*para*” position is F, Cl, or Br, (**20**, **18** and **19**, respectively) exhibit the lowest activity in the RLIT. Finally, the analog with no substituent in the phenyl ring (**15**) is as active as brassinolide (**1**). In summary, at 0.01 μM the activity follows the order **17** = **21** = **22** > **1** = **15** = **16** > **18** = **19** = **20**. These results suggest that at this concentration, the substituent atom with the higher size induces the highest activity of BR analogs, whereas smallest atoms lead to activities like that shown by brassinolide.

On the other hand, compound **15** shows similar effects on RLIT at all tested concentrations. In other words, **15** at 0.01 μM concentration behaves as **1**, the most effective natural BR, and keeps the same activity at much higher concentrations.

Finally, a comparison of results reported for the series of compounds that have hydroxyl groups at both C2 and C3 [37] with those listed in Table 1 will allow us to assess the effect of -OH at C2 on RLIT activity for these BR analogs. Thus, the ratio of growth activity, **1**/analog, calculated for non-benzoylated analogs, **31** (Figure 3) and **8** (Figure 2, Ref. [47]) are 2.2 and 1.8, respectively, which indicate that the presence of -OH group at C2 enhances growth activity. On the other hand, for benzoylated compounds, this effect depends on the structure of the aromatic ring, namely, for no substituted benzoylated analogs, **12** and **15**, the growth activity slightly decreases (ratios are 2.2 and 1.7, respectively), whereas, for analogs carrying F-benzoate groups, 1**4** and **20**, there is almost no effect (ratios are 1.4 and 1.5, respectively). Interestingly, it has also been found that the presence of -OH at C2 in BR analog (22*R*, 23*R*)-3α,22,23-trihydroxy-23-phenyl-24-nor-5α-cholan-6-one increases RLIT activity [48]. Therefore, it becomes necessary to elucidate if the presence of both hydroxyl groups is a structural requirement for the activity of BR analogs in RLIT bioassay.

#### 2.2.2. Inhibition of Root and Hypocotyl Elongation in *Arabidopsis thaliana* Seedlings Assays

The effect of exogenous application of natural BRs on root growth has been studied and both inhibitory and stimulant effects have been reported [49,50,51]. Root growth enhancement is observed at low BR concentrations, whereas inhibitory effects appear above a threshold concentration of **1** equal to 1 µM [51,52]. This behavior has been explained in terms of enhanced biosynthesis of ethylene, which in turn regulates root growth.

**Figure 4:**
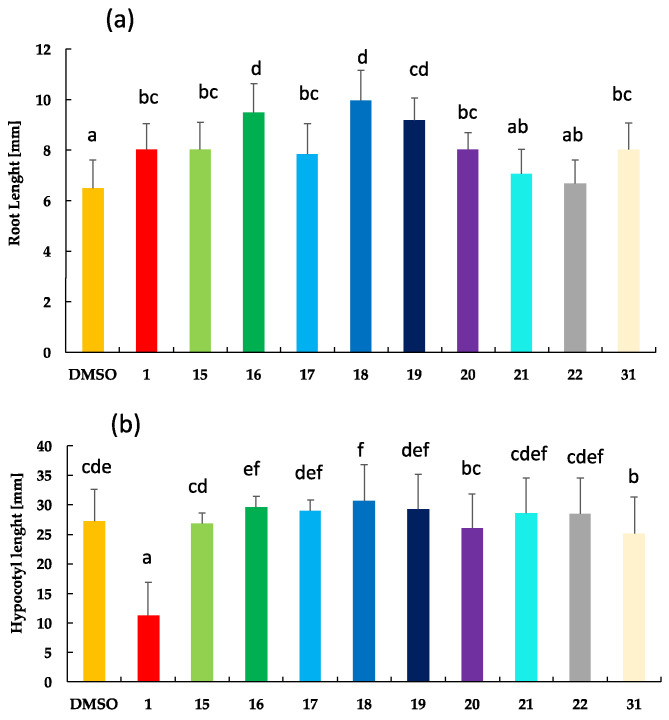
Effect of brassinolide (**1**), BR analogs with 23,24-bisnor-5α-cholane-type side chains (**15**–**22**) and analog **31** on the inhibition growth of Arabidopsis (**a**) root length and (**b**) hypocotyl length. Five days old *Arabidopsis thaliana* seedlings (Columbia ecotype, Col-0) were treated with 1 μM of **1** (positive control) and BR analogs, whereas 0.1% of DMSO was used as negative control. For each treatment, more than 15 seedlings were analyzed. Letters represent experiments significantly different at the 0.05 significance level. Error bars represent standard deviations of the means.

Thus, to compare activities on root length, all BR analogs were tested at 1 µM concentration, i.e., the limiting concentration at which the inhibitory effect is observed. Results are shown in Figure 4.

Results shown in Figure 4a indicate that brassinolide (**1**) and analogs **15**, **17**, **and 20** show similar activities; **16**, **18**, and **19** are less active than **1**; **21** and **22** are the most active BR analogs in this bioassay. Structurally, this relationship means that analogs with no substituted phenyl or substituted with methoxy group or F atom show the same activity as **1**. The activity decreases with the methyl group, Cl, and Br atoms, whereas it increases with the I atom and CN as substituents at the “*para*“ position. It is worth emphasizing that these results are clearly correlated with those obtained by RLIT, i.e., compounds **17**, **21** and **22** are as active as a brassinolide.

On the other hand, results from the hypocotyl growth inhibition test indicate that activities shown by all BR analogs are much lower than those determined for the positive control (Figure 4b). Additionally, there is almost no variation in activity by changing analogs’ chemical structure.

These results indicate that exogenous BRs have different effects on plant growth depending on the site of action, which could be attributed to differences in the local concentration of BRs. It is worth emphasizing that natural BRs induce ethylene biosynthesis through interaction with the transcription factors BES1 and BZR1 and promoters of ACSs, which encode the key enzyme of ethylene biosynthesis [51]. The signaling levels are due to local BRs accumulation, which is the result of the expression of BR biosynthetic enzymes and short-distance radial transport of BRs and their precursors [53]. Due to their limited mobility, local production of BRs leads to a local accumulation of hormones that, in turn, triggers signaling and allows the timely transition of meristematic cells to the elongation zone.

Thus, bioactivity results obtained with RLIT and *Arabidopsis bioassays* lead to a completely different structure–activity relationship. It must be emphasized that bioactivities obtained from different bioassays can vary because they measure intrinsically different plant processes, but also it should be considered that exogenous application of BRs introduces uncertainty regarding the exact quantity of BRs that is adsorbed by the plant and then transported to the active site [54]. For example, in RLIT and *Arabidopsis* bioassays BRs are added to the substrate in which rice plants or *Arabidopsis* seeds are growing, and therefore these molecules must enter through different plant barriers, and then diffuse to reach the active site to interact with. Thus, comparison of data from different bioassays must be avoided.

In previous works, Kvasnica et al. [36,38] have shown that substitution of phenyl group in the side chain of (22*R*, 23*R*)-2α,3α,22,23-tetr

ahydroxy-23-aryl-24-nor-5α-cholan-6-one induces important changes in bioactivity. Their results indicate that the most active compounds in *Arabidopsis* sensitive bioassays, namely those with no substitution or substitution with small groups (F, Cl) at “*ortho*” or “*meta*” positions (compounds **5**–**7**, Figure 2), induce accumulation of unphosphorylated BES-1, which play important role in BRs signaling pathway [55,56]. The effects of these types of BR analogs on *Arabidopsis* were explained by their interaction with the BR receptor revealed by a docking study. A comparison of both series of BR analogs shows that the main differences between them regarding the length of the side alkyl chain and the number of hydroxyl groups; namely, **15**–**22** have a side chain longer and just one hydroxyl group as compared to compounds **5**–**7** (see Figure 2). Thus, the different behavior in bioactivity shown by analogs **15**–**22** will be analyzed below by molecular docking and molecular dynamic simulations.

### 2.3. Molecular Docking and Induced Fit (IFD)

It has been well established that BRs are perceived at the cell surface by BRASSINOSTEROID INSENSITIVE 1 (BRI1) [57,58,59,60,61]. This is a leucine-rich repeat receptor kinase (LRR-RK) consisting of an extracellular LRR domain of 25 LRRs, a single-pass transmembrane segment, followed by a juxta membrane region and a cytoplasmic Ser-Thr kinase domain. The binding of BR to the extracellular domain induces phosphorylation and heterodimerization of BRI1 and BRI1-ASSOCIATED RECEPTOR KINASE 1 (BAK1), another leucine-rich repeat receptor kinase. BAK1 is expressed in all plant tissues, and despite BAK1 not being involved in BR binding, it does promote BR-induced signaling by physically associating with BRI1 [57,62,63].

Nowadays, it is well known that molecular docking is a cost-effective computational approach that provides reliable results with easy parameterization that makes it a widely used tool for simulation of binding processes of a ligand to its receptor, thus revealing the steric and electrostatic complementarity between them.

The elucidation of the crystal structure of the BRI1/BAK1 complex in the absence and presence of brassinolide (PDB: 4M7e) [61,64,65] has prompted several molecular docking studies to gain further insight into the way that BRs structure determines the bioactivity of BR analogs [16,38,66]. Herein, due to the clear relationship observed between the structural characteristics of different synthesized BR analogs and brassinolide (**1**), we have decided to carry out molecular docking studies on the crystal structure of the activation of the BRI1-BAK1 complex (PDB 4M7E) induced by brassinolide.

Therefore, all synthesized BR analogs were docked into the hydrophobic brassinolide-binding surface groove to determine the most likely binding mode, key interactions within the active site, and binding free energy for each of them. The crystallized inhibitor was removed and then redocked into the active site using the same protocol to validate the docking procedure and to ensure that the inhibitor binds exactly to the active site cleft. Furthermore, the root mean square deviation (RMSD) of the redocked ligand was calculated with respect to the reference crystallized ligand, obtaining a value of 0.56 Å for the best conformation. Thus, both binding energy and RMSD values provide a clear knowledge of ligand stability relative to the protein and its binding site. Hence, pose clustering was carried out based on RMSD values and docking score values obtained for each pose.

The binding mode and affinity of all compounds were analyzed, and it was found that the best docking poses share a common binding pattern into the hydrophobic groove located between the insertion domain and the concave side of the solenoid. An analysis of docking results shows that docked molecules and co-crystallized ligands occupy the active site in a similar way, namely with bulky methyl groups deeply buried into the hydrophobic surface groove by docking in two cavities at the bottom. The highest Glide scores range from −9.67 to −13.17 kcal mol^−1^ (see Table S2, Supplementary Material).

Also, these results show that these compounds adopt this orientation into the binding site, even though the polar groups and aromatic rings are not properly oriented to achieve favorable interactions. Therefore, to solve this drawback, the flexibility of the binding site has been considered by incorporating an Induced-Fit Docking (IFD) protocol into docking studies. This is based on Glide software that accurately predicts ligand binding modes and concomitant structural changes in the receptor. Therefore, to optimize the network of protein–ligand interactions, as compared to previous docking, the binding modes of all compounds were regenerated through the Induced-Fit Docking protocol.

**Figure 5:**
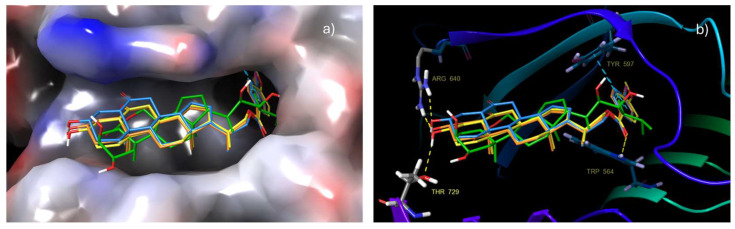
Superimposition of compounds **17**, **21** and **22** with brassinolide: (**a**) Aromatic ring of the benzoate group is buried in the same hydrophobic pocket where the brassinolide side alkyl chain fits. (**b**) Hydrogen bonding interactions and π–π stacking interactions of the aromatic ring with the Tyr 597 residue are observed (target: PDB: 4M7e).

Induced-Fit Docking (IFD) analysis of compounds **17**, **21** and **22** strongly suggests that these compounds share a common binding mode and adopt a brassinolide-like orientation when bound to the surface groove in BRI1-LRR, which supports initial docking results (Figure 5a). Likewise, these compounds show a key π–π stacking interaction between the benzoate ring attached to C22 and the Tyr 597 residue existing in the helical.

Additionally, it is also possible that benzoate groups interact with the Trp 564 residue, located around the binding site, by π–π stacking or by hydrogen bond formation between the benzoate carbonyl group and the protonated nitrogen of this amino acid. The formation of either of these interactions will depend on the ligand–protein complex conformation. In this sense, it is worth emphasizing that alkyl chains with substituted benzoate groups are completely buried in the hydrophobic pocket where the alkyl chain of brassinolide (**1**) is housed. Most of the hydrophobic interactions and Van der Waals contacts, formed by these analogs at the surface grove, involve highly conserved hydrophobic residues formed by side chains of Lys 601, Thr 729, Asn 705, Ile 706, Ile 682, Phe 681, Met 657, Ser 647, Leu 615 Trp 564, Tyr 599, Tyr 597, Ile 540, Trp 516 and Tyr 642, which along with π–π stacking interactions play a predominant role in protein–ligand recognition. Application of IFD leads to the highest Glide scores for the most stable conformations of all compounds, i.e., −10.17 to −13.17 kcal mol^−1^ (see Table S2, Supplementary Material). This increase in Glide score value suggests that these interactions as well as ligand orientation, like that adopted by **1**, provide a stabilizing effect on the brassinolide groove binding surface. Hydrophobic interactions with Thr 63 and Phe 60 residues can be observed as well, indicating that compounds **17**, **21** and **22** are interacting with BAK1 (LRR), and therefore forming a more stable BRI1-BAK1 complex in the same way that cocrystallized ligand does. The stabilizing effect of the benzoate group on the interactions of BR analogs with the receptor complex has been previously reported [16,18,37].

Further analysis of results obtained for these compounds revealed that A ring makes minor hydrophobic interactions with the concave surface, whereas the hydroxyl at C-3 can form two stable hydrogen bonds with Thr 729 and Arg 640 residues, reinforcing the interactions around the contact zone (Figure 5b). On the other hand, the B ring interacts tightly with Tyr residue 642 and with Tyr residue 599. Finally, rings C and D closely stack to residues of Ph 681 and Ph 60.

In summary, alkyl chains of analogs with the benzoate group are in the same hydrophobic pocket where the brassinolide (**1**) alkyl chain is housed; hence, π–π stacking interactions between the benzoate group and aromatic groups located in the binding site are produced. These interactions added to hydrogen bonding observed for these analogs explain the activity shown by these analogs on inhibition of root growth in Arabidopsis, acting identically to or like **1**. It is worth mentioning that these compounds exhibit the highest activities in the RLI test at 1 × 10^−8^ M. These results are in line with those previously reported.

### 2.4. Molecular Dynamic Simulations

To investigate the dynamic behavior of proteins and the reliability of results obtained previously, docking protocols have been combined with accurate molecular dynamics (MD) simulation techniques. MD simulations were performed on complexes of compounds **21** and **22**, which gave the highest GlideScore values in the IFD study, for 50 ns in an explicit aqueous solution environment with periodic boundary conditions. These simulations provided minute details about the motion of each atom over the simulation time. OPLS4 force field and TIP3P solvent model, employing the Desmond simulation package seamlessly integrated into the Maestro software, were used.

**Figure 6:**
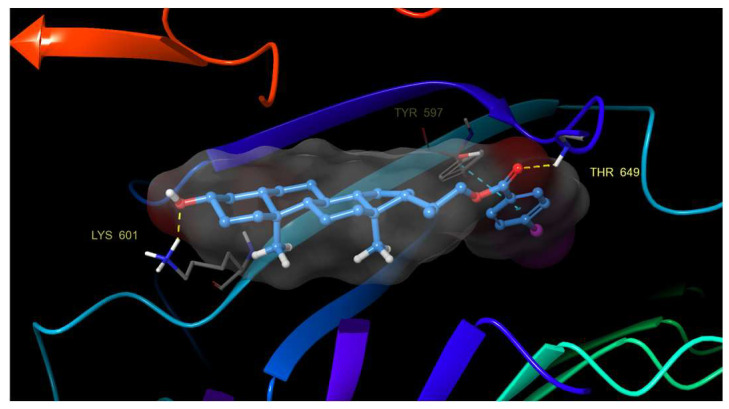
Hydrogen bonding and π–π stacking interactions of compound **21** with residues of the BRI1-BAK1 complex (PDB 4M7E).

An analysis of the trajectories obtained during the simulation, as well as the study of protein–ligand contacts, reveal that both compounds, **21** and **22**, do not leave the contact area on the surface groove and remain in the same orientation throughout the simulation process. Additionally, a series of new and previously unobserved hydrogen bonding interactions between the hydroxyl group and Lys 601, His 61 and Asn 705 residues were detected. Also, two other hydrogen bonds between the benzoate carbonyl group with Thr 649 and Ser 647 residues were observed (Figure 6).

These hydrogen bonds are added to those previously mentioned, and π–π stacking interactions prevent compounds from releasing from the binding site. This finding confirms the importance of aromatic rings on the activity of these analogs.

**Figure 7:**
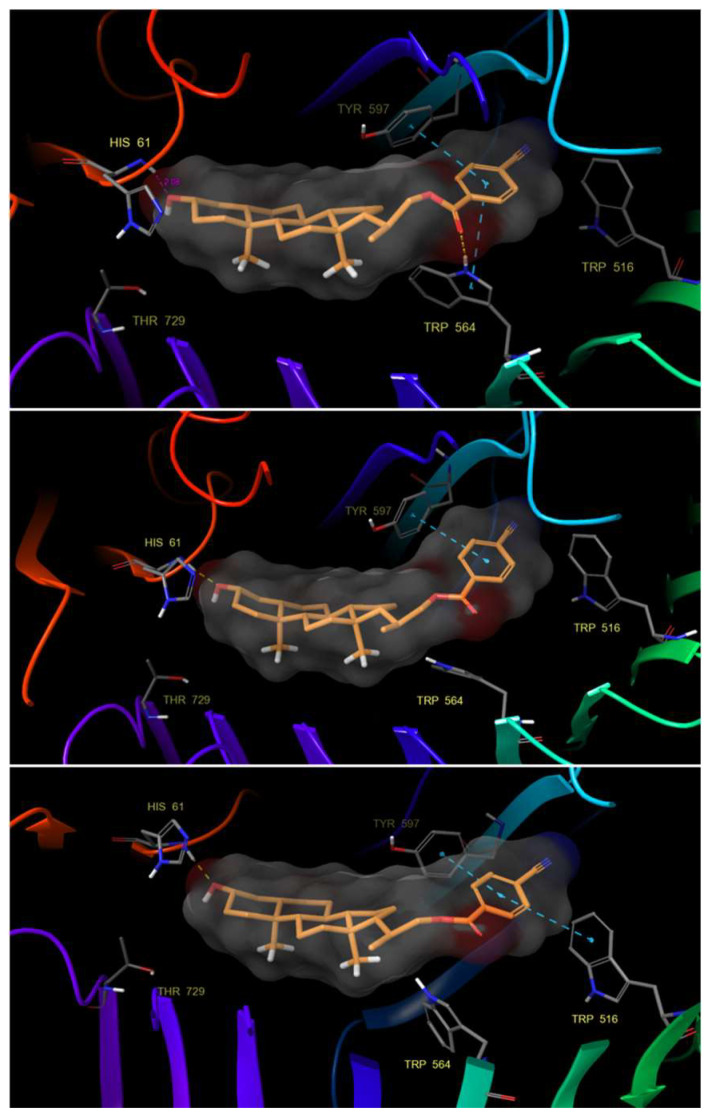
Hydrogen bond and π–π stacking interactions of compound **22** with residues of the BRI1-BAK1 complex (PDB 4M7E).

Although the results obtained from IFD analysis indicate that hydrophobic interactions are very similar for all compounds, the molecular dynamics processes show that compound **22** interacts strongly and stably through a hydrogen bond with the His 61 residue for almost the entire simulation time (Figure 7).

Likewise, we can observe how the aromatic ring of the benzoate group substituted in *para* position by a nitrile group is stabilized by π–π stacking interactions with the residues of Tyr 597, Trp 516 and Trp 564. The latter can form a hydrogen bond between its protonated nitrogen and the benzoate carbonyl group, creating a network of hydrophobic and hydrogen bonding interactions that are important for protein–ligand recognition. It is worth highlighting that this stabilization allows compound **22** to adopt a convex shape that can interact with the His 61 residue efficiently and adapt properly to the hydrophobic pocket.

**Figure 8:**
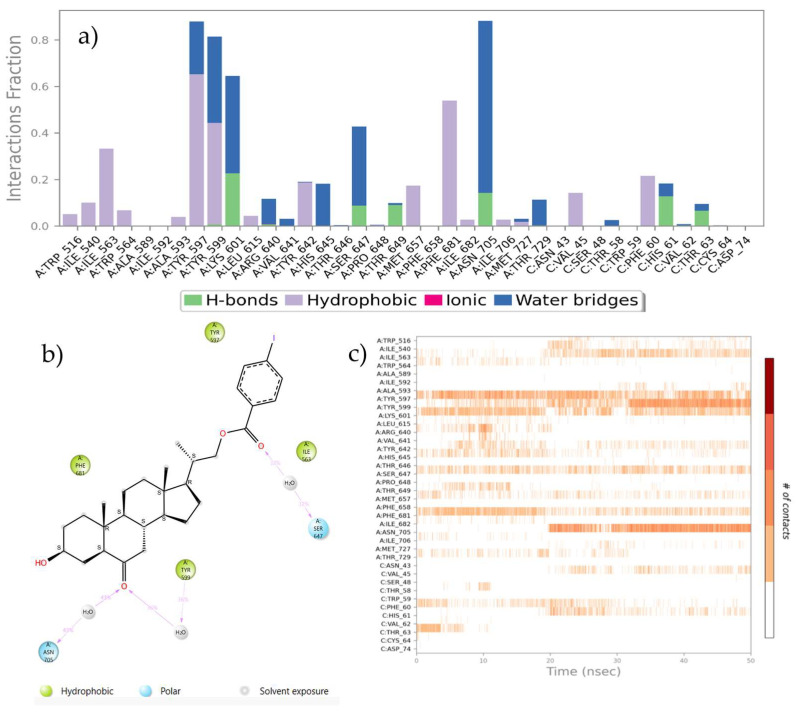
(**a**) Stacked bar chart of BRI1-BAK1 complex (PDB 4M7E) interaction with compound **21**. (**b**) Interactions that occur more than 30.0% of the simulation time in the selected trajectory. (**c**) A timeline representation of the interactions and contacts.

**Figure 9:**
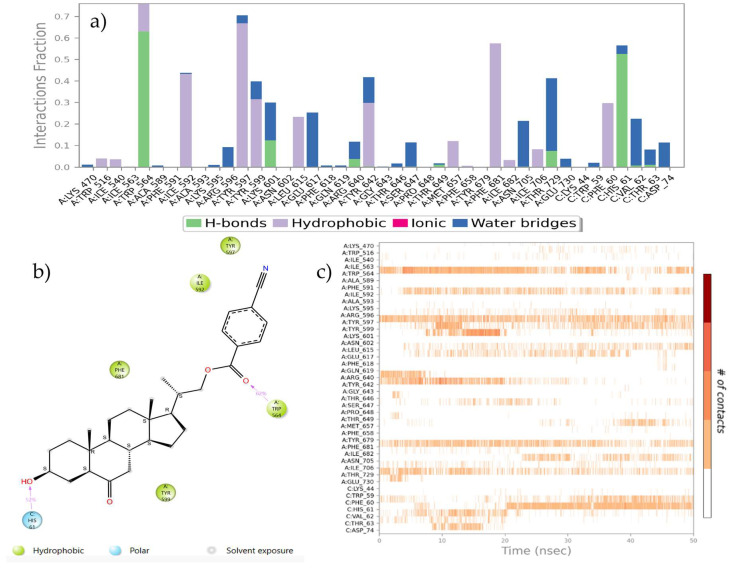
(**a**) The stacked bar charts of BRI1-BAK1 complex (PDB 4M7E) interaction with compound **22**. (**b**) Interactions that occur more than 30.0% of the simulation time in the selected trajectory. (**c**) A timeline representation of the interactions and contacts.

Protein–ligand interactions are observed as interaction fractions throughout the molecular dynamic simulation. Stacked bar charts are normalized along the path; for example, a value of 0.7 suggests that a specific interaction is maintained for 70% of the simulation time (Figure 8a and Figure 9a). In this way, the contribution of each amino acid residue can be determined, as well as the respective interaction responsible for complex stabilization.

The nitrile group plays different roles in its interaction with the receptor. In some cases, nitrile simply polarizes the density of adjacent electrons, while in other cases nitrile is a key component for molecular recognition. The nitrile group has a powerful electron-withdrawing nature in addition to being a strong hydrogen bond acceptor with a significant solvation capacity. Also, nitrile mimics halide polarization and often it is an excellent halogen bioisostere, and being smaller than bromine or iodine, nitrile is capable of achieving better contact with amino acids present in the active site [67].

Therefore, this model could help to explain the biological activity of this compound.

## 3. Materials and Methods

### 3.1. General

All chemical reagents were obtained from commercial suppliers and used as received. Melting points were determined on an SMP3 apparatus (Stuart-Scientific, now Merck KGaA, Darmstadt, Germany) and are uncorrected. A detailed description of conditions used to register Fourier transform infrared spectra ^1^H, ^13^C, ^13^C DEPT-135, sel. gs 1D ^1^H NOESY, gs 2D HSQC and gs 2D HMBC NMR spectra have been given elsewhere [37]. High-resolution mass spectra (HRMS-ESI) were recorded in a Bruker Daltonik. Analysis of reaction products was performed with the following relevant parameters: dry temperature, 180 °C; nebulizer 0.4 bar; dry gas, 4 L/min; and spray voltage, 4.5 kV at positive mode. The accurate mass measurements were performed at a resolving power: 140,000 FWHM at range *m*/*z* 50–1300. For analytical TLC, silica gel 60 in a 0.25 mm layer was used and TLC spots were detected by heating after spraying with 25% H_2_SO_4_ in H_2_O. Chromatographic separations were carried out on a glass column with silica gel 60 (230–400 mesh) and using EtOAc-hexane as eluent with increasing polarity gradient. All organic extracts were dried over anhydrous magnesium sulfate and evaporated under reduced pressure, below 40 °C.

### 3.2. Synthesis

#### 3.2.1. Methyl (20*S*)-3β-acetoxypregn-5-ene-20-carboxylate (**24**)

To a solution of bisnorcholenic acid 3β-acetate (**23**) (3.02 g, 7.77 mmol) in 30 mL of ether, 180 mL of ethereal CH_2_N_2_ was added. The reaction mixture was kept under constant stirring and room temperature for 6 h. The end of the reaction was verified by TLC, the mixture was then concentrated to dryness under reduced pressure. The solid obtained was recrystallized with ether/CH_2_Cl_2_ (1:1) system. Compound **24** (3.12 g, 99.8% yield) was a colorless solid (m.p. = 147.9–152.8 °C (144–146 °C [39]). IR_νmax_ (cm^−1^): 3060 (C=C-H); 2938 (CH_3_-); 2898 (CH_2_-); 2848 (CH_2_-); 1734 (C=O); 1715 (C=O); 1466 (CH_2_-); 1386 (CH_3_-); 1262 and 1065 (C-O). ^1^H NMR (400.1 MHz, CDCl_3_): δ (ppm) = 5.30 (1H, bd, *J* = 5.1 Hz, H-6); 4.56–4.48 (1H, m, H-3); 3.57 (3H, s, OCH_3_); 2.42 (1H, dq, *J* = 10.8 and 6.9 Hz, H-20); 2.27–2.23 (2H, m, H-4); 1.96 (3H, s, CH_3_CO); 1.12 (3H, d, *J* = 6.9 Hz, H-21); 0.95 (3H, s, H-19); 0.63 (3H, s, H-18). ^13^C NMR (100.6 MHz, CDCl_3_): δ (ppm) = 177.03 (C-22); 170.24 (CH_3_CO); 139.42 (C-5); 122.28 (C-6); 73.68 (C-3); 56.05 (C-14); 51.12 (OCH_3_); 52.67 (C-17); 49.76 (C-9); 42.26 (C-13); 42.19 (C-20); 39.30 (C-12); 37.90 (C-4); 36.79 (C-1); 36.38 (C-10); 31.62 (C-7); 31.67 (C-8); 27.56 (C-2); 26.95 (C-16); 24.15 (C-15); 21.22 (CH_3_CO); 20.78 (C-11); 19.12 (C-19); 16.94 (C-21); 11.84 (C-18). IR and ^1^H NMR spectroscopic data were consistent with those reported [39,43].

#### 3.2.2. Methyl (20*S*)-3β-acetoxypregn-5β,6β-epoxy-20-carboxylate (**25**)

KMnO_4_ (6.30 g 39.87 mmol) and Fe_2_(SO_4_)_3_.nH_2_O (3.20 g, 7.41 mmol) were finely grounded in a mortar, 0.6 mL of H_2_O was added and the mixture was placed in a round bottom flask containing CH_2_Cl_2_ (15 mL). Then, a solution of compound **24** (1.309 g, 3.252 mmol) in CH_2_Cl_2_ (15 mL) was added followed by the addition of tert-butyl alcohol (1.5 mL). Then, the reaction mixture was under constant stirring and room temperature for 2 h. The end of the reaction was verified by TLC, the mixture was then filtered on silica gel (26 g) and EtOAc (60 mL). The solvent was evaporated under reduced pressure and the crude was recrystallized with an ether/CH_2_Cl_2_ (1:1) system. Compound **25** (1.262 g, 3.015 mmol 92.7% yield) was a colorless powder (m.p. = 136.0–140.0 °C). IR_νmax_ (cm^−1^): 2848 (CH_3_-); 2870 (CH_2_-); 1736 (C=O); 1457 (CH_2_-); 1366 (CH_3_-); 1242 y 1036 (C-O). ^1^H NMR (400.1 MHz, CDCl_3_): δ (ppm) = 4.77–4.69 (1H, m, H-3); 3.60 (3H, s, OCH_3_); 3.04 (1H, bd, *J* = 2.1 Hz, H-6); 2.41–2.23 (1H, m, H-20); 1.99 (3H, s, CH_3_CO); 1.14 (3H, d, *J* = 7.0 Hz, H-21); 0.974 (3H, s, H-19); 0.625 (3H, s, H-18). ^13^C NMR (100.6 MHz, CDCl_3_): δ (ppm) = 177.13 (C-22); 170.38 (CH_3_CO); 71.18 (C-3); 63.34 (C-6); 62.38 (C-5); 55.64 (C-14); 52.81 (C-17); 51.37 (OCH_3_); 50.79 (C-9); 42.28 (C-20); 42.26 (C-13); 39.43 (C-12); 37.87 (C-4); 36.55 (C-1); 34.96 (C-10); 32.31 (C-7); 29.66 (C-8); 27.09 (C-2); 26.98 (C-16); 24.13 (C-15); 21.75 (C-11); 21.20 (CH_3_CO); 16.99 (C-21); 16.94 (C-19); 11.82 (C-18).

#### 3.2.3. Methyl (20*S*)-3β-acetoxypregn-5α-bromo-6β-hydroxy-20-carboxylate (**26**)

To a solution of epoxide **25** (1.83 g, 4.377 mmol) in 69 mL of CH_2_Cl_2_, 25 mL of HBr (48% *w*/*w*) was added. The reaction mixture was stirred constantly at room temperature for 4 h. The end of the reaction was verified by TLC, the mixture was then neutralized by slow addition of 20 mL of a saturated NaHCO_3_ solution. Another 10 mL of CH_2_Cl_2_ was added and the organic phase was washed with a saturated solution of NaCl (3 × 30 mL) and water (3 × 20 mL). The organic phase was dried over MgSO_4_ and filtered; then, the solvent was evaporated under reduced pressure. The crude was re-dissolved in CH_2_Cl_2_ (3.0 mL) and chromatographed on silica gel with EtOAc/cyclohexane (80:20) mixture. The fractions containing the product were combined, the solvent evaporated under reduced pressure and the crude was recrystallized with a CH_2_Cl_2_/hexane (1:1) system. Compound **26** (1.04 g, 2.09 mmol, 47.7% yield) was a colorless powder (m.p. = 159.8–161.3 °C). IR_νmax_ (cm^−1^): 3486 (O-H); 2943 (CH_3_-); 2873 (CH_2_-); 1734 (C=O); 1713 (C=O); 1460 (CH_2_-); 1374 (CH_3_-); 1240 and 1033 (C-O). ^1^H NMR (400.1 MHz, CDCl_3_): δ (ppm) = 5.54–5.45 (1H, m, H-3); 4.20 (1H, bt, *J* = 2.9 Hz, H-6); 3.66 (3H, s, OCH_3_); 2.52 (1H, dd, *J* = 13.6 and 10.7 Hz, H-4β); 2.44 (1H, ddd, *J* = 10.3, 6.8 and 6.6 Hz, H-20); 2.22–2.16 (1H, m, H-4α); 2.05 (3H, s, CH_3_CO); 1.34 (3H, s, H-19); 1.21 (3H, d, *J* = 6.8 Hz, H-21); 0.716 (3H, s, H-18). ^13^C NMR (100.6 MHz, CDCl_3_): δ (ppm) = 177.22 (C-22); 170.40 (CH_3_CO); 86.42 (C-5); 75.68 (C-6); 72.06 (C-3); 55.30 (C-14); 52.83 (C-17); 51.32 (OCH_3_); 47.37 (C-9); 42.77 (C-13); 42.42 (C-20); 40.34 (C-10); 39.40 (C-12); 38.39 (C-4); 35.08 (C-1); 34.51 (C-7); 30.61 (C-8); 27.07 (C-16); 26.32 (C-2); 24.09 (C-15); 21.22 (C-11); 21.33 (CH_3_CO); 17.94 (C-19); 17.09 (C-21); 12.35 (C-18).

#### 3.2.4. Methyl (20*S*)-3β-acetoxypregn-5α-bromo-6-oxo-20-carboxylate (**27**)

To a solution of **26** (0.20 g, 0.403 mmol) in CH_2_Cl_2_ (5 mL) and acetone (25 mL), 0.64 mL of Jones reagent was added by slow dripping. The reaction mixture was stirred slowly for 2 h at room temperature and the end of the reaction was verified by TLC. Then, 3 mL of methanol (to destroy excess oxidizing agent) was added, and the reaction mixture was stirred for another 10 min; then, the solvent was evaporated under reduced pressure. Another 20 mL of CH_2_Cl_2_ was added and the organic phase was washed with a saturated solution of NaCl (3 × 10 mL) and water (2 × 10 mL). The organic phase was dried over NaSO_4_ and filtered; then, the solvent was evaporated under reduced pressure and the crude was re-dissolved in CH_2_Cl_2_ (5 mL) and chromatographed on silica gel with EtOAc/cyclohexane (85:15) mixture. The fractions containing the product were combined, the solvent evaporated under reduced pressure and the crude was recrystallized with a CH_2_Cl_2_/hexane (1:1) system. Compound **27** (0.200 g, 0.403 100% yield) was a colorless powder (m.p. = 159.9–162.0 °C). IR_νmax_ (cm^−1^): 2943 (CH_3_-); 2870 (CH_2_-); 1736 (C=O); 1713 (C=O); 1460 (CH_2_-); 1374 (CH_3_-); 1233 y 1042 (C-O). ^1^H NMR (400.1 MHz, CDCl_3_): δ (ppm) = 5.36–5.27 (1H, m, H-3); 3.64 (3H, s, OCH_3_); 3.16 (1H, dd, *J* = 14.0 and 12.0 Hz, H-7 α); 2.27 (1H, dd, *J* = 14.0 and 6.0 Hz, H-7β); 2.00 (3H, s, CH_3_CO); 1.18 (3H, d, *J* = 8.0 Hz, H-21); 0.98 (3H, s, H-19); 0.66 (3H, s, H-18). ^13^C NMR (100.6 MHz, CDCl_3_): δ (ppm) = 203.64 (C-6); 176.99 (C-22); 170.27 (CH_3_CO); 79.46 (C-5); 70.88 (C-3); 55.71 (C-14); 52.64 (C-17); 51.37 (OCH_3_); 47.20 (C-9); 43.03 (C-13); 42.61 (C-10); 42.30 (C-20); 40.26 (C-7); 38.98 (C-12); 36.12 (C-8); 34.76 (C-4); 30.36 (C-1); 26.92 (C-16); 25.98 (C-2); 23.79 (C-15); 21.63 (C-11); 21.23 (CH_3_CO); 17.07 (C-21); 14.46 (C-19); 12.24 (C-18).

#### 3.2.5. Methyl (20*S*)-3β-acetoxy-5α-pregn-6-oxo-20-carboxylate (**28**)

A mixture of the bromoketone **27** (5.642 g, 11.37 mmol), zinc powder (14.15 g, 216.37 mmol) and glacial acetic acid (400.0 mL) was stirred under reflux for 4 h. The end of the reaction was verified by TLC; then, the reaction mixture was cooled at room temperature and later filtered. The acidic solution was neutralized by slow addition of 80 mL of a saturated NaHCO_3_ solution, until pH = 8.0 and extracted with CH_2_Cl_2_ (2 × 50 mL), washed with water (2 × 30 mL), dried over Na_2_SO_4_ and filtered. Then, the solvent was evaporated under reduced pressure and the crude was re-dissolved in CH_2_Cl_2_ (3 mL) and chromatographed on silica gel with EtOAc/cyclohexane (80:20) mixture. The fractions containing the product were combined, the solvent evaporated under reduced pressure and the crude was recrystallized with a CH_2_Cl_2_/hexane (1:1) system. Compound **28** (3.514 g, 8.42 mmol, 74.1% yield) was a colorless powder (m.p. = 157.8–162.9 °C). IR_νmax_ (cm^−1^): 2946 (CH_3_-); 2874 (CH_2_-); 1733 (C=O); 1713 (C=O); 1446 (CH_2_-); 1374 (CH_3_-); 1238 and 1036 (C-O). ^1^H NMR (400.1 MHz, CDCl_3_): δ (ppm) = 4.68–4.60 (1H, m, H-3); 3.62 (3H, s, OCH_3_); 2.44–2.36 (1H, m, H-17); 2.29 (1H, dd, *J* = 16.0 and 4.0 Hz, H-7α); 2.25 (1H, dd, *J* = 14.0 and 4.0 Hz, H-5); 2.00 (3H, s, CH_3_CO); 1.17 (3H, d, *J* = 8.0 Hz, H-21); 0.75 (3H, s, H-19); 0.66 (3H, s, H-18). ^13^C NMR (100.6 MHz, CDCl_3_): δ (ppm) = 209.96 (C-6); 177.05 (C-22); 170.50 (CH_3_CO); 72.71 (C-3); 56.39 (C-5); 56.17 (C-14); 53.68 (C-9); 52.73 (C-20); 51.32 (OCH_3_); 46.46 (C-7); 42.96 (C-13); 42.27 (C-17); 40.83 C-10); 39.13 (C-12); 37.78 (C-8); 36.32 (C-1); 26.91 (C-16); 26.76 (C-2); 26.05 (C-4); 23.94 (C-15); 21.34 (C-11); 21.26 (CH_3_CO) 17.03 (C-21); 12.97 (C-19); 12.13 (C-18).

#### 3.2.6. Methyl (20*S*)-3β-hydroxy-5α-pregn-6-oxo-20-carboxylate (**29**)

A solution of **28** (2.049 g, 4.897 mmol) and NaHCO_3_ (1.976 g, 19.73 mmol) in MeOH/H_2_O (200/20 mL) was stirred under reflux for 6 h. The end of the reaction was verified by TLC; then, the solvent was evaporated under reduced pressure. Then, 50 mL of a solution of HCl (pH = 5) was added, and the reaction mixture was stirred for another 10 min. The acidic solution was extracted with 50 mL of CH_2_Cl_2_. The organic phase was washed with water (2 × 30 mL), dried over Na_2_SO_4_ and filtered. Then, the solvent was evaporated under reduced pressure and the crude was recrystallized with an EtOAc/hexane system. Compound **29** (1.665 g, 4.434 mmol, 90.5% yield) was obtained a colorless powder (m.p. = 169.2–170.0 °C). IR_νmax_ (cm^−1^): 3504–3393 (O-H); 2943 (CH_3_-); 2848 (CH_2_-); 1733 (C=O); 1710 (C=O); 1446 (CH_2_-); 1358 (CH_3_-); 1259 y 1037 (C-O). ^1^H NMR (400.1 MHz, CDCl_3_): δ (ppm) = 3.63 (3H, s, OCH_3_); 3.59–3.52 (1H, m, H-3); 2.45–2.37 (1H, m, H-20); 2.29 (1H, dd, *J* = 13.2 and 4.5 Hz, H-7); 2.19 (1H, bd, *J* = 10.9 Hz, H-5); 1.17 (3H, d, *J* = 6.8 Hz, H-21); 0.73 (3H, s, H-19); 0.66 (3H, s, H-18). ^13^C NMR (100.6 MHz, CDCl_3_): δ (ppm) = 210.64 (C-6); 177.15 (COO); 70.53 (C-3); 56.69 (C-5); 56.24 (C-14); 53.76 (C-9); 52,72 (C-17); 51.35 (OCH_3_); 46.53 (C-7); 42.96 (C-13); 42.28 (C-20); 40.85 (C-10); 39,18 (C12); 37.76 (C-8); 36.58 (C-1); 30.58 (C-16); 29.92 (C-4); 26.92 (C-15); 23.95 (C-2); 21.38 (C-11); 17.02 (C-21); 13.08 (C-19); 12.13 (C-18).

#### 3.2.7. Methyl (20*S*)-3β-hydroxy-5α-pregn-6-dioxolan-20-carboxylate (**30**)

Ethylene glycol (3.24 mL, 57.94 mmol) and TsOH (0.1234 g, 0.6487 mmol) were added sequentially to a solution of **29** (1.03 g, 2.7427 mmol) in C_6_H_6_ (25 mL). The solution was refluxed for 2.5 h, using a Dean–Stark apparatus. The solution was cooled, quenched with saturated aqueous NaHCO_3_ (ca. 60 mL) and extracted with Et_2_O (3 × 30 mL). The combined organic extracts were dried (MgSO_4_) and evaporated to afford compound **30** (0.873 g, 2.0809 mmol, 75.9% yield) was a colorless powder (m.p. = 205.6–206.9 °C). IR _νmax_ (cm^−1^): 3446 (O-H); 2943 (CH_3_-); 2871 (CH_2_-); 1735 (C=O); 1446 (CH_2_-); 1383 (CH_3_-); 1276 y 1068 (C-O). ^1^H NMR (400.1 MHz, CDCl_3_): δ (ppm) = 3.96–8.86 (3H, m, ketal); 3.78–3.73 (1H, m, ketal); 3.62 (3H, s, O-CH_3_); 3.60–3.54 (1H, m, H-3); 2.44–2.36 (1H, m, H-20); 1.16 (3H, d, *J* = 7.1 Hz, H-21); 0.92 (3H, s, H-19); 0.67 (3H, s, H-18). ^13^C NMR (100.6 MHz, CDCl_3_): δ (ppm) = 177.29 (COO); 109.52 (C-6); 71.44 (C-3); 65.46 and 64.22 (O-(CH_2_)_2_-O); 55.53 (C-14); 53.49 (C-9); 52.86 (C-17); 51.30 (O-CH_3_); 50.61 (C-5); 42.63 (C-13); 42.41 (C-20); 41.29 (C-7); 39.49 (C-12); 38.14 (C-1); 36.78 (C-10); 33.36 (C-8); 31.05 (C-4); 29.06 (C-16); 27.05 (C-15); 24.18 (C-2); 20.98 (C-11); 17.03 (C-21); 14.18 (C-19); 12.17 (C-18).

#### 3.2.8. 3β-22-dihydroxy-5α-cholan-23,24-dinor-6-oxa (**31**)

A solution of the ester **30** (1 g, 2.4904 mmol) in THF (90 mL) was added to a stirred suspension of LiAlH_4_ (22 mL, 2M) in THF. The mixture was stirred at room temperature for 1.5 h and subsequently treated with AcOEt (20 mL). Later, it was treated with a HCl solution (5%) (50 mL) and stirred for 1 h. The organic layer was extracted with AcOEt (2 × 50 mL) and washed with water (3 × 50 mL). The organic layer was dried (Na_2_SO_4_) and evaporated. The residue was chromatographed on SiO_2_ with hexane–EtOAc (9:1 ⇒ 1:1) to give diol **31** (0.656 g, 1.99 mmol, 79.9% yield) was a colorless powder (m.p.= 169.3–172.5 °C). IR _νmax_ (cm^−1^): 3600–3100 (O-H); 3000–2850 (CH_3_-) and (CH_2_-); 1712 (C=O); 1070 (C-O). ^1^H NMR (400.1 MHz, CDCl_3_): δ (ppm) = 3.63 (1H, dd, *J* = 10.5 and 3.2 Hz, H-22a); 3.61–3.53 (1H, m, H-3); 3.38 (1H, dd, *J* = 10.5 and 6.7 Hz, H-22b); 2.31 (1H, dd, *J* = 13.0 and 4.5 Hz, H-7); 2.20 (1H, bdd, *J* = 12.6 and 2.7 Hz, H-5); 2.03 (1H, bdt, *J* = 12.7, 6.5 and 4.0 Hz, H-12); 1.95 (1H, bt, *J* = 13.0 Hz, H-7); 1.05 (3H, d, *J* = 6.5 Hz, H-21); 0.75 (3H, s, H-19); 0.68 (3H, s, H-18). ^13^C NMR (100.6 MHz, CDCl_3_): δ (ppm) = 210.78 (C-6); 70.65 (C-3); 67.81 (C-22); 56.74 (C-5); 56.46 (C-14); 53.85 (C-9); 52,30 (C-17); 46.66 (C-7); 43.02 (C-13); 40.90 (C-10); 39,30 (C12); 38.58 (C-20); 37.88 (C-8); 36.62 (C-1); 30.66 (C-16); 30.01 (C-4); 27.51 (C-15); 24.04 (C-2); 21.47 (C-11); 16.69 (C-21); 13.11 (C-19); 12.06 (C-18).

#### 3.2.9. 3β-hydroxy-5α-cholan-6-oxo-23,24-dinor-22-(4-substituted)benzoate-22-yl (**15**–**22**)

General procedure: Compound **31** was dissolved in DCM (20 mL) and pyridine (0.3 mL). Later, DMAP (15 mg) and *p*-PhCOCl were added with slow stirring at room temperature. The end of the reaction was verified by TLC (3 h); solvent volume was reduced to about 10 mL, and then EtOAc (20 mL) was added. The organic layer was washed with 5% KHSO_4_ (2 × 5 mL) and water (2 × 10 mL), dried over Na_2_SO_4_ and filtered. The solvent was evaporated under reduced pressure. The crude was redissolved in DCM (5 mL) and chromatographed on silica gel with PE/EtOAc mixtures of increasing polarity (9:1 → 4:6) [14].

3β-hydroxy-5α-cholan-6-oxo-23,24-dinor-22-benzoate-22-yl (**15**)

Compound **31** (0.11 g, 0.317 mmol); PhCOCl (0.25 mL, 2.152 mmol). Compound **15** (0.0677 g, 0.150 mmol, 47.4% yield) was obtained as a colorless solid (m.p. = 183.8–184.5 °C). IR_νmax_ (cm^−1^): 3450 (O-H); 3064 (=C-H); 2981–2827 (CH_3_-) and (CH_2_-); 1716 (C=O); 1682 (C=O conjugate); 1601 (C=C); 1275 (C-O ester) y 1068 (C-O alcohol). ^1^H NMR (400.1 MHz, CDCl_3_): δ (ppm) = 8.03 (2H, d, *J* = 8.2 Hz, ArH-2′); 7.55 (1H, bt, *J* = 7.6 Hz, ArH-4′); 7.43 (2H, bt, *J* = 7.6 Hz, ArH-3′); 4.30 (1H, dd, *J* = 10.7 and 3.6 Hz, H-22a); 4.04 (1H, dd, *J* = 10.7 and 7.2 Hz, H-22b); 3.59–3.53 (1H, m, H-3); 2.31 (1H, dd, *J* = 13.1 and 4.5 Hz, H-7α); 2.19 (1H, dd, *J* = 12.2 and 2.7 Hz, H-5); 2.05 (1H, tt, *J* = 13.1, 6.4 and 2.9 Hz, H-12); 1.11 (3H, d, *J* = 6.6 Hz, H-21); 0.74 (3H, s, H-19); 0.70 (3H, s, H-18). ^13^C NMR (100.6 MHz, CDCl_3_): δ (ppm) = 210.75 (C-6); 166.64 (Ar-CO_2_); 132.78 (ArC-4′), 130.41 (ArC-1′); 129.43 (ArC-2′); 128.29 (ArC-3′); 70.50 (C-3); 69.77 (C-22); 56.69 (C-5); 56.34 (C-14); 53.75 (C-9); 52.76 (C-17); 46.55 (C-7); 43.09 (C-13); 40.83 (C-10); 39.26 (C12); 37.80 (C-8); 36.58 (C-1); 35.88 (C-20); 30.55 (C-16); 29.89 (C-4); 27.50 (C-15); 23.99 (C-2); 21.42 (C-11); 17.25 (C-21); 13.06 (C-19); 12.02 (C-18). HRMS-ESI (positive mode): *m*/*z* calculated for C_29_H_40_O_4_: 453.2999 [M]^+^; found 453.2998 [M + H]^+^.

3β-hydroxy-5α-cholan-6-oxo-23,24-dinor-22-(4-methyl)benzoate-22-yl (**16**)

Compound **31** (0.12 g, 0.344 mmol); 4-CH_3_PhCOCl (0.1 mL, 0.756 mmol). Compound **16** (0.0524 g, 0.112 mmol, 32.6% yield) was obtained as a colorless solid (m.p. = 198.7–199.6 °C). IR_νmax_ (cm^−1^): 3521 (O-H); 3080 (=C-H); 2941–2864 (CH_3_-) and (CH_2_-); 1711 (C=O); 1680 (C=O); 1608 (C=C); 1273 (C-O) y 1066 (C-O). ^1^H NMR (400.1 MHz, CDCl_3_): δ (ppm) = 7.92 (2H, d, *J* = 8.0 Hz, ArH-2′); 7.24 (2H, d, *J* = 8.0 Hz, ArH-3′); 4.29 (1H, dd, *J* = 10.8 and 3.5 Hz, H-22a); 4.04 (1H, dd, *J* = 10.8 and 7.13 Hz, H-22b); 3.61–3.54 (1H, m, H-3); 2.41 (3H, s, CH_3_-Ar); 2.32 (1H, dd, *J* = 13.2 and 4.5 Hz, H-7α); 2.20 (1H, dd, *J* = 12.5 and 2.5 Hz, H-5); 2.06 (1H, dt, *J* = 12.5, 6.1 and 3.2 Hz, H-12); 1.11 (3H, d, *J* = 6.9 Hz, H-21); 0.76 (3H, s, H-19); 0.72 (3H, s, H-18). ^13^C NMR (100.6 MHz, CDCl_3_): δ (ppm) = 210.70 (C-6); 166.78 (Ar-CO_2_); 143.47 (ArC-4′); 129.52 (ArC-3′); 129.05 (ArC-2′); 127.75 (ArC-1′); 70.63 (C-3); 69.63 (C-22); 56.74 (C-5); 56.40 (C-14); 53.84 (C-9); 52.86 (C-17); 46.62 (C-7); 43.15 (C-13); 40.88 (C-10); 39.32 (C12); 37.86 (C-8); 36.64 (C-1); 35.95 (C-20); 30.65 (C-16); 30.00 (C-4); 27.55 (C-15); 24.05 (C-2); 21.63 (C-28); 21.48 (C-11); 17.20 (C-21); 13.11 (C-19); 12.07 (C-18). HRMS-ESI (positive mode): *m*/*z* calculated for C_30_H_42_O_4_: 467.3156 [M]^+^; found 467.3153 [M + H]^+^.

3β-hydroxy-5α-cholan-6-oxo-23,24-dinor-22-(4-methoxy)benzoate-22-yl (**17**)

Compound **31** (0.12 g, 0.344 mmol); 4-CH_3_OPhCOCl (0.1 mL, 0.739 mmol). Compound **17** (0.0680 g, 0.141 mmol, 40.9% yield) was obtained as a colorless solid (m.p. = 200.6–201.3 °C). IR_νmax_ (cm^−1^): 3518 (O-H); 3074 (=C-H); 2937–2858 (CH_3_-) and (CH_2_-); 1707 (C=O); 1680 (C=O); 1603 (C=C); 1255 (C-O) and 1068 (C-O). ^1^H NMR (400.1 MHz, CDCl_3_): δ (ppm) = 7.98 (2H, d, *J* = 8.8 Hz, ArH-2′); 6.91 (2H, d, *J* = 8.8 Hz, ArH-3′); 4.26 (1H, dd, *J* = 10.8 and 3.5 Hz, H-22a); 4.00 (1H, dd, *J* = 10.8 and 7.1 Hz, H-22b); 3.84 (3H, s, OCH_3_); 3.59–3.52 (1H, m, H-3); 2.30 (1H, dd, *J* = 13.2 and 4.5 Hz, H-7α); 2.18 (1H, d, *J* = 12.5 Hz, H-5); 2.04 (1H, t, *J* = 13.4 Hz, H-12); 1.09 (3H, d, *J* = 6.9 Hz, H-21); 0.73 (3H, s, H-19); 0.69 (3H, s, H-18). ^13^C NMR (100.6 MHz, CDCl_3_): δ (ppm) = 210.75 (C-6); 166.40 (Ar-CO_2_); 163.20 (ArC-4′); 131.43 (ArC-2′); 122.85 (ArC-1′); 113.53 (ArC-3′); 70.50 (C-3); 69.48 (C-22); 56.69 (C-5); 56.33 (C-14); 55.35 (C-28); 53.77 (C-9); 52.82 (C-17); 46.55 (C-7); 43.09 (C-13); 40.83 (C-10); 39.26 (C12); 37.80 (C-8); 36.59 (C-1); 35.90 (C-20); 30.55 (C-16); 29.89 (C-4); 27.49 (C-15); 23.99 (C-2); 21.42 (C-11); 17.25 (C-21); 13.05 (C-19); 12.01 (C-18). HRMS-ESI (positive mode): *m*/*z* calculated for C_30_H_42_O_5_: 483.3105 [M]^+^; found 483.3106 [M + H]^+^.

3β-hydroxy-5α-cholan-6-oxo-23,24-dinor-22-(4-chloro)benzoate-22-yl (**18**)

Compound **31** (0.12 g, 0.344 mmol); 4-ClPhCOCl (0.15 mL, 1.170 mmol). Compound **18** (0.00692 g, 0.142 mmol, 41.3% yield) was obtained as a colorless solid (m.p. = 210.3–212.0 °C). IR_νmax_ (cm^−1^): 3535 (O-H); 3085–3070 (=C-H); 2941–2864 (CH_3_-) and (CH_2_-); 1718 (C=O); 1693 (C=O); 1593 (C=C); 1275 (C-O) y 1061 (C-O).; ^1^H NMR (400.1 MHz, CDCl_3_): δ (ppm) = 7.96 (2H, d, *J* = 8.6 Hz, ArH-2′); 7.41 (2H, d, *J* = 8.6 Hz, ArH-3′); 4.30 (1H, dd, *J* = 10.7 and 3.4 Hz, H-22a); 4.04 (1H, dd, *J* = 10.7 and 7.2 Hz, H-22b); 3.61–3.53 (1H, m, H-3); 2.32 (1H, dd, *J* = 13.2 and 4.4 Hz, H-7α); 2.20 (1H, dd, *J* = 12.3 and 2.1 Hz, H-5); 2.05 (1H, dt, *J* = 12.7 and 3.1 Hz, H-12); 1.11 (3H, d, *J* = 6.7 Hz, H-21); 0.75 (3H, s, H-19); 0.71 (3H, s, H-18). ^13^C NMR (100.6 MHz, CDCl_3_): δ (ppm) = 210.66 (C-6); 165.82 (Ar-CO_2_); 139.26 (ArC-4′), 130.87 (ArC-2′); 128.90 (ArC-1′); 128.69 (ArC-3′); 70.60 (C-3); 70.06 (C-22); 56.74 (C-5); 56.38 (C-14); 53.81 (C-9); 52.81 (C-17); 46.59 (C-7); 43.15 (C-13); 40.87 (C-10); 39.31 (C12); 37.83 (C-8); 36.63 (C-1); 35.91 (C-20); 30.63 (C-16); 29.98 (C-4); 27.54 (C-15); 24.03 (C-2); 21.46 (C-11); 17.27 (C-21); 13.11 (C-19); 12.06 (C-18). HRMS-ESI (positive mode): *m*/*z* calculated for C_29_H_39_O_4_Cl: 487.2610 [M]^+^; found 487.2607 [M + H]^+^.

3β-hydroxy-5α-cholan-6-oxo-23,24-dinor-22-(4-bromo)benzoate-22-yl (**19**)

Compound **31** (0.12 g, 0.344 mmol); 4-BrPhCOCl (0.238 g, 1.084 mmol). Compound **19** (0.0471 g, 0.0886 mmol, 25.7% yield) was obtained as a colorless solid (m.p. = 210.0–211.6 °C). IR_νmax_ (cm^−1^): 3521 (O-H); 3080–3060 (=C-H); 2941–2864 (CH_3_-) and (CH_2_-); 1716 (C=O); 1690 (C=O); 1583 (C=C); 1273 (C-O) and 1068 (C-O). ^1^H NMR (400.1 MHz, CDCl_3_): δ (ppm) = 7.89 (2H, d, *J* = 8.6 Hz, ArH-2′); 7.58 (2H, d, *J* = 8.6 Hz, ArH-3′); 4.30 (1H, dd, *J* = 10.8 and 3.7 Hz, H-22a); 4.05 (1H, dd, *J* = 10.8 and 7.3 Hz, H-22b); 3.62–3.54 (1H, m, H-3); 2.33 (1H, dd, *J* = 13.1 and 4.4 Hz, H-7α); 2.20 (1H, dd, *J* = 12.6 and 2.4 Hz, H-5); 2.05 (1H, dt, *J* = 12.6, 6.1 and 3.2 Hz, H-12); 1.11 (3H, d, *J* = 6.6 Hz, H-21); 0.76 (3H, s, H-19); 0.72 (3H, s, H-18). ^13^C NMR (100.6 MHz, CDCl_3_): δ (ppm) = 210.64 (C-6); 165.96 (Ar-CO_2_); 131.70 (ArC-3′), 131.03 (ArC-2′); 129.37 (ArC-1′); 127.94 (ArC-4′); 70.64 (C-3); 70.09 (C-22); 56.75 (C-5); 56.40 (C-14); 53.83 (C-9); 52,83 (C-17); 46.61 (C-7); 43.17 (C-13); 40.88 (C-10); 39,33 (C12); 37.85 (C-8); 36.64 (C-1); 35.92 (C-20); 30.66 (C-16); 30.00 (C-4); 27.56 (C-15); 24.05 (C-2); 21.48 (C-11); 17.28 (C-21); 13.12 (C-19); 12.07 (C-18). HRMS-ESI (positive mode): *m*/*z* calculated for C_29_H_39_O_4_Br: 550.2354 [M + NH_4_]^+^; found 550.2348 [M + NH_4_]^+^.

3β-hydroxy-5α-cholan-6-oxo-23,24-dinor-22-(4-fluoro)benzoate-22-yl (**20**)

Compound **31** (0.158 g, 0.453 mmol); 4-FPhCOCl (0.11 mL, 0.906 mmol). Compound **20** (0.098 g, 0.208 mmol, 46.0% yield) was obtained as a colorless solid (m.p. = 209.0–210.5 °C). IR ν_max_ (cm^−1^): 3545 (O-H); 3084 (=C-H); 2976–2862 (CH_3_-) and (CH_2_-); 1712 (C=O); 1690 (C=O); 1599 (C=C); 1263 (C-O) and 1066 (C-O). ^1^H NMR (400.1 MHz, CDCl_3_): δ (ppm) = 8.04 (2H, dd, *J* = 8.8 and 5.5 Hz, ArH-2′); 7.11 (2H, t, *J* = 8.8 Hz, ArH-3′); 4.30 (1H, dd, *J* = 10.9 and 3.5 Hz, H-22a); 4.04 (1H, dd, *J* = 10.9 and 7.2 Hz, H-22b); 3.62–3.55 (1H, m, H-3); 2.31 (1H, bdd, *J* = 12.9 and 2.5 Hz, H-7α); 2.20 (1H, bd, *J* = 12.1 Hz, H-5); 2.05 (1H, bd, *J* = 11.2 Hz, H-12); 1.10 (3H, d, *J* = 6.7 Hz, H-21); 0.76 (3H, s, H-19); 0.72 (3H, s, H-18). ^13^C NMR (100.6 MHz, CDCl_3_): δ (ppm) = 210.71 (C-6); 165.71 (Ar-CO_2_); 165.66 (d, ^1^*J*_C-F_ = 253.5 Hz, ArC-4′), 131.98 (d, ^2^*J*_C-F_ = 9.5 Hz, ArC-2′); 126.68 (d, ^4^*J*_C-F_ = 2.1 Hz, ArC-1′); 115.46 (d, ^3^*J*_C-F_ = 22.3 Hz, ArC-3′); 70.55 (C-3); 69.94 (C-22); 56.73 (C-5); 56.37 (C-14); 53.80 (C-9); 52.81 (C-17); 46.58 (C-7); 43.14 (C-13); 40.86 (C-10); 39,30 (C12); 37.83 (C-8); 36.62 (C-1); 35.91 (C-20); 30.60 (C-16); 29.95 (C-4); 27.53 (C-15); 24.02 (C-2); 21.45 (C-11); 17.26 (C-21); 13.09 (C-19); 12.04 (C-18). HRMS-ESI (positive mode): *m*/*z* calculated for C_29_H_39_O_4_F: 471.2905 [M]^+^; found 471.2904 [M + H]^+^.

3β-hydroxy-5α-cholan-6-oxo-23,24-dinor-22-(4-iodine)benzoate-22-yl (**21**)

Compound **31** (0.0556 g, 0.1595 mmol); 4-IPhCOCl (0.1275 g, 0.4785 mmol). Compound **21** (0.0351 g, 0.0607 mmol, 38.1% yield) was obtained as a colorless solid (m.p. = 205.3–207.9 °C). IR ν_max_ (cm^−1^): 3397 (O-H); 2940–2863 (CH_3_-) and (CH_2_-); 1716 (C=O); 1684 (C=O); 1590 (C=C); 1273 (C-O) and 1060 (C-O). ^1^H NMR (400.1 MHz, CDCl_3_): δ (ppm) = 7.79 (2H, dd, *J* = 8.6 and 3.8 Hz, ArH-3′); 7.72 (2H, dd, *J* = 8.6 and 3.8 Hz, ArH-2′); 4.29 (1H, dd, *J* = 10.7 and 3.5 Hz, H-22a); 4.03 (1H, dd, *J* = 10.7 and 7.2 Hz, H-22b); 3.60–3.54 (1H, m, H-3); 2.31 (1H, dd, *J* = 13.1 and 4.4 Hz, H-7α); 2.19 (1H, dd, *J* = 12.6 and 2.4 Hz, H-5); 2.04 (1H, dt, *J* = 12.6, 6.4 and 3.2 Hz, H-12); 1.09 (3H, d, *J* = 6.6 Hz, H-21); 0.75 (3H, s, H-19); 0.71 (3H, s, H-18). ^13^C NMR (100.6 MHz, CDCl_3_): δ (ppm) = 210.71 (C-6); 166.16 (Ar-CO_2_); 137.68 (ArC-3′), 130.92 (ArC-2′); 129.89 (ArC-4′); 100.58 (ArC-1′); 70.56 (C-3); 70.05 (C-22); 56.72 (C-5); 56.34 (C-14); 53.77 (C-9); 52,78 (C-17); 46.57 (C-7); 43.13 (C-13); 40.85 (C-10); 39,28 (C12); 37.81 (C-8); 36.60 (C-1); 35.87 (C-20); 30.59 (C-16); 29.93 (C-4); 27.52 (C-15); 24.01 (C-2); 21.43 (C-11); 17.24 (C-21); 13.09 (C-19); 12.04 (C-18). HRMS-ESI (positive mode): *m*/*z* calculated for C_29_H_39_O_4_I: 579.1966 [M]^+^; found 579.1959 [M + H]^+^.

3β-hydroxy-5α-cholan-6-oxo-23,24-dinor-22-(4-cyan)benzoate-22-yl (**22**)

Compound **31** (0.0547 g, 0.1570 mmol); 4-CNPhCOCl (0.08 g, 0.483 mmol). Compound **22** (0.033 g, 0.069 mmol, 44.0% yield) was obtained as a colorless solid (m.p. = 218.2–221.0 °C). IR ν_max_ (cm^−1^): 3432 (O-H); 2942–2866 (CH_3_-) and (CH_2_-); 1716 (C=O); 1474 (C=C); 1277 (C-O) and 1062 (C-O). ^1^H NMR (400.1 MHz, CDCl_3_): δ (ppm) = 8.12 (2H, dd, *J* = 8.6 and 3.3 Hz, ArH-2′); 7.74 (2H, dd, *J* = 8.6 and 3,3 Hz, ArH-3′); 4.34 (1H, dd, *J* = 10.7 and 3.3 Hz, H-22a); 4.08 (1H, dd, *J* = 10.7 and 7.2 Hz, H-22b); 3.61–3.53 (1H, m, H-3); 2.31 (1H, dd, *J* = 13.2 and 4.5 Hz, H-7α); 2.20 (1H, dd, *J* = 12.6 and 2.6 Hz, H-5); 2.05 (1H, ddt, *J* = 12.3, 6.0 and 2.6 Hz, H-12); 1.11 (3H, d, *J* = 6.7 Hz, H-21); 0.75 (3H, s, H-19); 0.70 (3H, s, H-18). ^13^C NMR (100.6 MHz, CDCl_3_): δ (ppm) = 210.63 (C-6); 164.99 (Ar-CO_2_); 134.24 (CN); 132.20 (ArC-3′), 129.98 (ArC-2′); 117.95 (ArC-4′); 116.26 (ArC-1′); 70.62 (C-3); 70.55 (C-22); 56.72 (C-5); 56.35 (C-14); 53.77 (C-9); 52,74 (C-17); 46.56 (C-7); 43.16 (C-13); 40.85 (C-10); 39,29 (C12); 37.80 (C-8); 36.60 (C-1); 35.86 (C-20); 30.60 (C-16); 29.94 (C-4); 27.54 (C-15); 24.00 (C-2); 21.43 (C-11); 17.24 (C-21); 13.09 (C-19); 12.04 (C-18). HRMS-ESI (positive mode): *m*/*z* calculated for C_30_H_39_O_4_N: 478.2952 [M]^+^; found 478.2947 [M + H]^+^.

### 3.3. Biological Activity

#### 3.3.1. Rice Lamina Inclination Test (RLIT)

All BR analogs (**15**–**22**) were tested by RLIT according to a modified procedure previously described [46,47]. Seeds of a local Rice cultivar (*Oryza sativa* L) of the *Zafiro* variety (provided by INIA-QUILAMAPU, CHILE) were sterilized and then synchronized by soaking in sterile distilled water for 24 h, sown and grown for about 10 days in pots with substrate, maintained at 22 °C, 16 h light/8 h dark photoperiod, and 50–60% relative humidity in a plant growth chamber. Uniformly growing rice plants were selected to cut an approximately 8 cm segment containing the second internode of the rice lamina. These segments were then placed in a Petri dish containing sterile distilled water (60 mL) and BR analogs at different concentrations (1 × 10^−8^; 1 × 10^−7^ and 1 × 10^−6^ M) or brassinolide (APExBIO) used as a positive control. The negative control included dimethyl sulfoxide (DMSO) which was used as a solvent to dilute BR analogs. Segments were then left to incubate for 72 h at 25 °C in the dark to finally measure the angle of inclination of the unrolled sheet between the leaf and the sheath. Each treatment consisted of 10 independent replicates; with these data, significant differences between the positive control and treatments were evaluated. Mean values with the least significant difference (*p* < 0.05; Student’s *t*-test) were considered. Images were taken with a Leica EZ4HD stereo microscope with camera software.

#### 3.3.2. Inhibition of Root and Hypocotyl Elongation in *Arabidopsis thaliana* Seedlings

Seeds of *Arabidopsis thaliana* L. (Heyhn.) (Columbia ecotype, Col-0; referred to Arabidopsis) were sterilized and stratified for 3 days at 4 °C on Murashige and Skoog culture medium (1 %*w*/*v* sucrose with pH adjusted to 5.8) with a 1 × 10^−6^ M concentration of **1** and BR analogs (**15**–**22**). Germinated seeds were then transferred to a plant growth chamber in an upright position, at 22 °C in a 16 h/8 h light–dark cycle for 5 days. For the hypocotyl elongation test, stratified seeds were exposed to light for 6 h and then transferred to a plant growth chamber under the same above conditions, except that they were kept in darkness. Both roots and hypocotyls, from independent experiments, were straightened on solid medium plates and scanned with an Epson high-resolution scanner. Total root and hypocotyl lengths were measured with ImageJ/FIJI 1.46r image processing software (National Institutes of Health) (http://rsbweb.nih.gov/ij/; accessed on 20 February 2022). For each treatment, 15 seedlings in two biological replicates were analyzed. Mean values with at least significant difference (*p* < 0.05; Student’s *t*-test) were considered [38].

### 3.4. Computational Studies

#### 3.4.1. Protein Preparation and Molecular Docking

Docking studies were performed using Glide v9.7. The crystal structure of the complex formed by BRI1-BAK1 and natural ligand brassinolide (BL) was extracted from the Protein Data Bank (PDB code 4M7E). PDB structures were prepared for docking using the Protein Preparation Workflow (Schrodinger, LLC, New York, NY, USA, 2022), accessible from the Maestro program (Maestro, version 13.4, Schrodinger, LLC, New York, NY, USA, 2022). Substrate and water molecules were removed beyond 5 Å, bond corrections were applied to the co-crystallized ligand, and exhaustive sampling of group orientations was performed. Finally, the receptor was optimized in Maestro 13.0 by using an OPLS4 force field before the docking study. The receptor grid was generated using the prepared protein, with the docking grid centered at the bound ligand. Three-dimensional structures of ligands to be docked were generated and prepared using LigPrep implemented in Maestro 13.4 (LigPrep, Schrodinger, LLC: New York, NY, USA, 2022). The geometries were optimized using an OPLS4 force field. Docking of ligands was performed using the extra precision mode (XP) and without any constraints [68]. Flexibility of pocket residues near the docked ligand was considered. The generated ligand poses were evaluated with an empirical scoring function implemented in Glide [69], GlideScore, which was used to estimate binding affinity and rank ligands [70]. The XP Pose Rank was used to select the best-docked pose for each ligand.

#### 3.4.2. Induced-Fit Docking

Induced-Fit Docking (IFD) experiment was carried out to confer flexibility to protein side chains, allowing adjustment and optimization of ligand binding interactions within the active site. Ligand docking was carried out following the IFD procedure [71], which is based on the Glide search algorithm that uses the standard protocol, OPLS4 force field (Induced Fit Docking Protocol 2022, Glide version 9.7 [69], and Prime, Schrodinger, LLC, New York, NY, 2022). The centroid of co-crystallized ligand residue was selected as the center of the Glide grid (inner box side = 10 Å and outer box side = auto). Ligands were initially docked into the receptor by applying a scaling factor of 0.5 to both ligand and protein van der Waals radius. Up to 20 poses per ligand were collected, and side chains of residues within 5 Å of the ligand were refined with Prime. After Prime minimization of selected residues and ligand for each pose, a Glide redocking of each protein–ligand complex structure within 30 kcal/mol of the lowest energy structure was performed. Ligands were redocked into these newly generated receptor conformations, generating up to 10 poses using the extra precision mode (XP).

#### 3.4.3. Molecular Dynamics Simulation

Optimized Potentials for Liquid Simulations (OPLS4) [72,73] force field in Desmond Molecular Dynamic System was used to study the behavior of ligand-target complex. The docking resulting complexes were solvated with an orthorhombic box of TIP3P (Transferable Intermolecular Potential 3-Point) water [74] and counter ions were added, creating an overall neutral system simulating approximately 0.15 M NaCl. Ions were equally distributed in a water box. The final system was subjected to an MD simulation of up to 50 ns using the Desmond program [68]. The method selected was NPT (Noose–Hover chain thermostat at 300 K, Martyna–Tobias–Klein barostat method at 1.01325 bar with a relaxation time of 2 ps, isotropic coupling, and a 9 Å radius cut-off was used for coulombic short-range interaction) constraints were not applied. During the simulation process, the smooth-particle Mesh–Ewald method was used to calculate long-range electrostatic interactions. For multiple-time-step integration, RESPA (reversible reference system propagator algorithm) was applied to integrate the equation of motion with Fourier-space electrostatics computed every 6 fs, and all remaining interactions were computed every 2 fs [75]. MD simulations were carried out on these equilibrated systems for a period of 50 ns, frames of energy and trajectory were captured every 1.2 ps and 9.6 ps, respectively. The quality of MD simulations was assessed by the Simulation Event Analysis tool. Ligand–receptor interactions were identified using the Simulation Interaction Diagram tool.

## 4. Conclusions

A synthetic route is described that allows obtaining eight new BR analogs with 4-substituted benzoate at C-22 in the side chain (compounds **15**–**22**). This series of compounds has been designed to compare their bioactivity with that exhibited by similar compounds having hydroxyl groups at C2 and C3. The idea is to assess the role played by this group in the activity of BR analogs. Thus, synthesized BR analogs were tested for activity on different assays, namely, Rice Lamina Inclination Test, and Inhibition of Root and Hypocotyl Elongation of *Arabidopsis* Seedlings. Results of RLIT at 1 × 10^−8^ M suggest that growth activity of these BR analogs is like that shown by brassinolide. To assess the effect of hydroxyl group at C2 a comparison of activities previously reported at 1 × 10^−7^ M was performed. Results are not conclusive, i.e., the presence of -OH at C22 increases the activity of non-benzoylated analogs, whereas for no substituted benzoylated analogs the growth activity slightly decreases, and for analogs carrying F-benzoate groups the effect is null. On the other hand, no clear relationship between the chemical structure of BR analogs and bioactivity results obtained from both assays based on elongation inhibition of *Arabidopsis thaliana* seedlings. Finally, molecular docking and molecular dynamics studies were carried out with all new analogs. Results indicate that a brassinolide-like orientation is adopted for all of them, and the benzoate group interacts with the receptor complex giving energy binding values ranging between −10.17 and −13.17 kcal mol^−1^. Finally, the analog with nitrile group (compound **22**), which is the most active compound in the RLIT assay, can achieve better contact with amino acids present in the active site.

We believe that these results are in line with the idea that BR bioactivities should be explained by considering the total structure instead of individual structural requirements for activity. In this sense, molecular docking is a valuable tool for designing analogs that could be as active as natural BRs. However, binding to the active site is just one step in the complex mechanism of action of exogenous applied BR analogs.

## Figures and Tables

**Table 1:** Effect of BR analogs with 23,24-dinor-5α-cholane-type side chains on lamina inclination of rice seedlings. Brassinolide (**1**) was used as positive control.

Bending Angle between Laminae and Sheaths (°) (Degrees ± Standard Error) ^1^			
Compounds	1 × 10^−8^ M	1 × 10^−7^ M	1 × 10^−6^ M
**1**	22 ± 3.5 ^b^	31 ± 2.6 ^a^	71 ± 4.5 ^a^
**15**	21 ± 1.0 ^b^	18 ± 2.7 ^b^	24 ± 1.2 ^b^
**16**	23 ± 2.7 ^b^	10 ± 1.2 ^c^	18 ± 2.6 ^c^
**17**	33 ± 2.6 ^a^	15 ± 2.0 ^b^	5 ± 2.0 ^e^
**18**	12 ± 2.0 ^d^	19 ± 2.6 ^b^	9 ± 2.1 ^d^
**19**	15 ± 2.0 ^c^	19 ± 2.6 ^b^	3 ± 2.7 ^e^
**20**	16 ± 2.0 ^c^	21 ± 3.2 ^b^	20 ± 2.0 ^c^
**21**	38 ± 2.5 ^a^	24 ± 2.0 ^b^	6 ± 1.8 ^e^
**22**	37 ± 2.0 ^a^	9 ± 2.0 ^c^	18 ± 1.0 ^c^
**31**	21 ± 1.2 ^b^	14 ± 2.8 ^b^	4 ± 2.4 ^e^

^1^ These values represent the mean ± standard deviation of two independent experiments with at least six replicates each (*n* = 12). Average angle of negative control: 4 ± 0.5. Letters represent experiments with a significant difference between positive control (**1**) and analog treatments at the 0.05 significance level (Student’s *t*-test).

## Data Availability

Data are contained within the article or supplementary material.

## Supplementary Materials

The following supporting information can be downloaded at: https://www.mdpi.com/article/10.3390/ijms251810158/s1.

## References

[1] Bajguz A., Hayat S., Ahmad A. (2016). Brassinosteroids—Occurence and chemical structures in plants. Brassinosteroids: A Class of Plant Hormone.

[2] Oklestkova J., Rarova L., Kvasnica M., Strnad M. (2015). Brassinosteroids: Synthesis and biological activities. Phytochem. Rev..

[3] Bajguz A., Hayat S. (2009). Effects of brassinosteroids on the plant responses to environmental stresses. Plant Physiol. Biochem..

[4] Manghwar H., Hussain A., Ali Q., Liu F. (2022). Brassinosteroids (BRs) Role in Plant Development and Coping with Different Stresses. Int. J. Mol. Sci..

[5] Temmem O., Uguen D., De Cian A., Gruber N. (2002). Toward a total synthesis of brassinosteroids; structure assessment of the Ireland–Claisen products of geranyl and neryl esters. Tetrahedron Lett..

[6] Temmem O., Zoller T., Uguen D. (2002). Toward a total synthesis of brassinosteroids; stereoselective generation of the hydrindane ring system. Tetrahedron Lett..

[7] Aburatani M., Takeuchi T., Mori K. (1987). Facile Syntheses of Brassinosteroids: Brassinolide, Castasterone, Teasterone and Typhasterol. Agric. Biol. Chem..

[8] Back T.G., Blazecka P.G., Krishna M.V. (1993). A new synthesis of castasterone and brassinolide from stigmasterol—A concise and stereoselective elaboration of the side-chain from a C-22 aldehyde. Can. J. Chem..

[9] Khripach V.A., Zhabinskii V.N., de Groot A.E., Khripach V.A., Zhabinskii V.N., de Groot A.E. (1999). Chapter VII—Syntheses of natural BS. Brassinosteroids.

[10] Back T., Pharis R. (2004). Structure-Activity Studies of Brassinosteroids and the Search for Novel Analogues and Mimetics with Improved Bioactivity. J. Plant Growth Regul..

[11] Baron D.L., Luo W., Janzen L., Pharis R.P., Back T.G. (1998). Structure–activity studies of brassinolide B-ring analogues. Phytochem..

[12] Liu J., Zhang D., Sun X., Ding T., Lei B., Zhang C. (2017). Structure-activity relationship of brassinosteroids and their agricultural practical usages. Steroids.

[13] Tian W.S., Zhou W., Jiang B., Pan X.F. (1989). Studies on steroidal plant-growth regulator IX: The preparation of 22R- and 22S-24, 25, 26, 27, 28-penta-nor-brassinolides. Acta Chim. Sinica.

[14] Carvajal R., Gonzalez C., Olea A.F., Fuentealba M., Espinoza L. (2018). Synthesis of 2-Deoxybrassinosteroids Analogs with 24-nor, 22(S)-23-Dihydroxy-Type Side Chains from Hyodeoxycholic Acid. Molecules.

[15] Oyarce J., Aitken V., Gonzalez C., Ferrer K., Olea A.F., Parella T., Espinoza L. (2019). Synthesis and structural determination of new brassinosteroid 24-nor-5α-cholane type analogs. Molecules.

[16] Diaz K., Espinoza L., Carvajal R., Conde-Gonzalez M., Niebla V., Olea A.F., Coll Y. (2020). Biological Activities and Molecular Docking of Brassinosteroids 24-Norcholane Type Analogs. Int. J. Mol. Sci..

[17] Soto N., Ferrer K., Díaz K., González C., Taborga L., Olea A.F., Carrasco H., Espinoza L. (2021). Synthesis and Biological Activity of New Brassinosteroid Analogs of Type 24-Nor-5β-Cholane and 23-Benzoate Function in the Side Chain. Int. J. Mol. Sci..

[18] Ferrer K., Díaz K., Kvasnica M., Olea A.F., Cuellar M., Espinoza L. (2021). Synthesis of New Brassinosteroid 24-Norcholane Type Analogs Conjugated in C-3 with Benzoate Groups. Molecules.

[19] Voigt B., Schmidt J., Adam G. (1996). Synthesis of 24-epiteasterone, 24-epityphasterol and their B-homo-6a-oxalactones from ergosterol. Tetrahedron.

[20] Brosa C., Capdevila J.M., Zamora I. (1996). Brassinosteroids: A new way to define the structural requirements. Tetrahedron.

[21] Voigt B., Porzel A., Bruhn C., Wagner C., Merzweiler K., Adam G. (1997). Synthesis of 24-epicathasterone and related brassinosteroids with modified side chain. Tetrahedron.

[22] Seto H., Fujioka S., Koshino H., Yoshida S., Tsubuki M., Honda T. (1999). Synthesis and biological evaluation of extra-hydroxylated brassinolide analogs. Tetrahedron.

[23] Iglesias-Arteaga M., Gil R.P., Leliebre-Lara V., Martinez C.S.P., Manchado F. (1996). Synthesis and biological activity of (22R,25R)-5 alpha-furostan-2 alpha,3 alpha,26-triol. J. Chem. Res..

[24] Iglesias-Arteaga M., Gil R., Leliebre-Lara V., Coll-Manchado F., Pérez C.S., Rosado A. (1998). Synthesis of (25R)-5α-Spirostan-2α,3α,6β-triol Triacetate. Synth. Commun..

[25] Iglesias-Arteaga M., Gil R.P., Leliebre-Lara V., Martinez C.S.P., Manchado F. (1998). Synthesis of (22R,25R)-2 alpha,3 alpha,26-trihydroxy-5 alpha-furostanaone-6-one. Synth. Commun..

[26] Iglesias-Arteaga M., Gil R.P., Leliebre-Lara V., Martinez C.S.P., Manchado F., Perez A.R., Rios L.P. (1998). Synthesis of (22R,25R)-3 beta,26-dihydroxy-5 alpha-furostan-6-one. Synth. Commun..

[27] Iglesias-Arteaga M.A., PérezGil R., LeliebreLara V., CollManchado F., PérezMartínez C.S. (1998). Synthesis of (25R)-2α,3α-Epoxy-5α-Spirostan-6,23-Dione. Synth. Commun..

[28] Iglesias-Arteaga M.A., Martinez C.S.P., Manchado F.C. (1999). Synthesis and characterization of (25R)-2 alpha,3 alpha-epoxy-5 alpha-spirostan-12,23-dione. Synth. Commun..

[29] Iglesias-Arteaga M.A., Gil R.-P., Pérez-Martínez C.S., Coll-Manchado F. (2000). Synthetic Steroidal Sapogenins. Part III 23-Ketohecogenin and 23-Ketoisochiapagenin. Synth. Commun..

[30] Zhou W., Jiang B., Shen J. (1998). Synthesis of cholesteric lactones and analogs as plant growth regulators.

[31] Zhou W.S., Tian W.S. (1984). The Synthesis of Steroids Containing Structural Unit of A, B Ring of Brassinolide and Ecdysone from Hyodeoxycholic Acid. Acta Chim. Sinica.

[32] Kvasnica M., Oklestkova J., Bazgier V., Rarova L., Berka K., Strnad M. (2014). Biological activities of new monohydroxylated brassinosteroid analogues with a carboxylic group in the side chain. Steroids.

[33] Duran M.I., Gonzalez C., Acosta A., Olea A.F., Diaz K., Espinoza L. (2017). Synthesis of Five Known Brassinosteroid Analogs from Hyodeoxycholic Acid and Their Activities as Plant-Growth Regulators. Int. J. Mol. Sci..

[34] Diachkov M.V., Ferrer K., Oklestkova J., Rarova L., Bazgier V., Kvasnica M. (2021). Synthesis and Biological Activity of Brassinosteroid Analogues with a Nitrogen-Containing Side Chain. Int. J. Mol. Sci..

[35] Huang L.F., Zhou W.S. (1994). Studies on Steroidal Plant-Growth Regulators. Part 33. Novel Method for Construction of the Side-Chain of 23-Arylbrassinosteroids Via Heck Arylation and Asymmetric Dihydroxylation As Key Steps. J. Chem. Soc. Perkin Trans. 1.

[36] Korinkova P., Bazgier V., Oklestkova J., Rarova L., Strnad M., Kvasnica M. (2017). Synthesis of novel aryl brassinosteroids through alkene cross-metathesis and preliminary biological study. Steroids.

[37] Aitken V., Diaz K., Soto M., Olea A.F., Cuellar M.A., Nuñez M., Espinoza-Catalán L. (2024). New Brassinosteroid Analogs with 23,24-Dinorcholan Side Chain, and Benzoate Function at C-22: Synthesis, Assessment of Bioactivity on Plant Growth, and Molecular Docking Study. Int. J. Mol. Sci..

[38] Kvasnica M., Oklestkova J., Bazgier V., Rárová L., Korinkova P., Mikulík J., Budesinsky M., Béres T., Berka K., Lu Q. (2016). Design, synthesis and biological activities of new brassinosteroid analogues with a phenyl group in the side chain. Org. Biomol. Chem..

[39] Kohout L.J., Chodounská H., Macek T., Strnad M. (2000). Synthesis of (20S)-2α,3α-Dihydroxy-6-oxo-7-oxa-7a-homo-5α-pregnane-20-carboxylic Acid as a Brassinosteroid Part of Ligands for Binding to Affinity Chromatography Carriers. Collect. Czech. Chem. Commun..

[40] Iglesias-Arteaga M.A., Símuta-Lopez E.M., Xochihua-Moreno S., Viñas-Bravo O., Montiel Smith S., Meza Reyes S., Sandoval-Ramírez J. (2005). A Convenient Procedure for the Synthesis of 3β-Hydroxy-6-oxo-5α-steroids: Application to the Synthesis of Laxogenin. J. Mex. Chem. Soc..

[41] Rosado-Abon A., Romero-Avila M., Iglesias-Arteaga M.A. (2008). An unexpected and useful E-ring oxidative cleavage in furostanes. ARKIVOC.

[42] Hunter A.C., Priest S.-M. (2006). An efficient one-pot synthesis generating 4-ene-3,6-dione functionalised steroids from steroidal 5-en-3β-ols using a modified Jones oxidation methodology. Steroids.

[43] Cimino F.P., Núñez M.G., Rosado-Abón A., Amesty Á., Estévez-Braun A., Díaz K., Espinoza L.C., Iglesias-Arteaga M.A. (2023). Methyl esters of 23,24-Dinor-5α-cholan-22-oic acids as brassinosteroid Analogues. Synthesis, evaluation of plant growth promoting activity and Molecular docking. Steroids.

[44] Antonchick A.P., Schneider B., Zhabinskii V.N., Khripach V.A. (2004). Synthesis of [26,27-2H6]brassinosteroids from 23,24-bisnorcholenic acid methyl ester. Steroids.

[45] Yu G., Clive D.L. (2016). Conversion of cycloalk-2-enones into 2-methylcycloalkane-1,3-diones--assessment of various Tamao-Fleming procedures and mechanistic insight into the use of the Me3SiMe2Si unit. Org. Biomol. Chem..

[46] Wada K., Marumo S., Abe H., Morishita T., Nakamura K., Uchiyama M., Mori K. (1984). A Rice Lamina Inclination Test—A Micro-Quantitative Bioassay for Brassinosteroids. Agric. Biol. Chem..

[47] Li H., Wang H., Jang S. (2017). Rice Lamina Joint Inclination Assay. Bio-Protocol.

[48] Wang Y.-Q., Luo W.-H., Xu R.-J., Zhao Y.-J., Zhou W.-S., Huang L.-F., Shen J.-M. (1994). Biological Activity of Brassinosteroids and Relationship of Structure to Plant Growth Promoting Effects. Chin. Sci. Bull..

[49] Müssig C., Shin G.-H., Altmann T. (2003). Brassinosteroids Promote Root Growth in Arabidopsis. Plant Physiol..

[50] Planas-Riverola A., Gupta A., Betegón-Putze I., Bosch N., Ibañes M., Caño-Delgado A.I. (2019). Brassinosteroid signaling in plant development and adaptation to stress. Development.

[51] Lv B., Tian H., Zhang F., Liu J., Lu S., Bai M., Li C., Ding Z. (2018). Brassinosteroids regulate root growth by controlling reactive oxygen species homeostasis and dual effect on ethylene synthesis in Arabidopsis. PLoS Genet..

[52] González-García M.-P., Vilarrasa-Blasi J., Zhiponova M., Divol F., Mora-García S., Russinova E., Caño-Delgado A.I. (2011). Brassinosteroids control meristem size by promoting cell cycle progression in Arabidopsis roots. Development.

[53] Vukašinović N., Russinova E. (2018). BRexit: Possible Brassinosteroid Export and Transport Routes. Trends Plant Sci..

[54] Hola D., Hayat S., Yusuf M., Bhardwaj R., Bajguz A. (2019). Role of Brassinosteroids in the Plant Response to Drought: Do We Know Anything for Certain?. Brassinosteroids: Plant Growth and Development.

[55] Yin Y., Wang Z.-Y., Mora-Garcia S., Li J., Yoshida S., Asami T., Chory J. (2002). BES1 Accumulates in the Nucleus in Response to Brassinosteroids to Regulate Gene Expression and Promote Stem Elongation. Cell.

[56] Ryu H., Kim K., Cho H., Hwang I. (2010). Predominant Actions of Cytosolic BSU1 and Nuclear BIN2 Regulate Subcellular Localization of BES1 in Brassinosteroid Signaling. Mol. Cells.

[57] Nam K.H., Li J. (2002). BRI1/BAK1, a receptor kinase pair mediating brassinosteroid signaling. Cell.

[58] Wang X., Goshe M.B., Soderblom E.J., Phinney B.S., Kuchar J.A., Li J., Asami T., Yoshida S., Huber S.C., Clouse S.D. (2005). Identification and functional analysis of in vivo phosphorylation sites of the Arabidopsis BRASSINOSTEROID-INSENSITIVE1 receptor kinase. Plant Cell.

[59] Clouse S.D. (2011). Brassinosteroid signal transduction: From receptor kinase activation to transcriptional networks regulating plant development. Plant Cell.

[60] Kinoshita T., Caño-Delgado A., Seto H., Hiranuma S., Fujioka S., Yoshida S., Chory J. (2005). Binding of brassinosteroids to the extracellular domain of plant receptor kinase BRI1. Nature.

[61] She J., Han Z., Kim T.-W., Wang J., Cheng W., Chang J., Shi S., Wang J., Yang M., Wang Z.-Y. (2011). Structural insight into brassinosteroid perception by BRI1. Nature.

[62] Wang J., Jiang J., Wang J., Chen L., Fan S.-L., Wu J.-W., Wang X., Wang Z.-X. (2014). Structural insights into the negative regulation of BRI1 signaling by BRI1-interacting protein BKI1. Cell Res..

[63] Yang C.-J., Zhang C., Lu Y.-N., Jin J.-Q., Wang X.-L. (2011). The Mechanisms of Brassinosteroids’ Action: From Signal Transduction to Plant Development. Mol. Plant.

[64] Hothorn M., Belkhadir Y., Dreux M., Dabi T., Noel J.P., Wilson I.A., Chory J. (2011). Structural basis of steroid hormone perception by the receptor kinase BRI1. Nature.

[65] Sun Y., Han Z., Tang J., Hu Z., Chai C., Zhou B., Chai J. (2013). Structure reveals that BAK1 as a co-receptor recognizes the BRI1-bound brassinolide. Cell Res..

[66] Lei B., Liu J., Yao X. (2015). Unveiling the molecular mechanism of brassinosteroids: Insights from structure-based molecular modeling studies. Steroids.

[67] Fleming F.F., Yao L., Ravikumar P.C., Funk L., Shook B.C. (2010). Nitrile-Containing Pharmaceuticals: Efficacious Roles of the Nitrile Pharmacophore. J. Med. Chem..

[68] Friesner R.A., Murphy R.B., Repasky M.P., Frye L.L., Greenwood J.R., Halgren T.A., Sanschagrin P.C., Mainz D.T. (2006). Extra Precision Glide:  Docking and Scoring Incorporating a Model of Hydrophobic Enclosure for Protein−Ligand Complexes. J. Med. Chem..

[69] Glide (2022). Glide Software.

[70] Friesner R.A., Banks J.L., Murphy R.B., Halgren T.A., Klicic J.J., Mainz D.T., Repasky M.P., Knoll E.H., Shelley M., Perry J.K. (2004). Glide:  A New Approach for Rapid, Accurate Docking and Scoring. 1. Method and Assessment of Docking Accuracy. J. Med. Chem..

[71] Sherman W., Beard H.S., Farid R. (2006). Use of an induced fit receptor structure in virtual screening. Chem. Biol. Drug Des..

[72] Lu C., Wu C., Ghoreishi D., Chen W., Wang L., Damm W., Ross G.A., Dahlgren M.K., Russell E., Von Bargen C.D. (2021). OPLS4: Improving Force Field Accuracy on Challenging Regimes of Chemical Space. J. Chem. Theory Comput..

[73] Jorgensen W.L., Chandrasekhar J., Madura J.D., Impey R.W., Klein M.L. (1983). Comparison of simple potential functions for simulating liquid water. J. Chem. Phys..

[74] Bowers K.J., Dror R.O., Shaw D.E. (2006). The midpoint method for parallelization of particle simulations. J. Chem. Phys..

[75] Gibson D.A., Carter E.A. (1993). Time-reversible multiple time scale ab initio molecular dynamics. J. Phys. Chem..

