# Supplementary material for: Synthesis, Biological Activity, and Molecular-Docking Studies of New Brassinosteroid Analogs
Source: Int J Mol Sci. 2024 Sep 21;25(18):10158. doi: 10.3390/ijms251810158 (PMC11432311; doi:10.3390/ijms251810158)
Supplement: Supplementary file 1 [file ijms-25-10158-s001.zip › ijms-3154815-supplementary.pdf]

International Journal of *Molecular Sciences*

MDPI

*Supplementary Material*

# Synthesis, Biological Activity, and Molecular-Docking Studies of New Brassinosteroid Analogs

**María Nuñez [1], Yaowei Wang [2,3], Eugenia Russinova [2,3], Ana Estévez-Braun [4], Angel Amesty [4], Andrés F. Olea [5], Marco Mellado [6], Katy Díaz [1,*] and Luis Espinoza Catalán [1,*]**

[1] Departamento de Química, Universidad Técnica Federico Santa María, Avenida España 1680, Valparaíso 2340000, Chile; maria.nunezg@usm.cl
[2] Department of Plant Biotechnology and Bioinformatics, Ghent University, 9052 Ghent, Belgium; yaoweiwang@126.com (Y.W.); eugenia.russinova@psb.vib-ugent.be (E.R.)
[3] Center for Plant Systems Biology, VIB, 9052 Ghent, Belgium
[4] Departamento de Química Orgánica, Instituto Universitario de Bio-Orgánica Antonio González, Universidad de La Laguna, Avda. Astrofísico Francisco Sánchez 2, 38206 La Laguna, Tenerife, Spain; aestebra@ull.edu.es (A.E.-B.); aarnesty@ull.edu.es (A.A.)
[5] Grupo QBAB, Instituto de Ciencias Aplicadas, Facultad de Ingeniería, Universidad Autónoma de Chile, El Llano Subercaseaux 2801, Santiago 8900000, Chile; andres.olea@uautonoma.cl
[6] Facultad de Medicina y Ciencias de la Salud, Universidad Central de Chile, Santiago 8330507, Chile; marco.mellado@ucentral.cl
* Correspondence: katy.diaz@usm.cl (K.D.); luis.espinozac@usm.cl (L.E.C.); Tel.: +56-32-2654425 (L.E.C.)

**Citation:** Nuñez, M.; Wang, Y.; Russinova, E.; Estévez-Braun, A.; Amesty, A.; Olea, A.F.; Mellado, M.; Díaz, K.; Espinoza Catalán, L. Synthesis, Biological Activity, and Molecular-Docking Studies of New Brassinosteroid Analogs. *Int. J. Mol. Sci.* **2024**, *25*, 10158. https://doi.org/10.3390/ijms251810158

Academic Editors: Guzel Kudoyarova and Antonio Rescifina

Received: 29 July 2024
Revised: 29 August 2024
Accepted: 8 September 2024
Published: 21 September 2024

**Copyright:** © 2024 by the authors. Licensee MDPI, Basel, Switzerland. This article is an open access article distributed under the terms and conditions of the Creative Commons Attribution (CC BY) license (https://creativecommons.org/licenses/by/4.0/).

**Index** Pag.


**Figure S1:** IR spectrum of Methyl (20*S*)-3*β*-Acetoxypregn-5-ene-20-carboxylate (**24**)........ 1

**Figure S2:** $^{1}$H NMR spectrum of Methyl (20*S*)-3*β*-Acetoxypregn-5-ene-20-carboxylate (**24**)........ 2

**Figure S3:** $^{13}$C NMR spectrum of Methyl (20*S*)-3*β*-Acetoxypregn-5-ene-20-carboxylate (**24**)........ 3

**Figure S4:** 2D HSQC NMR spectrum of of Methyl (20*S*)-3*β*-Acetoxypregn-5-ene-20-carboxylate (**24**)........ 4

**Figure S5:** 2D HMBC NMR spectrum of of Methyl (20*S*)-3*β*-Acetoxypregn-5-ene-20-carboxylate (**24**)........ 5

**Figure S6:** IR spectrum of Methyl (20*S*)-3*β*-Acetoxypregn-5β,6β-epoxy-20-carboxylate (**25**)........ 6

**Figure S7:** $^{1}$H NMR spectrum of Methyl (20*S*)-3*β*-Acetoxypregn-5β,6β-epoxy-20-carboxylate (**25**)........ 7

**Figure S8:** 1D selective NOESY NMR spectrum of Methyl (20*S*)-3*β*-Acetoxypregn-5β,6β-epoxy-20-carboxylate (**25**).. 8

**Figure S9:** $^{13}$C NMR spectrum of Methyl (20*S*)-3*β*-Acetoxypregn-5β,6β-epoxy-20-carboxylate (**25**)........ 9

**Figure S10:** $^{13}$C DEPT-135 NMR spectrum of Methyl (20*S*)-3*β*-Acetoxypregn-5β,6β-epoxy-20-carboxylate (**25**)........ 10

**Figure S11:** 2D HSQC NMR spectrum of Methyl (20*S*)-3*β*-Acetoxypregn-5β,6β-epoxy-20-carboxylate (**25**)........ 11

**Figure S12:** 2D HMBC NMR spectrum of Methyl (20*S*)-3*β*-Acetoxypregn-5β,6β-epoxy-20-carboxylate (**25**)........ 12

**Figure S13:** IR spectrum of Methyl (20*S*)-3*β*-Acetoxypregn-5*α*-bromo-6β-hydroxy-20-carboxylate (**26**)........ 13

**Figure S14:** $^{1}$H NMR spectrum of Methyl (20*S*)-3*β*-Acetoxypregn-5*α*-bromo-6β-hydroxy-20-carboxylate (**26**)........ 14

**Figure S15:** $^{13}$C NMR spectrum of Methyl (20*S*)-3*β*-Acetoxypregn-5*α*-bromo-6β-hydroxy-20-carboxylate (**26**)........ 15

**Figure S16:** $^{13}$C DEPT-135 NMR spectrum of Methyl (20*S*)-3*β*-Acetoxypregn-5*α*-bromo-6β-hydroxy-20-carboxylate (**26**)........ 16

**Figure S17:** 2D HSQC NMR spectrum of Methyl (20*S*)-3*β*-Acetoxypregn-5*α*-bromo-6β-hydroxy-20-carboxylate (**26**).. 17

**Figure S18:** 2D HMBC NMR spectrum of Methyl (20*S*)-3*β*-Acetoxypregn-5*α*-bromo-6β-hydroxy-20-carboxylate (**26**).. 18

**Figure S19:** IR spectrum of Methyl (20*S*)-3*β*-Acetoxypregn-5*α*-bromo-6-oxo-20-carboxylate (**27**)........ 19

**Figure S20:** $^{1}$H NMR spectrum of Methyl (20*S*)-3*β*-Acetoxypregn-5*α*-bromo-6-oxo-20-carboxylate (**27**)........ 20

**Figure S21:** $^{13}$C NMR spectrum of Methyl (20*S*)-3*β*-Acetoxypregn-5*α*-bromo-6-oxo-20-carboxylate (**27**)........ 21

**Figure S22:** $^{13}$C DEPT-135 NMR spectrum of Methyl (20*S*)-3*β*-Acetoxypregn-5*α*-bromo-6-oxo-20-carboxylate (**27**)..... 22

**Figure S23:** 2D HSQC NMR spectrum of Methyl (20*S*)-3*β*-Acetoxypregn-5*α*-bromo-6-oxo-20-carboxylate (**27**)........ 23

**Figure S24:** 2D HMBC NMR spectrum of Methyl (20*S*)-3*β*-Acetoxypregn-5*α*-bromo-6-oxo-20-carboxylate (**27**)........ 24

**Figure S25:** IR spectrum of Methyl (20*S*)-3*β*-Acetoxy-5*α*-pregn-6-oxo-20-carboxylate (**28**)........ 25

**Figure S26:** $^{1}$H NMR spectrum of Methyl (20*S*)-3*β*-Acetoxy-5*α*-pregn-6-oxo-20-carboxylate (**28**)........ 26

**Figure S27:** $^{13}$C NMR spectrum of Methyl (20*S*)-3*β*-Acetoxy-5*α*-pregn-6-oxo-20-carboxylate (**28**)........ 27

**Figure S28:** $^{13}$C DEPT-135 NMR spectrum of Methyl (20*S*)-3*β*-Acetoxy-5*α*-pregn-6-oxo-20-carboxylate (**28**)........ 28

**Figure S29:** 2D HSQC NMR spectrum of Methyl (20*S*)-3*β*-Acetoxy-5*α*-pregn-6-oxo-20-carboxylate (**28**)........ 29

**Figure S30:** 2D HMBC NMR spectrum of Methyl (20*S*)-3*β*-Acetoxy-5*α*-pregn-6-oxo-20-carboxylate (**28**)........ 30

**Figure S31:** IR spectrum of Methyl (20*S*)-3*β*-hydroxy-5*α*-pregn-6-oxo-20-carboxylate (**29**)........ 31

**Figure S32:** $^{1}$H NMR spectrum of Methyl (20*S*)-3*β*-hydroxy-5*α*-pregn-6-oxo-20-carboxylate (**29**)........ 32

**Figure S33:** $^{13}$C NMR spectrum of Methyl (20*S*)-3*β*-hydroxy-5*α*-pregn-6-oxo-20-carboxylate (**29**)........ 33

**Figure S34:** $^{13}$C DEPT-135 NMR spectrum of Methyl (20*S*)-3*β*-hydroxy-5*α*-pregn-6-oxo-20-carboxylate (**29**)........ 34

**Figure S35:** 2D HSQC NMR spectrum of Methyl (20*S*)-3*β*-hydroxy-5*α*-pregn-6-oxo-20-carboxylate (**29**). . . . . . . . . . . . 35

**Figure S36:** 2D HMBC NMR spectrum of Methyl (20*S*)-3*β*-hydroxy-5*α*-pregn-6-oxo-20-carboxylate (**29**). . . . . . . . . . . . 36

**Figure S37:** IR spectrum of Methyl (20*S*)-3*β*-hydroxy-5*α*-pregn-6-dioxolan-20-carboxylate (**30**). . . . . . . . . . . . . 37

**Figure S38:** $^{1}$H NMR spectrum of Methyl (20*S*)-3*β*-hydroxy-5*α*-pregn-6-dioxolan-20-carboxylate (**30**). . . . . . . . . . . . . . 38

**Figure S39:** $^{13}$C NMR spectrum of Methyl (20*S*)-3*β*-hydroxy-5*α*-pregn-6-dioxolan-20-carboxylate (**30**). . . . . . . . . . . . . . 39

**Figure S40:** $^{13}$C DEPT-135 NMR spectrum of Methyl (20*S*)-3*β*-hydroxy-5*α*-pregn-6-dioxolan-20-carboxylate (**30**). . . . . . . . . . . . . . . . . . . . . . . . . . . . . . . . . . . . . . . . . . . . . . . . . . . . . . . . . . . . . . . . . . . . . . . . . . . . . 40

**Figure S41:** 2D HSQC NMR spectrum of Methyl (20*S*)-3*β*-hydroxy-5*α*-pregn-6-dioxolan-20-carboxylate (**30**). . . . . . . . . 41

**Figure S42:** 2D HMBC NMR spectrum of Methyl (20*S*)-3*β*-hydroxy-5*α*-pregn-6-dioxolan-20-carboxylate (**30**). . . . . . . . 42

**Figure S43:** IR spectrum of 3β-22-dihydroxy-5*α*-cholan-23,24-dinor-6-oxa (**31**). . . . . . . . . . . . . . . . . . . . . . . . . . . . . 43

**Figure S44:** $^{1}$H NMR spectrum of 3β-22-dihydroxy-5*α*-cholan-23,24-dinor-6-oxa (**31**). . . . . . . . . . . . . . . . . . . . . . . . . . . . . 44

**Figure S45:** $^{13}$C NMR spectrum of 3β-22-dihydroxy-5*α*-cholan-23,24-dinor-6-oxa (**31**). . . . . . . . . . . . . . . . . . . . . . . . . . . . 45

**Figure S46:** $^{13}$C DEPT-135 NMR spectrum of 3β-22-dihydroxy-5*α*-cholan-23,24-dinor-6-oxa (**31**). . . . . . . . . . . . . . . . . . . . 46

**Figure S47:** 2D HSQC NMR spectrum of 3β-22-dihydroxy-5*α*-cholan-23,24-dinor-6-oxa (**31**). . . . . . . . . . . . . . . . . . . . . . . 47

**Figure S48:** 2D HMBC NMR spectrum of 3β-22-dihydroxy-5*α*-cholan-23,24-dinor-6-oxa (**31**). . . . . . . . . . . . . . . . . . . . . . . 48

**Figure S49:** HRSM spectrum of 3β-hydroxy-5*α*-cholan-6-oxo-23,24-dinor-22-benzoate-22-yl (**15**). . . . . . . . . . . . . . . . . . . 49

**Figure S50:** IR spectrum of 3β-hydroxy-5*α*-cholan-6-oxo-23,24-dinor-22-benzoate-22-yl (**15**). . . . . . . . . . . . . . . . . . . . . . . 50

**Figure S51:** $^{1}$H NMR spectrum of 3β-hydroxy-5*α*-cholan-6-oxo-23,24-dinor-22-benzoate-22-yl (**15**). . . . . . . . . . . . . . . . . . 51

**Figure S52:** $^{13}$C NMR spectrum of 3β-hydroxy-5*α*-cholan-6-oxo-23,24-dinor-22-benzoate-22-yl (**15**). . . . . . . . . . . . . . . . . 52

**Figure S53:** $^{13}$C DEPT-135 NMR spectrum of 3β-hydroxy-5*α*-cholan-6-oxo-23,24-dinor-22-benzoate-22-yl (**15**). . . . . . . . 53

**Figure S54:** 2D HSQC NMR spectrum of 3β-hydroxy-5*α*-cholan-6-oxo-23,24-dinor-22-benzoate-22-yl (**15**). . . . . . . . . . . 54

**Figure S55:** 2D HMBC NMR spectrum of 3β-hydroxy-5*α*-cholan-6-oxo-23,24-dinor-22-benzoate-22-yl (**15**). . . . . . . . . . . 55

**Figure S56:** HRSM spectrum of 3β-hydroxy-5*α*-cholan-6-oxo-23,24-dinor-22-(4-methyl)benzoate-22-yl (**16**). . . . . . . . . . 56

**Figure S57:** IR spectrum of 3β-hydroxy-5*α*-cholan-6-oxo-23,24-dinor-22-(4-methyl)benzoate-22-yl (**16**). . . . . . . . . . . . . . 57

**Figure S58:** $^{1}$H NMR spectrum of 3β-hydroxy-5*α*-cholan-6-oxo-23,24-dinor-22-(4-methyl)benzoate-22-yl (**16**). . . . . . . . 58

**Figure S59:** $^{13}$C NMR spectrum of 3β-hydroxy-5*α*-cholan-6-oxo-23,24-dinor-22-(4-methyl)benzoate-22-yl (**16**). . . . . . . . 59

**Figure S60:** $^{13}$C DEPT-135 NMR spectrum of 3β-hydroxy-5*α*-cholan-6-oxo-23,24-dinor-22-(4-methyl)benzoate-22-yl (**16**). . . . . . . . . . . . . . . . . . . . . . . . . . . . . . . . . . . . . . . . . . . . . . . . . . . . . . . . . . . . . . . . . . . . . . . . . . . . . . . . 60

**Figure S61:** 2D HSQC NMR spectrum of 3β-hydroxy-5*α*-cholan-6-oxo-23,24-dinor-22-(4-methyl)benzoate-22-yl (**16**). . 61

**Figure S62:** 2D HMBC NMR spectrum of 3β-hydroxy-5*α*-cholan-6-oxo-23,24-dinor-22-(4-methyl)benzoate-22-yl (**16**). . 62

**Figure S63:** HRSM spectrum of 3β-hydroxy-5*α*-cholan-6-oxo-23,24-dinor-22-(4-methoxy)benzoate-22-yl (**17**). . . . . . . . . 63

**Figure S64:** IR spectrum of 3β-hydroxy-5*α*-cholan-6-oxo-23,24-dinor-22-(4-methoxy)benzoate-22-yl (**17**). . . . 64

**Figure S65:** $^{1}$H NMR spectrum of 3β-hydroxy-5*α*-cholan-6-oxo-23,24-dinor-22-(4-methoxy)benzoate-22-yl (**17**). . . . . . . 65

**Figure S66:** $^{13}$C NMR spectrum of 3β-hydroxy-5*α*-cholan-6-oxo-23,24-dinor-22-(4-methoxy)benzoate-22-yl (**17**). . . . . . . 66

**Figure S67:** $^{13}$C DEPT-135 NMR spectrum of 3β-hydroxy-5*α*-cholan-6-oxo-23,24-dinor-22-(4-methoxy)benzoate-22-yl (**17**). . . . . . . . . . . . . . . . . . . . . . . . . . . . . . . . . . . . . . . . . . . . . . . . . . . . . . . . . . . . . . . . . . . . . . . . . . . . . . . . 67

**Figure S68:** 2D HSQC NMR spectrum of 3β-hydroxy-5*α*-cholan-6-oxo-23,24-dinor-22-(4-methoxy)benzoate-22-yl (**17**). . . . . . . . . . . . . . . . . . . . . . . . . . . . . . . . . . . . . . . . . . . . . . . . . . . . . . . . . . . . . . . . . . . . . . . . . . . . . . . . 68

**Figure S69:** 2D HMBC NMR spectrum of 3β-hydroxy-5α-cholan-6-oxo-23,24-dinor-22-(4-methoxy)benzoate-22-yl (**17**). . . . . . . . . . 69
**Figure S70:** HRSM spectrum of 3β-hydroxy-5α-cholan-6-oxo-23,24-dinor-22-(4-chloro)benzoate-22-yl (**18**). . . . . . . . . . . 70
**Figure S71:** IR spectrum of 3β-hydroxy-5α-cholan-6-oxo-23,24-dinor-22-(4-chloro)benzoate-22-yl (**18**). . . . . . . 71
**Figure S72:** $^{1}$H NMR spectrum of 3β-hydroxy-5α-cholan-6-oxo-23,24-dinor-22-(4-chloro)benzoate-22-yl (**18**). . . . . . . . . 72
**Figure S73:** $^{13}$C NMR spectrum of 3β-hydroxy-5α-cholan-6-oxo-23,24-dinor-22-(4-chloro)benzoate-22-yl (**18**). . . . . . . . . 73
**Figure S74:** $^{13}$C DEPT-135 NMR spectrum of 3β-hydroxy-5α-cholan-6-oxo-23,24-dinor-22-(4-chloro)benzoate-22-yl (**18**). . . . . . . . . . 74
**Figure S75:** 2D HSQC NMR spectrum of 3β-hydroxy-5α-cholan-6-oxo-23,24-dinor-22-(4-chloro)benzoate-22-yl (**18**). . . 75
**Figure S76:** 2D HMBC NMR spectrum of 3β-hydroxy-5α-cholan-6-oxo-23,24-dinor-22-(4-chloro)benzoate-22-yl (**18**). . 76
**Figure S77:** HRSM spectrum of 3β-hydroxy-5α-cholan-6-oxo-23,24-dinor-22-(4-bromo)benzoate-22-yl (**19**). . . . . . . . . . . 77
**Figure S78:** IR spectrum of 3β-hydroxy-5α-cholan-6-oxo-23,24-dinor-22-(4-bromo)benzoate-22-yl (**19**). . . . . . . . . . . . . . . 78
**Figure S79:** $^{1}$H NMR spectrum of 3β-hydroxy-5α-cholan-6-oxo-23,24-dinor-22-(4-bromo)benzoate-22-yl (**19**). . . . . . . . . 79
**Figure S80:** $^{13}$C NMR spectrum of 3β-hydroxy-5α-cholan-6-oxo-23,24-dinor-22-(4-bromo)benzoate-22-yl (**19**). . . . . . . . . 80
**Figure S81:** $^{13}$C DEPT-135 NMR spectrum of 3β-hydroxy-5α-cholan-6-oxo-23,24-dinor-22-(4-bromo)benzoate-22-yl (**19**). . . . . . . . . . 81
**Figure S82:** 2D HSQC NMR spectrum of 3β-hydroxy-5α-cholan-6-oxo-23,24-dinor-22-(4-bromo)benzoate-22-yl (**19**). . 82
**Figure S83:** 2D HMBC NMR spectrum of 3β-hydroxy-5α-cholan-6-oxo-23,24-dinor-22-(4-bromo)benzoate-22-yl (**19**). . 83
**Figure S84:** HRSM spectrum of 3β-hydroxy-5α-cholan-6-oxo-23,24-dinor-22-(4-fluoro)benzoate-22-yl (**20**). . . . . . . . . . . 84
**Figure S85:** IR spectrum of 3β-hydroxy-5α-cholan-6-oxo-23,24-dinor-22-(4-fluoro)benzoate-22-yl (**20**). . . . . . . 85
**Figure S86:** $^{1}$H NMR spectrum of 3β-hydroxy-5α-cholan-6-oxo-23,24-dinor-22-(4-fluoro)benzoate-22-yl (**20**). . . . . . . . . 86
**Figure S87:** $^{13}$C NMR spectrum of 3β-hydroxy-5α-cholan-6-oxo-23,24-dinor-22-(4-fluoro)benzoate-22-yl (**20**). . . . . . . . . 87
**Figure S88:** $^{13}$C DEPT-135 NMR spectrum of 3β-hydroxy-5α-cholan-6-oxo-23,24-dinor-22-(4-fluoro)benzoate-22-yl (**20**). . . . . . . . . . 88
**Figure S89:** 2D HSQC NMR spectrum of 3β-hydroxy-5α-cholan-6-oxo-23,24-dinor-22-(4-fluoro)benzoate-22-yl (**20**). . . 89
**Figure S90:** 2D HMBC NMR spectrum of 3β-hydroxy-5α-cholan-6-oxo-23,24-dinor-22-(4-fluoro)benzoate-22-yl (**20**). . 90
**Figure S91:** HRSM spectrum of 3β-hydroxy-5α-cholan-6-oxo-23,24-dinor-22-(4-iodine)benzoate-22-yl (**21**). . . . . . . . . . . 91
**Figure S92:** IR spectrum of 3β-hydroxy-5α-cholan-6-oxo-23,24-dinor-22-(4-iodine)benzoate-22-yl (**21**). . . . . . . 92
**Figure S93:** $^{1}$H NMR spectrum of 3β-hydroxy-5α-cholan-6-oxo-23,24-dinor-22-(4-iodine)benzoate-22-yl (**21**). . . . . . . . . 93
**Figure S94:** $^{13}$C NMR spectrum of 3β-hydroxy-5α-cholan-6-oxo-23,24-dinor-22-(4-iodine)benzoate-22-yl (**21**). . . . . . . . . 94
**Figure S95:** $^{13}$C DEPT-135 NMR spectrum of 3β-hydroxy-5α-cholan-6-oxo-23,24-dinor-22-(4-iodine)benzoate-22-yl (**21**). . . . . . . . . . 95
**Figure S96:** 2D HSQC NMR spectrum of 3β-hydroxy-5α-cholan-6-oxo-23,24-dinor-22-(4-iodine)benzoate-22-yl (**21**). . . 96
**Figure S97:** 2D HMBC NMR spectrum of 3β-hydroxy-5α-cholan-6-oxo-23,24-dinor-22-(4-iodine)benzoate-22-yl (**21**). . 97
**Figure S98:** HRSM spectrum of 3β-hydroxy-5α-cholan-6-oxo-23,24-dinor-22-(4-cyan)benzoate-22-yl (**22**). . . . . . . . . . . . 98
**Figure S99:** IR spectrum of 3β-hydroxy-5α-cholan-6-oxo-23,24-dinor-22-(4-cyan)benzoate-22-yl (**22**). . . . . . . . . . . . . . . . 99
**Figure S100:** $^{1}$H NMR spectrum of 3β-hydroxy-5α-cholan-6-oxo-23,24-dinor-22-(4-cyan)benzoate-22-yl (**22**). . . . . . . . . . . 100
**Figure S101:** $^{13}$C NMR spectrum of 3β-hydroxy-5α-cholan-6-oxo-23,24-dinor-22-(4-cyan)benzoate-22-yl (**22**). . . . . . . . . . 101
**Figure S102:** $^{13}$C DEPT-135 NMR spectrum of 3β-hydroxy-5α-cholan-6-oxo-23,24-dinor-22-(4-cyan)benzoate-22-yl (**22**). 102

**Figure S103:** 2D HSQC NMR spectrum of 3β-hydroxy-5α-cholan-6-oxo-23,24-dinor-22-(4-cyan)benzoate-22-yl (**22**). . . . 103

**Figure S104:** 2D HMBC NMR spectrum of 3β-hydroxy-5α-cholan-6-oxo-23,24-dinor-22-(4-cyan)benzoate-22-yl (**22**). . . . 104

**Table S1:** Rice-lamina assays using the second leaf lamina joints (Angle Opening, Degrees) of excised leaf segments treated with BRs analogs (**1**, **31** and **15**-**22**) at different concentrations. Brassinolide was used as positive control at the same concentrations. . . . . . . . . . . . . . . . . . . . . . . . . . . . . . . . . . . . . . . . . . . . . . . . . . . . . . . . . . . 105

**Table S2:** Analysis of docked brassinolide (1) and synthetic analogs (**1**, **31** and **15**-**22**). ΔEb: Binding Energy in kcal/mol. Hydrogen bonds are represented in yellow segmented lines. π-π stacking are represented in green (turquoise) segmented lines. Visualization of the docked poses was performed using Discovery Studio Visualizer (BIOVIA, San Diego, CA, USA). . . . . . . . . . . . . . . . . . . . . . . . . . . . . . . . . . . . . . . . . . . . . 108

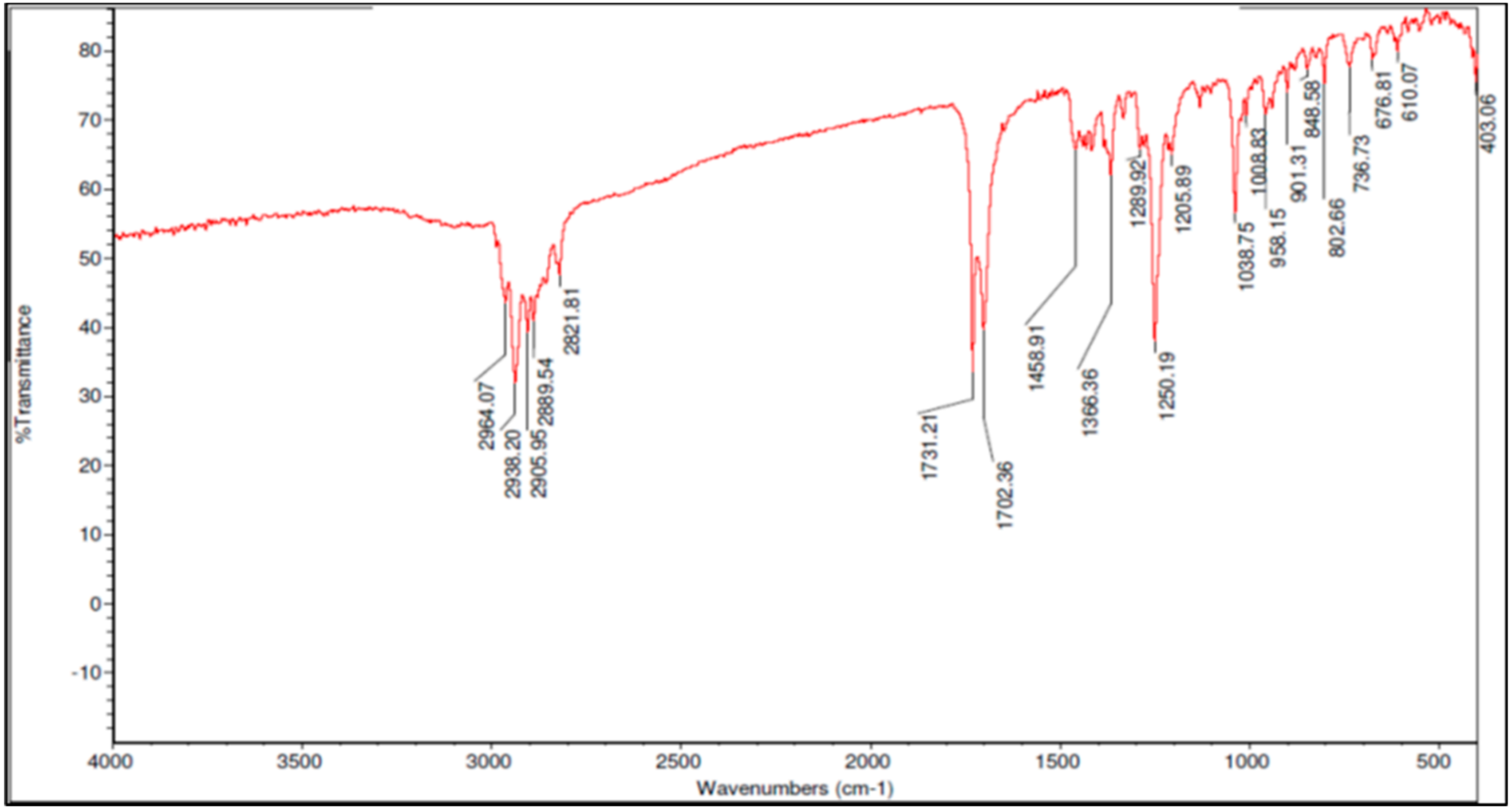


**Figure S1** IR spectrum of Methyl (20*S*)-3*β*-Acetoxypregn-5-ene-20-carboxylate (**24**).

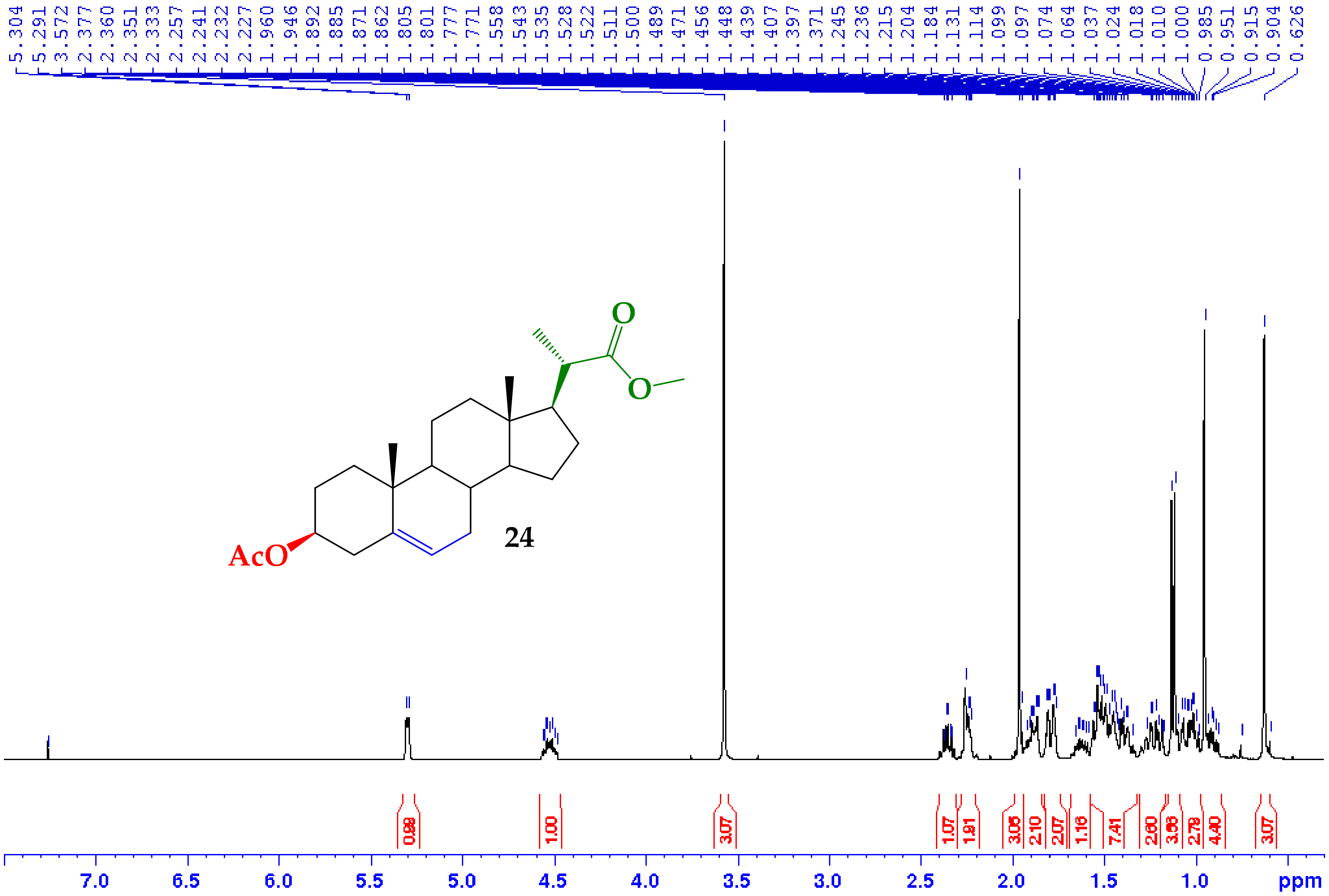

**Figure S2**. $^{1}$H NMR spectrum of Methyl (20*S*)-3*β*-Acetoxypregn-5-ene-20-carboxylate (**24**).

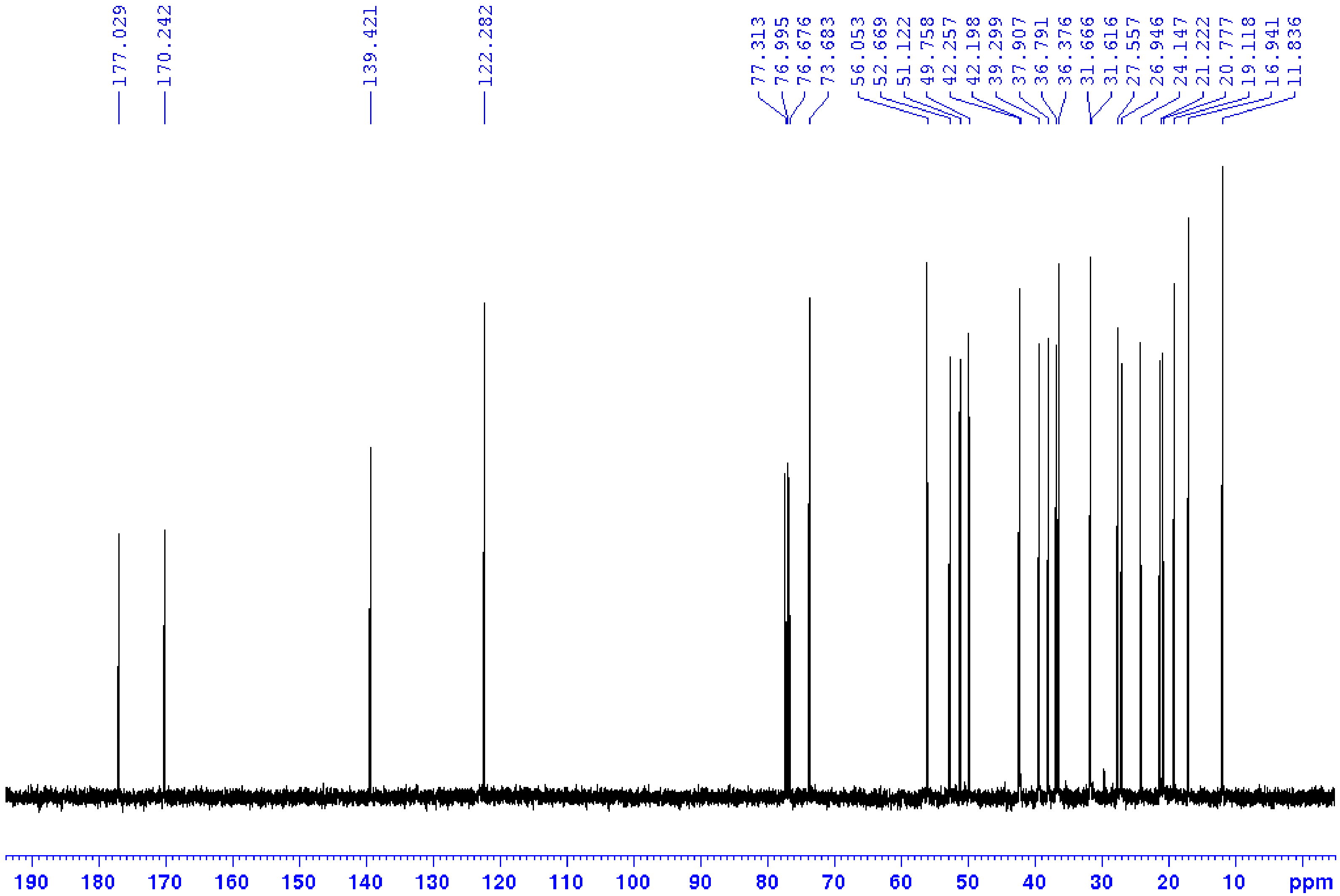


**Figure S3**. $^{13}$C NMR spectrum of Methyl (20*S*)-3*β*-Acetoxypregn-5-ene-20-carboxylate (**24**).

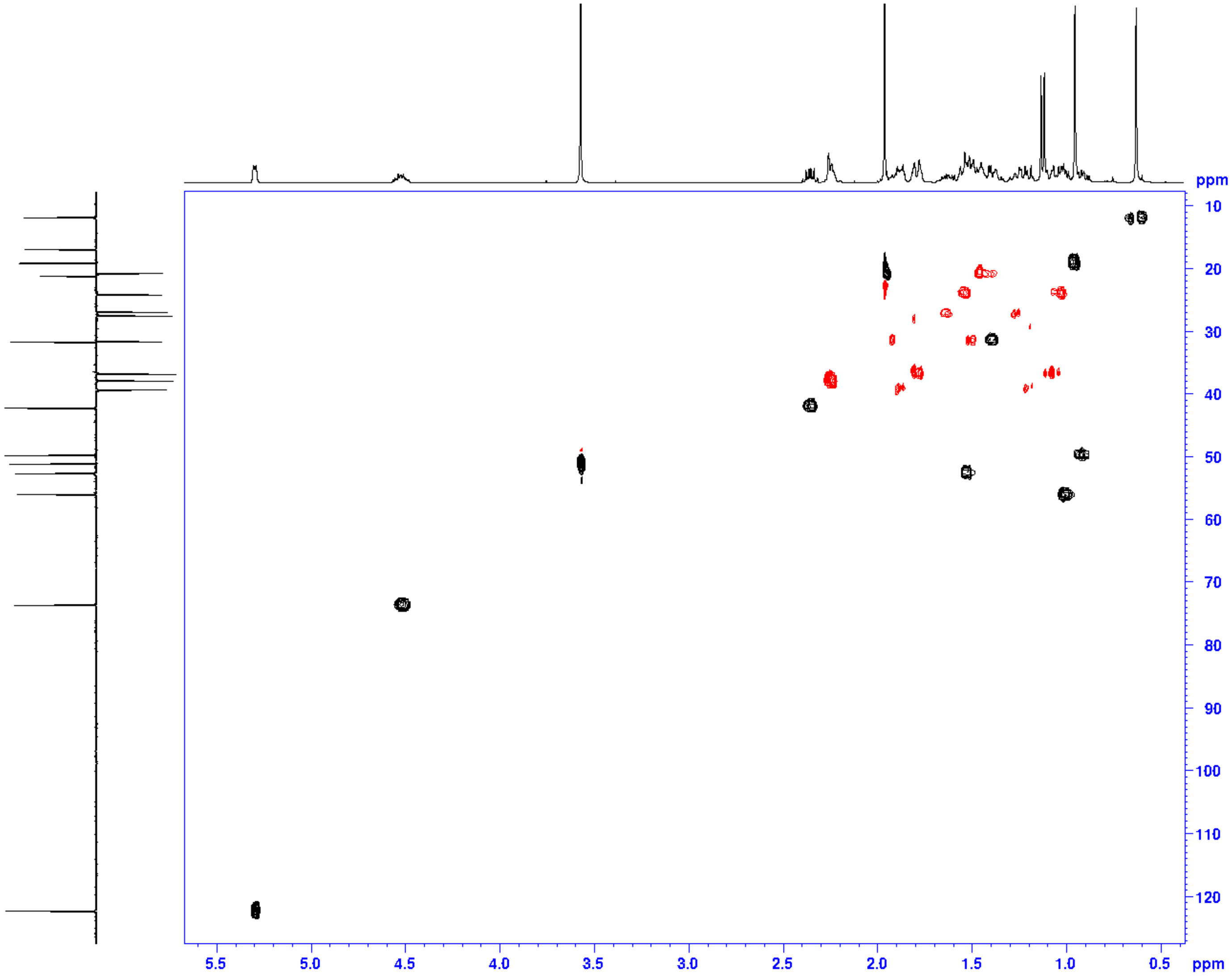


**Figure S4**. 2D HSQC NMR spectrum of of Methyl (20*S*)-3*β*-Acetoxypregn-5-ene-20-carboxylate (**24**).

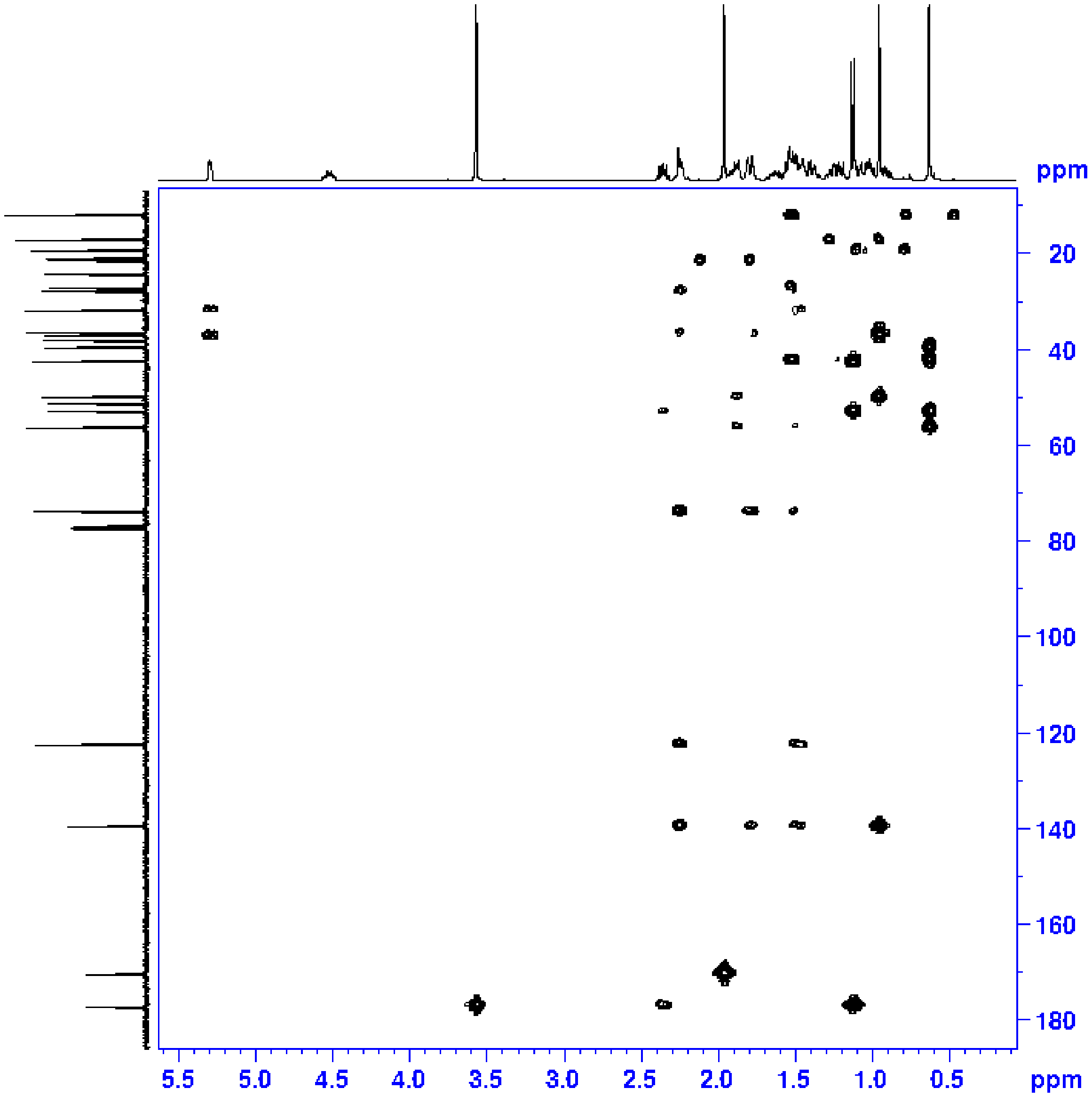


**Figure S5**. 2D HMBC NMR spectrum of of Methyl (20*S*)-3*β*-Acetoxypregn-5-ene-20-carboxylate (**24**).

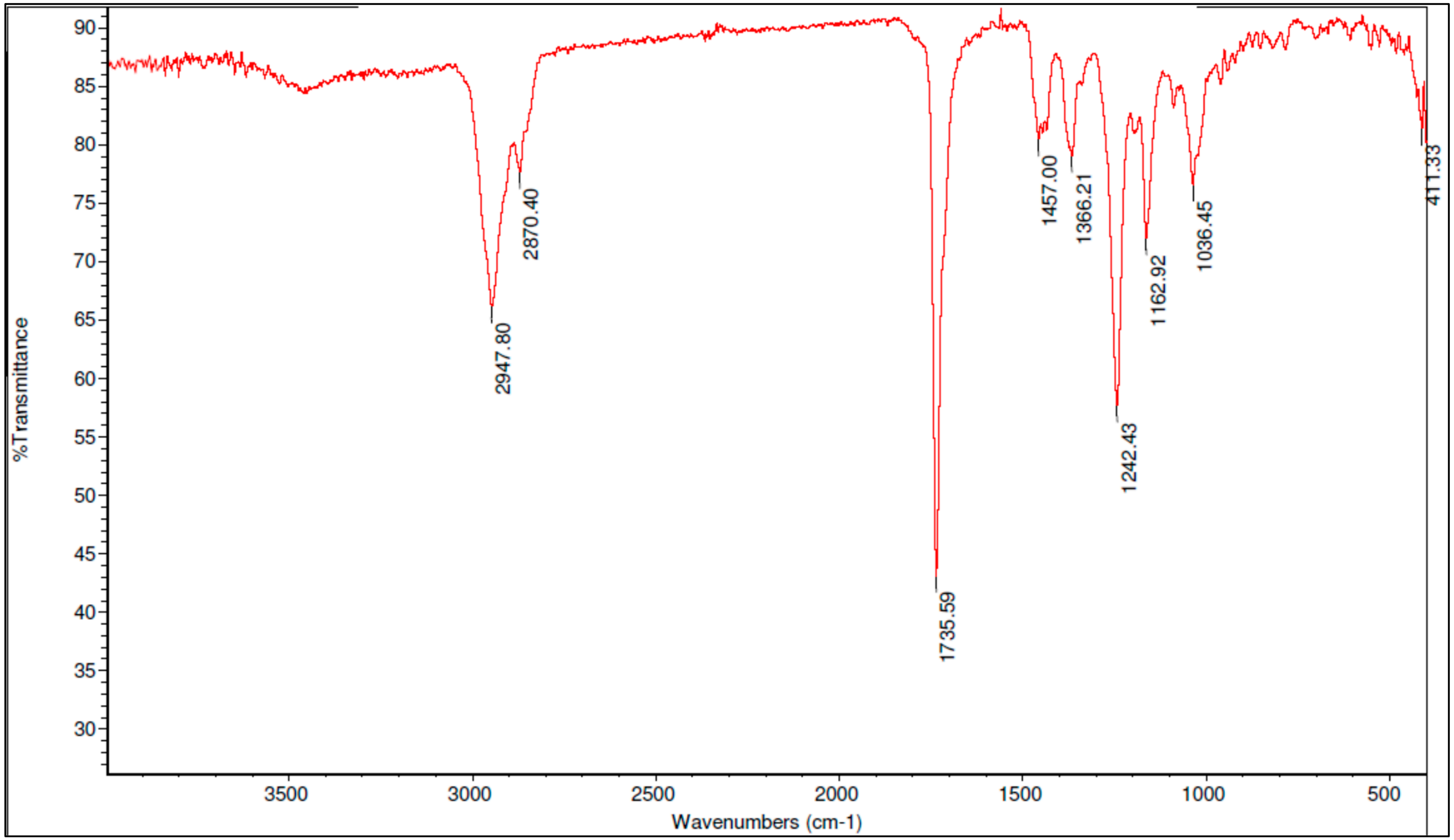


**Figure S6**. IR spectrum of Methyl (20*S*)-3*β*-Acetoxypregn-5β,6β-epoxy-20-carboxylate (**25**).

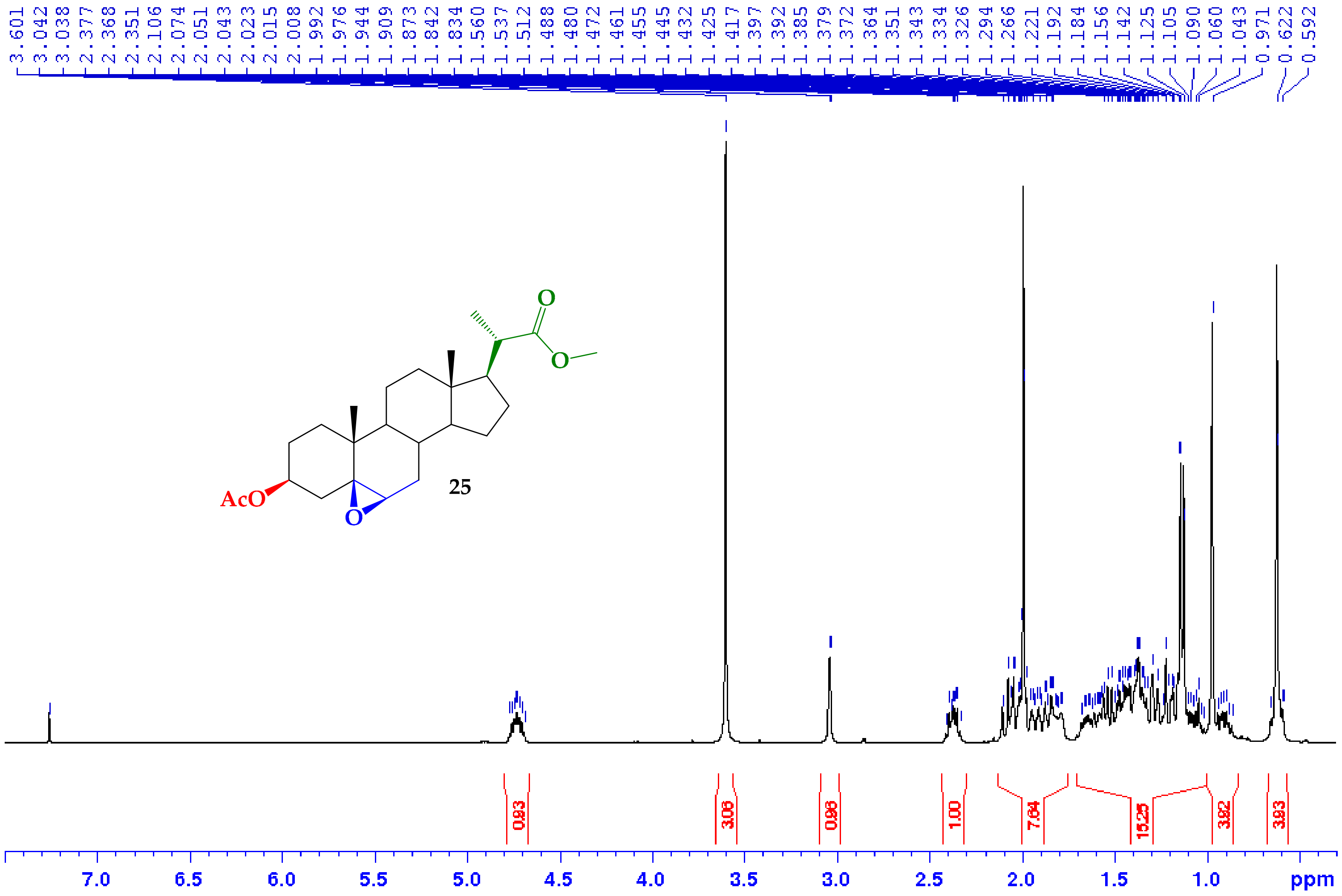

**Figure S7**. $^{1}$H NMR spectrum of Methyl (20*S*)-3*β*-Acetoxypregn-5β,6β-epoxy-20-carboxylate (**25**).

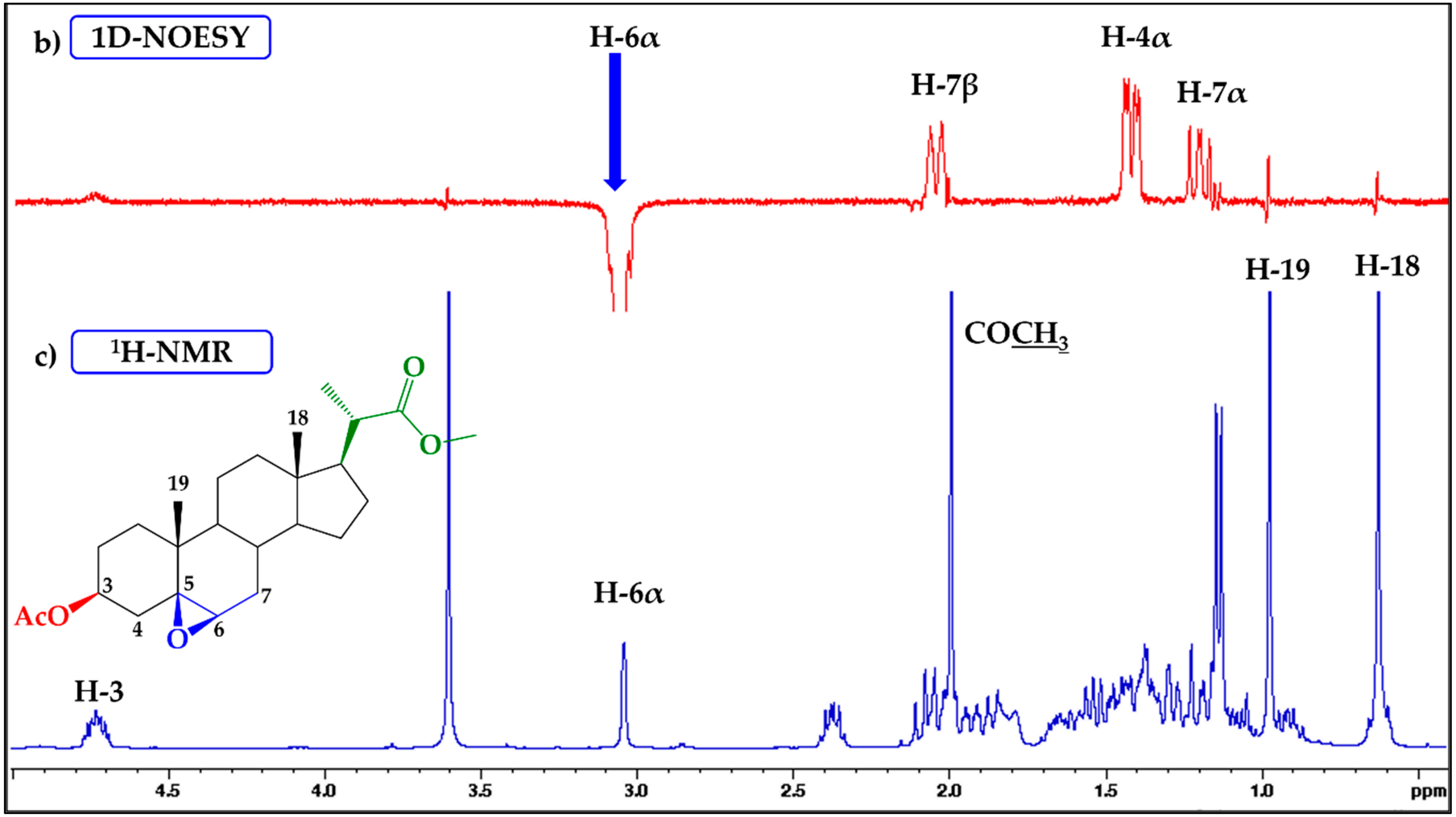


**Figure S8**. 1D selective NOESY NMR spectrum of Methyl (20S)-3β-Acetoxypregn-5β,6β-epoxy-20-carboxylate (**25**).

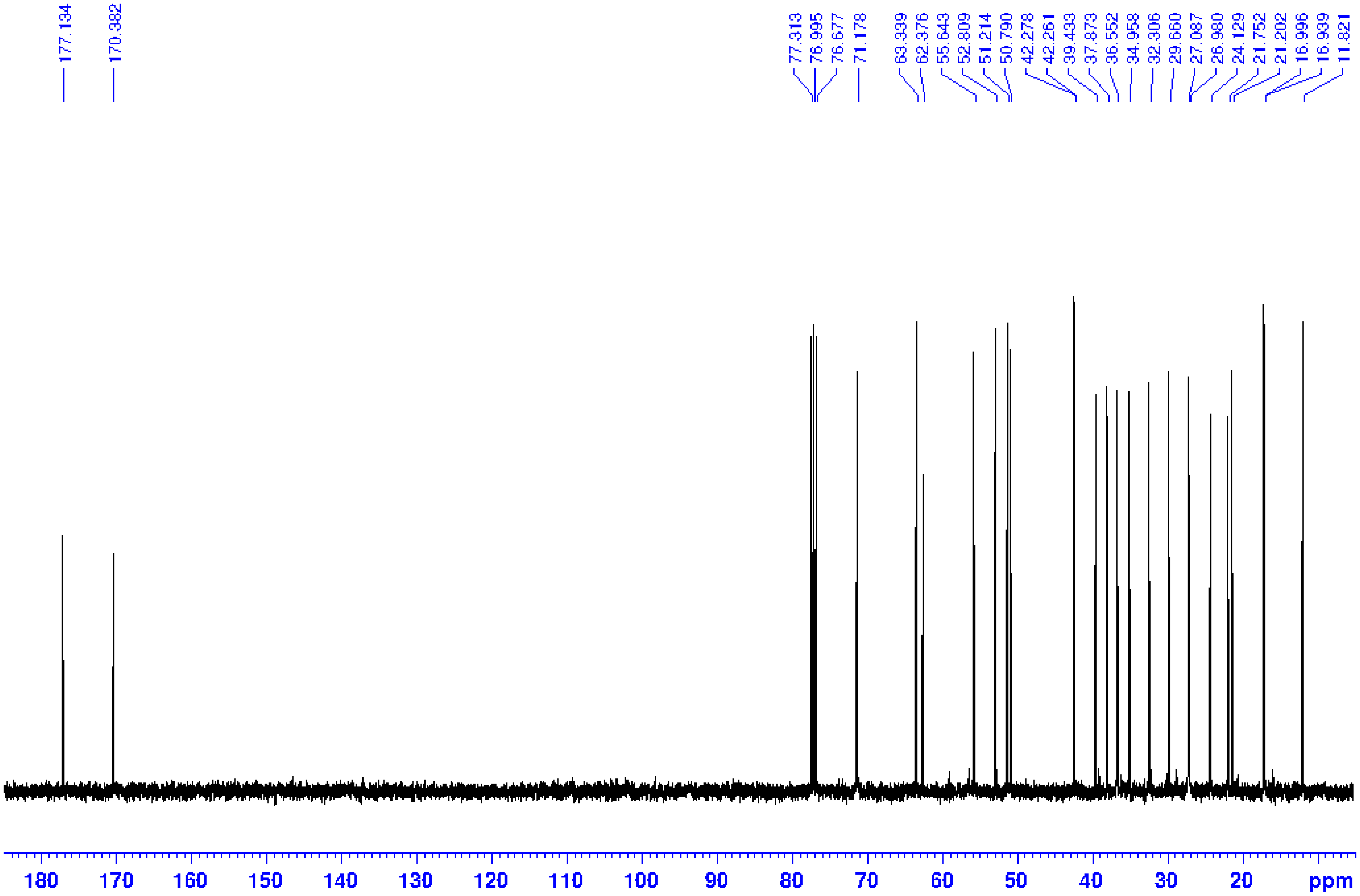


**Figure S9**. $^{13}C$ NMR spectrum of Methyl (20*S*)-3*β*-Acetoxypregn-5β,6β-epoxy-20-carboxylate (**25**).

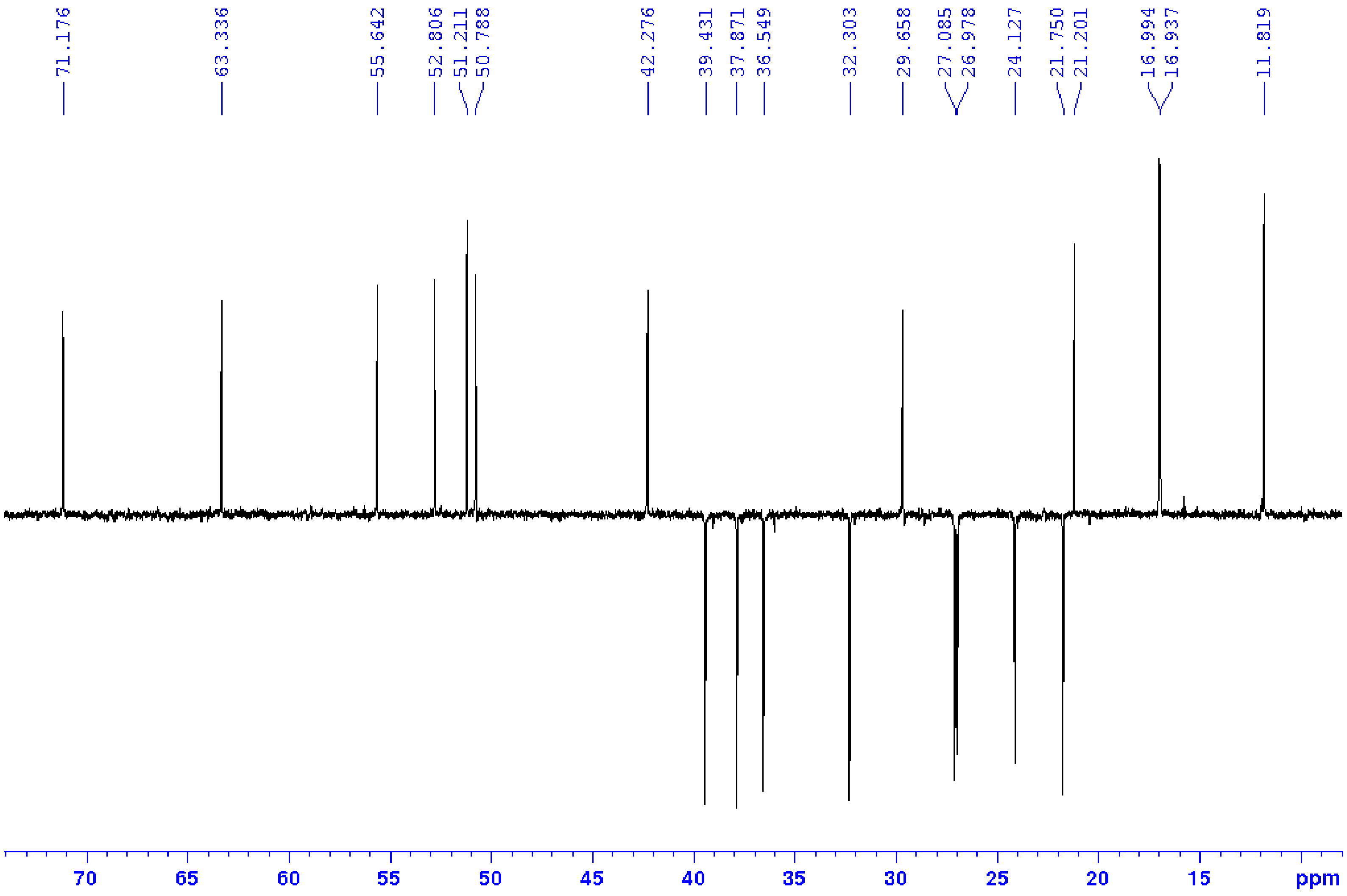


**Figure S10**. $^{13}C$ DEPT-135 NMR spectrum of Methyl (20*S*)-3*β*-Acetoxypregn-5β,6β-epoxy-20-carboxylate (**25**).

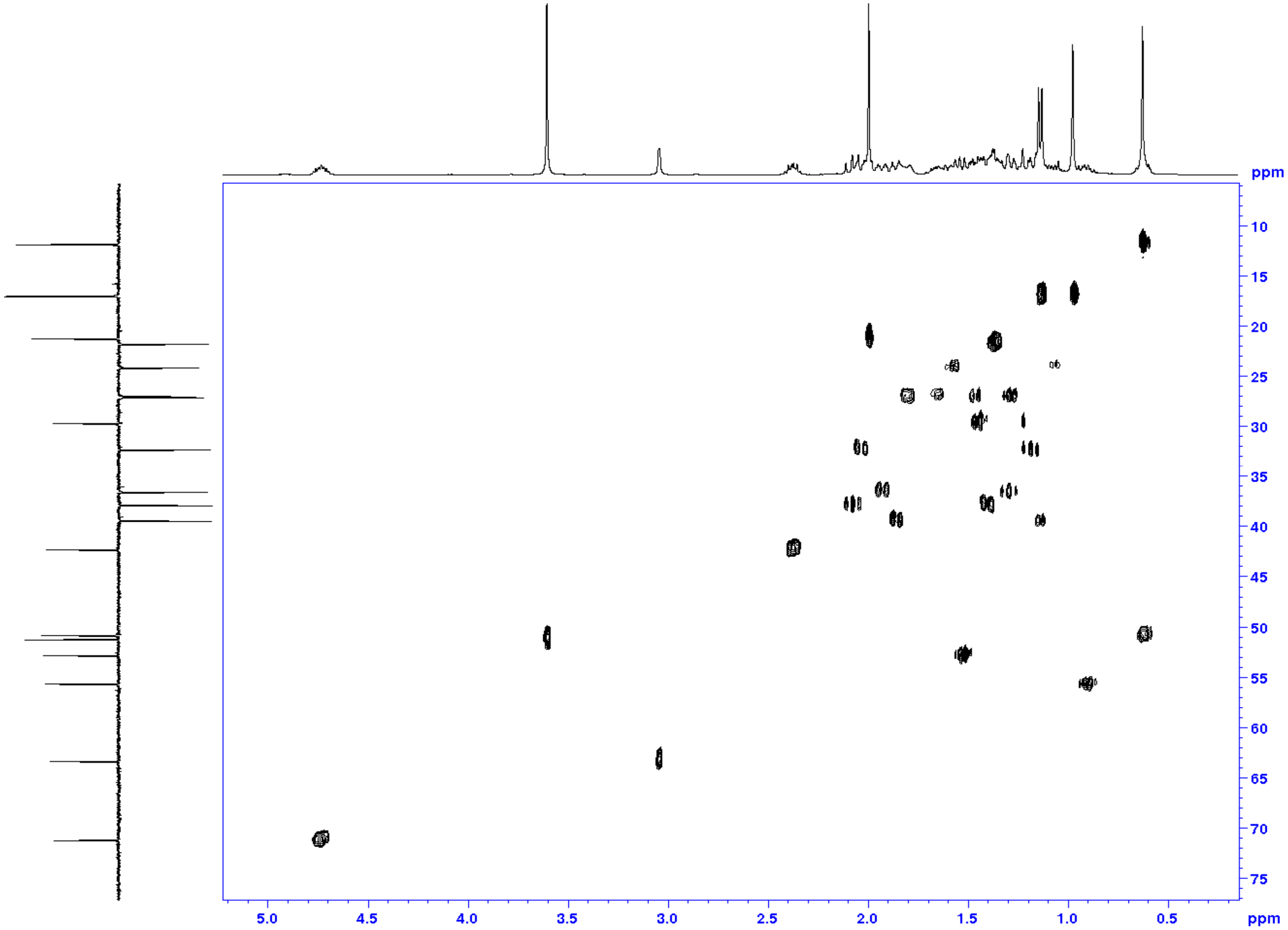


**Figure S11**. 2D HSQC NMR spectrum of Methyl (20*S*)-3*β*-Acetoxypregn-5β,6β-epoxy-20-carboxylate (**25**).

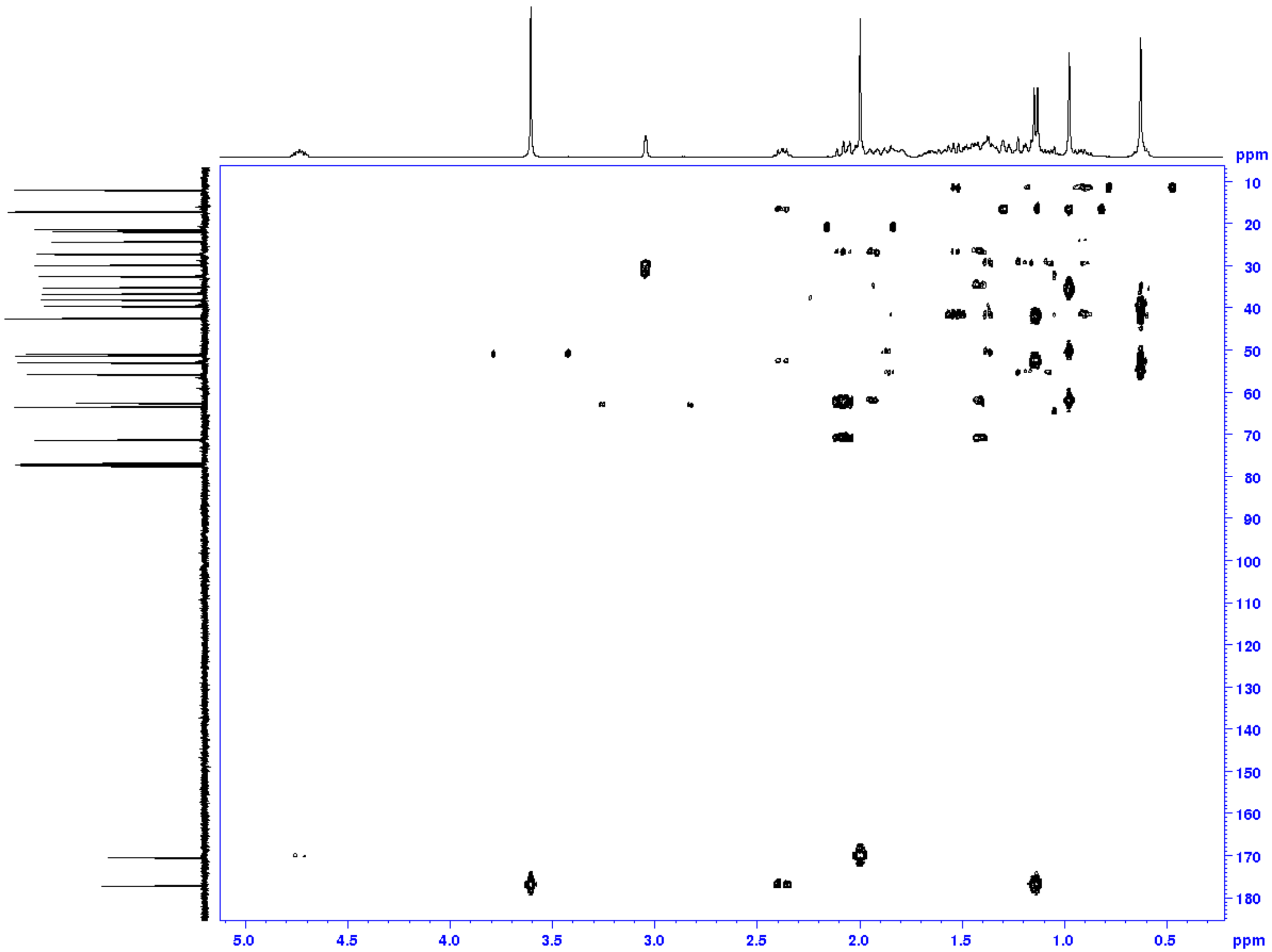


**Figure S12**. 2D HMBC NMR spectrum of Methyl (20*S*)-3*β*-Acetoxypregn-5β,6β-epoxy-20-carboxylate (**25**).

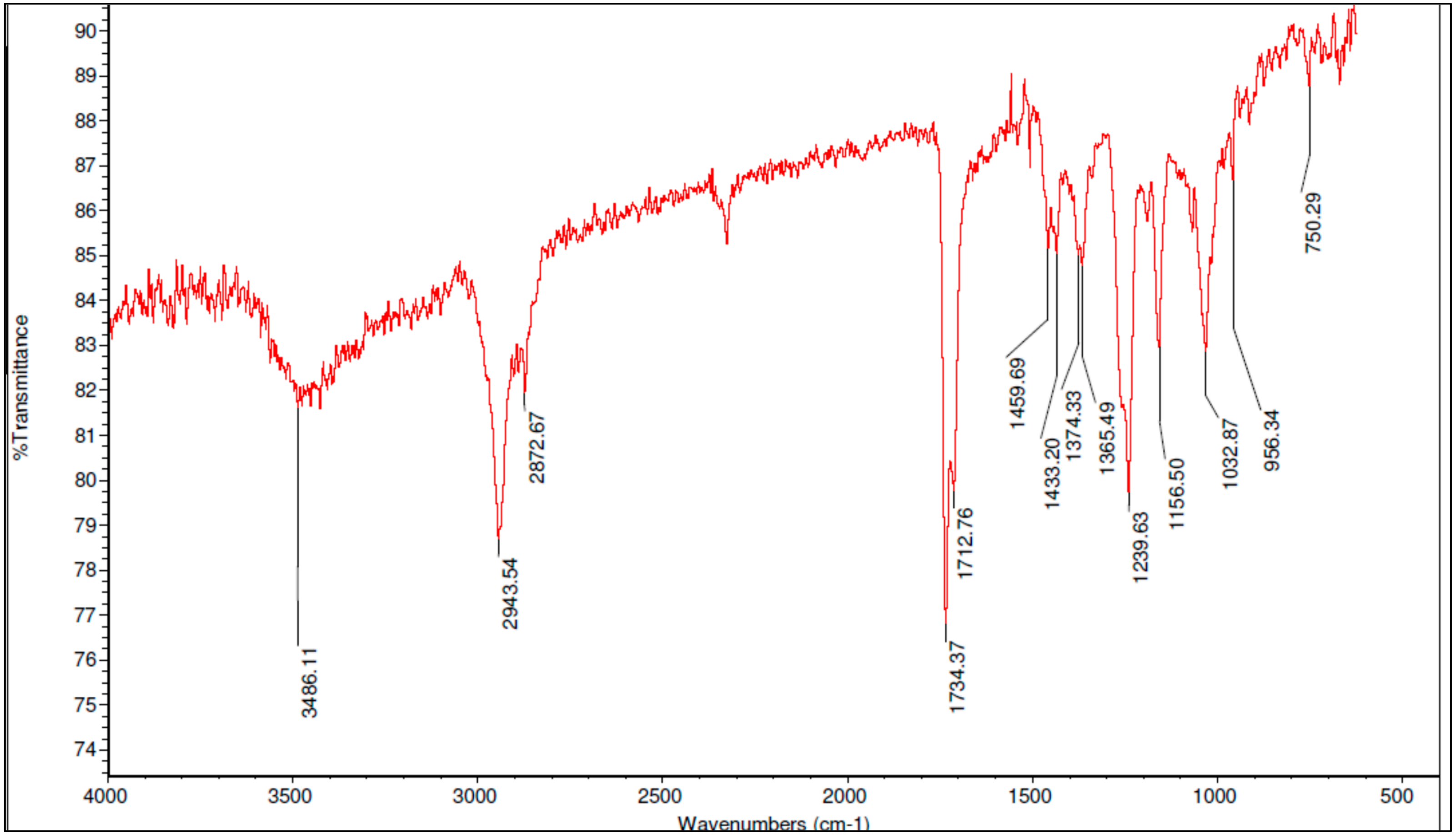


**Figure S13**. IR spectrum of Methyl (20*S*)-3*β*-Acetoxypregn-5*α*-bromo-6β-hydroxy-20-carboxylate (**26**).

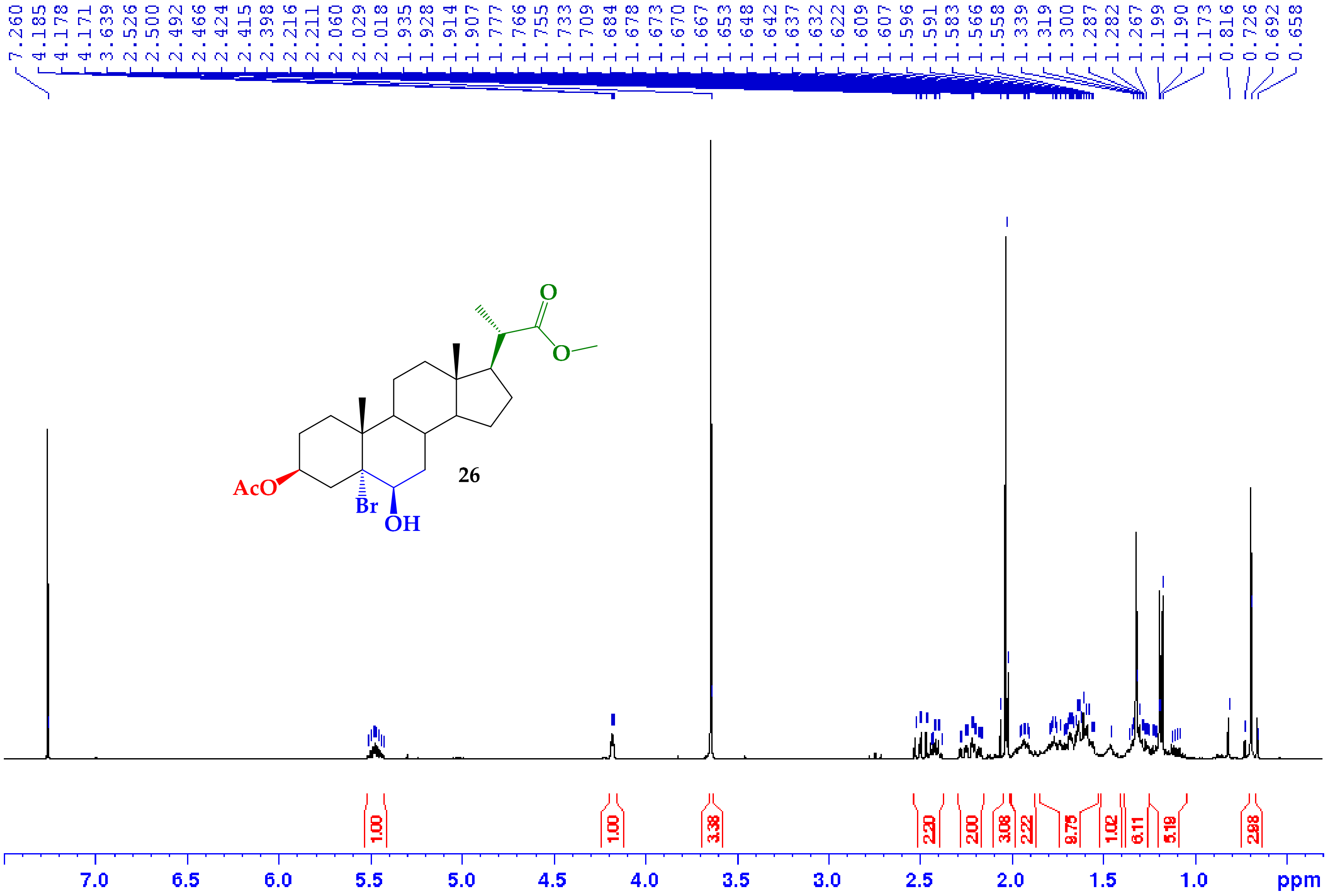

**Figure S14**. $^1$H NMR spectrum of Methyl (20*S*)-3*β*-Acetoxypregn-5α-bromo-6β-hydroxy-20-carboxylate (**26**).

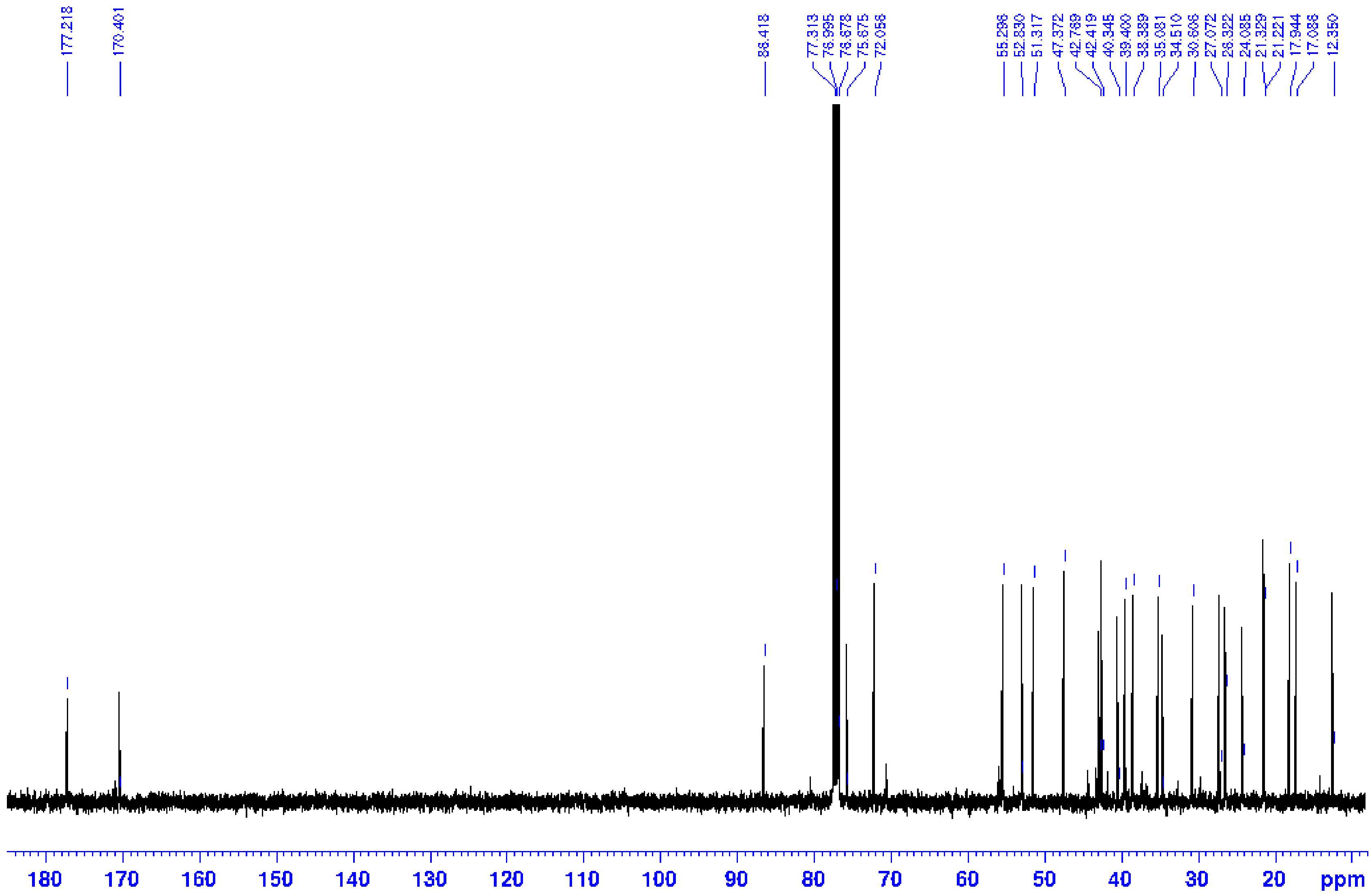


**Figure S15**. $^{13}$C NMR spectrum of Methyl (20*S*)-3*β*-Acetoxypregn-5α-bromo-6β-hydroxy-20-carboxylate (**26**).

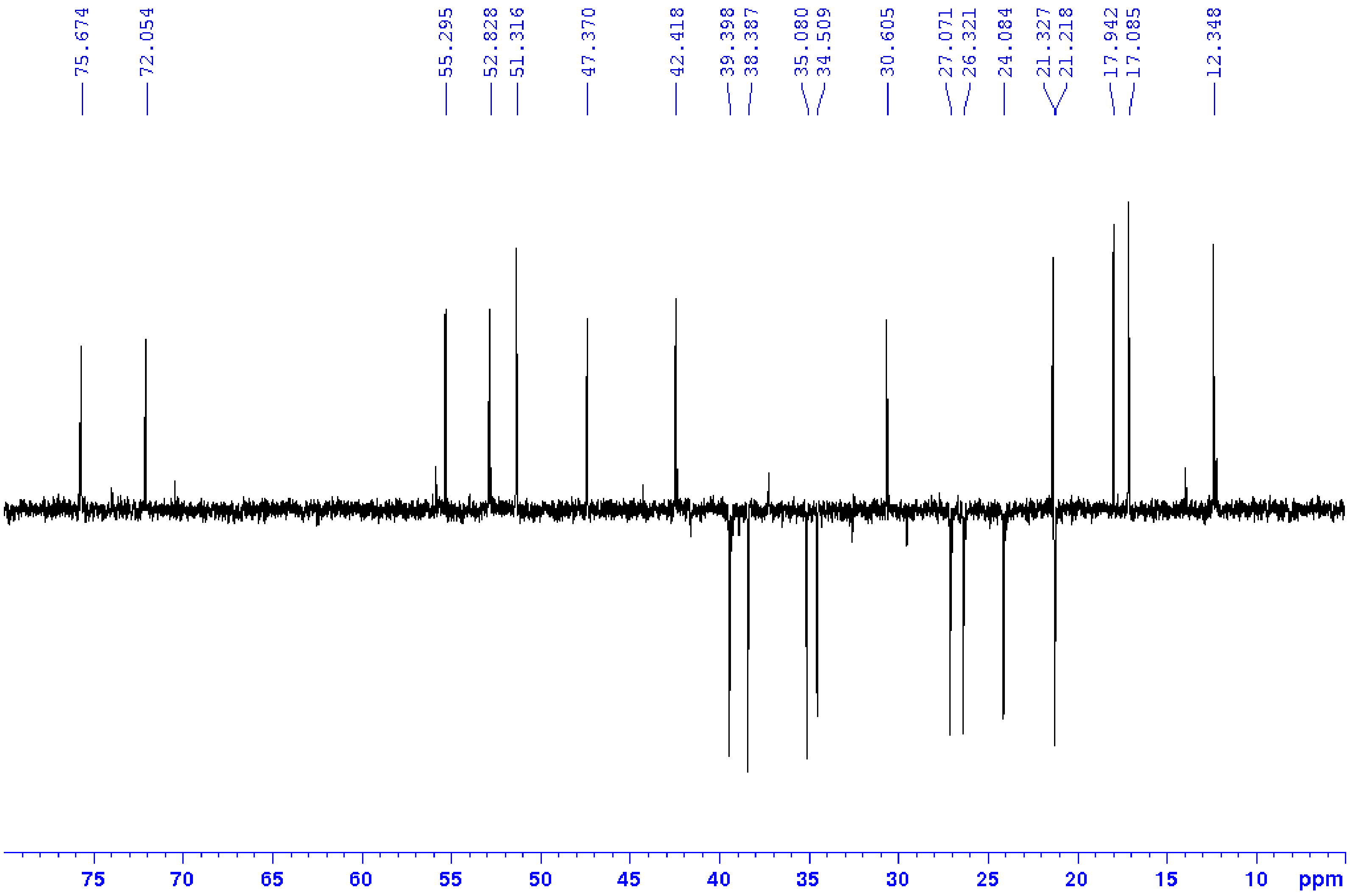


**Figure S16**. $^{13}$C DEPT-135 NMR spectrum of Methyl (20*S*)-3*β*-Acetoxypregn-5*α*-bromo-6β-hydroxy-20-carboxylate (**26**).

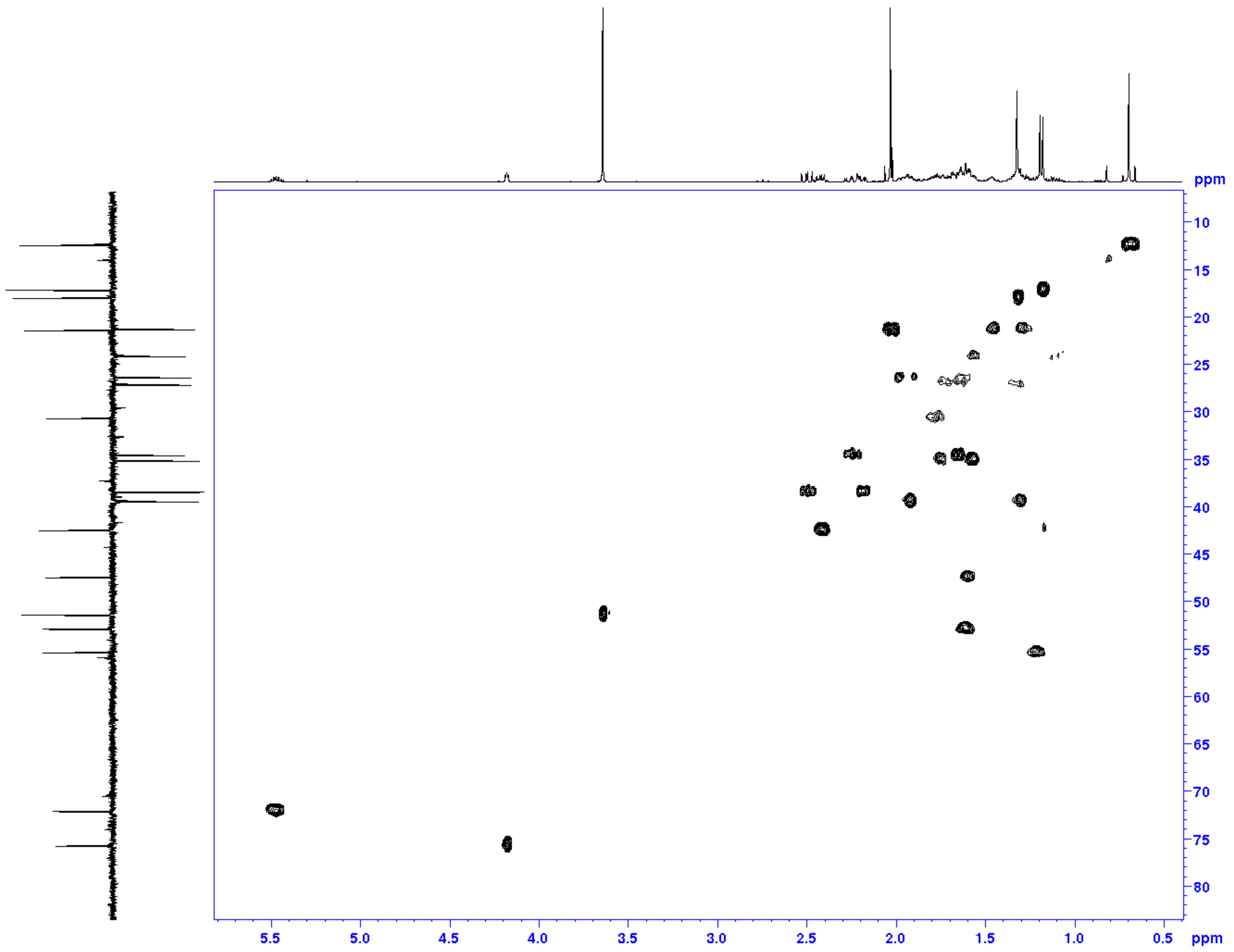


**Figure S17**. 2D HSQC NMR spectrum of Methyl (20*S*)-3*β*-Acetoxypregn-5*α*-bromo-6β-hydroxy-20-carboxylate (**26**).

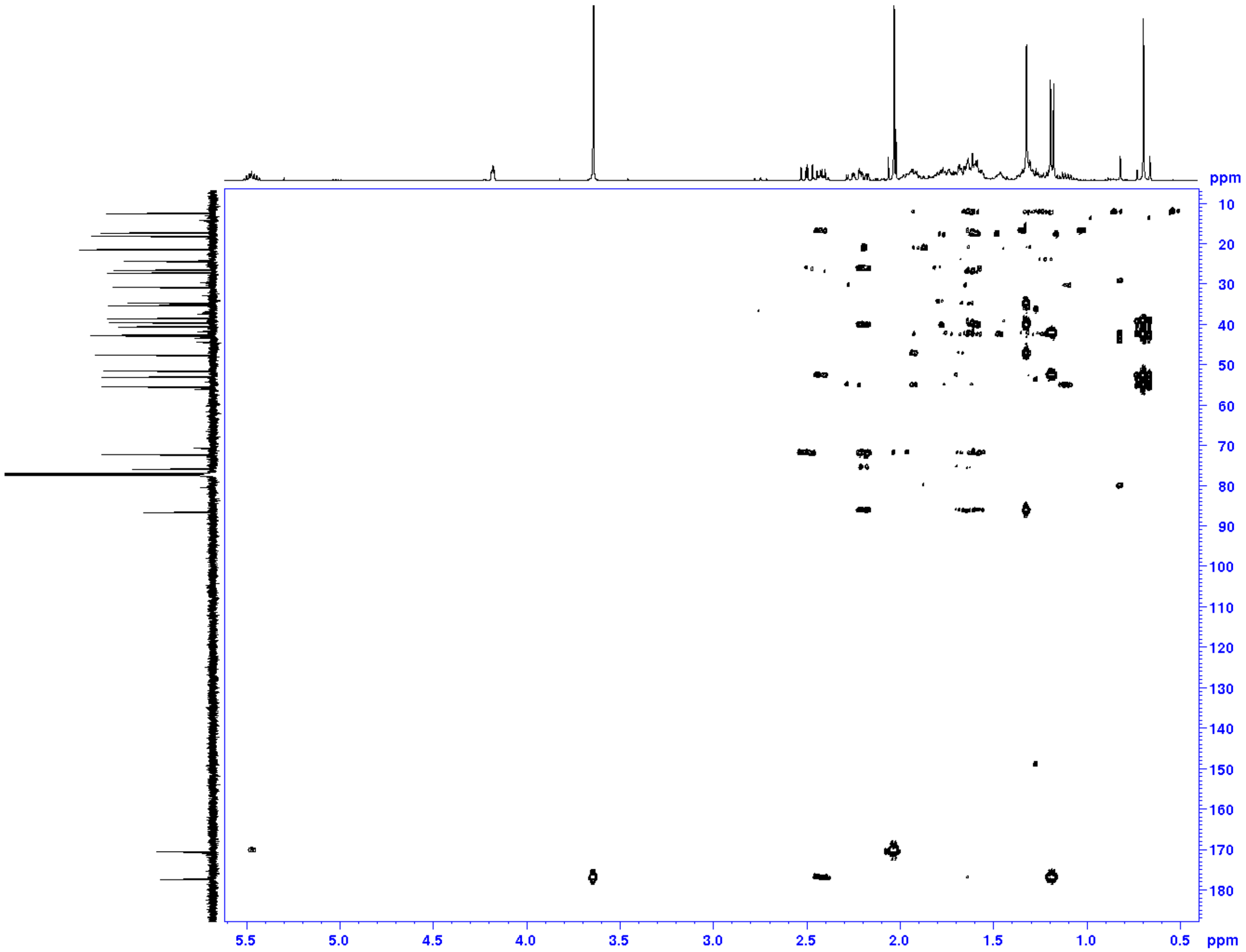


**Figure S18**. 2D HMBC NMR spectrum of Methyl (20*S*)-3*β*-Acetoxypregn-5*α*-bromo-6β-hydroxy-20-carboxylate (**26**).

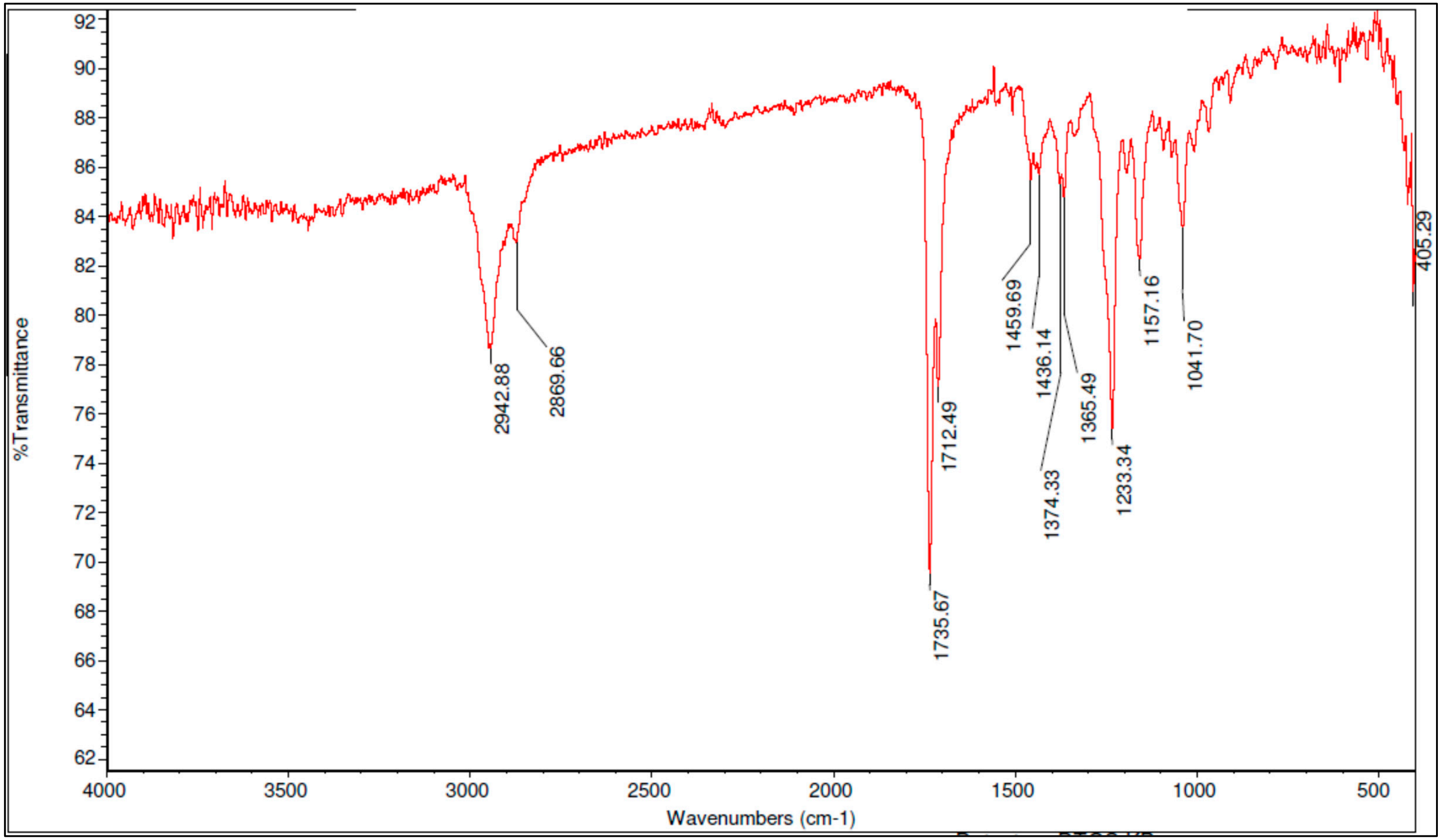

**Figure S19**. IR spectrum of Methyl (20*S*)-3*β*-Acetoxypregn-5*α*-bromo-6-oxo-20-carboxylate (**27**).

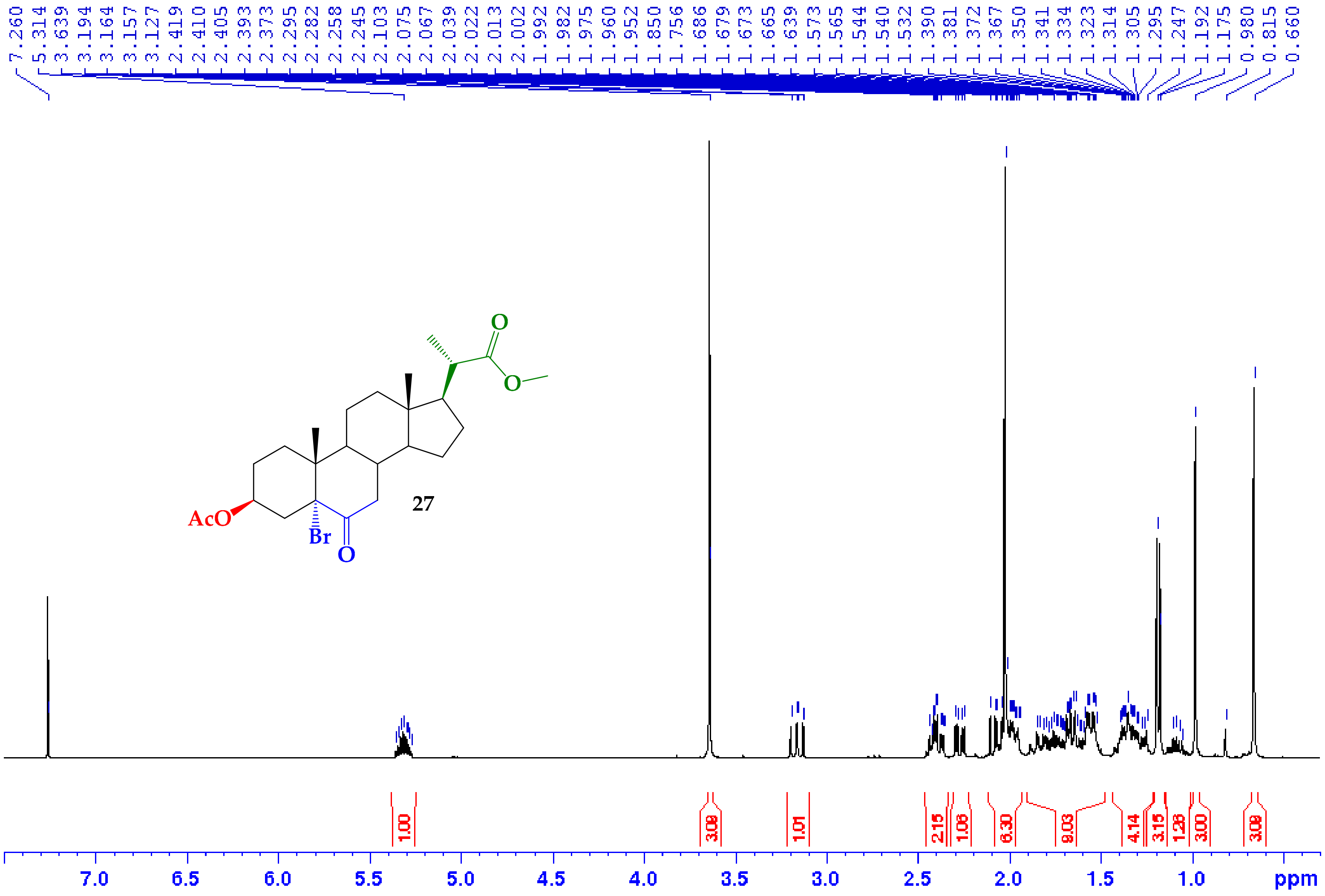

**Figure S20**. $^{1}$H NMR spectrum of Methyl (20*S*)-3*β*-Acetoxypregn-5*α*-bromo-6-oxo-20-carboxylate (**27**).

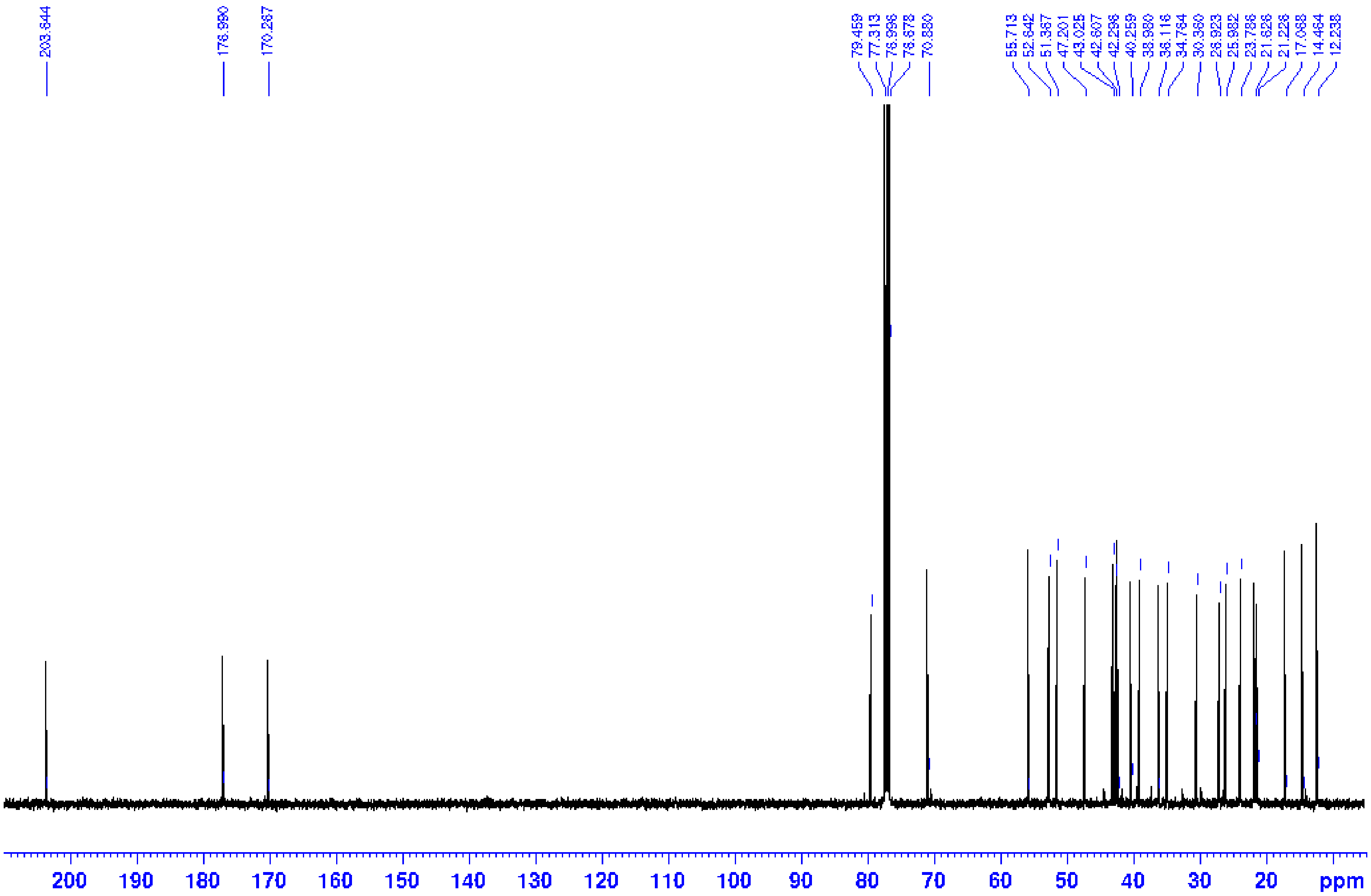

**Figure S21**. $^{13}C$ NMR spectrum of Methyl (20*S*)-3*β*-Acetoxypregn-5*α*-bromo-6-oxo-20-carboxylate (**27**).

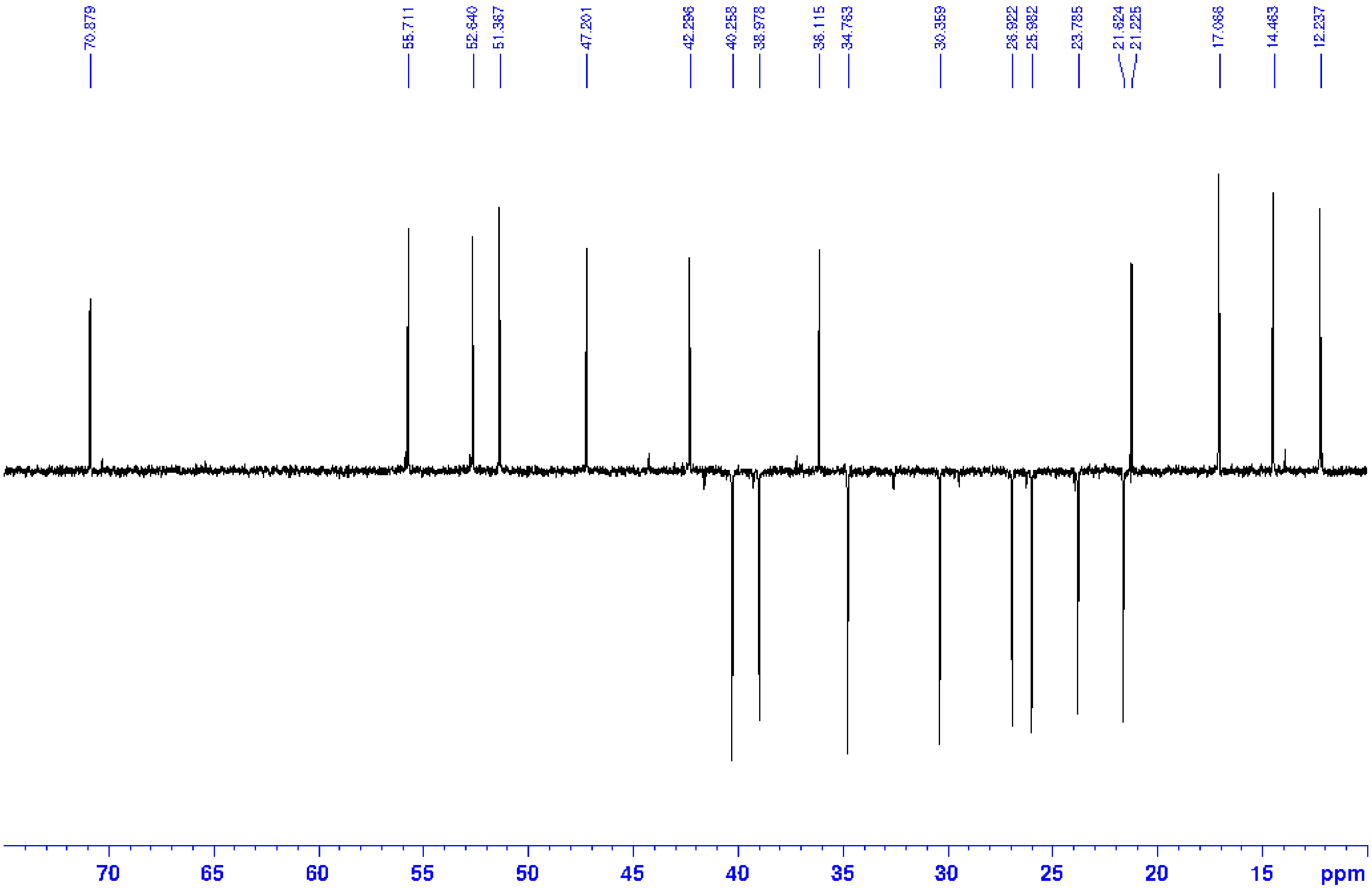


**Figure S22**. $^{13}$C DEPT-135 NMR spectrum of Methyl (20*S*)-3*β*-Acetoxypregn-5*α*-bromo-6-oxo-20-carboxylate (**27**).

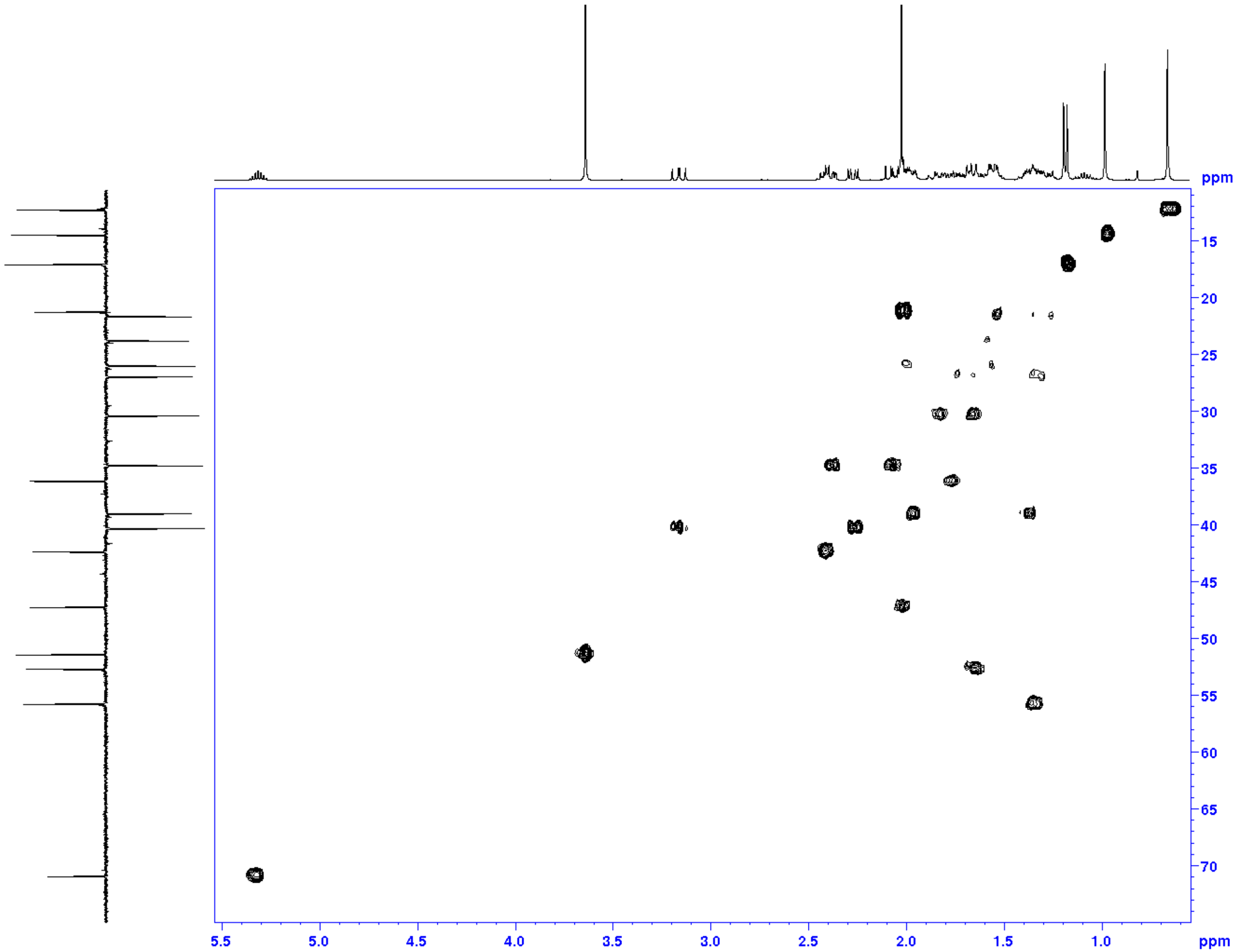


**Figure S23**. 2D HSQC NMR spectrum of Methyl (20*S*)-3*β*-Acetoxypregn-5*α*-bromo-6-oxo-20-carboxylate (**27**).

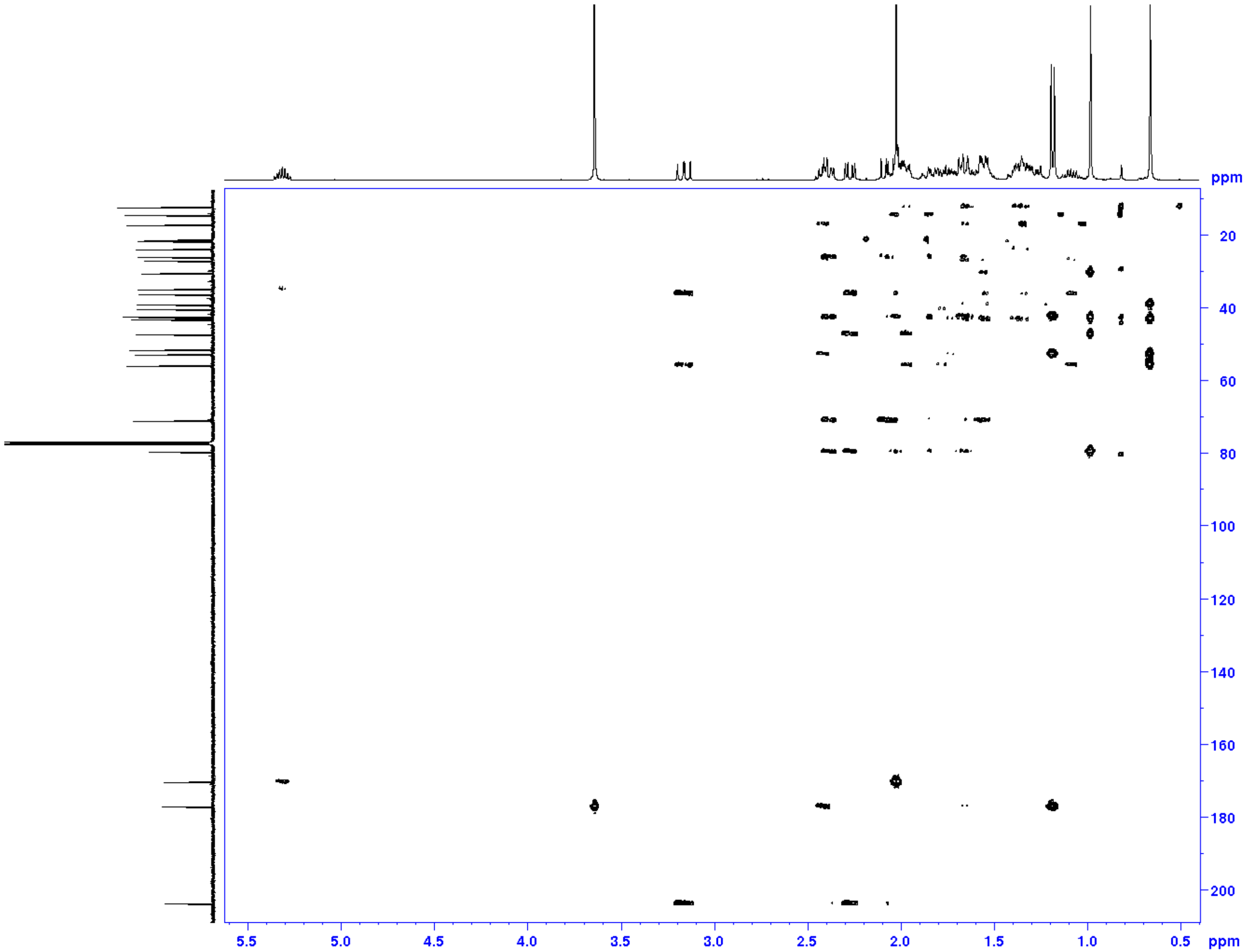


**Figure S24**. 2D HMBC NMR spectrum of Methyl (20*S*)-3*β*-Acetoxypregn-5*α*-bromo-6-oxo-20-carboxylate (**27**).

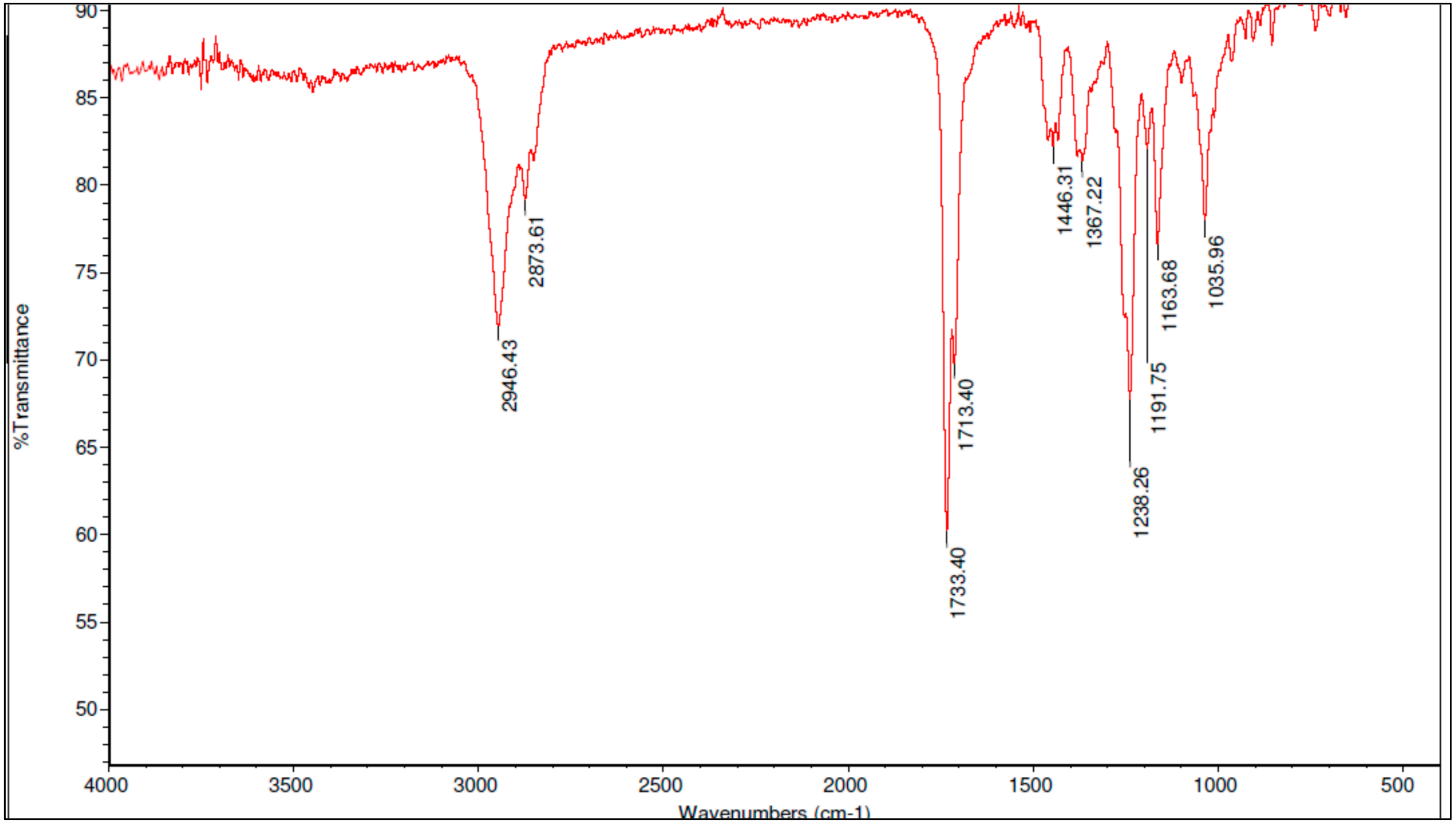


**Figure S25**. IR spectrum of Methyl (20*S*)-3*β*-Acetoxy-5*α*-pregn-6-oxo-20-carboxylate (**28**).

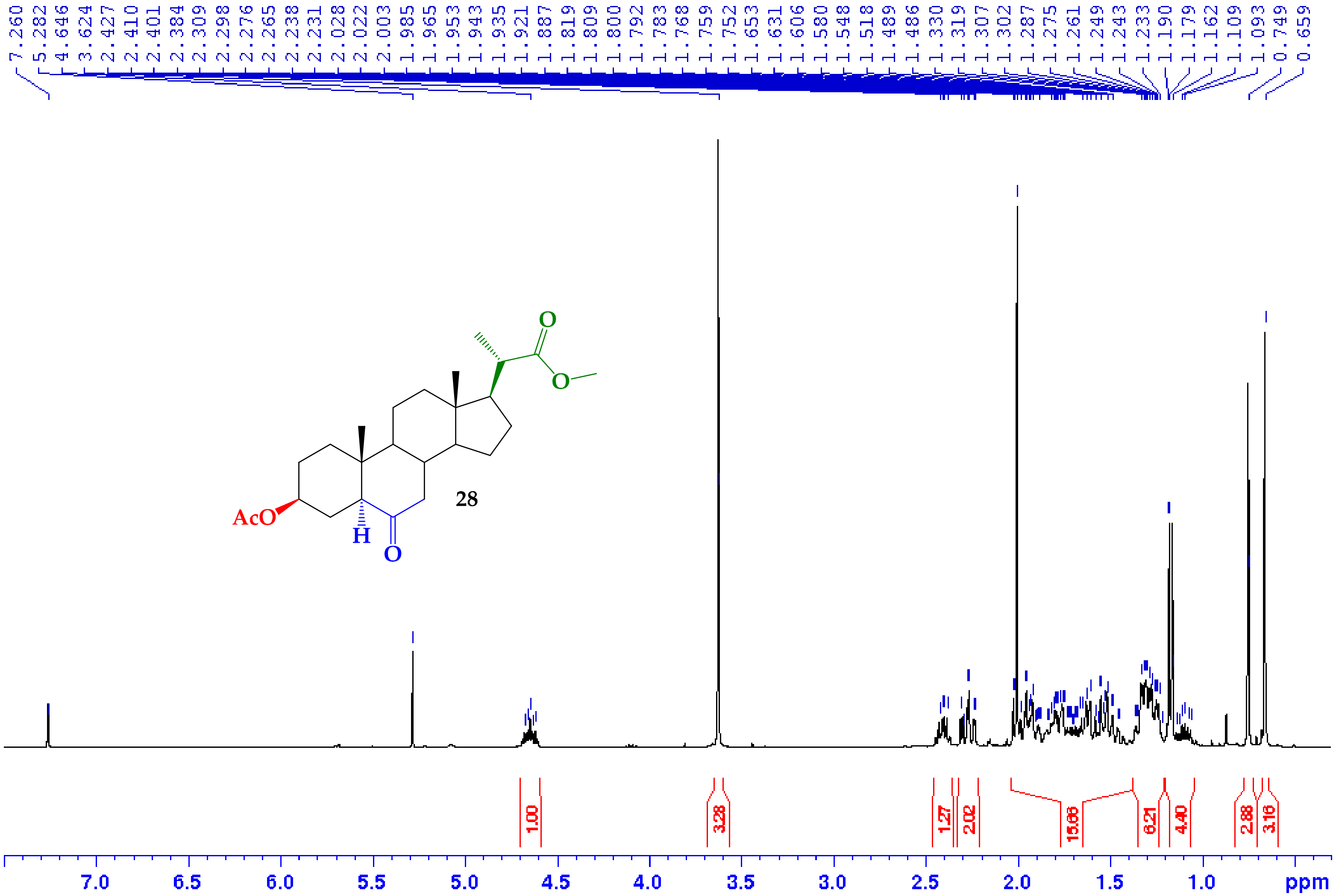

**Figure S26**. $^{1}$H NMR spectrum of Methyl (20*S*)-3*β*-Acetoxy-5*α*-pregn-6-oxo-20-carboxylate (**28**).

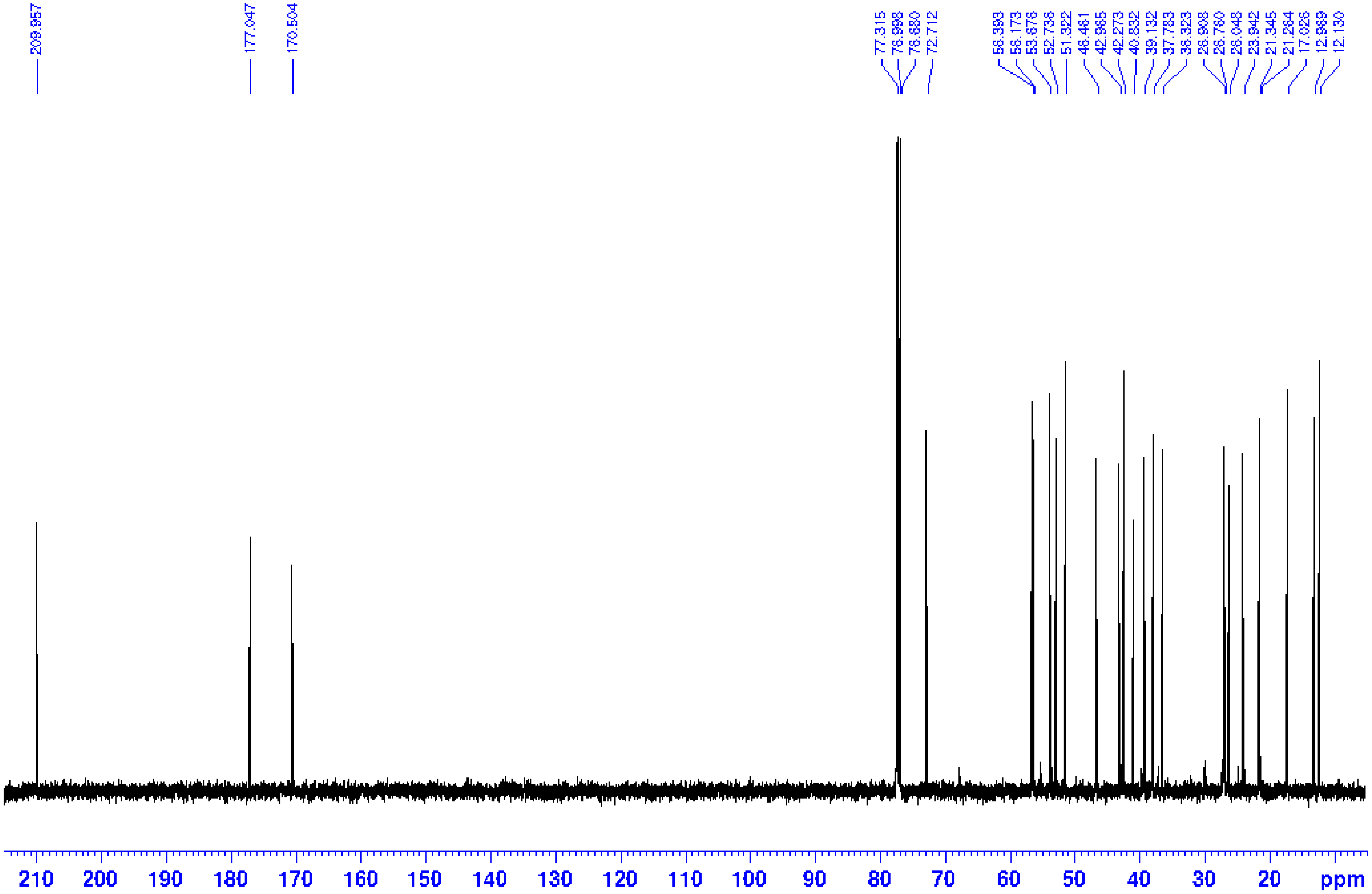


**Figure S27**. $^{13}$C NMR spectrum of Methyl (20*S*)-3*β*-Acetoxy-5*α*-pregn-6-oxo-20-carboxylate (**28**).

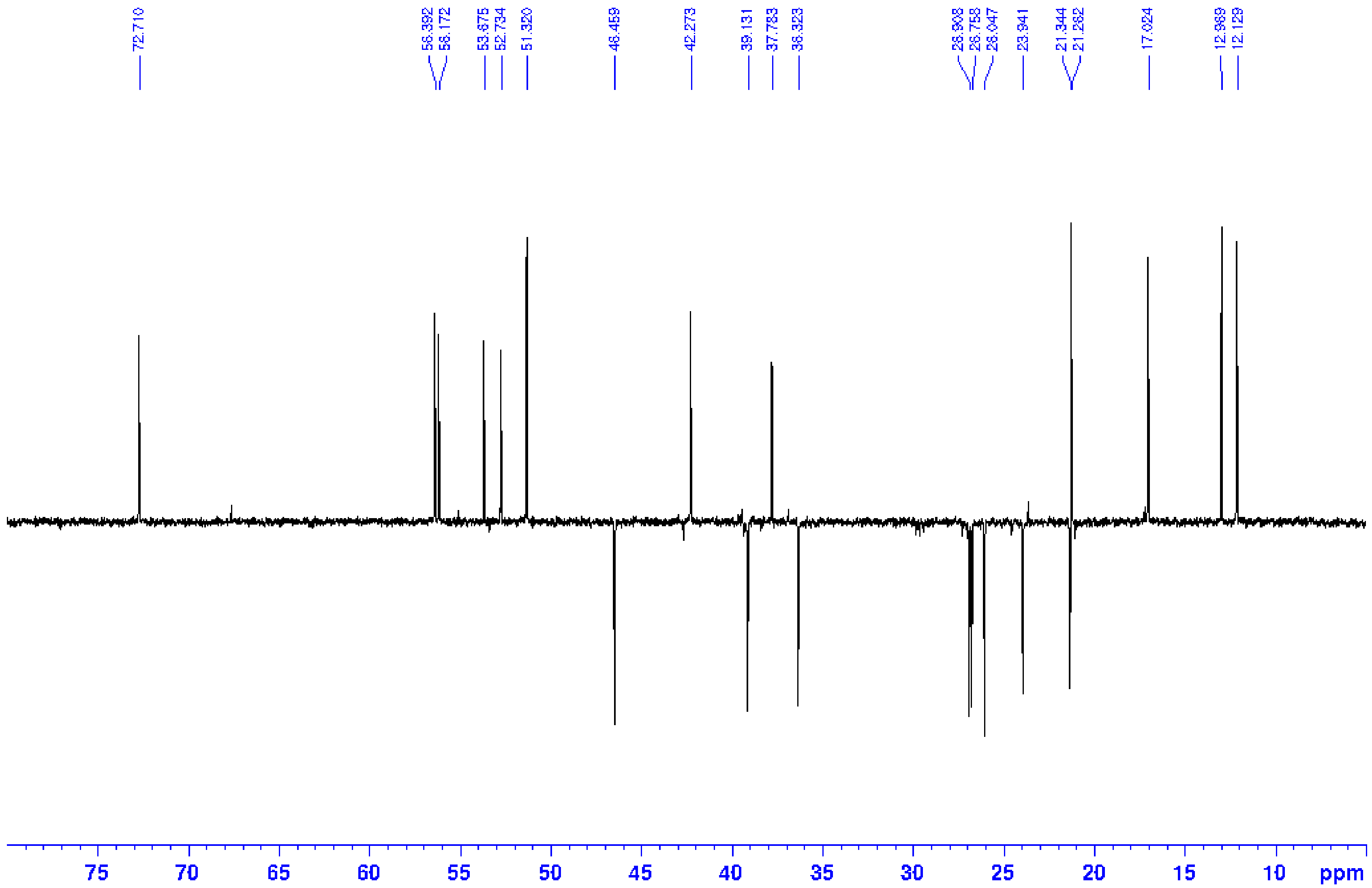


**Figure S28.** $^{13}C$ DEPT-135 NMR spectrum of Methyl (20*S*)-3*β*-Acetoxy-5*α*-pregn-6-oxo-20-carboxylate (**28**).

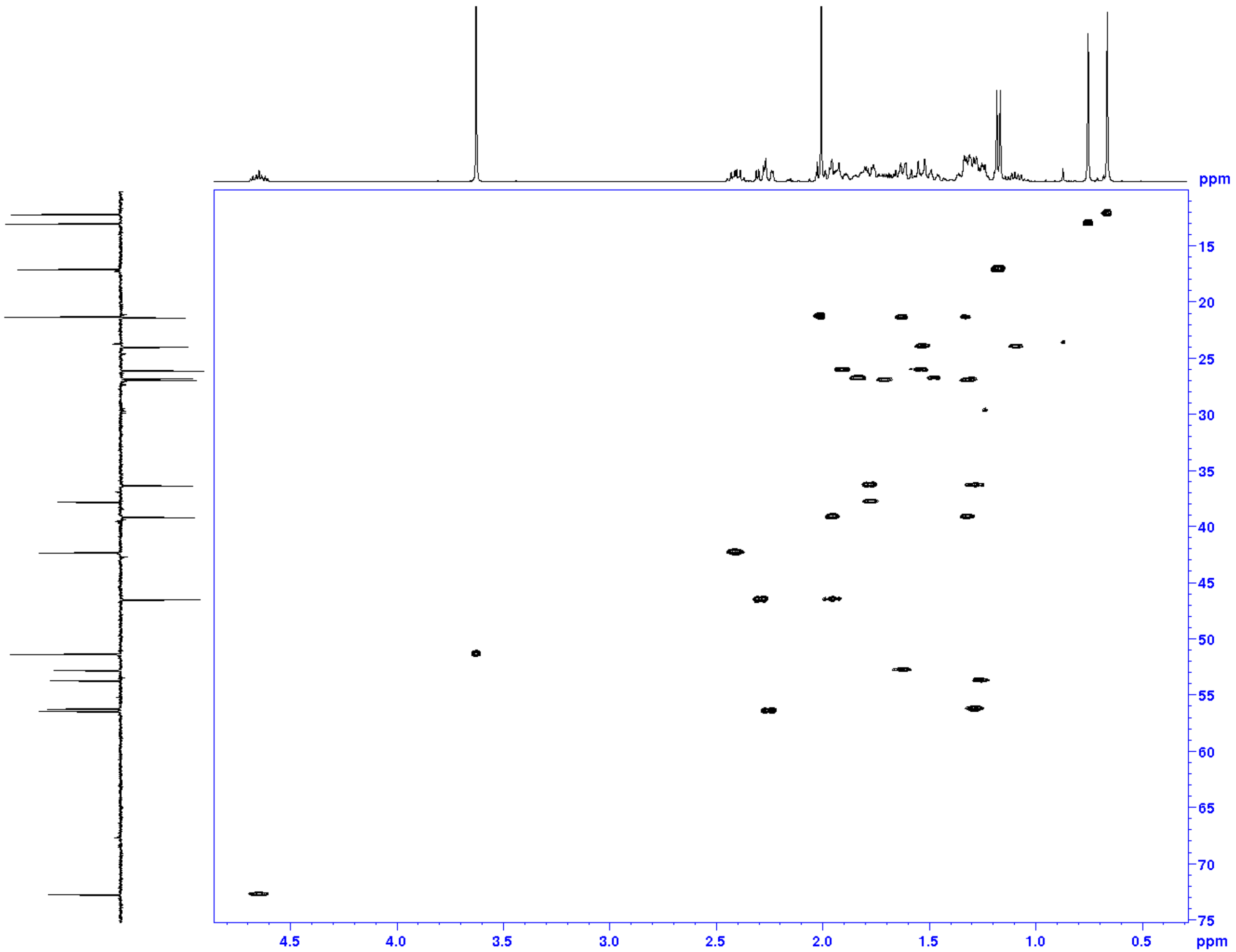


**Figure S29**. 2D HSQC NMR spectrum of Methyl (20*S*)-3*β*-Acetoxy-5*α*-pregn-6-oxo-20-carboxylate (**28**).

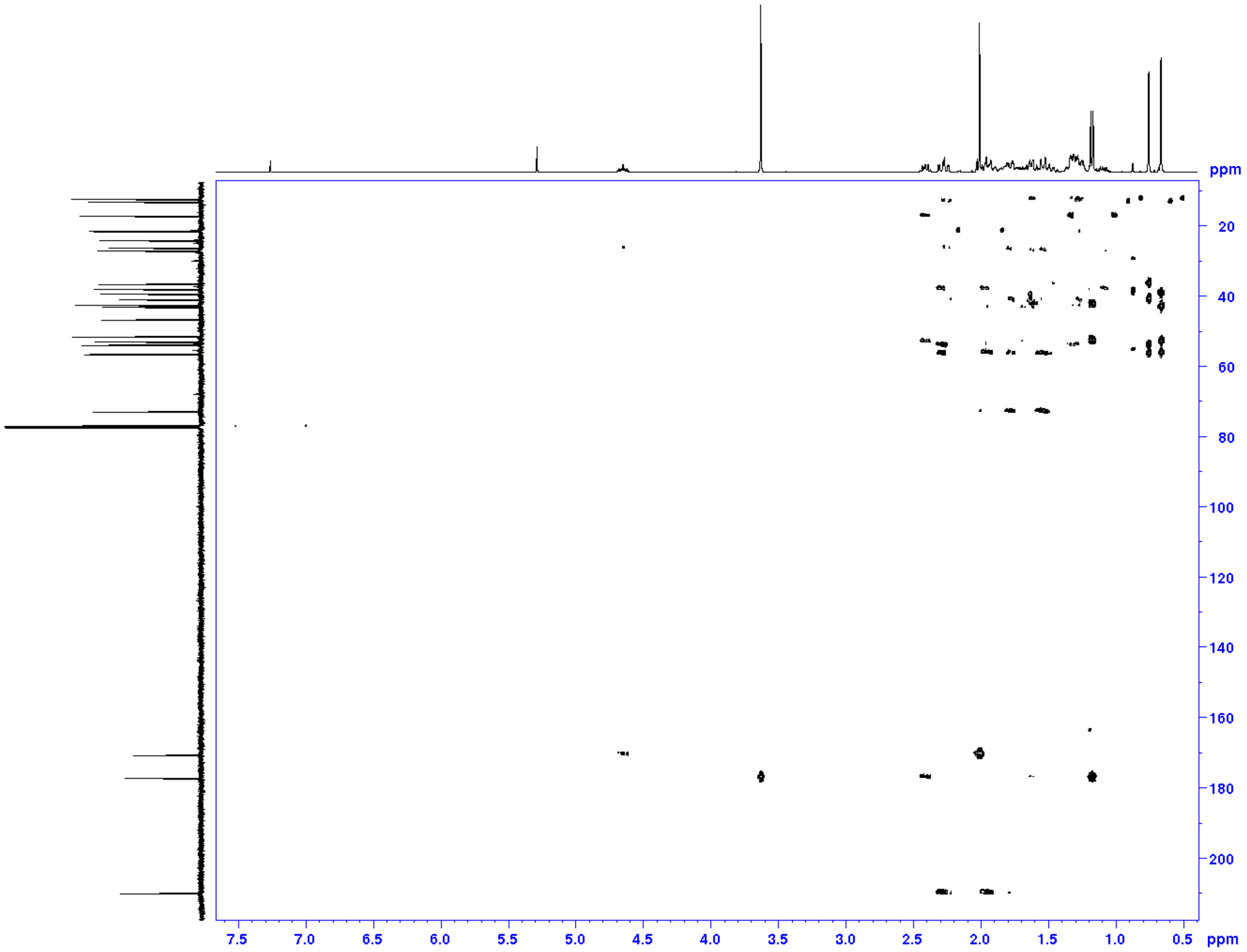


**Figure S30**. 2D HMBC NMR spectrum of Methyl (20*S*)-3*β*-Acetoxy-5*α*-pregn-6-oxo-20-carboxylate (**28**).

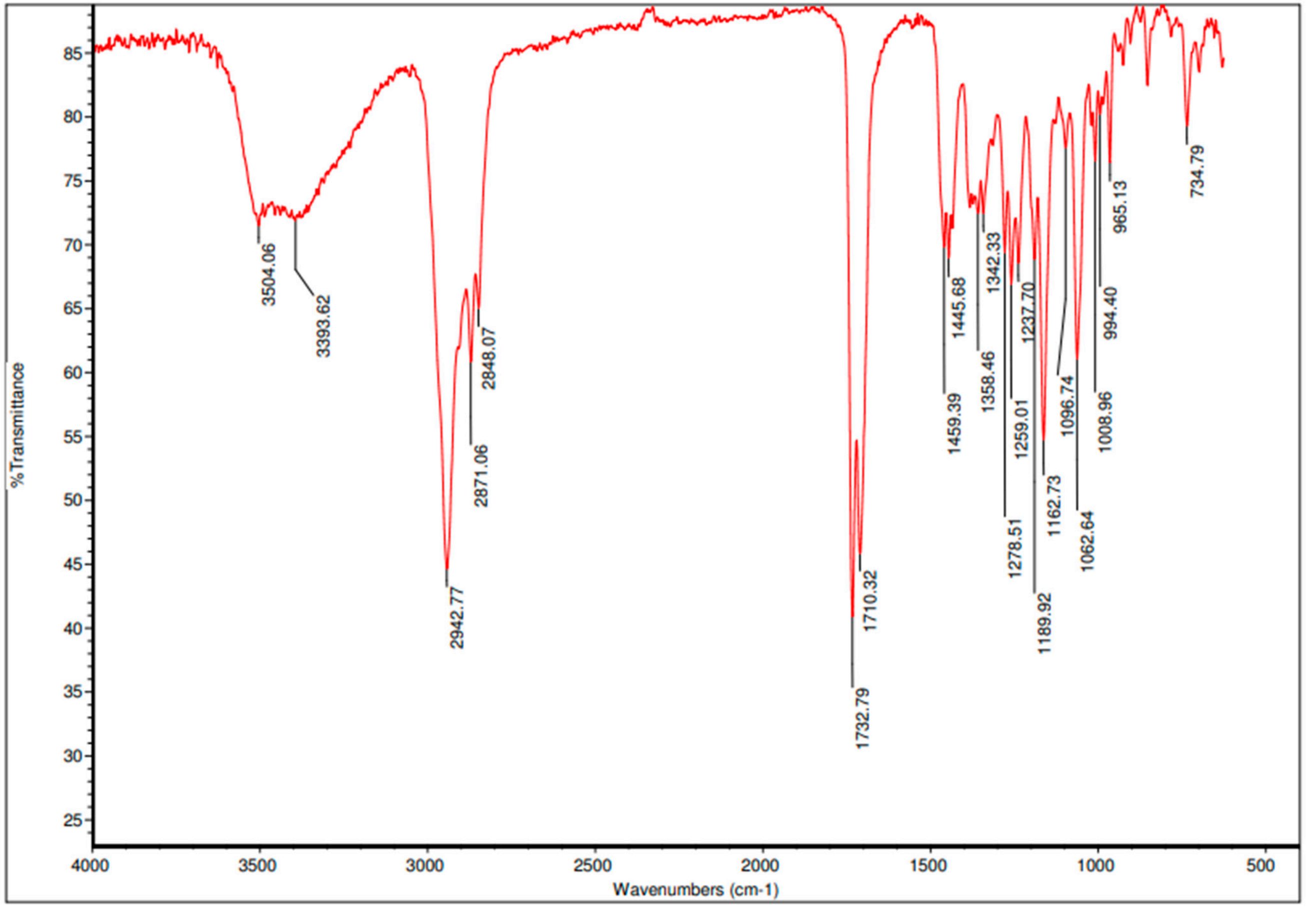

**Figure S31**. IR spectrum of Methyl (20*S*)-3*β*-hydroxy-5*α*-pregn-6-oxo-20-carboxylate (**29**).

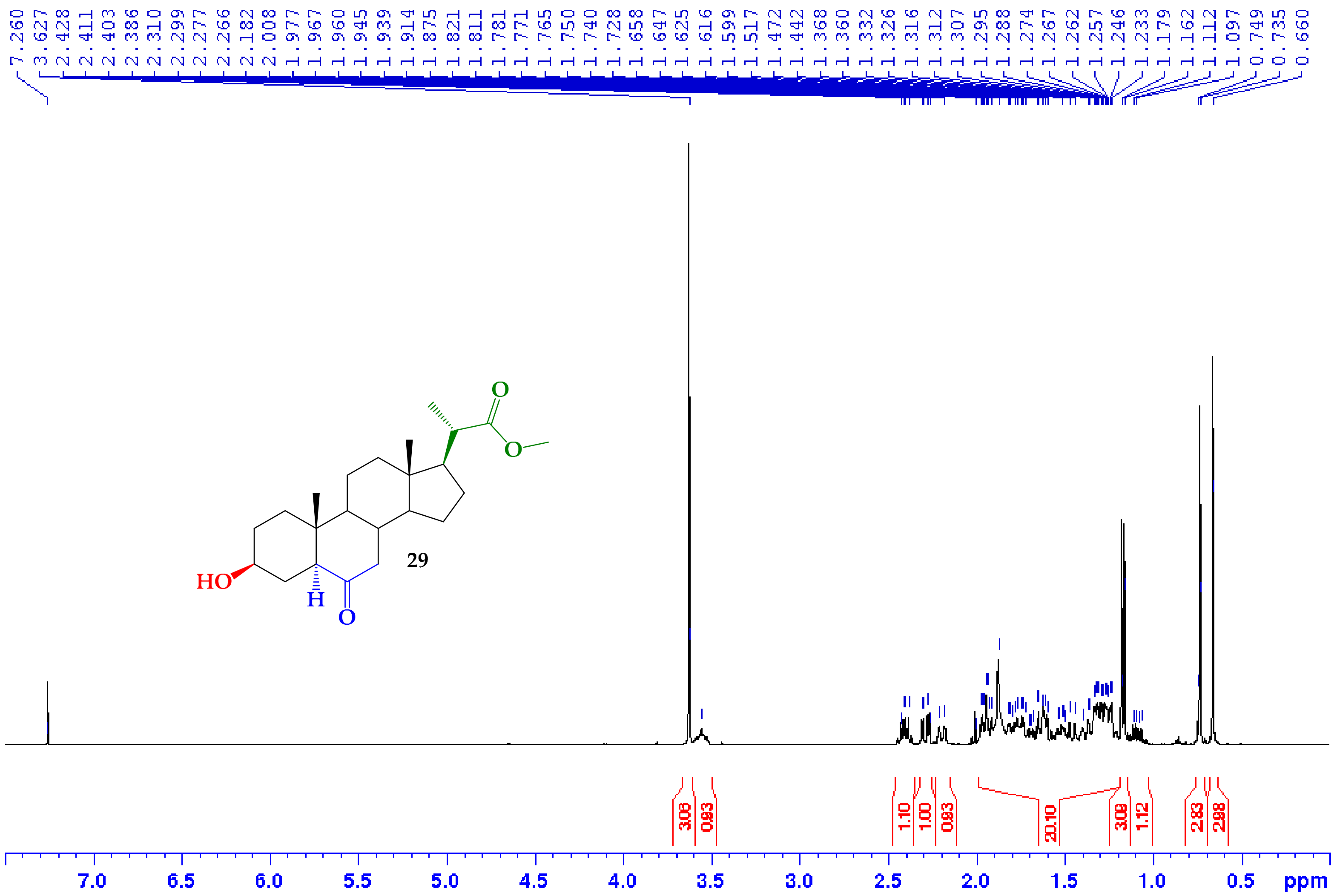

**Figure S32**. $^1H$ NMR spectrum of Methyl (20*S*)-3*β*-hydroxy-5*α*-pregn-6-oxo-20-carboxylate (**29**).

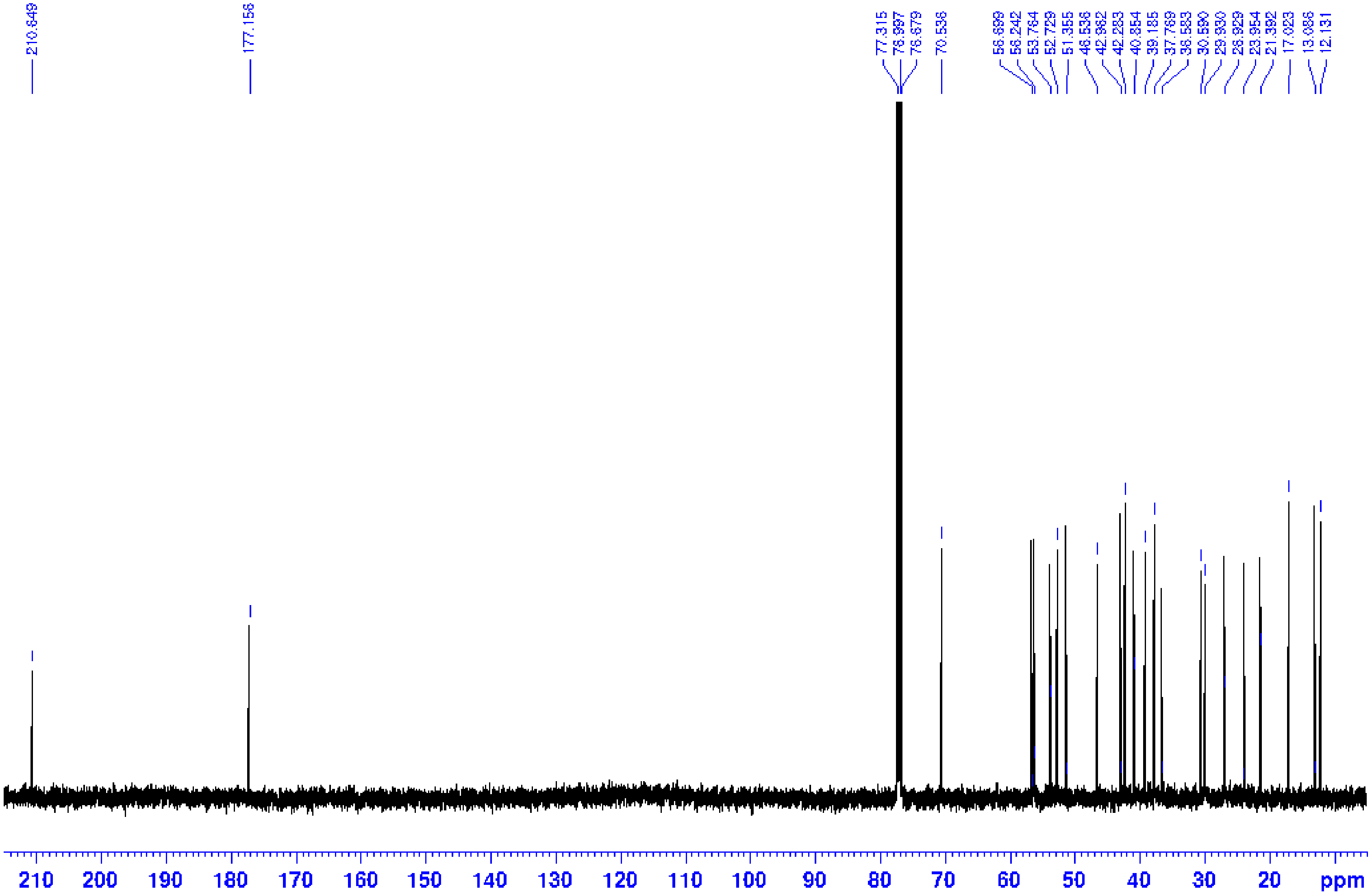

**Figure S33**. $^{13}C$ NMR spectrum of Methyl (20*S*)-3*β*-hydroxy-5*α*-pregn-6-oxo-20-carboxylate (**29**).

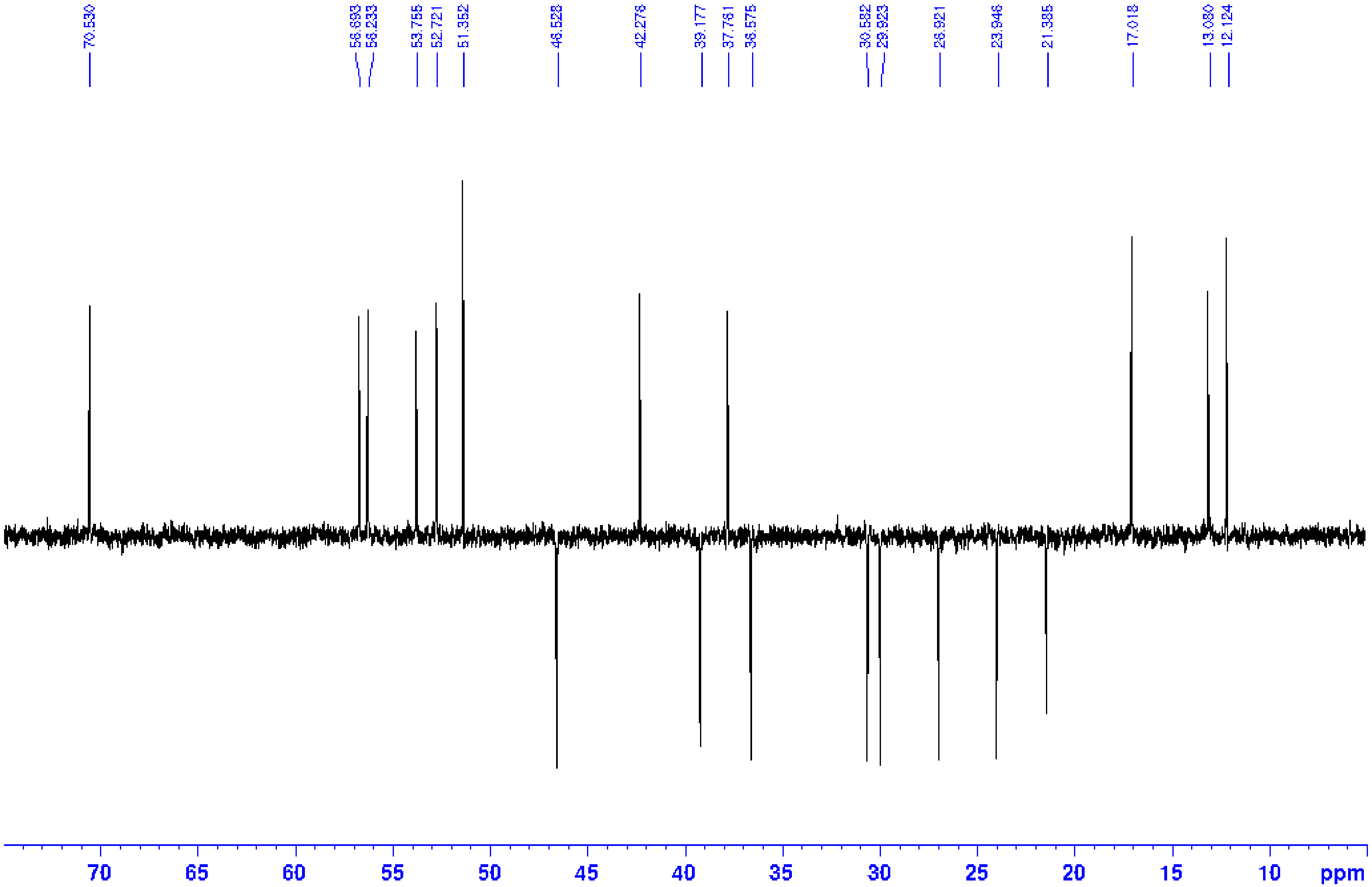


**Figure S34**. $^{13}$C DEPT-135 NMR spectrum of Methyl (20*S*)-3*β*-hydroxy-5*α*-pregn-6-oxo-20-carboxylate (**29**).

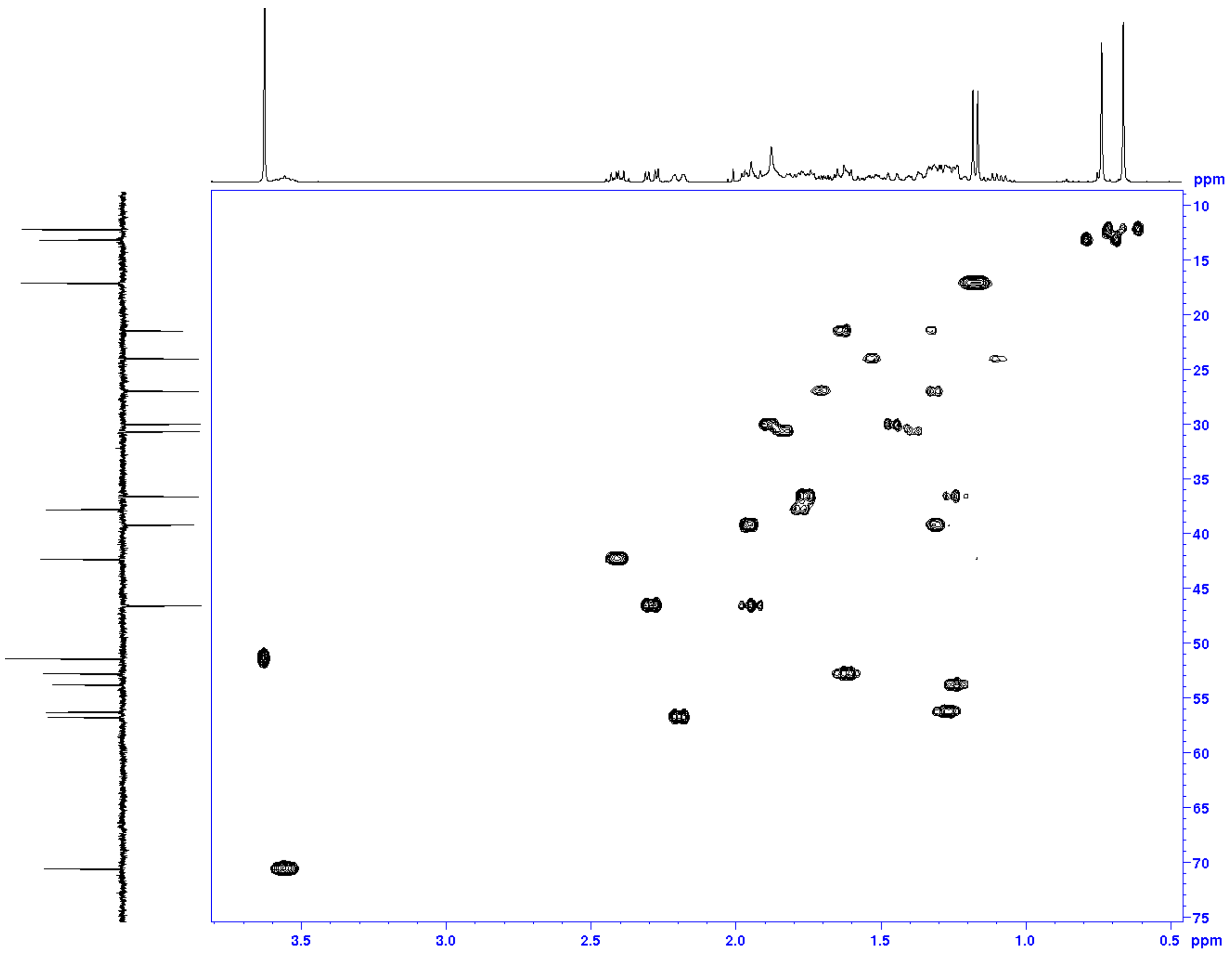


**Figure S35**. 2D HSQC NMR spectrum of Methyl (20*S*)-3*β*-hydroxy-5*α*-pregn-6-oxo-20-carboxylate (**29**).

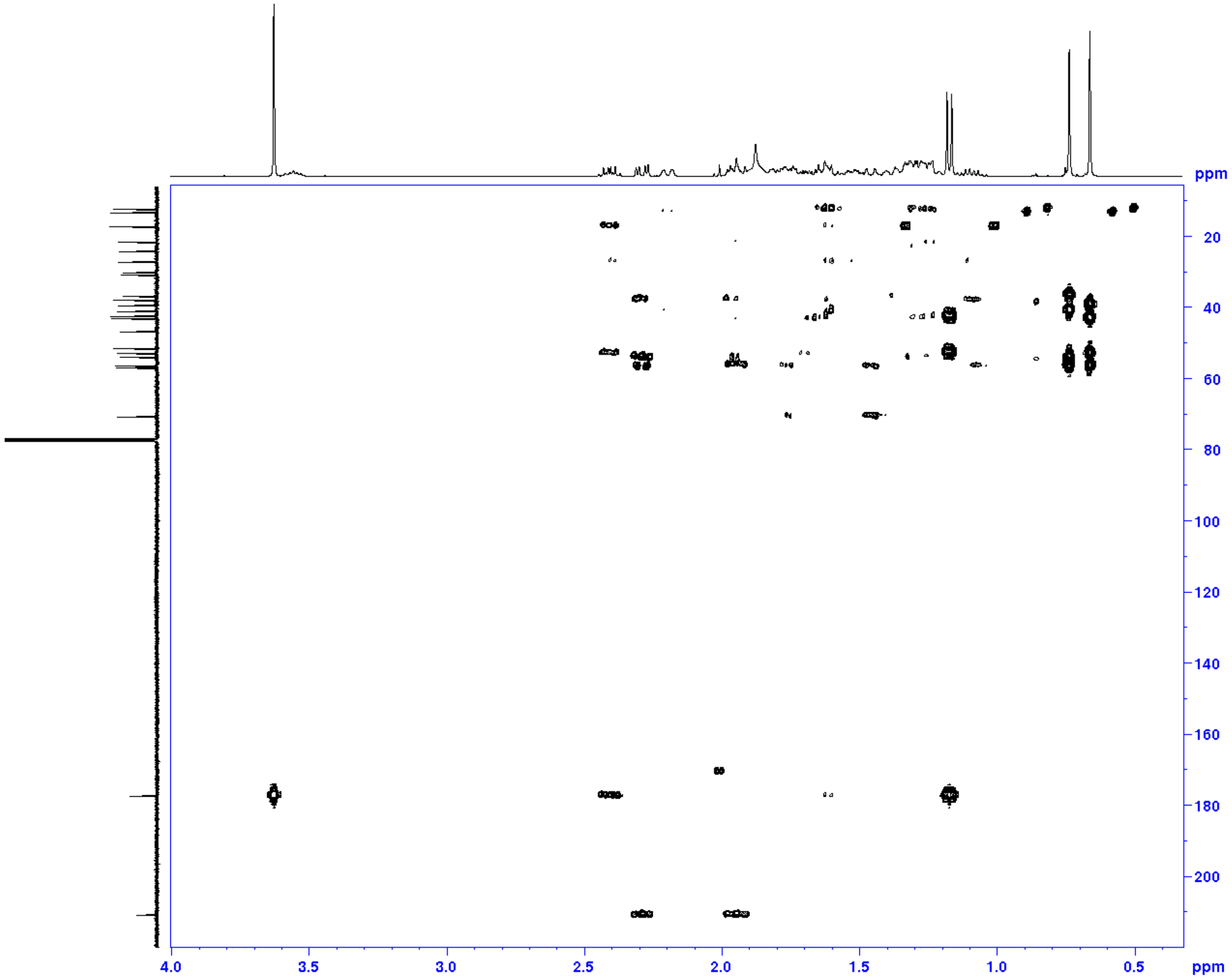


**Figure S36**. 2D HMBC NMR spectrum of Methyl (20*S*)-3*β*-hydroxy-5*α*-pregn-6-oxo-20-carboxylate (**29**).

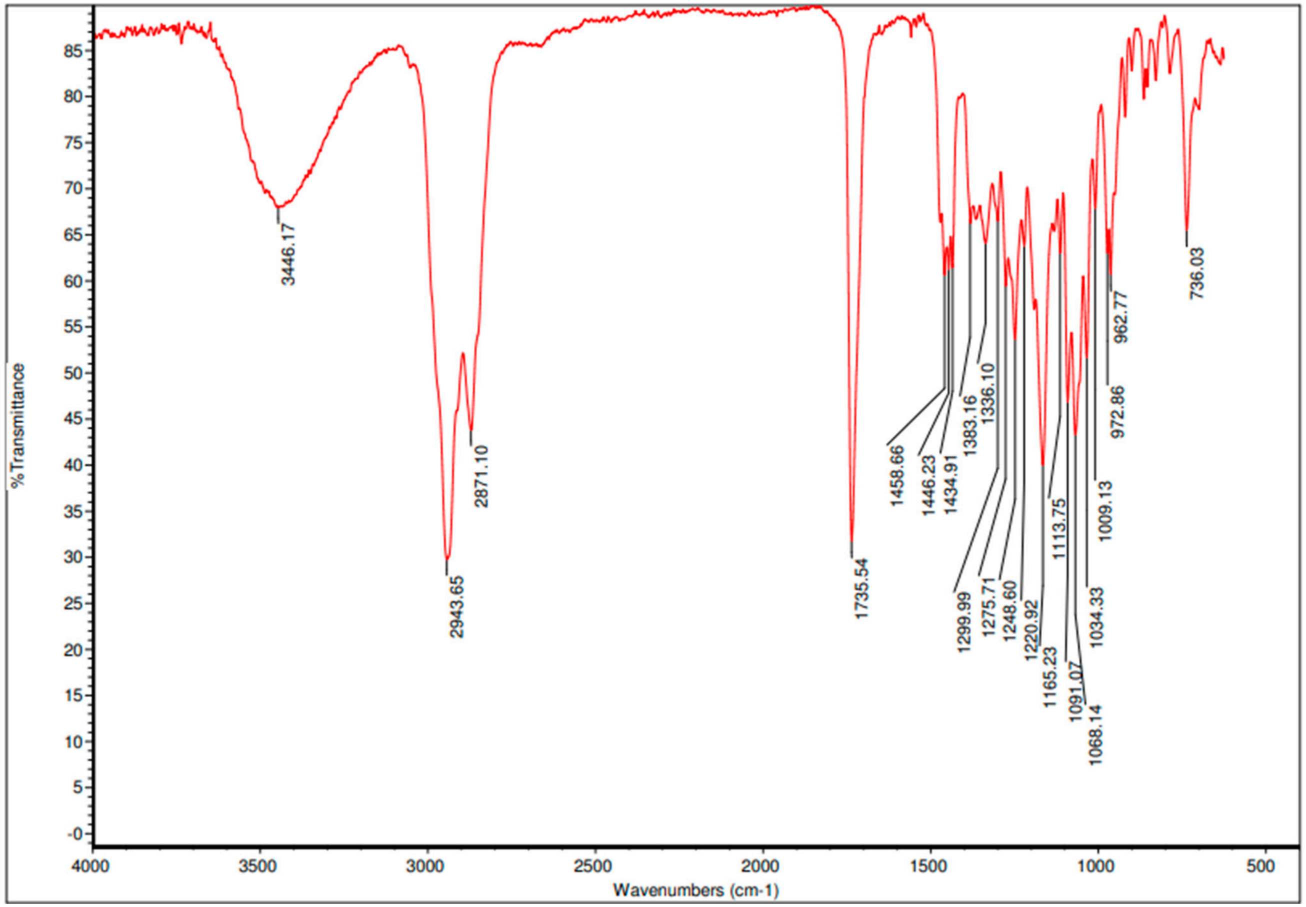

**Figure S37**. IR spectrum of Methyl (20*S*)-3*β*-hydroxy-5*α*-pregn-6-dioxolan-20-carboxylate (**30**).

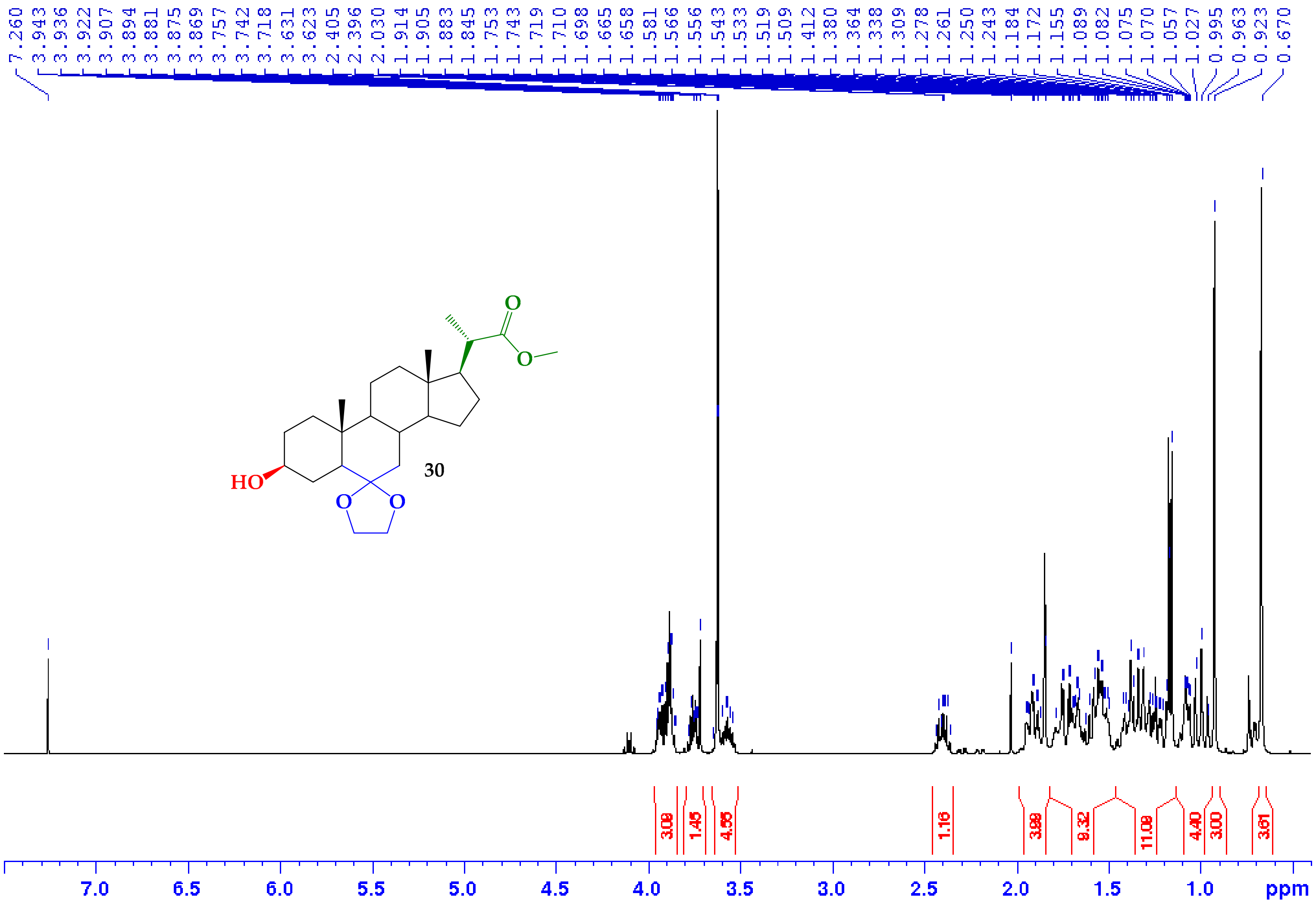

**Figure S38**. $^{1}$H NMR spectrum of Methyl (20*S*)-3*β*-hydroxy-5*α*-pregn-6-dioxolan-20-carboxylate (**30**).

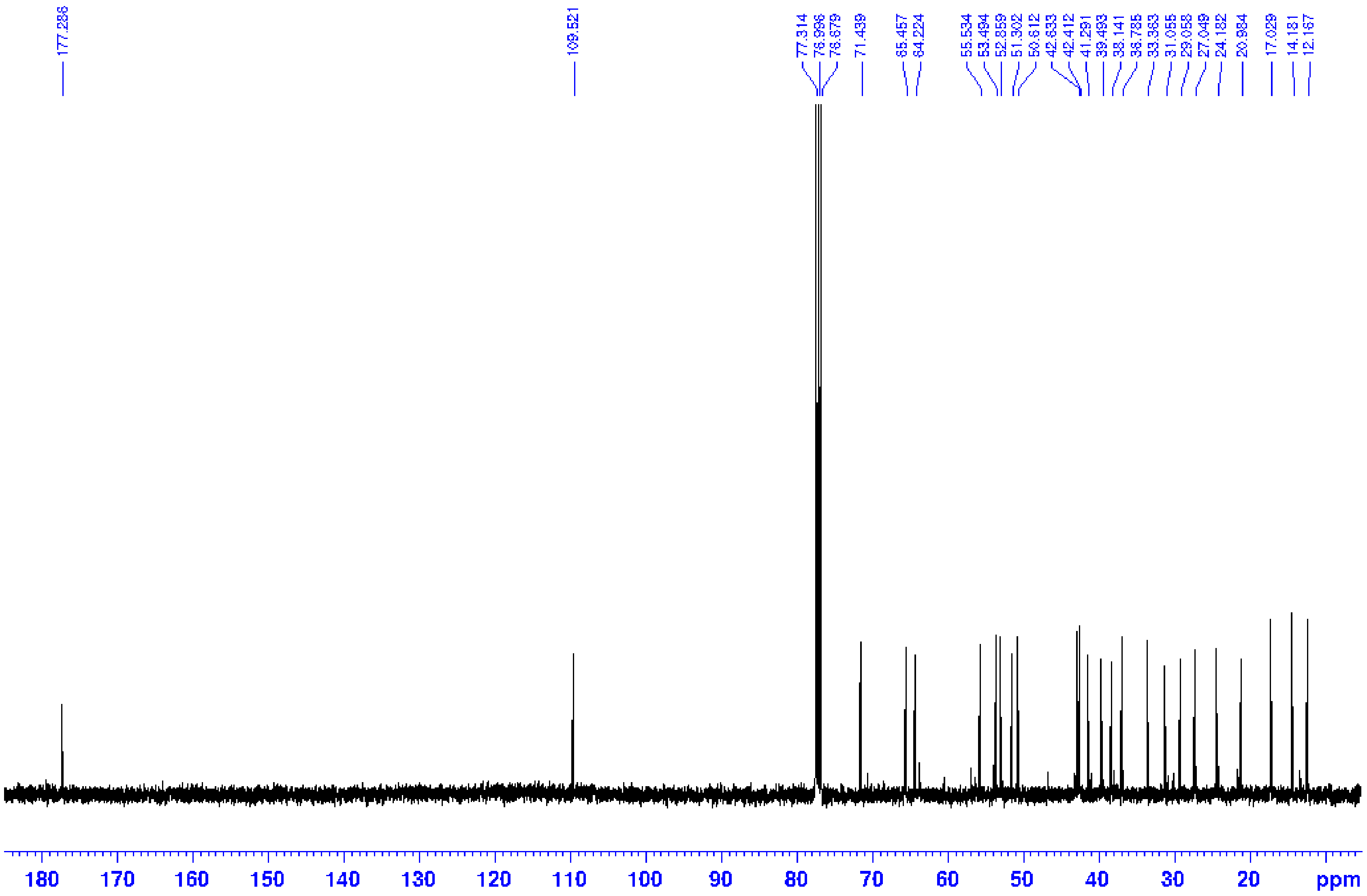


**Figure S39**. $^{13}C$ NMR spectrum of Methyl (20*S*)-3*β*-hydroxy-5*α*-pregn-6-dioxolan-20-carboxylate (**30**).

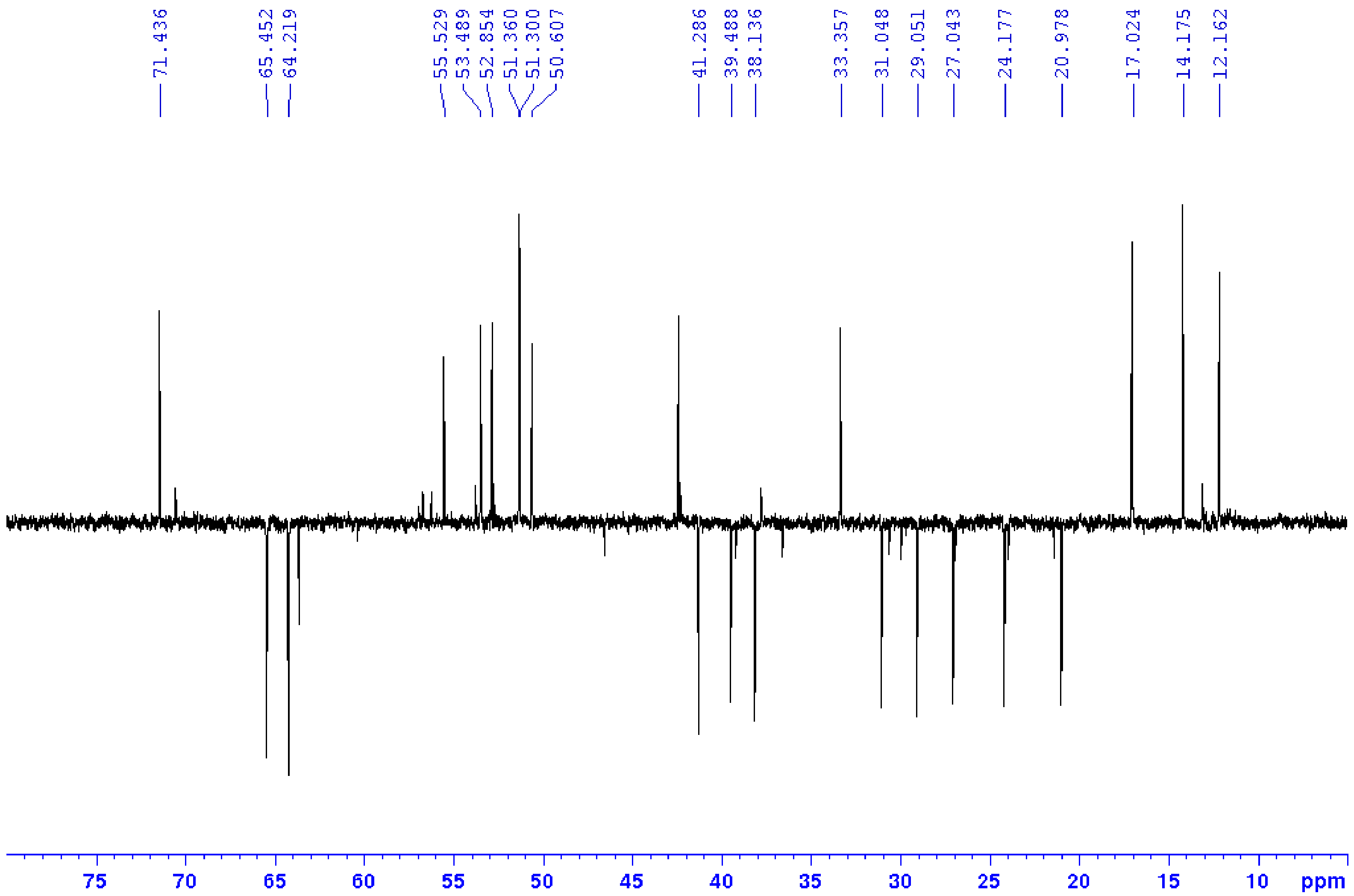


**Figure S40**. $^{13}C$ DEPT-135 NMR spectrum of Methyl (20*S*)-3*β*-hydroxy-5*α*-pregn-6-dioxolan-20-carboxylate (**30**).

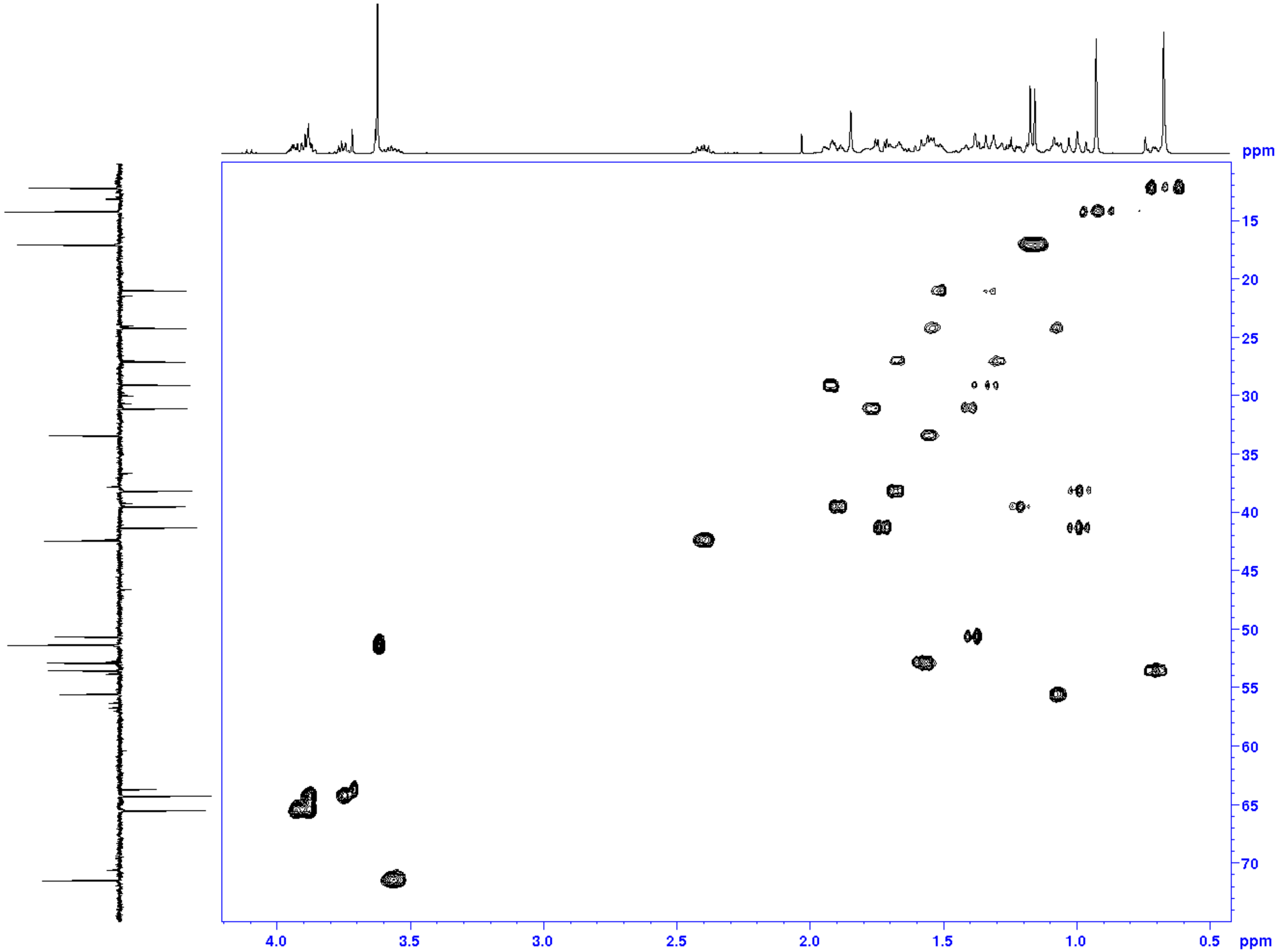


**Figure S41**. 2D HSQC NMR spectrum of Methyl (20*S*)-3*β*-hydroxy-5*α*-pregn-6-dioxolan-20-carboxylate (**30**).

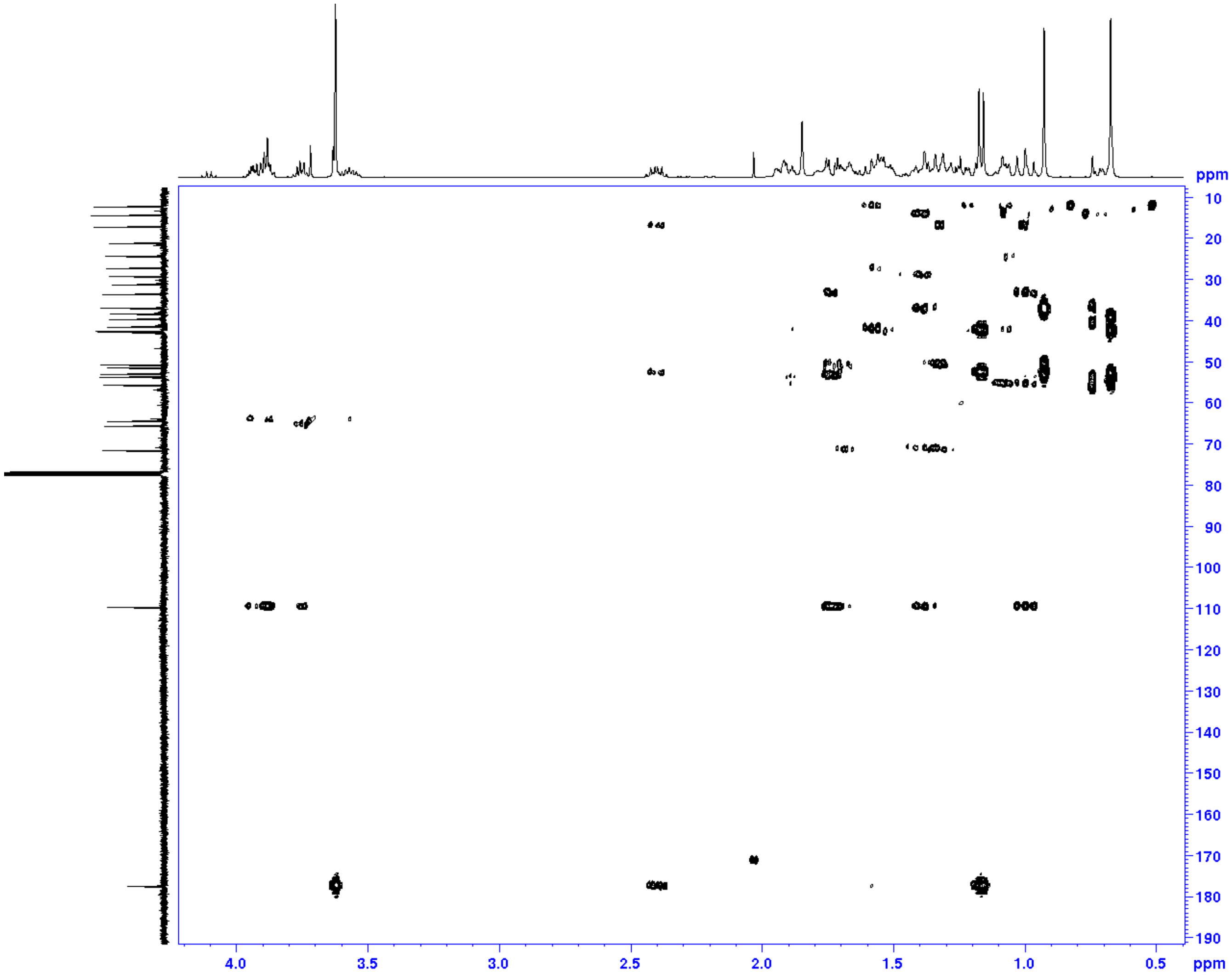


**Figure S42**. 2D HMBC NMR spectrum of Methyl (20*S*)-3*β*-hydroxy-5*α*-pregn-6-dioxolan-20-carboxylate (**30**).

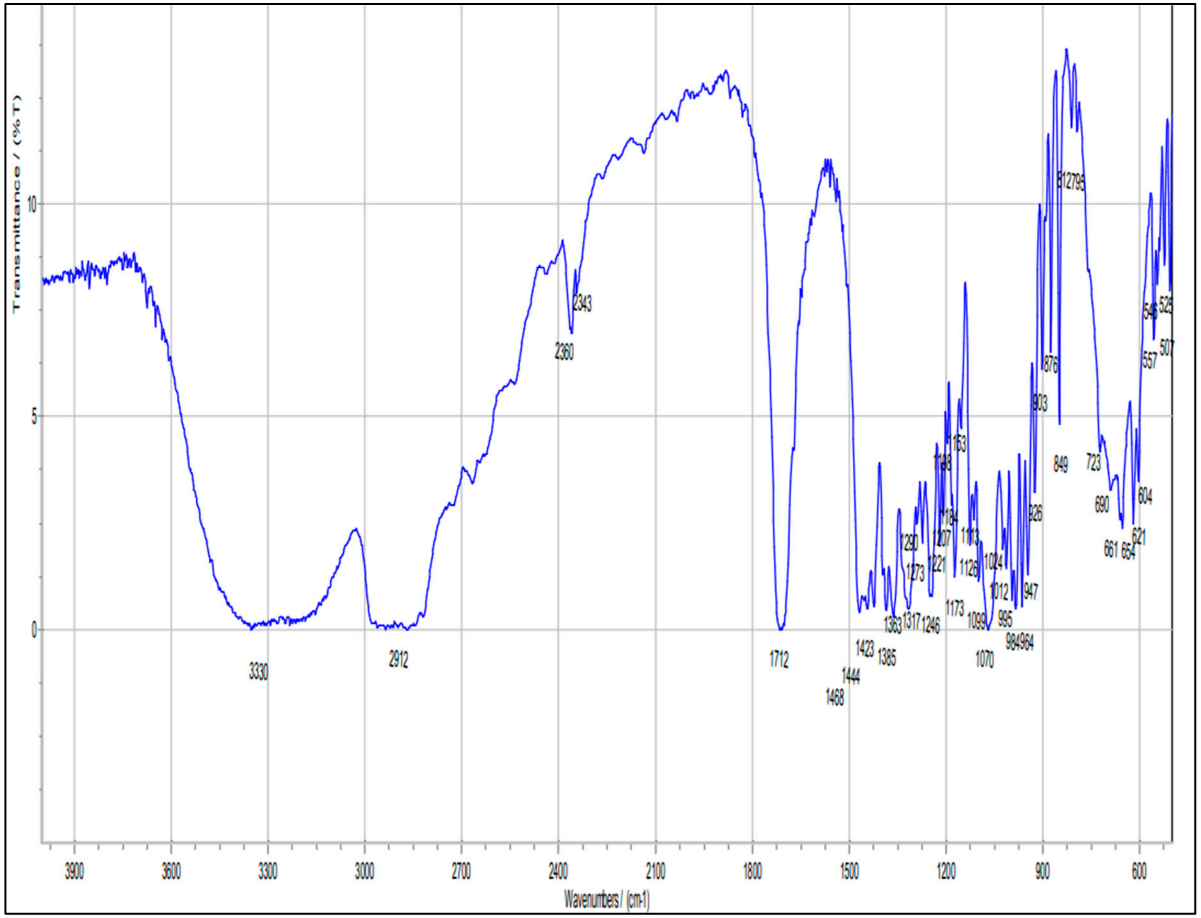


**Figure S43**. IR spectrum of 3β-22-dihydroxy-5α-cholan-23,24-dinor-6-oxa (**31**).

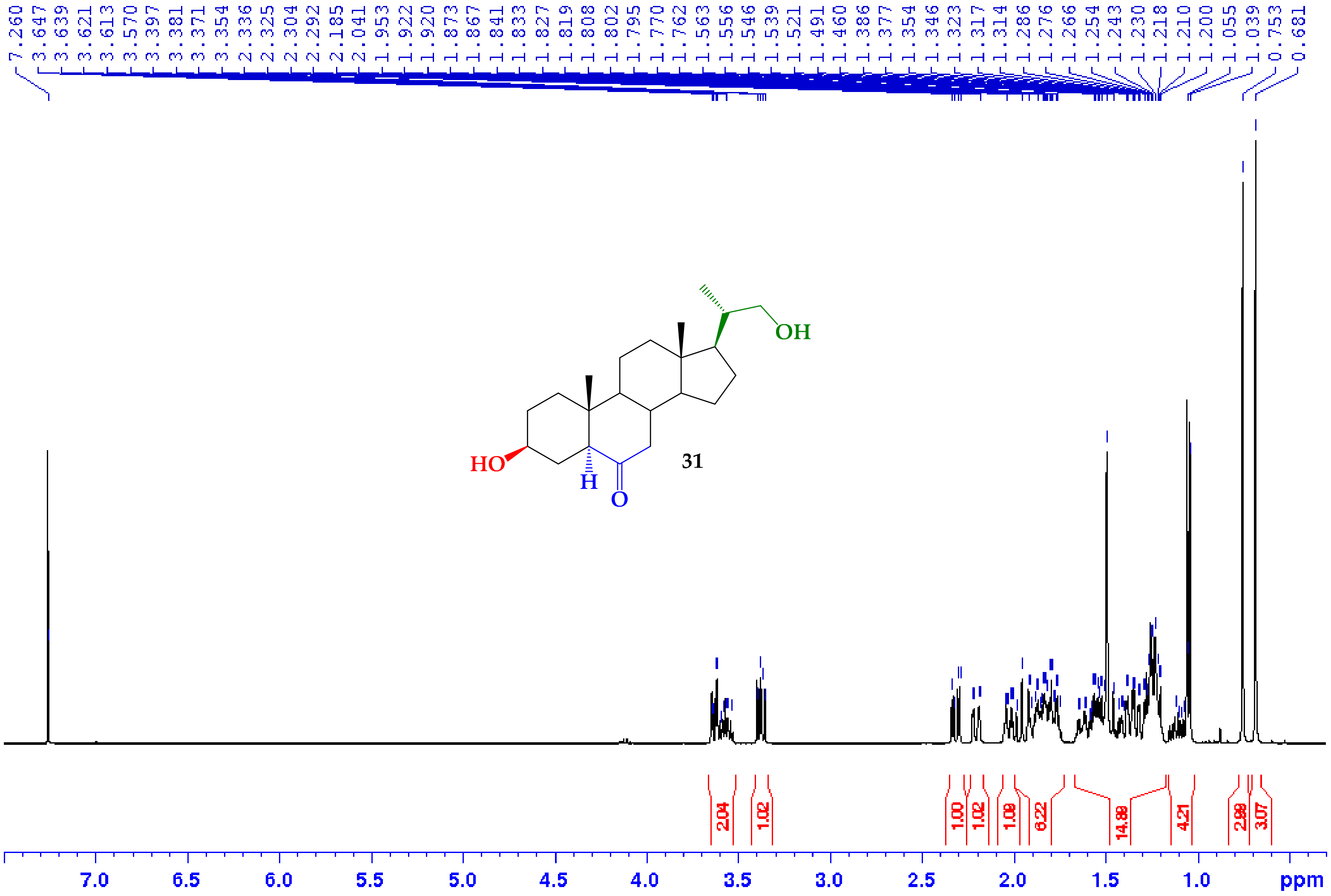

**Figure S44**. $^{1}$H NMR spectrum of 3β-22-dihydroxy-5α-cholan-23,24-dinor-6-oxa (**31**).

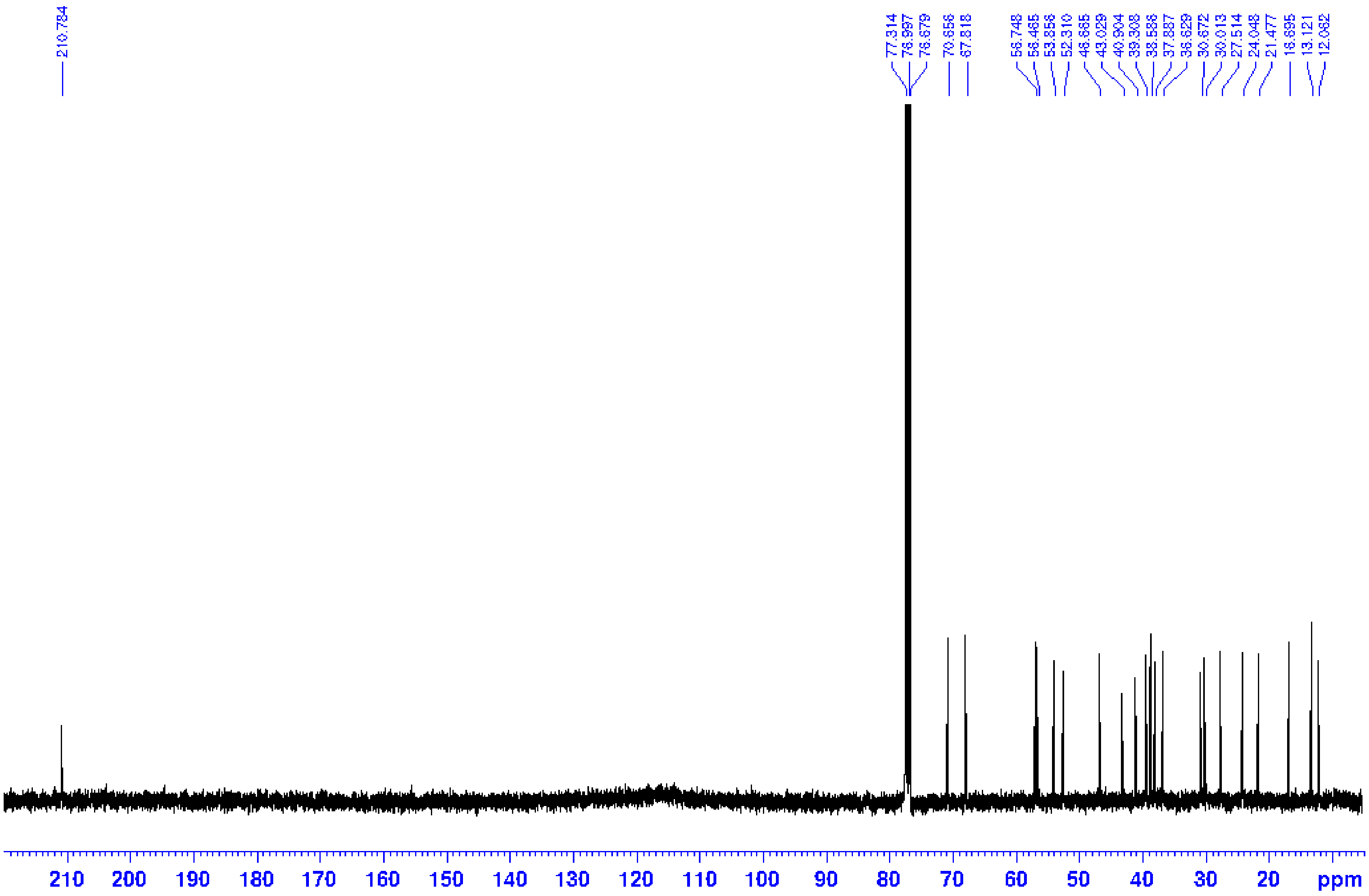


**Figure S45**. $^{13}$C NMR spectrum of 3β-22-dihydroxy-5α-cholan-23,24-dinor-6-oxa (**31**).

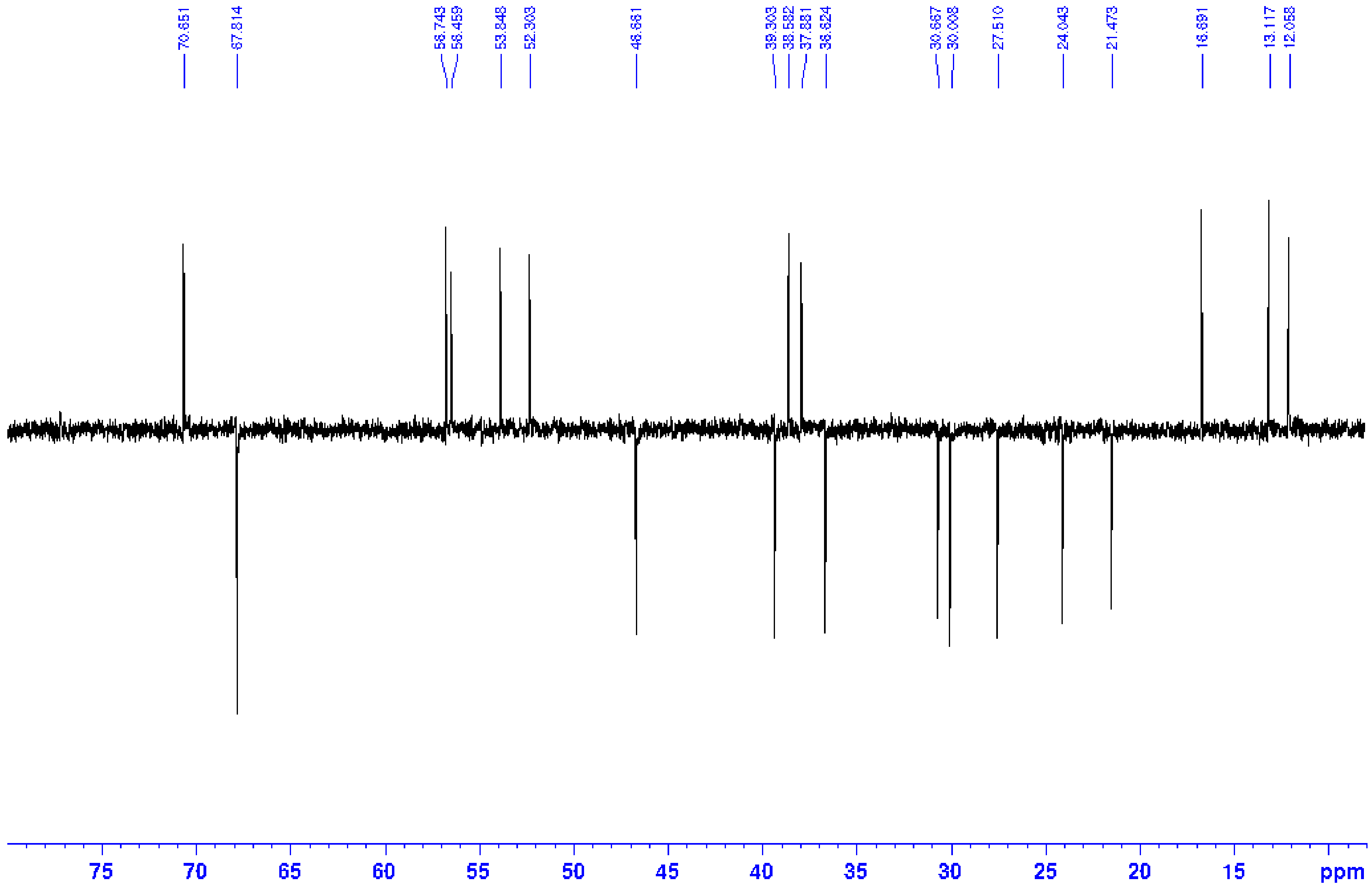


**Figure S46**. $^{13}$C DEPT-135 NMR spectrum of 3β-22-dihydroxy-5α-cholan-23,24-dinor-6-oxa (**31**).

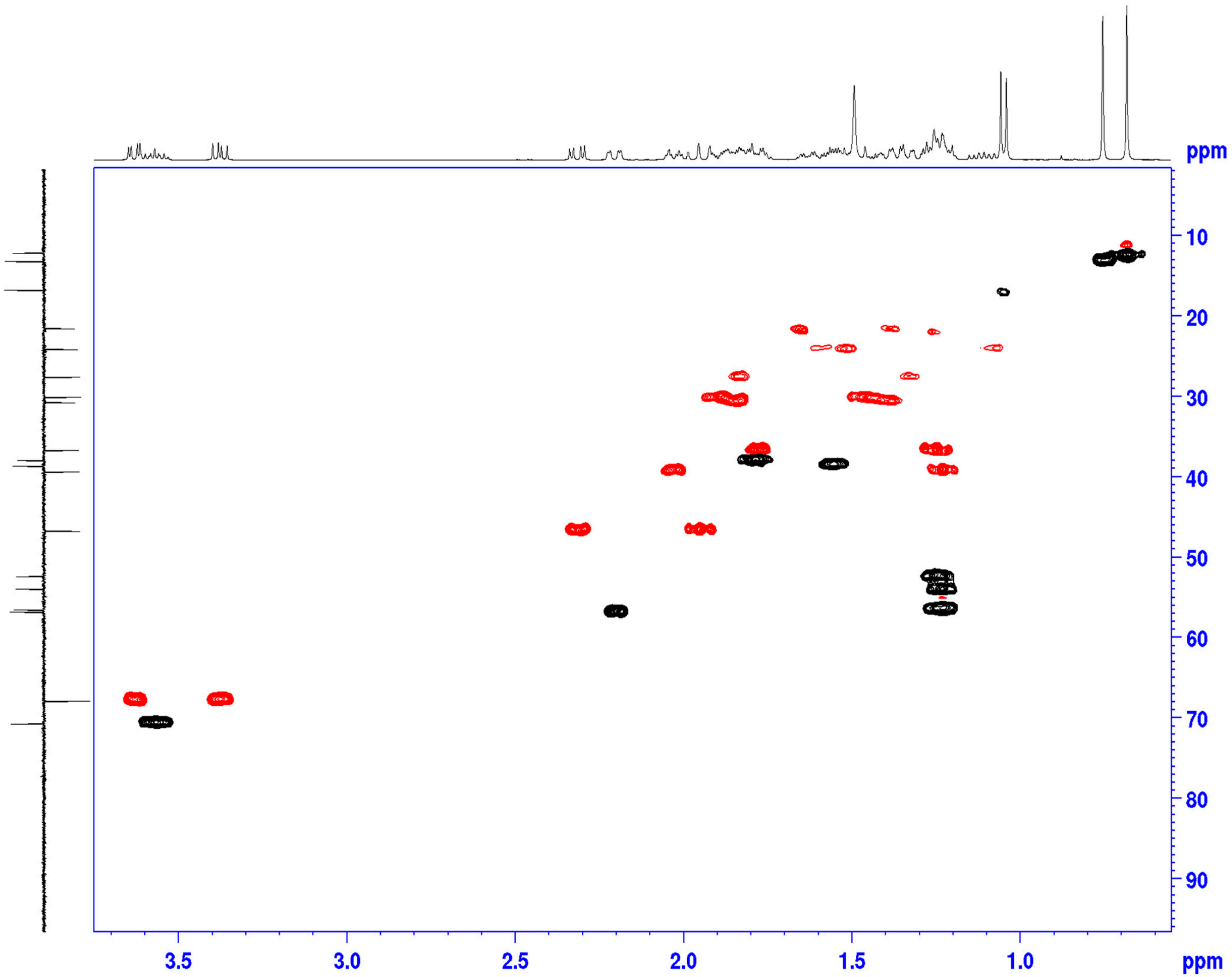


**Figure S47**. 2D HSQC NMR spectrum of 3β-22-dihydroxy-5α-cholan-23,24-dinor-6-oxa (**31**).

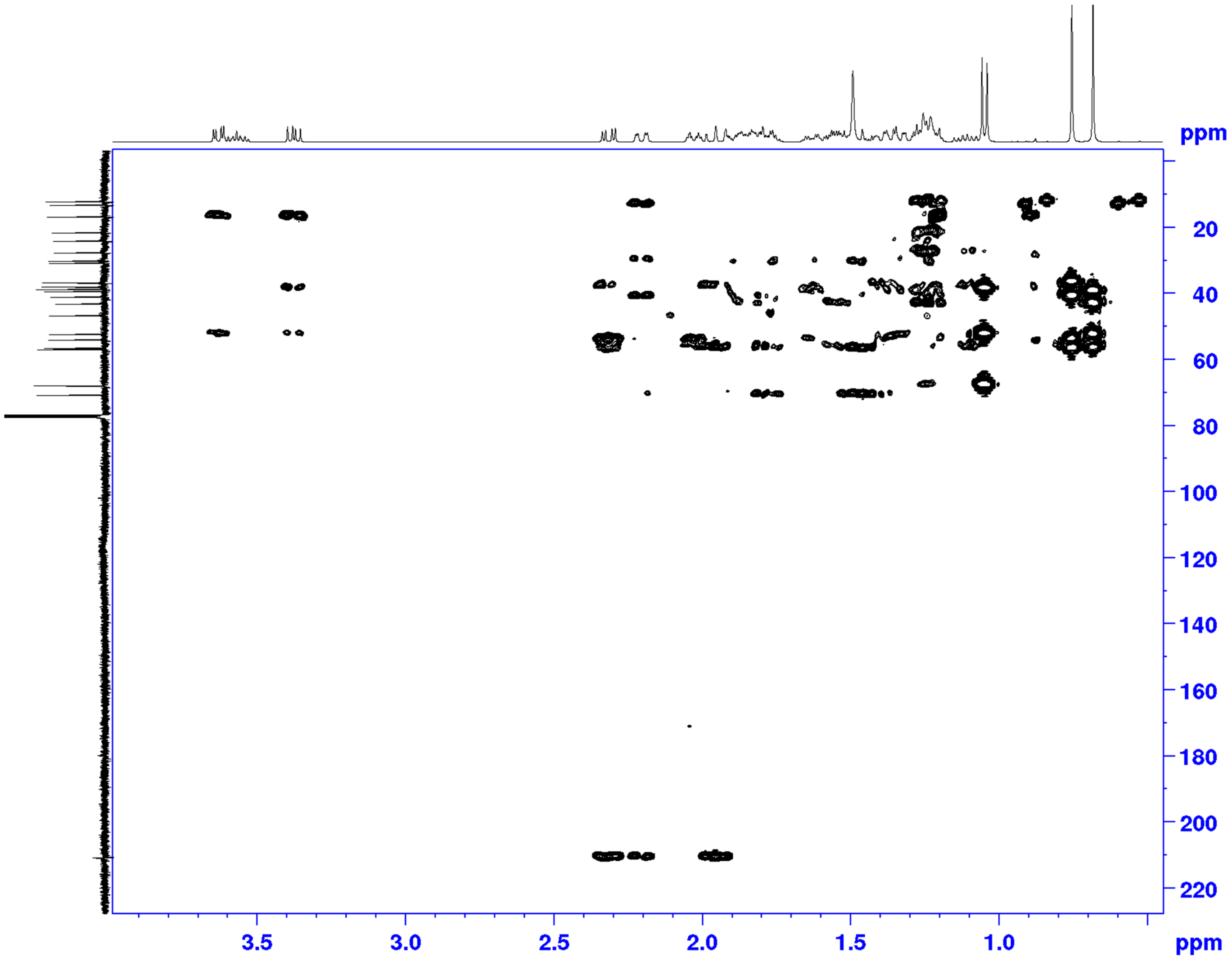


**Figure S48**. 2D HMBC NMR spectrum of 3β-22-dihydroxy-5α-cholan-23,24-dinor-6-oxa (**31**).

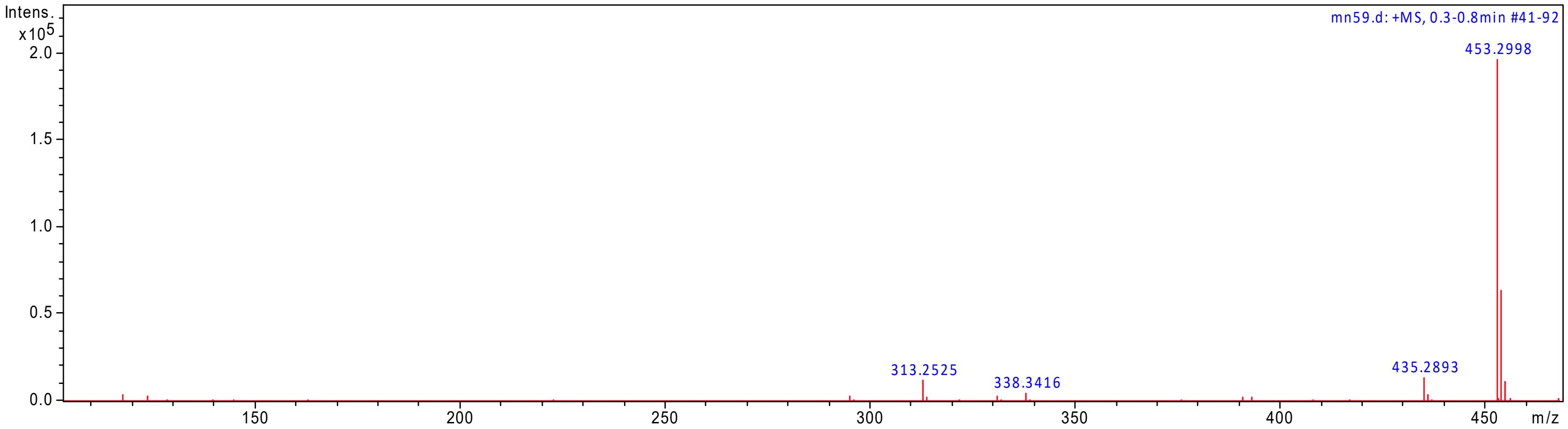


**Figure S49**. HRSM spectrum of 3β-hydroxy-5α-cholan-6-oxo-23,24-dinor-22-benzoate-22-yl (**15**).

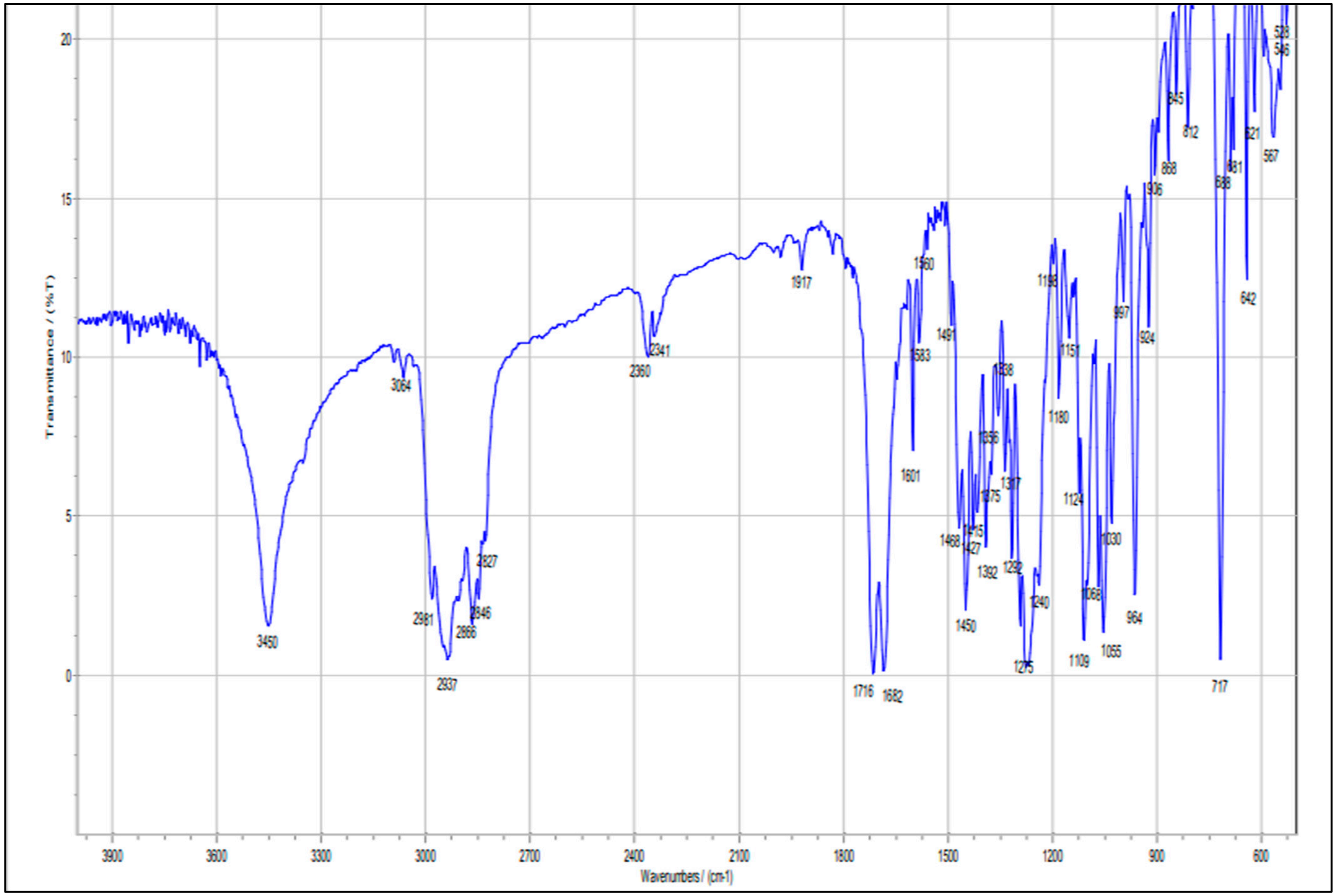


**Figure S50**. IR spectrum of 3β-hydroxy-5α-cholan-6-oxo-23,24-dinor-22-benzoate-22-yl (**15**).

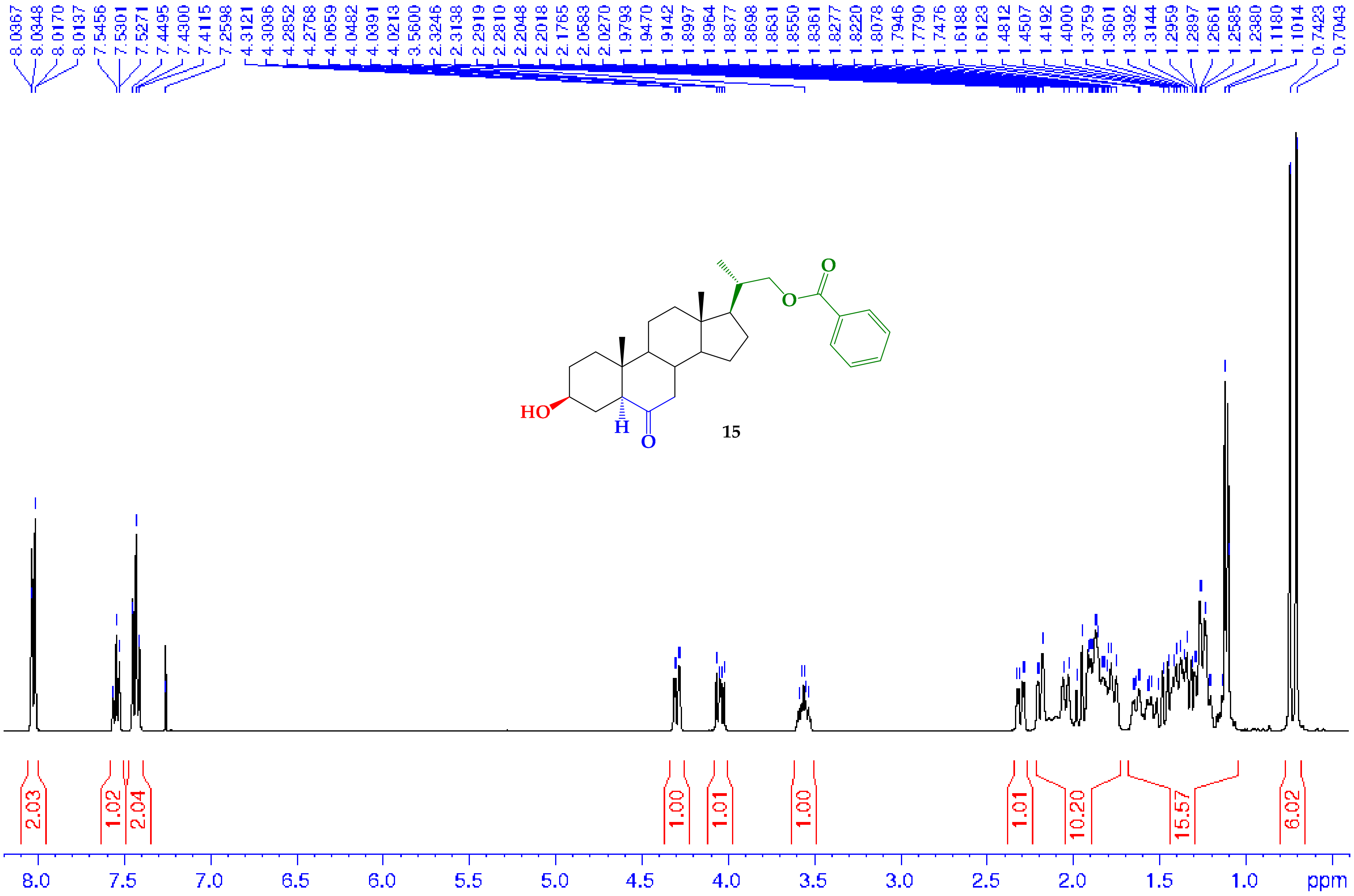

**Figure S51**. $^{1}$H NMR spectrum of 3β-hydroxy-5α-cholan-6-oxo-23,24-dinor-22-benzoate-22-yl (**15**).

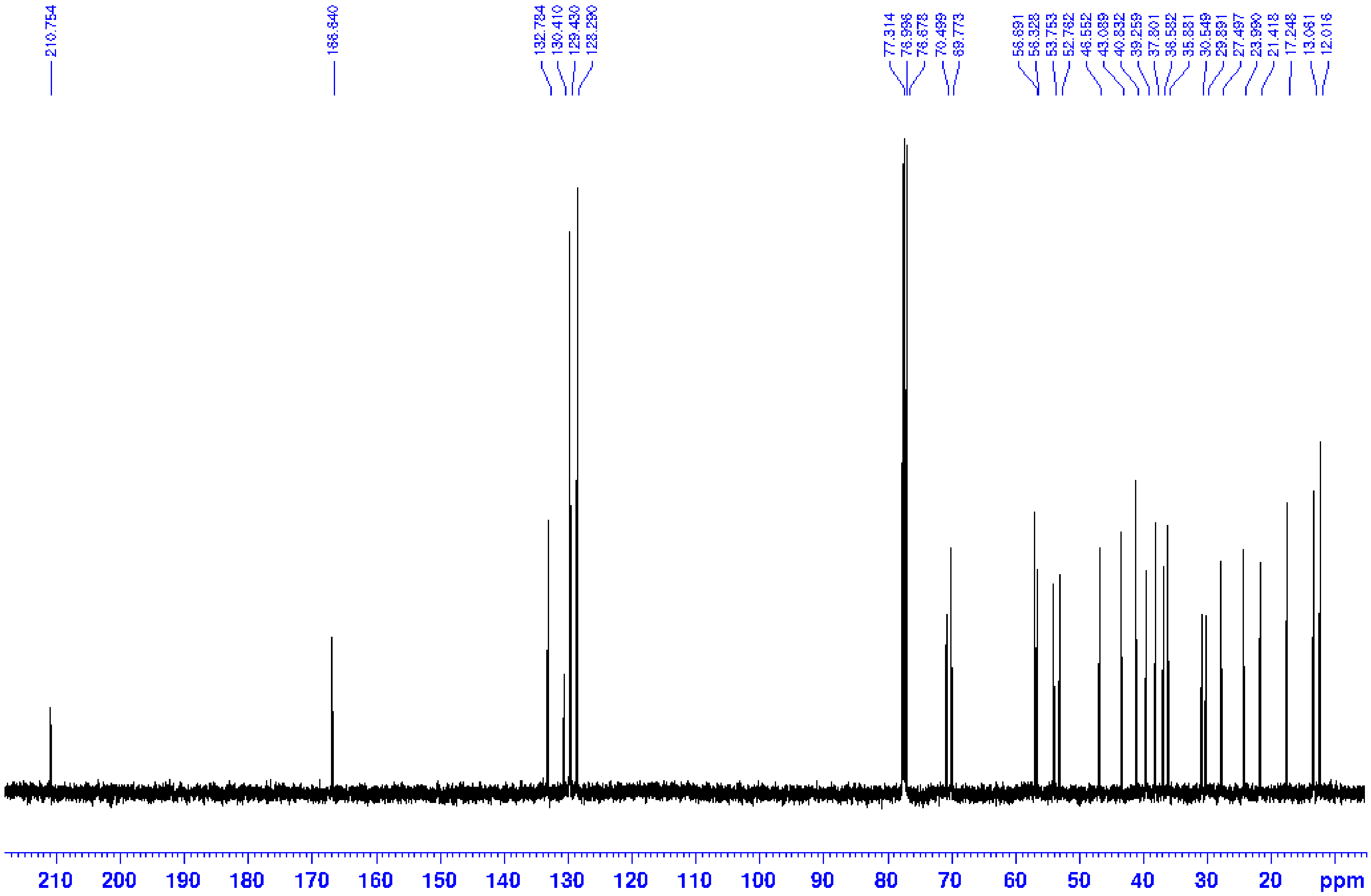

**Figure S52**. $^{13}$C NMR spectrum of 3β-hydroxy-5α-cholan-6-oxo-23,24-dinor-22-benzoate-22-yl (**15**).

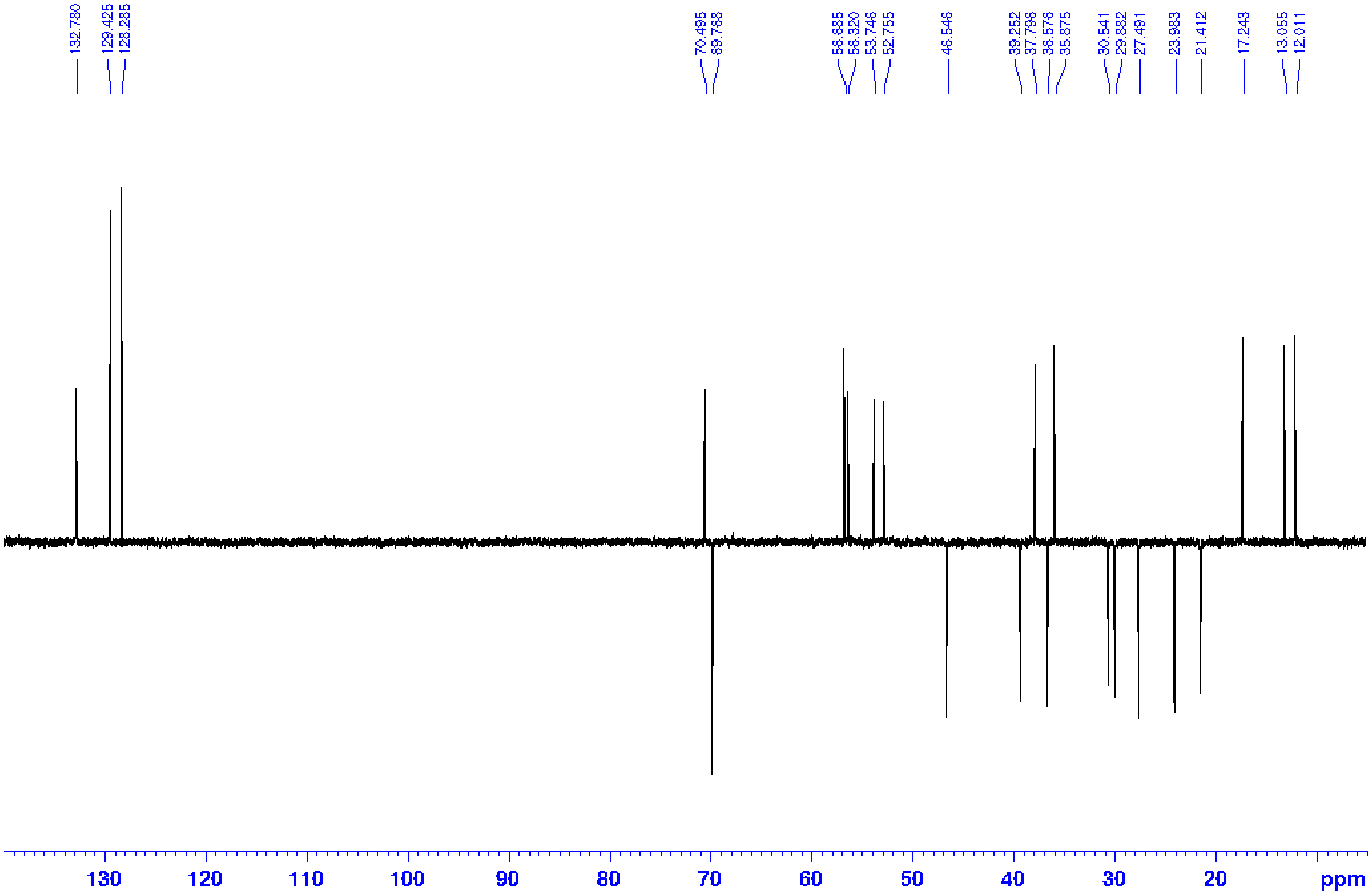


**Figure S53**. $^{13}$C DEPT-135 NMR spectrum of 3β-hydroxy-5α-cholan-6-oxo-23,24-dinor-22-benzoate-22-yl (**15**).

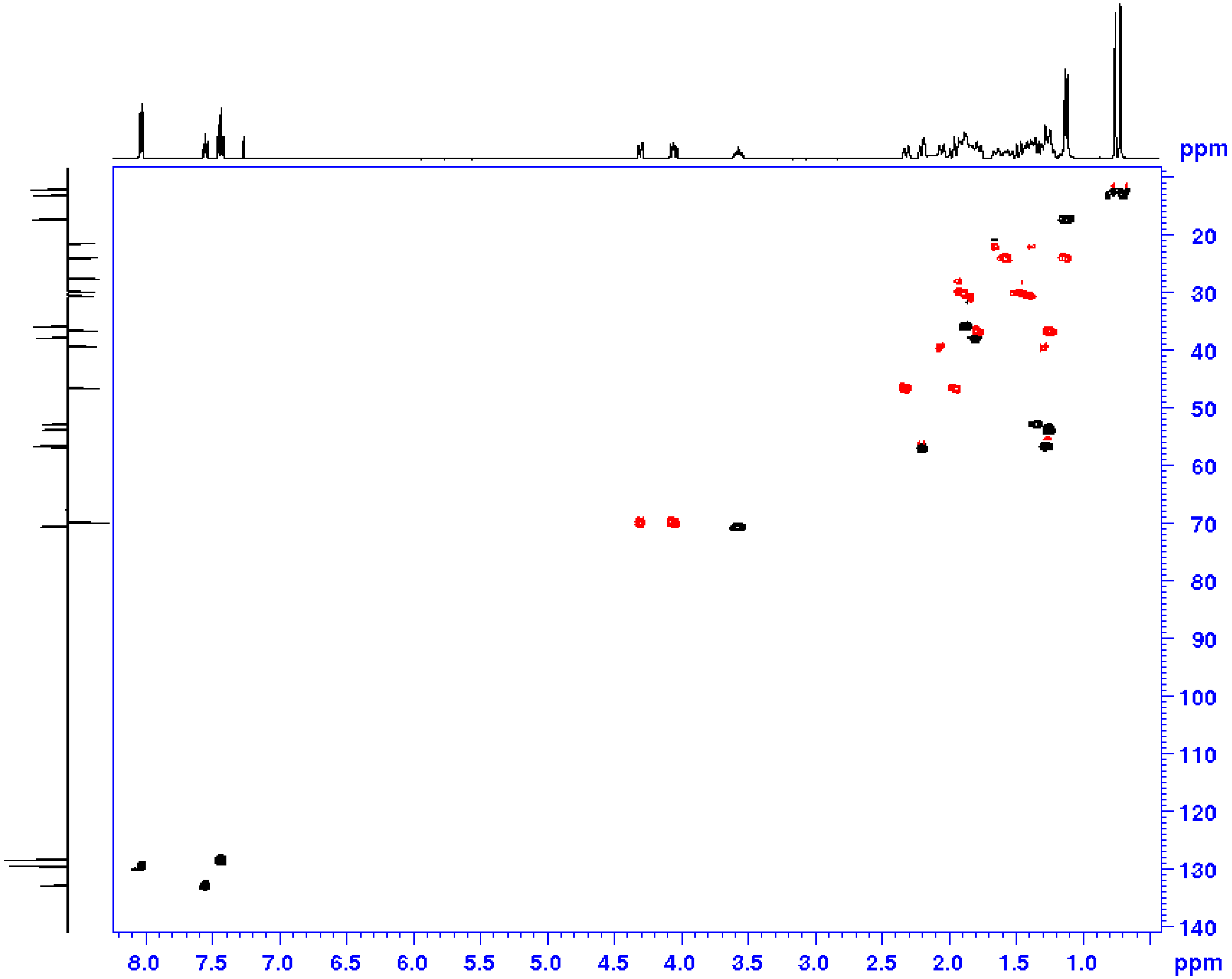


**Figure S54**. 2D HSQC NMR spectrum of 3β-hydroxy-5α-cholan-6-oxo-23,24-dinor-22-benzoate-22-yl (**15**).

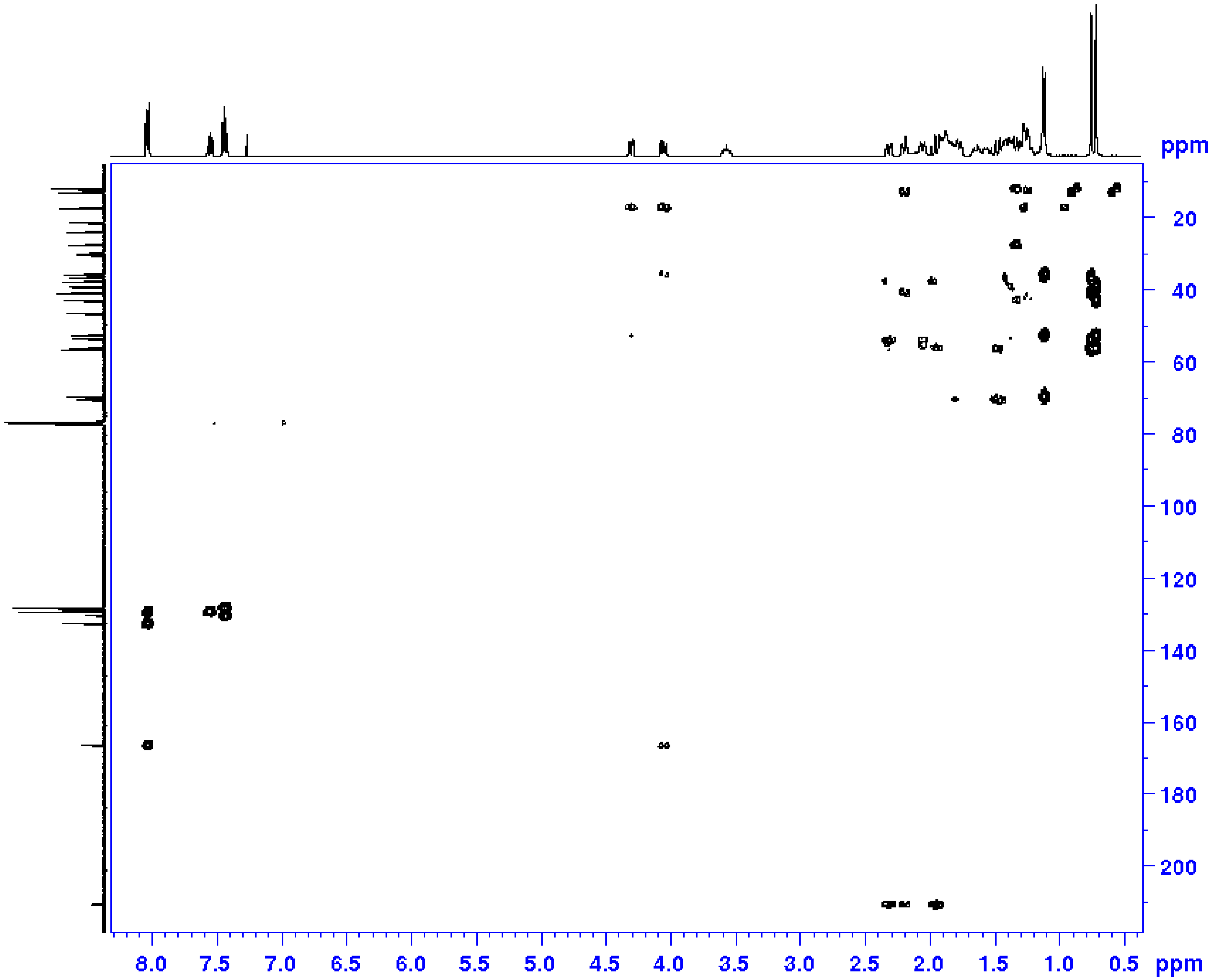


**Figure S55**. 2D HMBC NMR spectrum of 3β-hydroxy-5α-cholan-6-oxo-23,24-dinor-22-benzoate-22-yl (**15**).

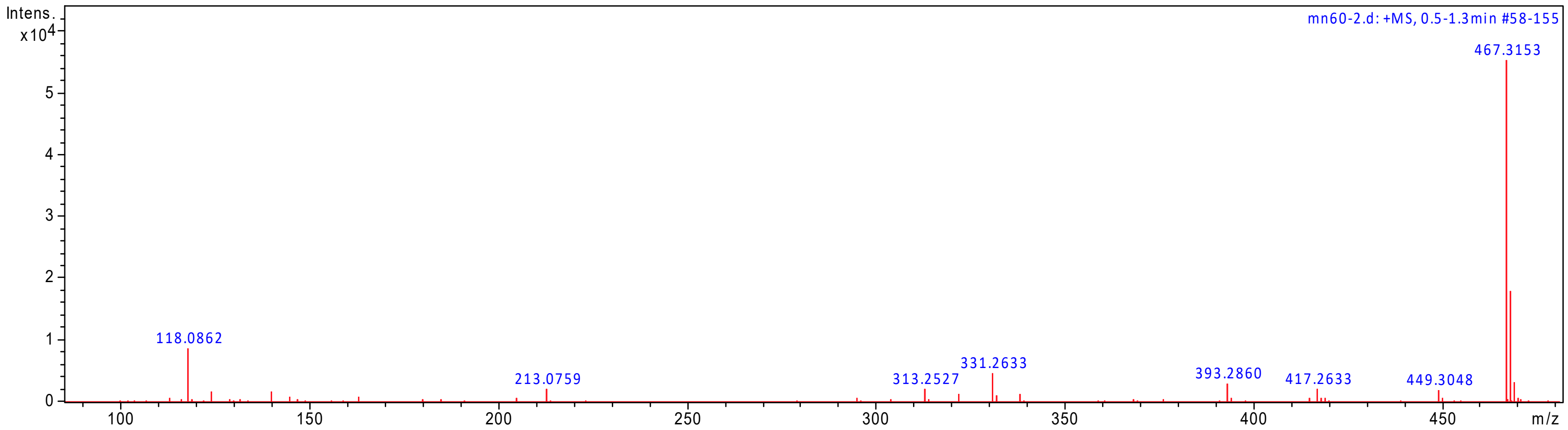


**Figure S56**. HRSM spectrum of 3β-hydroxy-5α-cholan-6-oxo-23,24-dinor-22-(4-methyl)benzoate-22-yl (**16**).

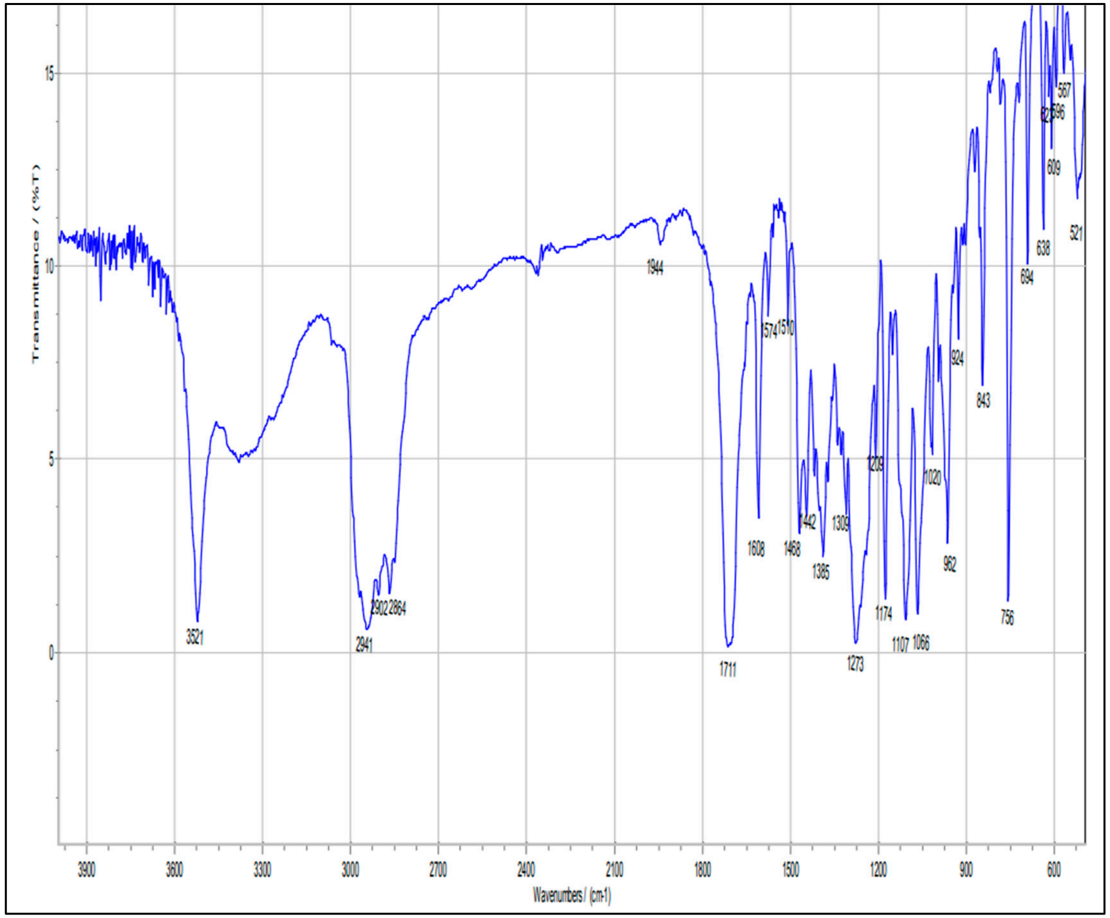


**Figure S57**. IR spectrum of 3β-hydroxy-5α-cholan-6-oxo-23,24-dinor-22-(4-methyl)benzoate-22-yl (**16**).

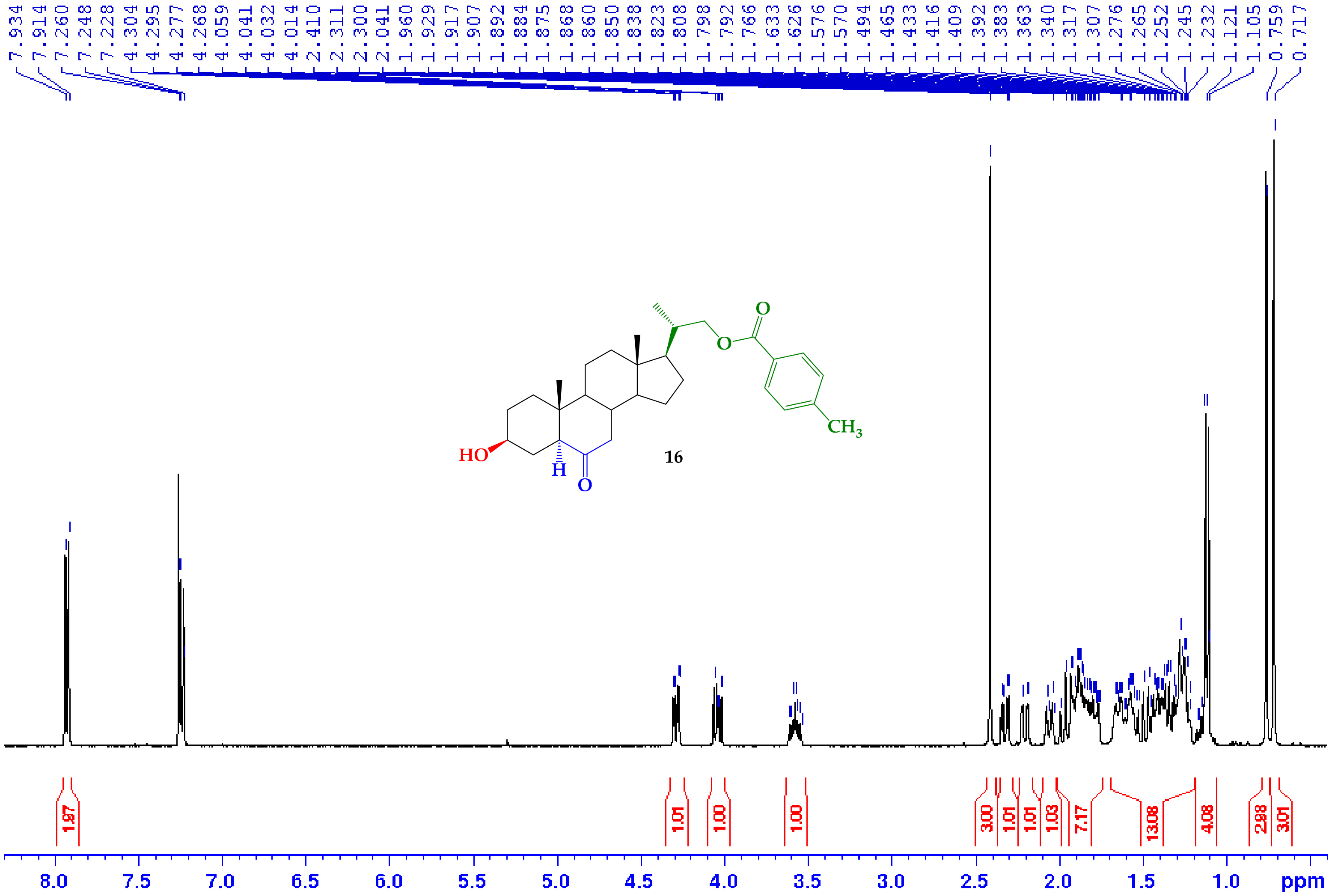

**Figure S58**. $^1H$ NMR spectrum of 3β-hydroxy-5α-cholan-6-oxo-23,24-dinor-22-(4-methyl)benzoate-22-yl (**16**).

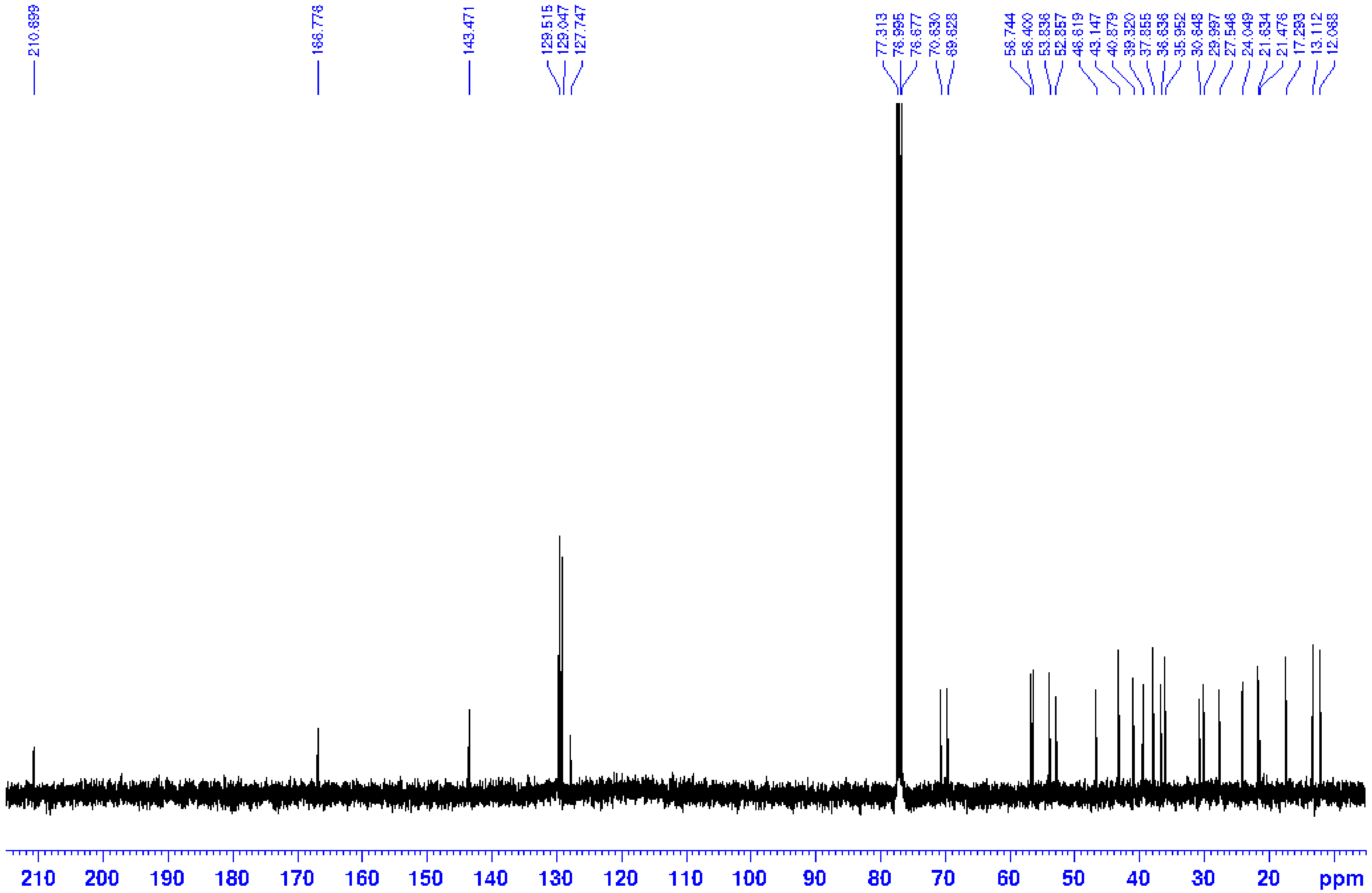


**Figure S59**. $^{13}C$ NMR spectrum of 3β-hydroxy-5α-cholan-6-oxo-23,24-dinor-22-(4-methyl)benzoate-22-yl (**16**).

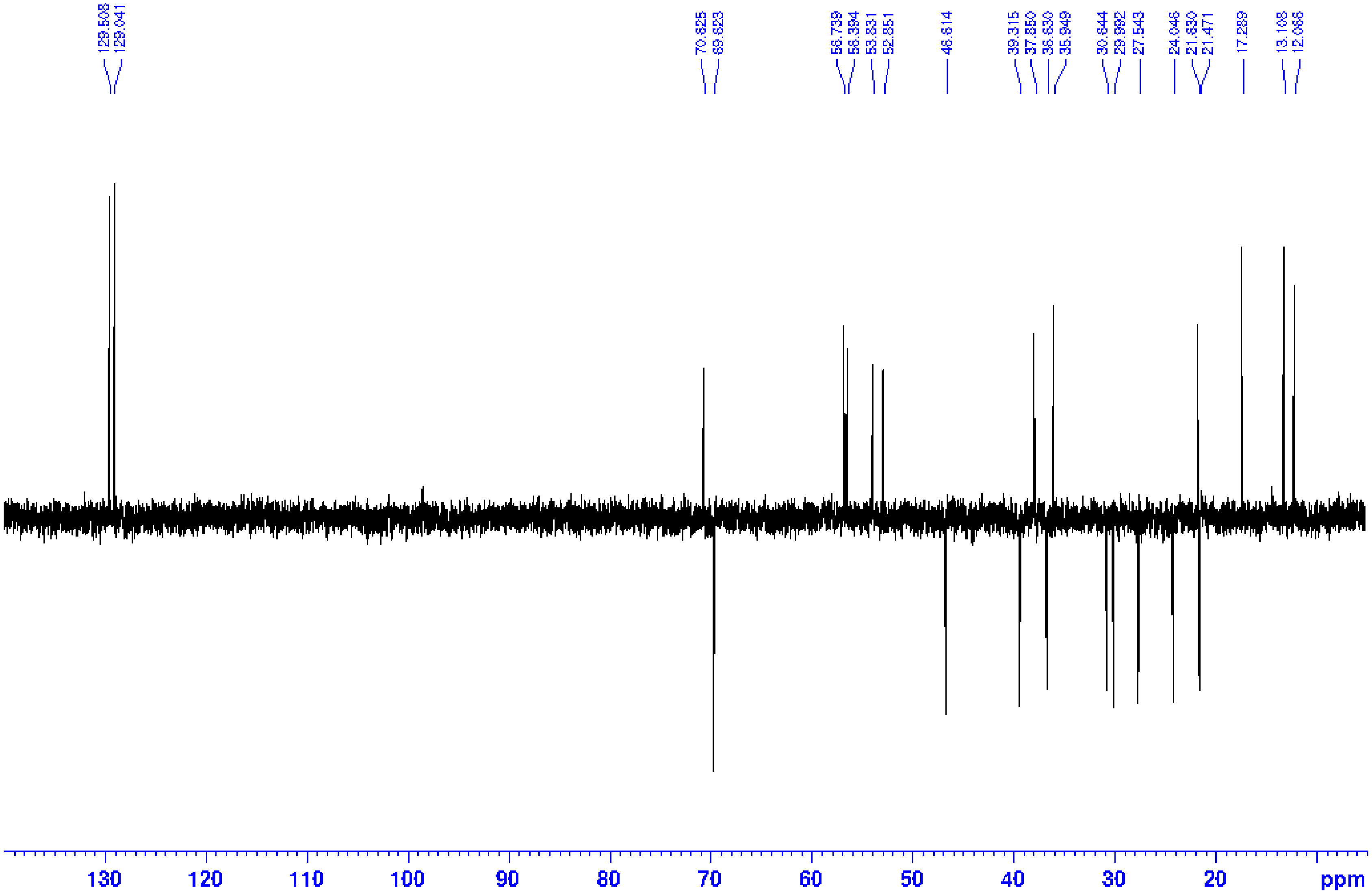


**Figure S60**. $^{13}C$ DEPT-135 NMR spectrum of 3β-hydroxy-5α-cholan-6-oxo-23,24-dinor-22-(4-methyl)benzoate-22-yl (**16**).

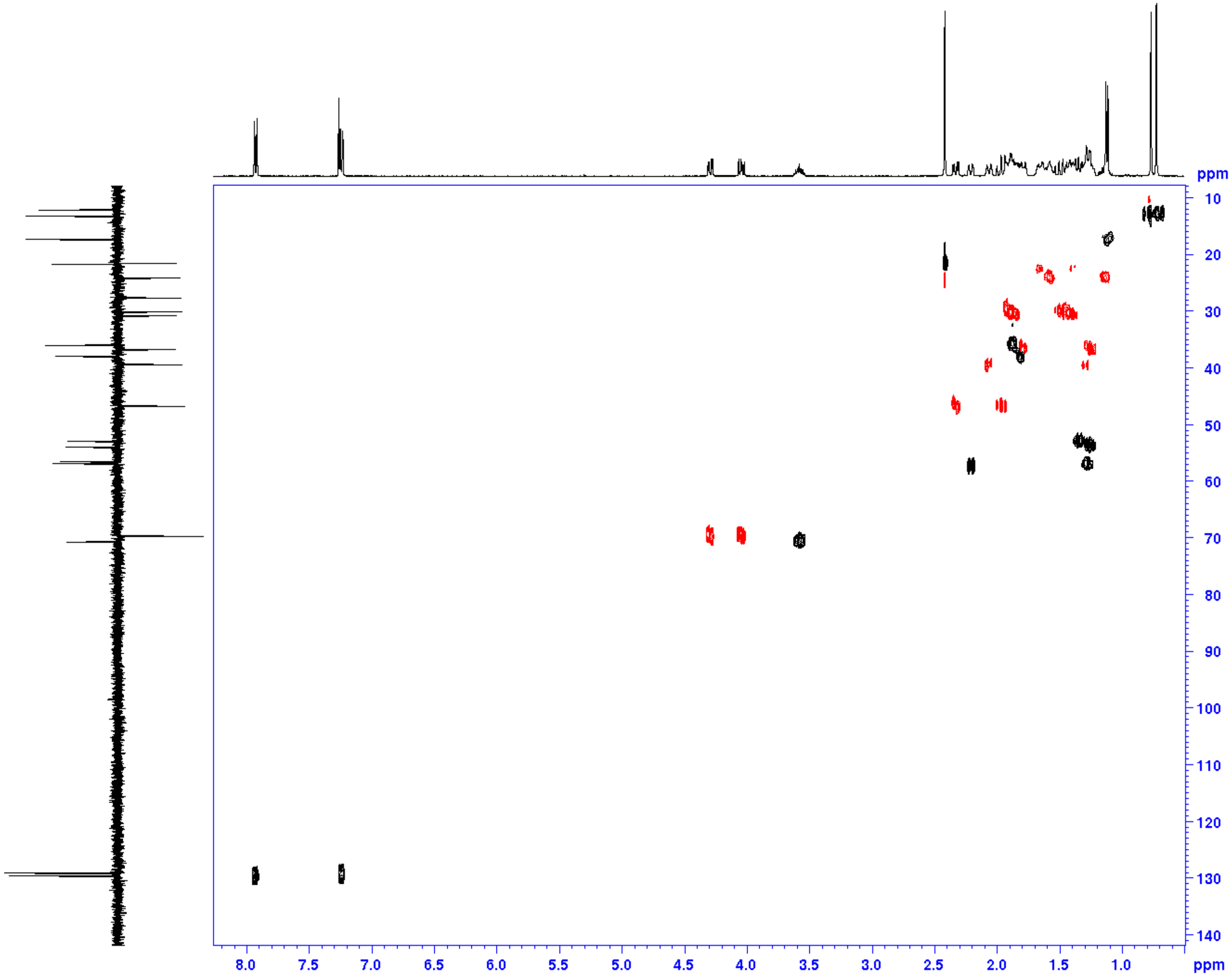


**Figure S61**. 2D HSQC NMR spectrum of 3β-hydroxy-5α-cholan-6-oxo-23,24-dinor-22-(4-methyl)benzoate-22-yl (**16**).

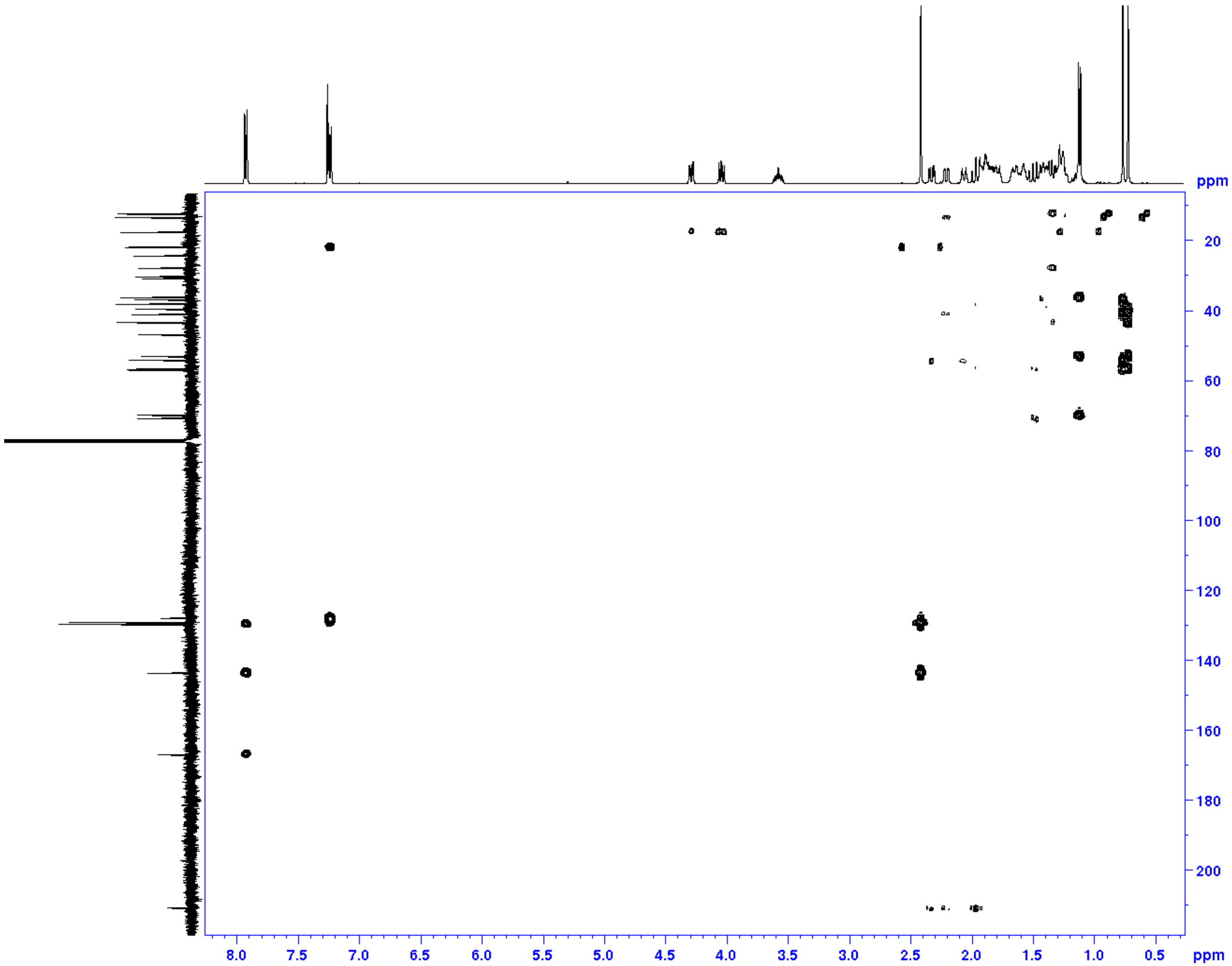


**Figure S62**. 2D HMBC NMR spectrum of 3β-hydroxy-5α-cholan-6-oxo-23,24-dinor-22-(4-methyl)benzoate-22-yl (**16**).

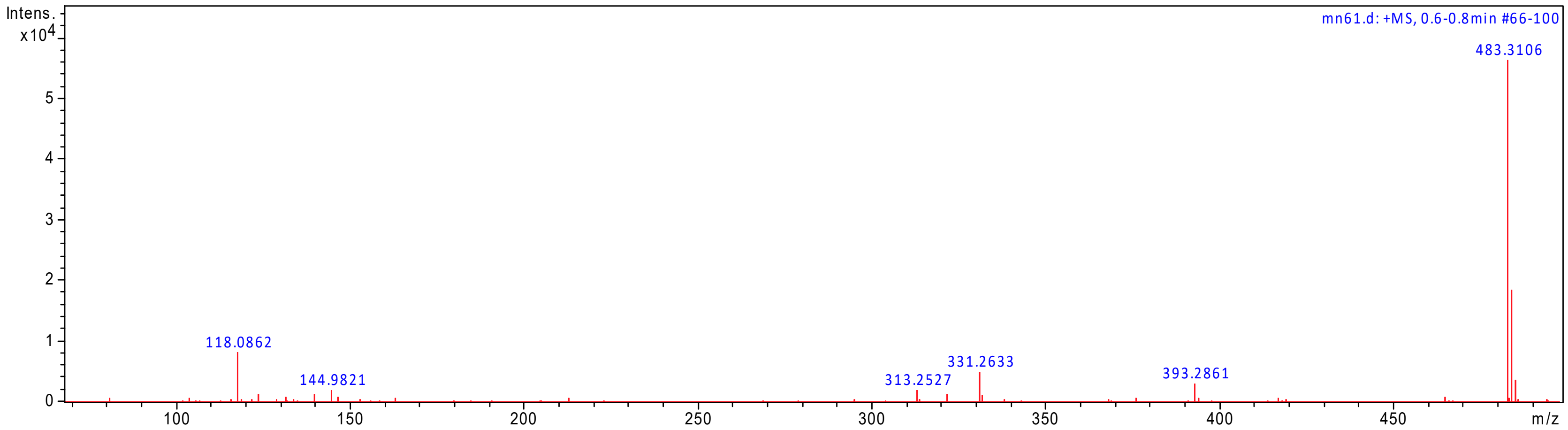


**Figure S63**. HRSM spectrum of 3β-hydroxy-5α-cholan-6-oxo-23,24-dinor-22-(4-methoxy)benzoate-22-yl (**17**).

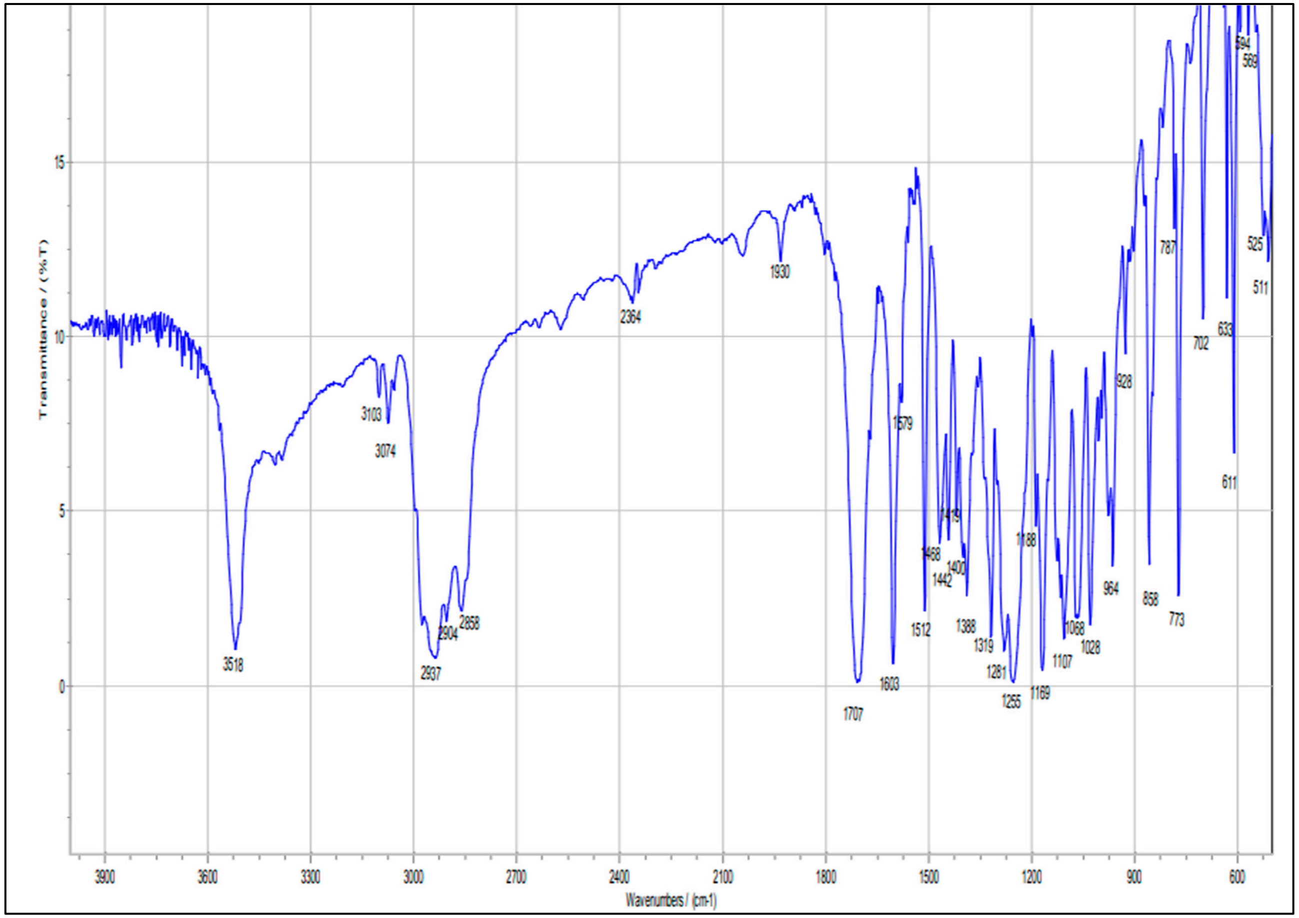


**Figure S64**. IR spectrum of 3β-hydroxy-5α-cholan-6-oxo-23,24-dinor-22-(4-methoxy)benzoate-22-yl (**17**).

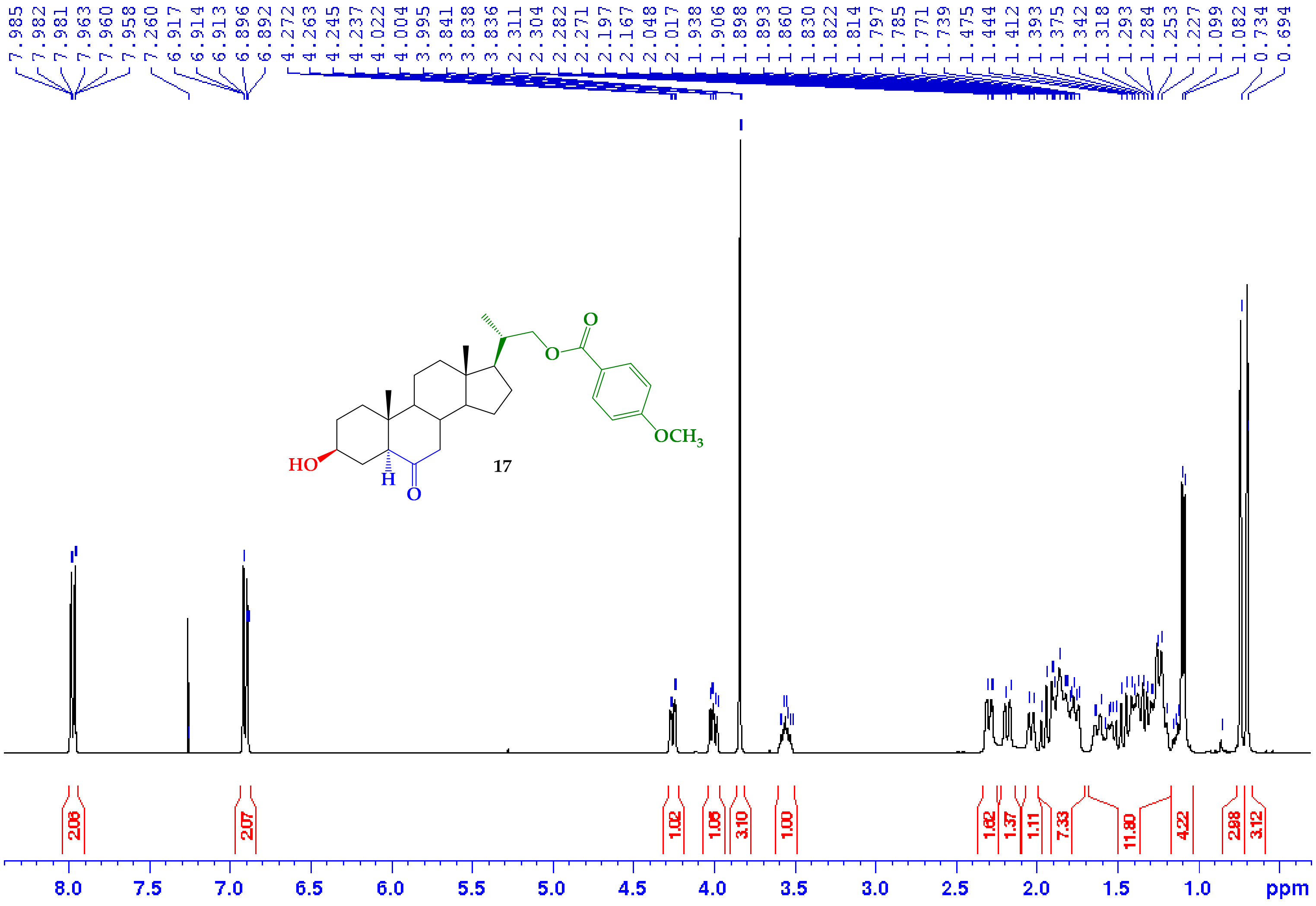

**Figure S65**. $^1H$ NMR spectrum of 3β-hydroxy-5α-cholan-6-oxo-23,24-dinor-22-(4-methoxy)benzoate-22-yl (**17**).

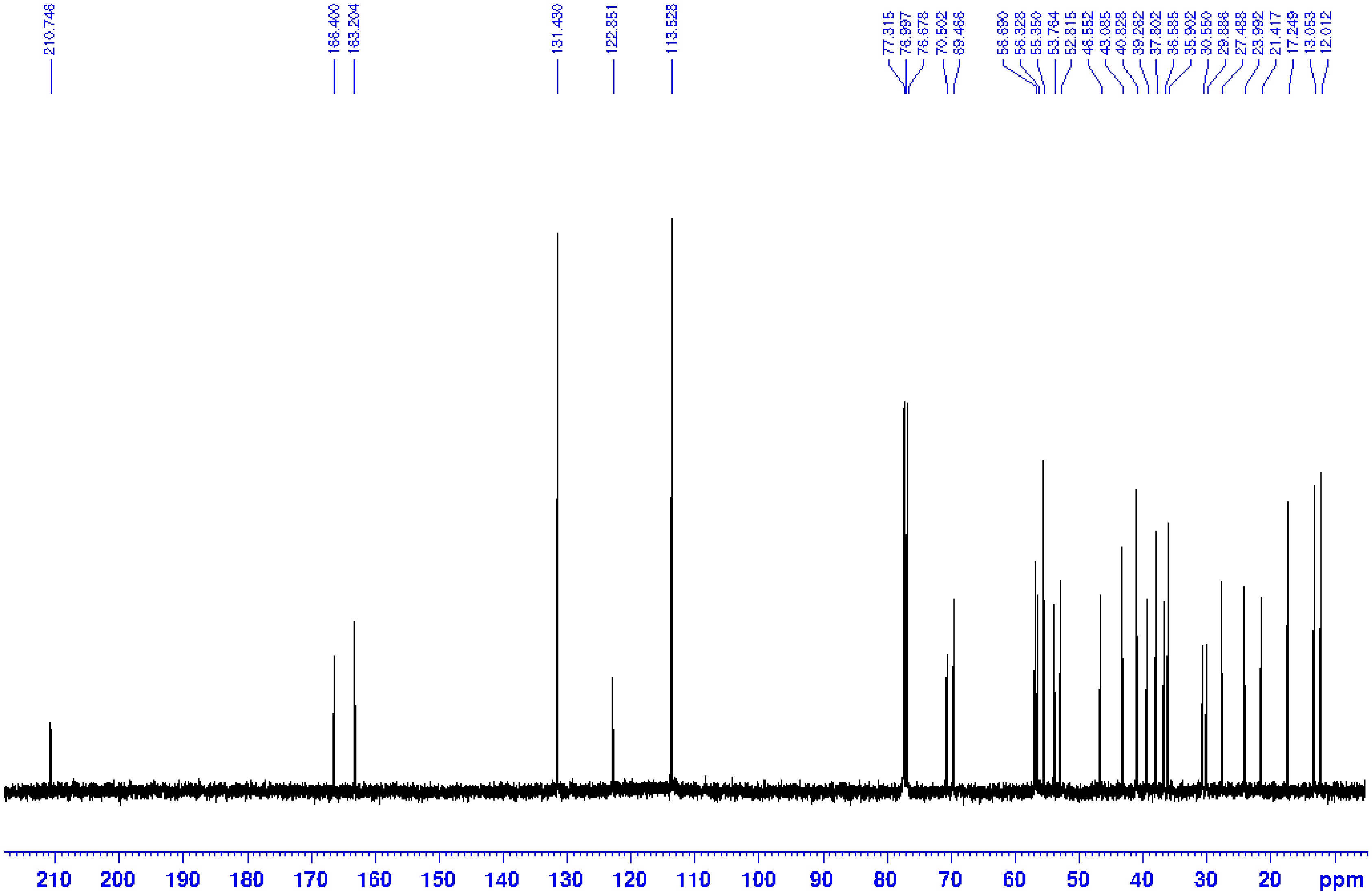


**Figure S66**. $^{13}C$ NMR spectrum of 3β-hydroxy-5α-cholan-6-oxo-23,24-dinor-22-(4-methoxy)benzoate-22-yl (**17**).

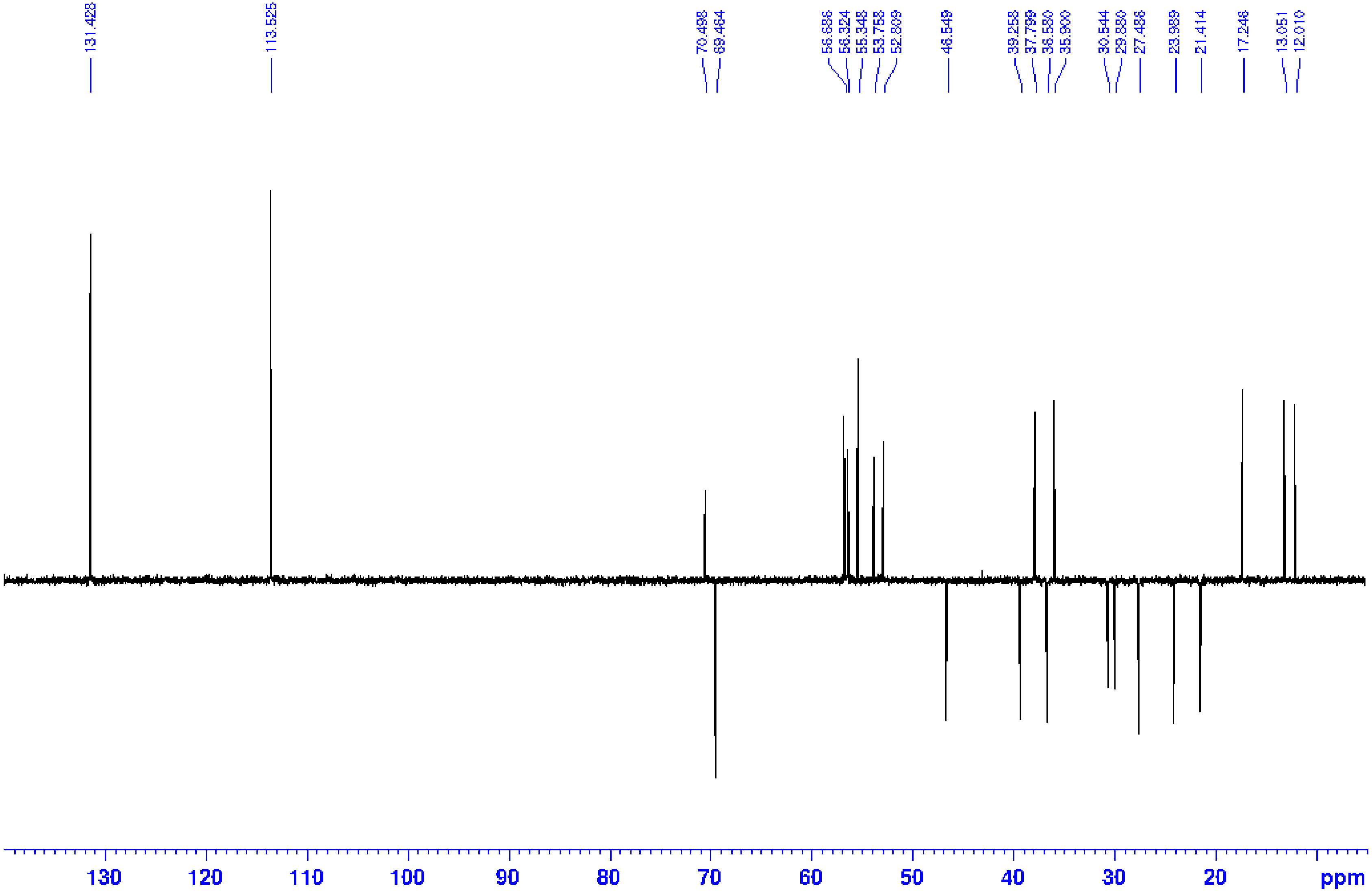


**Figure S67**. $^{13}$C DEPT-135 NMR spectrum of 3β-hydroxy-5α-cholan-6-oxo-23,24-dinor-22-(4-methoxy)benzoate-22-yl (**17**).

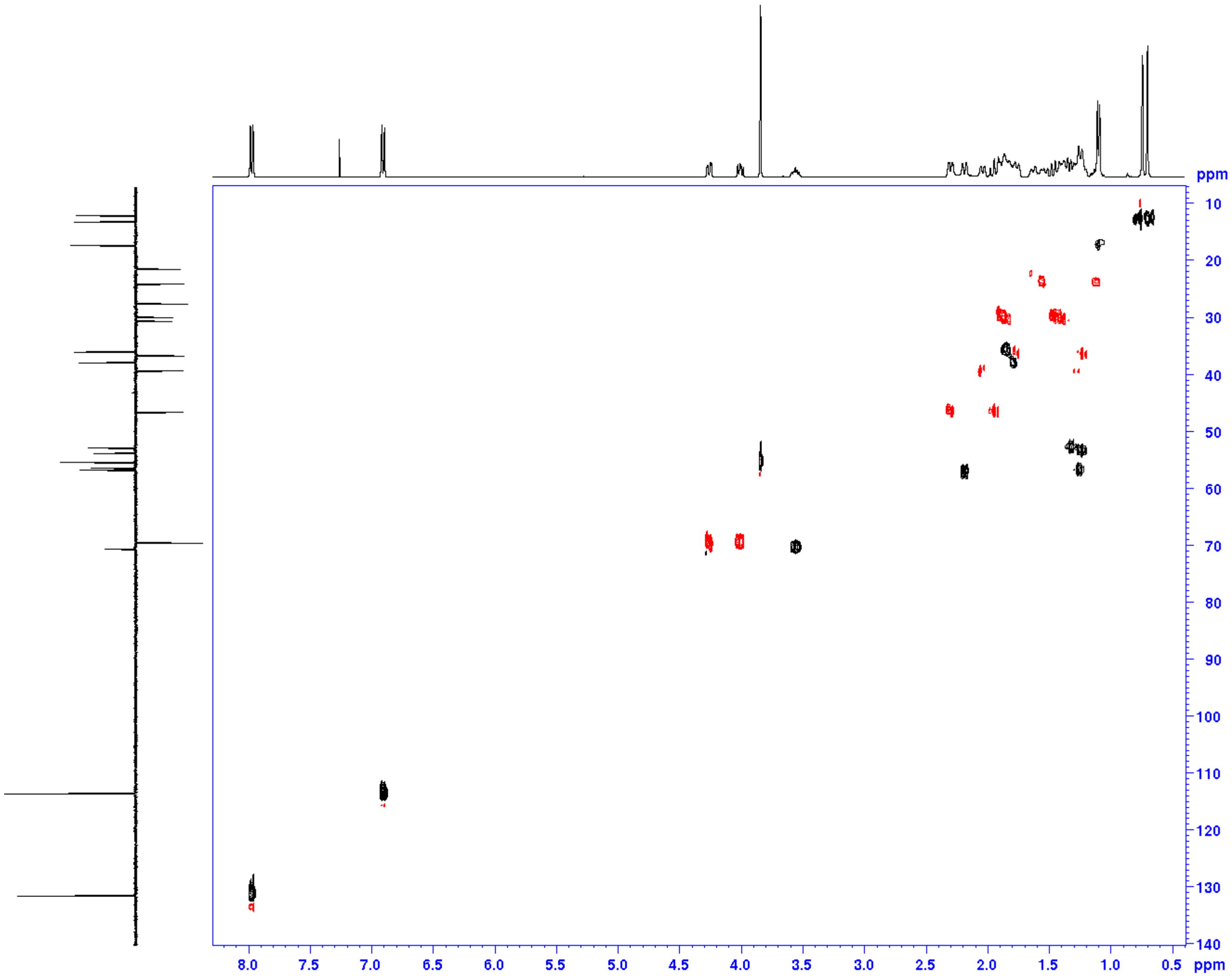


**Figure S68**. 2D HSQC NMR spectrum of 3β-hydroxy-5α-cholan-6-oxo-23,24-dinor-22-(4-methoxy)benzoate-22-yl (**17**).

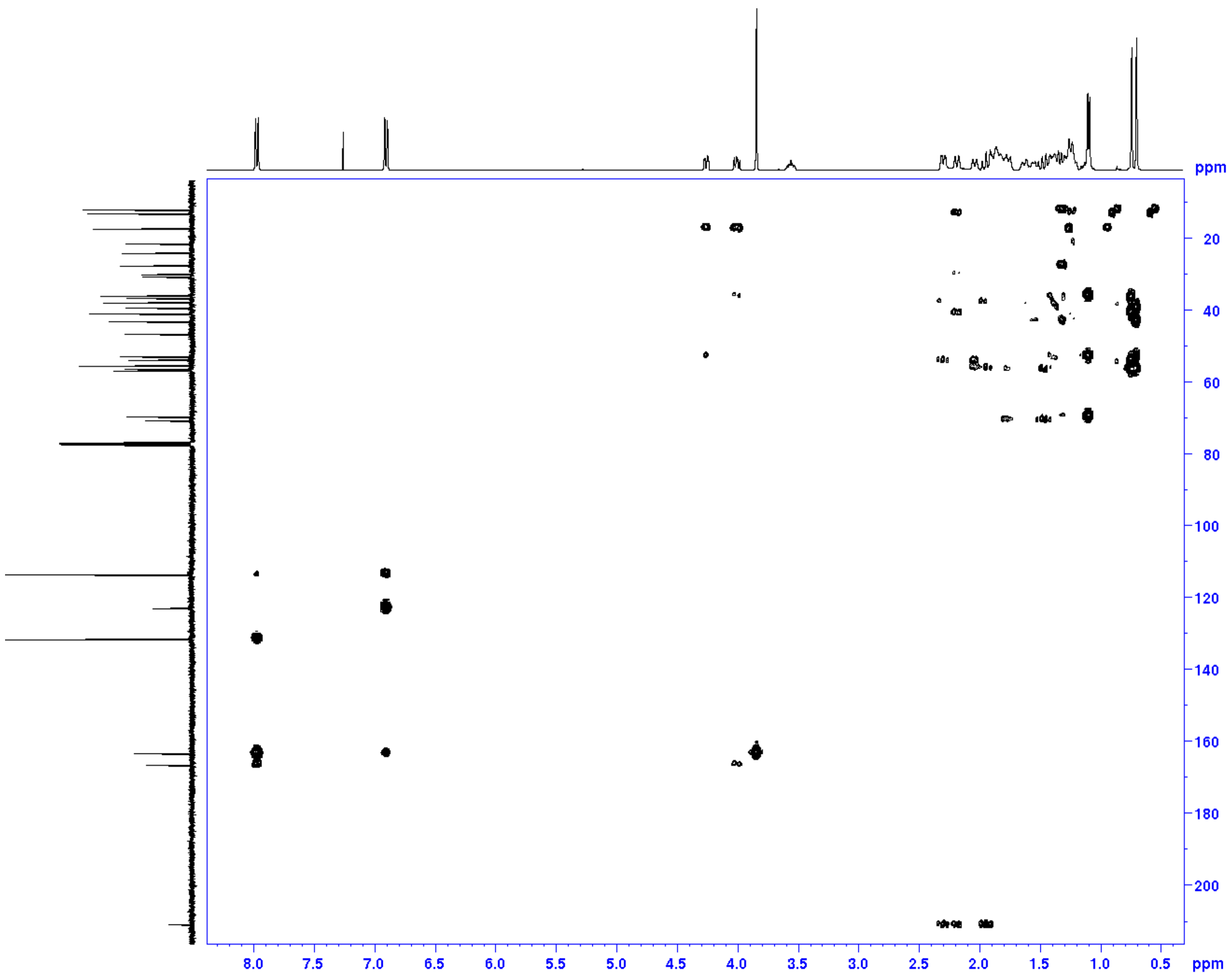


**Figure S69**. 2D HMBC NMR spectrum of 3β-hydroxy-5α-cholan-6-oxo-23,24-dinor-22-(4-methoxy)benzoate-22-yl (**17**).

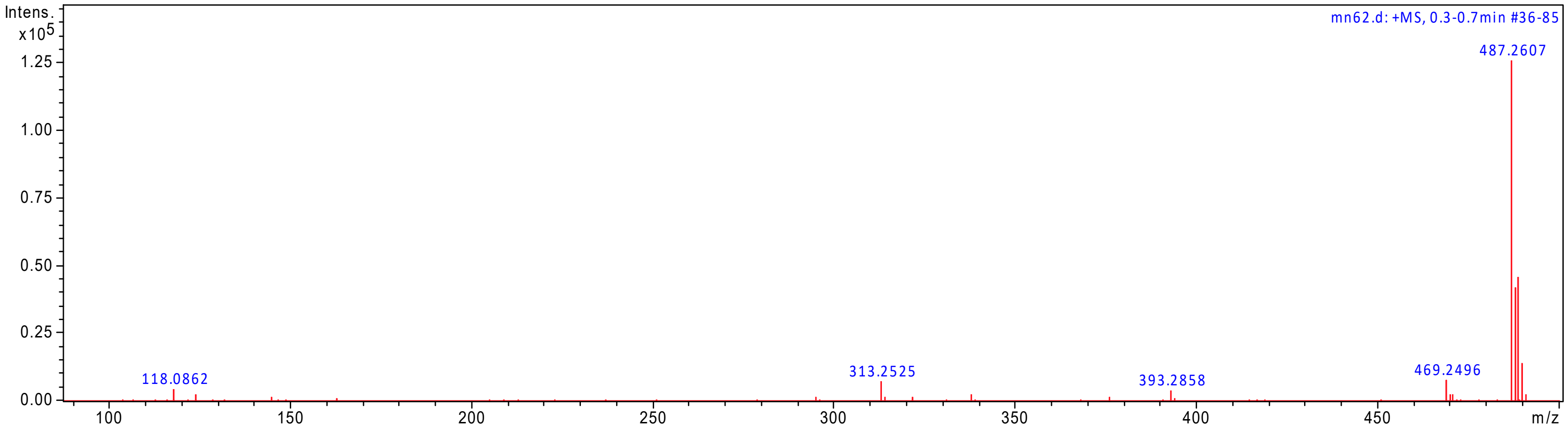


**Figure S70**. HRSM spectrum of 3β-hydroxy-5α-cholan-6-oxo-23,24-dinor-22-(4-chloro)benzoate-22-yl (**18**).

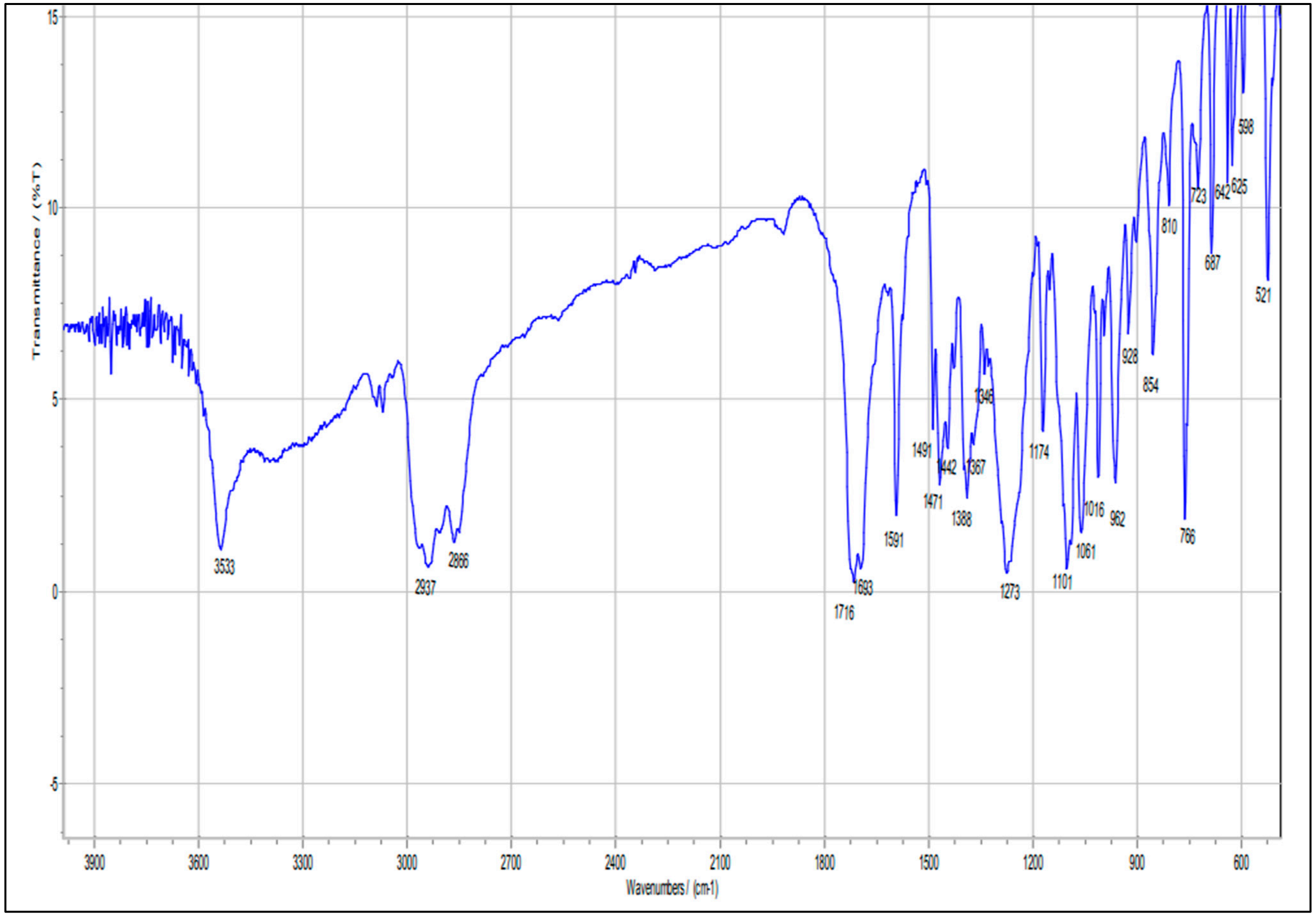


**Figure S71**. IR spectrum of 3β-hydroxy-5α-cholan-6-oxo-23,24-dinor-22-(4-chloro)benzoate-22-yl (**18**).

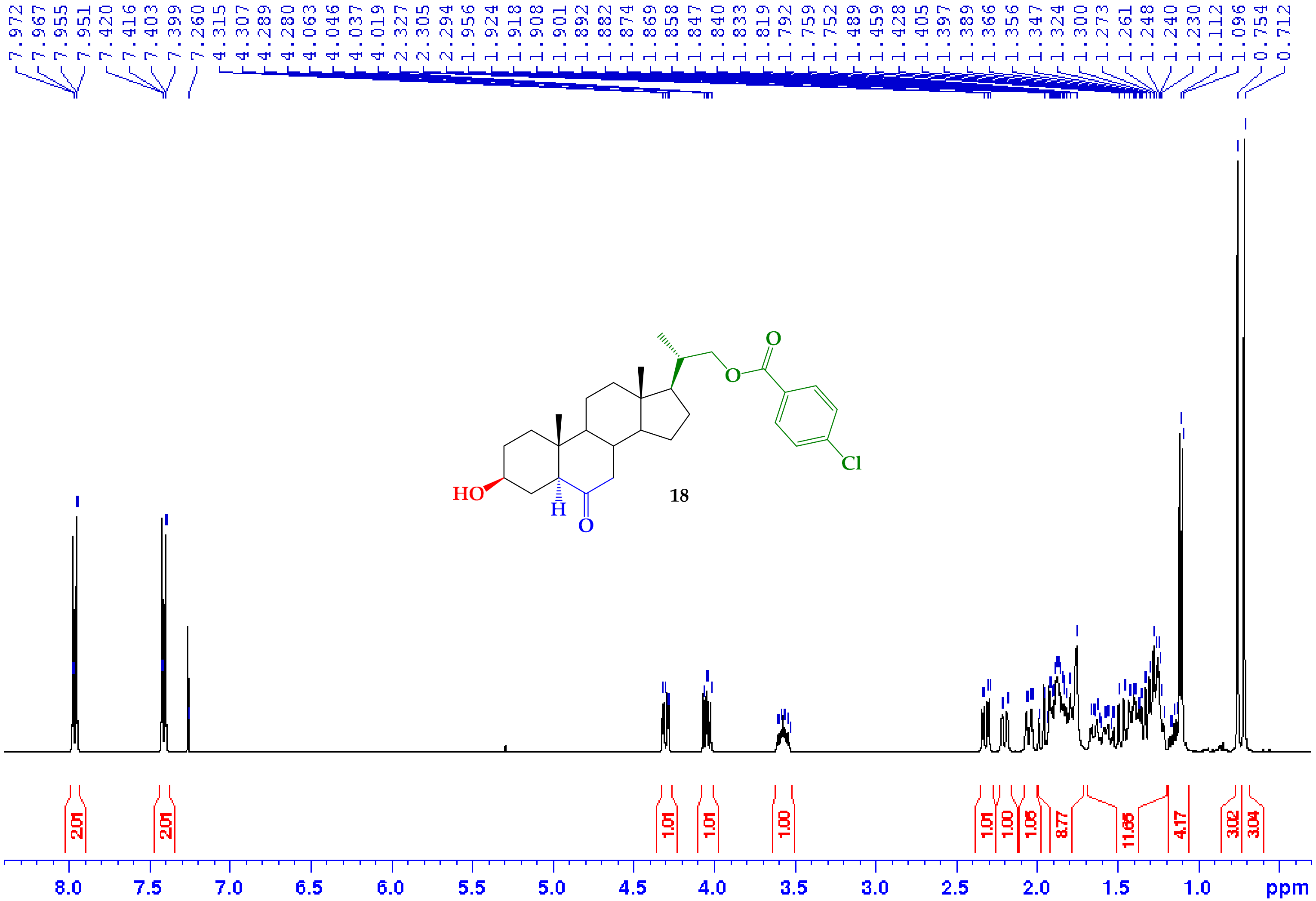

**Figure S72**. $^{1}$H NMR spectrum of 3β-hydroxy-5α-cholan-6-oxo-23,24-dinor-22-(4-chloro)benzoate-22-yl (**18**).

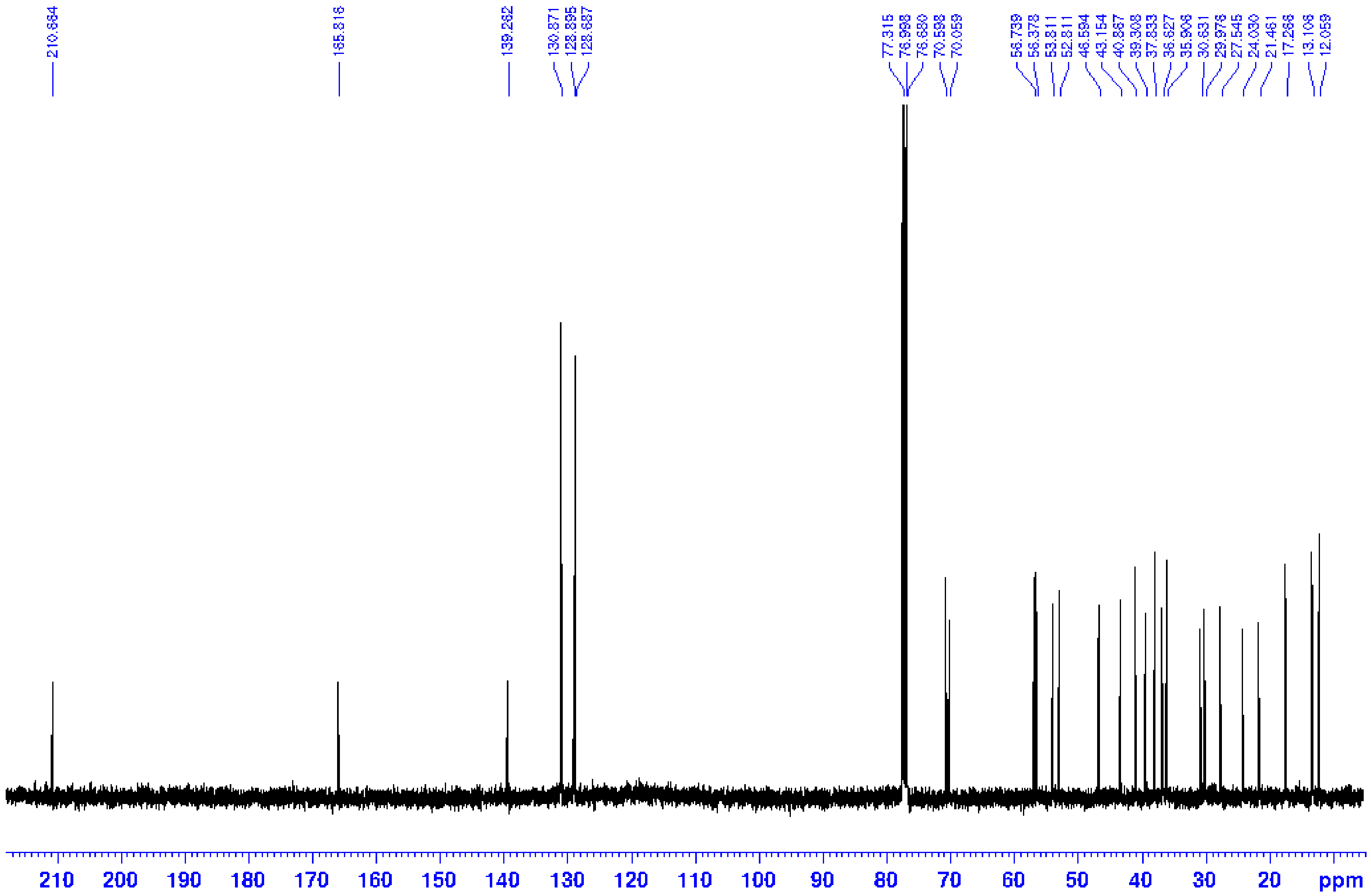


**Figure S73**. $^{13}C$ NMR spectrum of 3β-hydroxy-5α-cholan-6-oxo-23,24-dinor-22-(4-chloro)benzoate-22-yl (**18**).

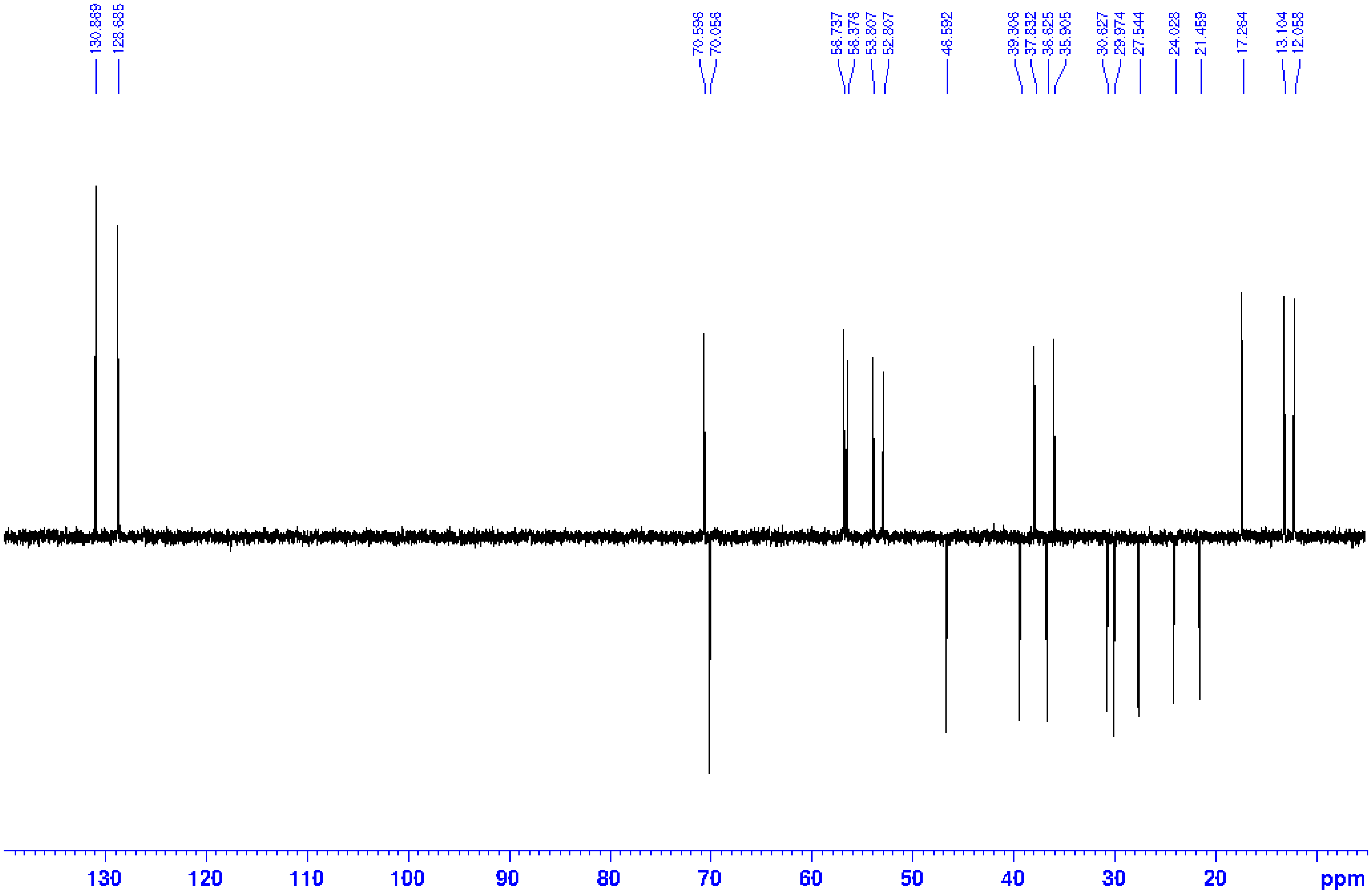


**Figure S74**. $^{13}$C DEPT-135 NMR spectrum of 3β-hydroxy-5α-cholan-6-oxo-23,24-dinor-22-(4-chloro)benzoate-22-yl (**18**).

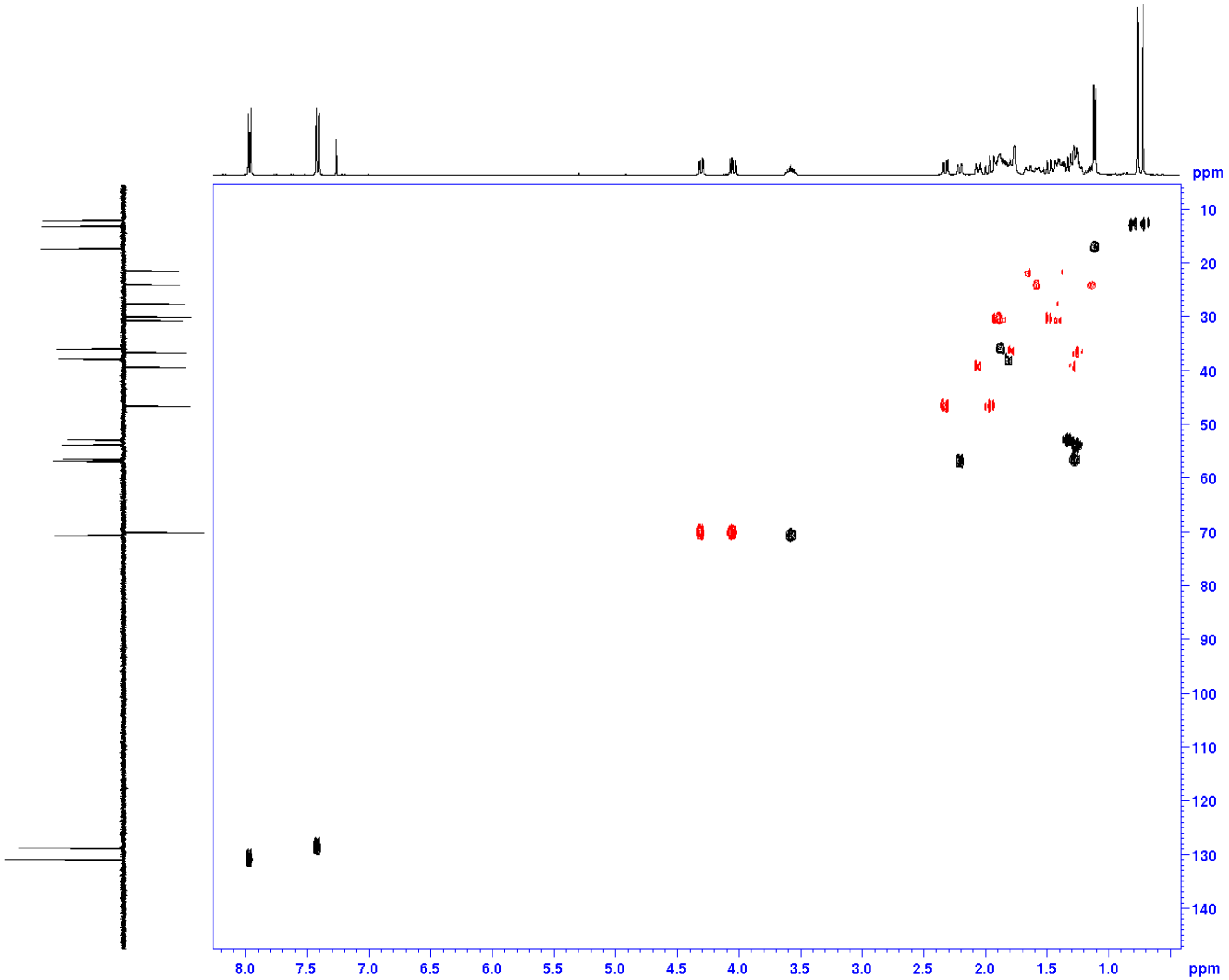


**Figure S75**. 2D HSQC NMR spectrum of 3β-hydroxy-5α-cholan-6-oxo-23,24-dinor-22-(4-chloro)benzoate-22-yl (**18**).

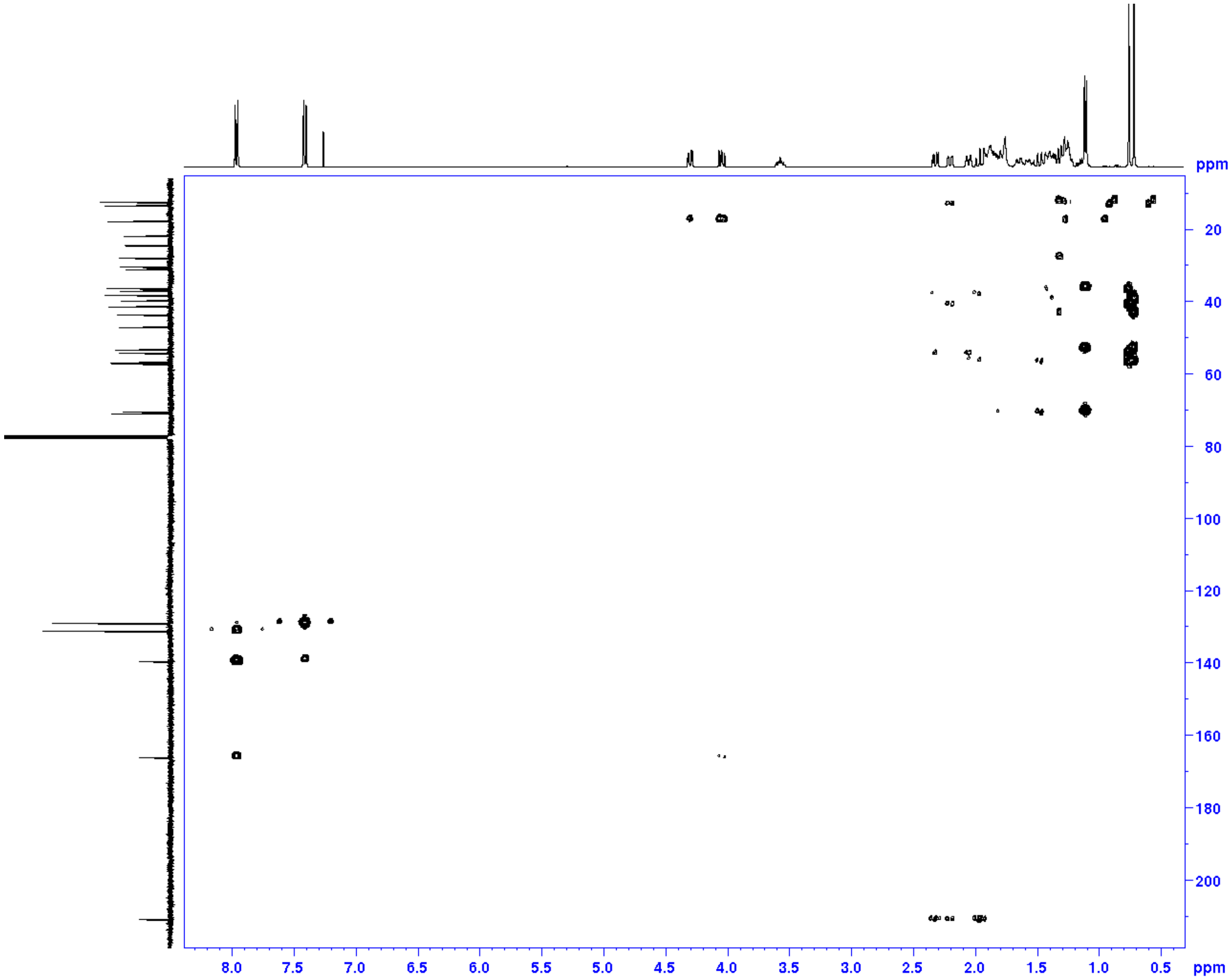


**Figure S76**. 2D HMBC NMR spectrum of 3β-hydroxy-5α-cholan-6-oxo-23,24-dinor-22-(4-chloro)benzoate-22-yl (**18**).

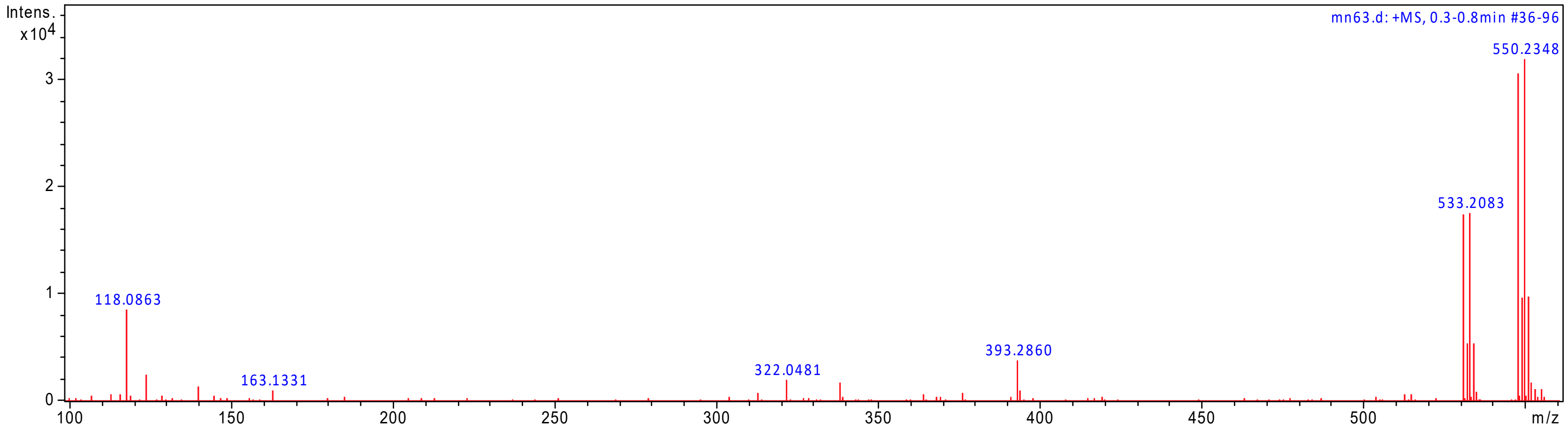


**Figure S77**. HRSM spectrum of 3β-hydroxy-5α-cholan-6-oxo-23,24-dinor-22-(4-bromo)benzoate-22-yl (**19**).

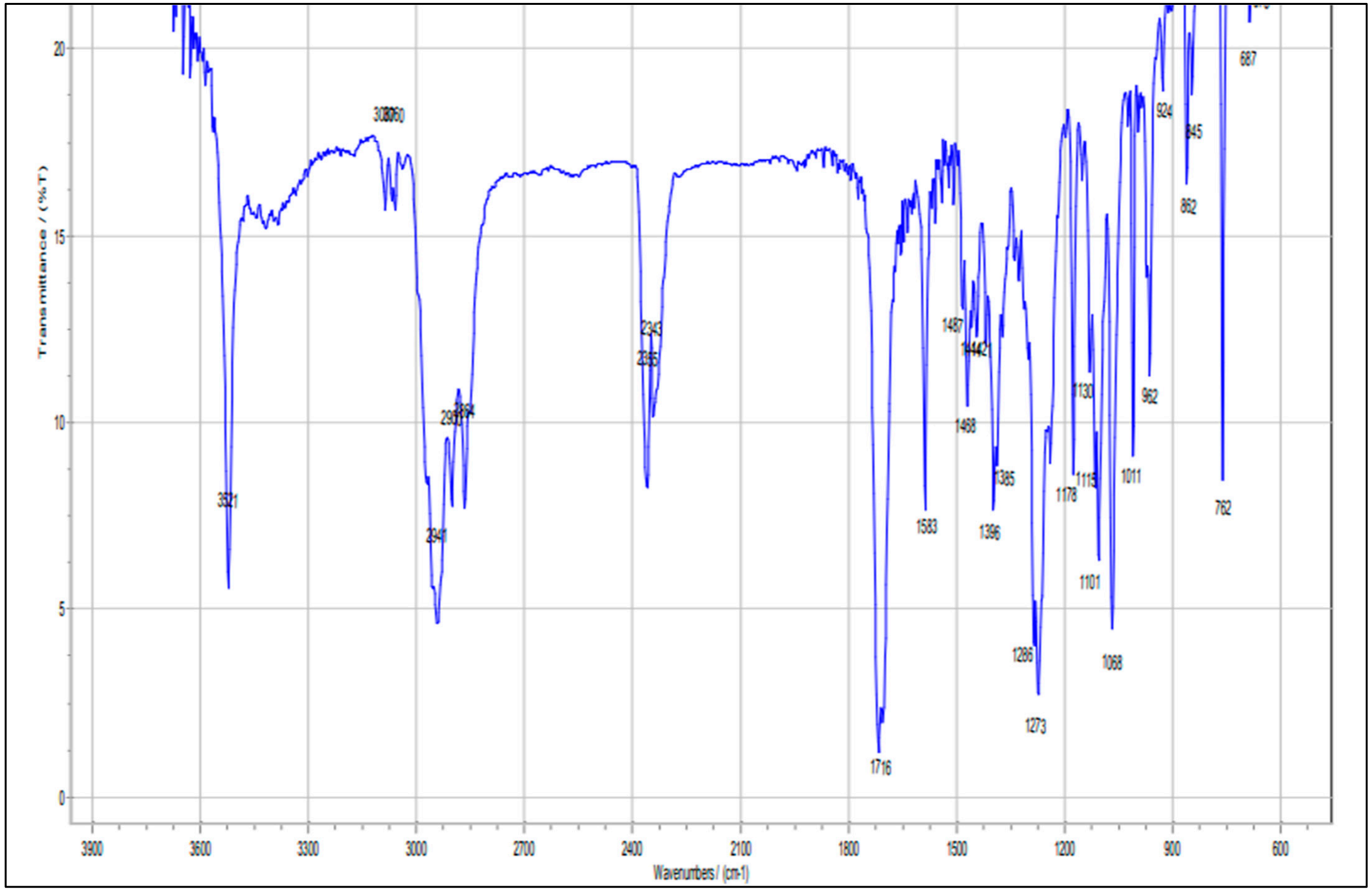

**Figure S78**. IR spectrum of 3β-hydroxy-5α-cholan-6-oxo-23,24-dinor-22-(4-bromo)benzoate-22-yl (**19**).

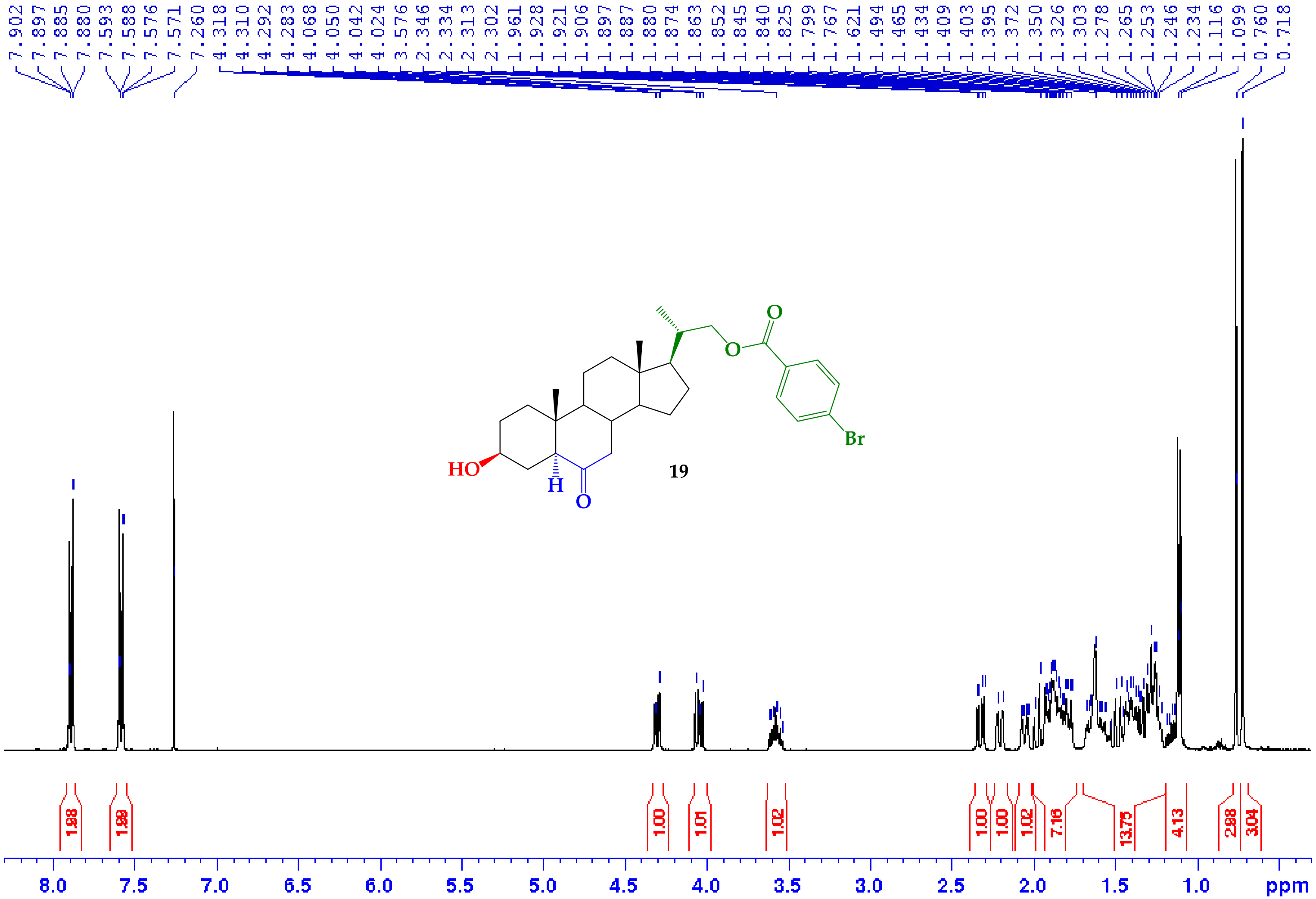

**Figure S79**. $^{1}$H NMR spectrum of 3β-hydroxy-5α-cholan-6-oxo-23,24-dinor-22-(4-bromo)benzoate-22-yl (**19**).

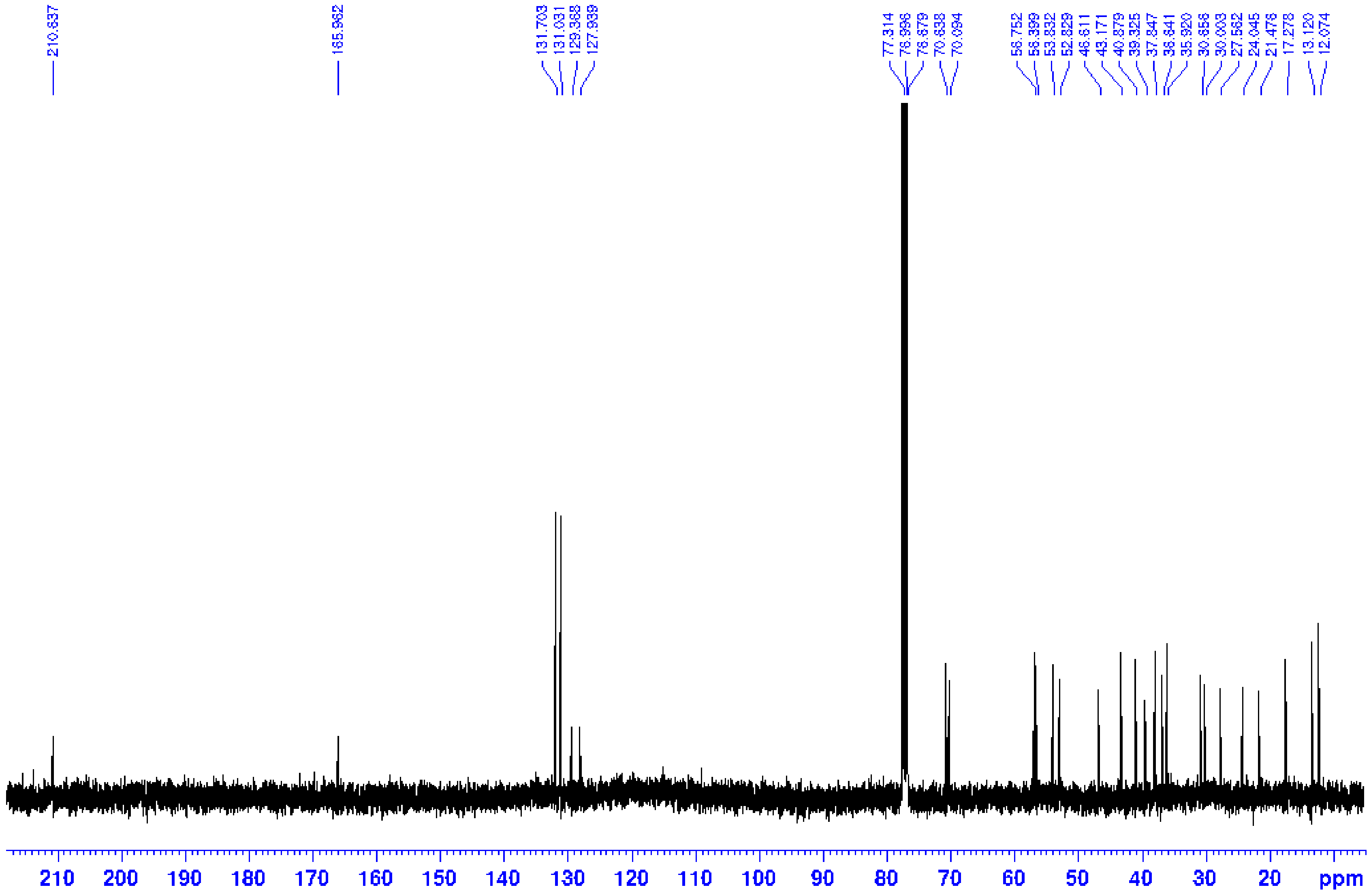


**Figure S80**. $^{13}$C NMR spectrum of 3β-hydroxy-5α-cholan-6-oxo-23,24-dinor-22-(4-bromo)benzoate-22-yl (**19**).

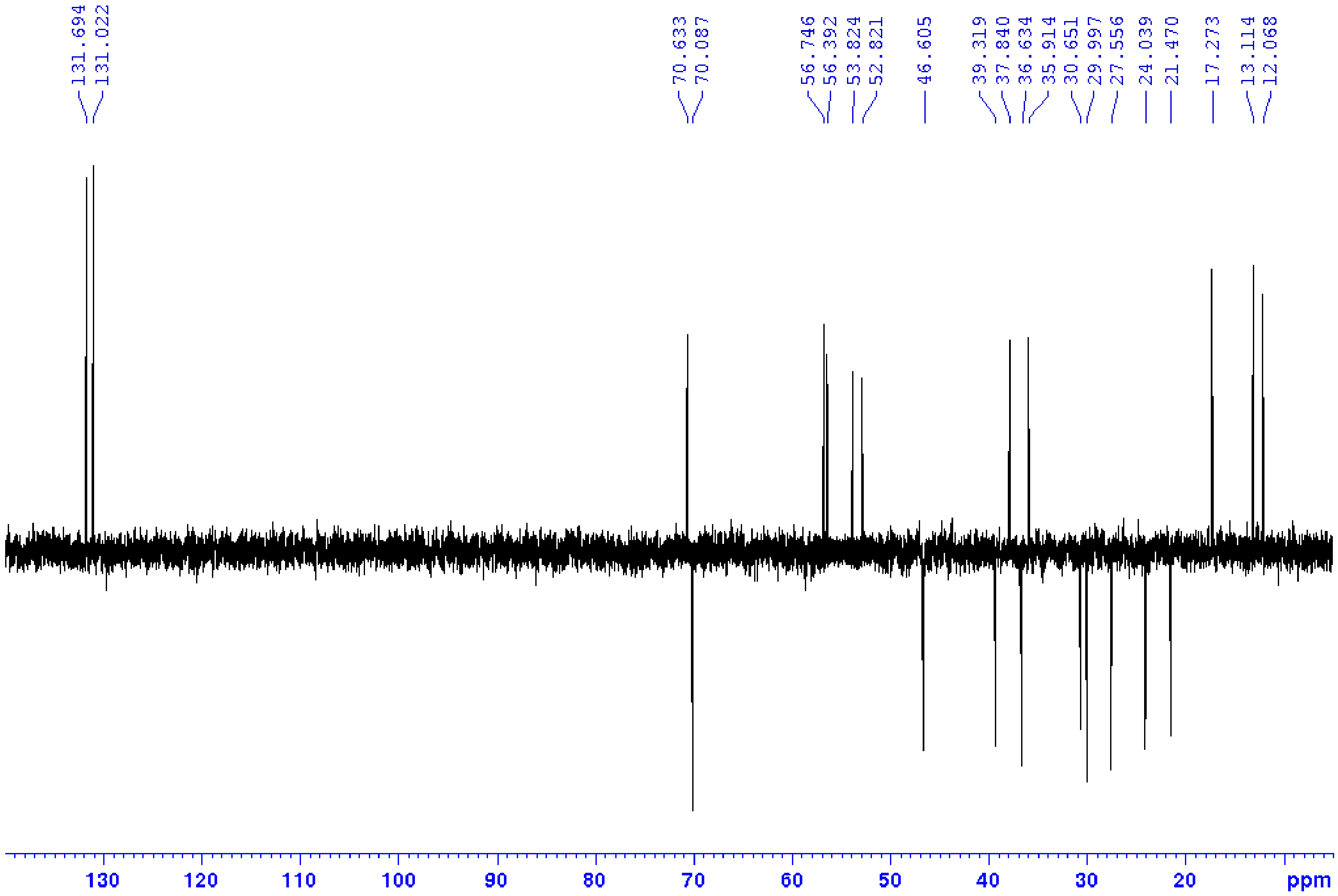


**Figure S81**. $^{13}$C DEPT-135 NMR spectrum of 3β-hydroxy-5α-cholan-6-oxo-23,24-dinor-22-(4-bromo)benzoate-22-yl (**19**).

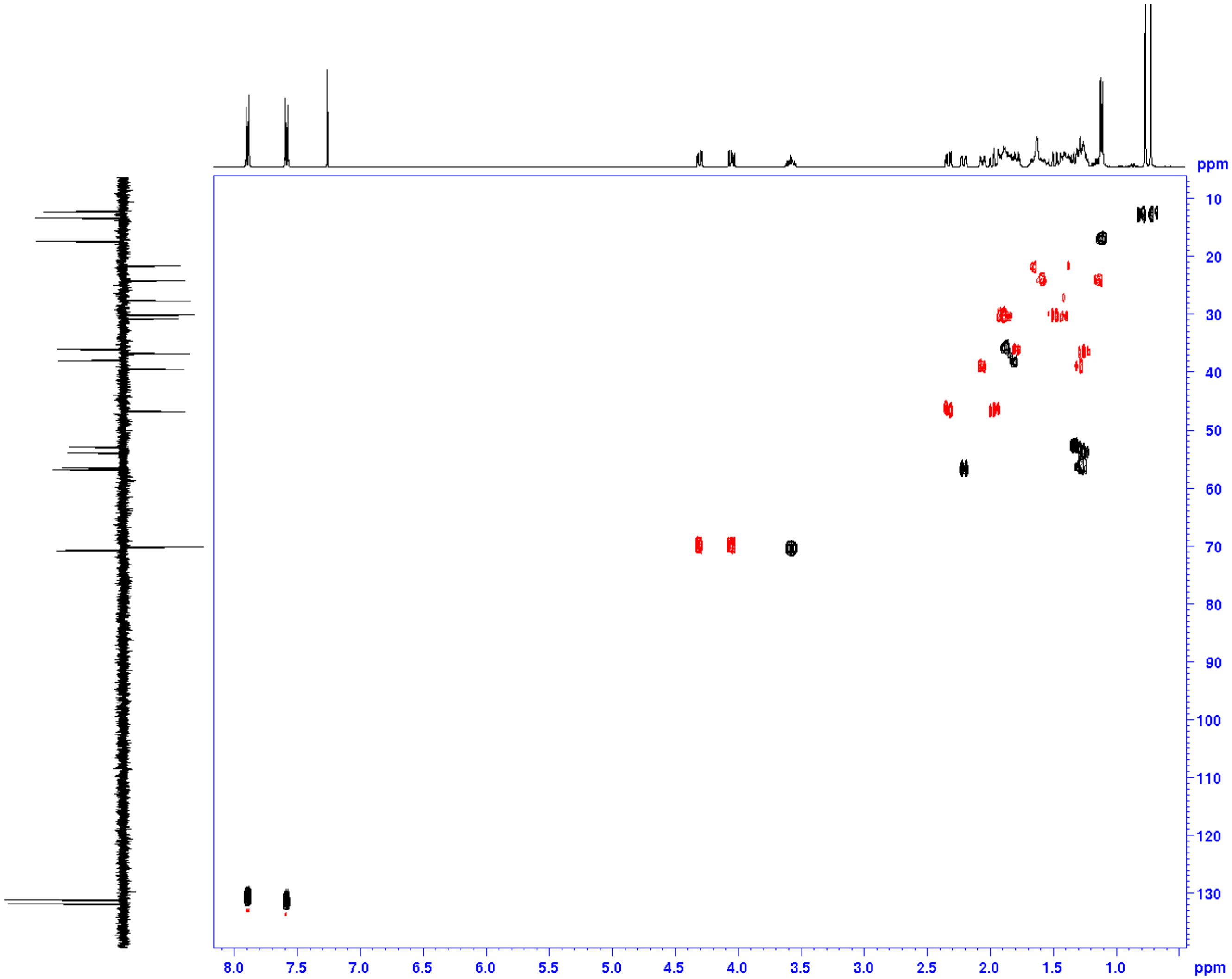


**Figure S82**. 2D HSQC NMR spectrum of 3β-hydroxy-5α-cholan-6-oxo-23,24-dinor-22-(4-bromo)benzoate-22-yl (**19**).

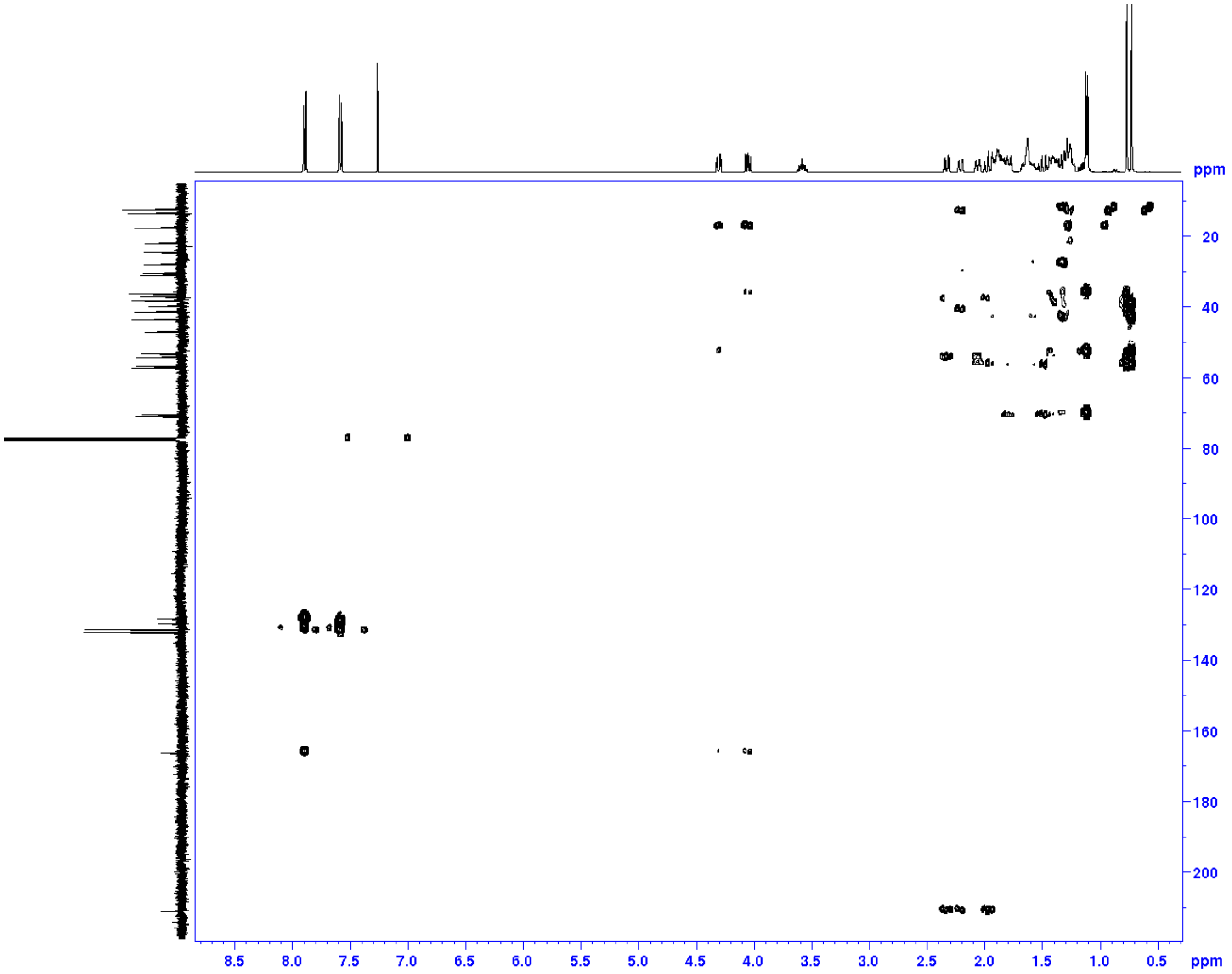


**Figure S83**. 2D HSQC NMR spectrum of 3β-hydroxy-5α-cholan-6-oxo-23,24-dinor-22-(4-bromo)benzoate-22-yl (**19**).

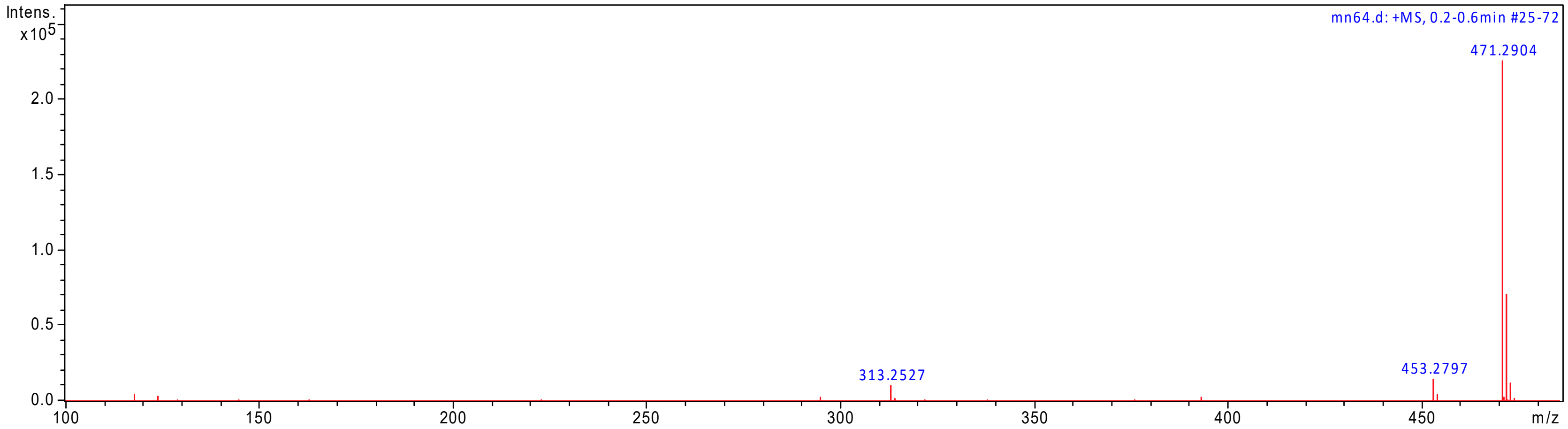


**Figure S84**. HRSM spectrum of 3β-hydroxy-5α-cholan-6-oxo-23,24-dinor-22-(4-fluoro)benzoate-22-yl (**20**).

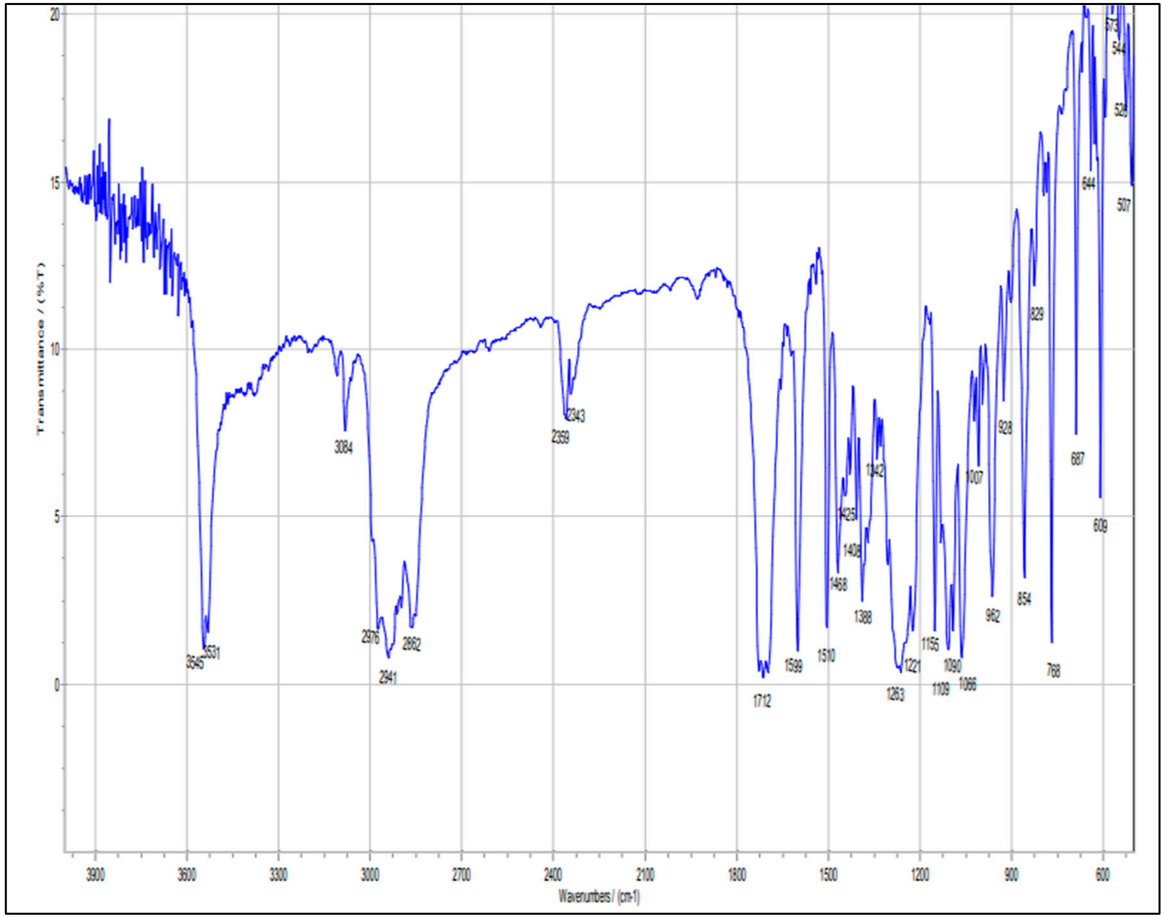


**Figure S85**. IR spectrum of 3β-hydroxy-5α-cholan-6-oxo-23,24-dinor-22-(4-fluoro)benzoate-22-yl (**20**).

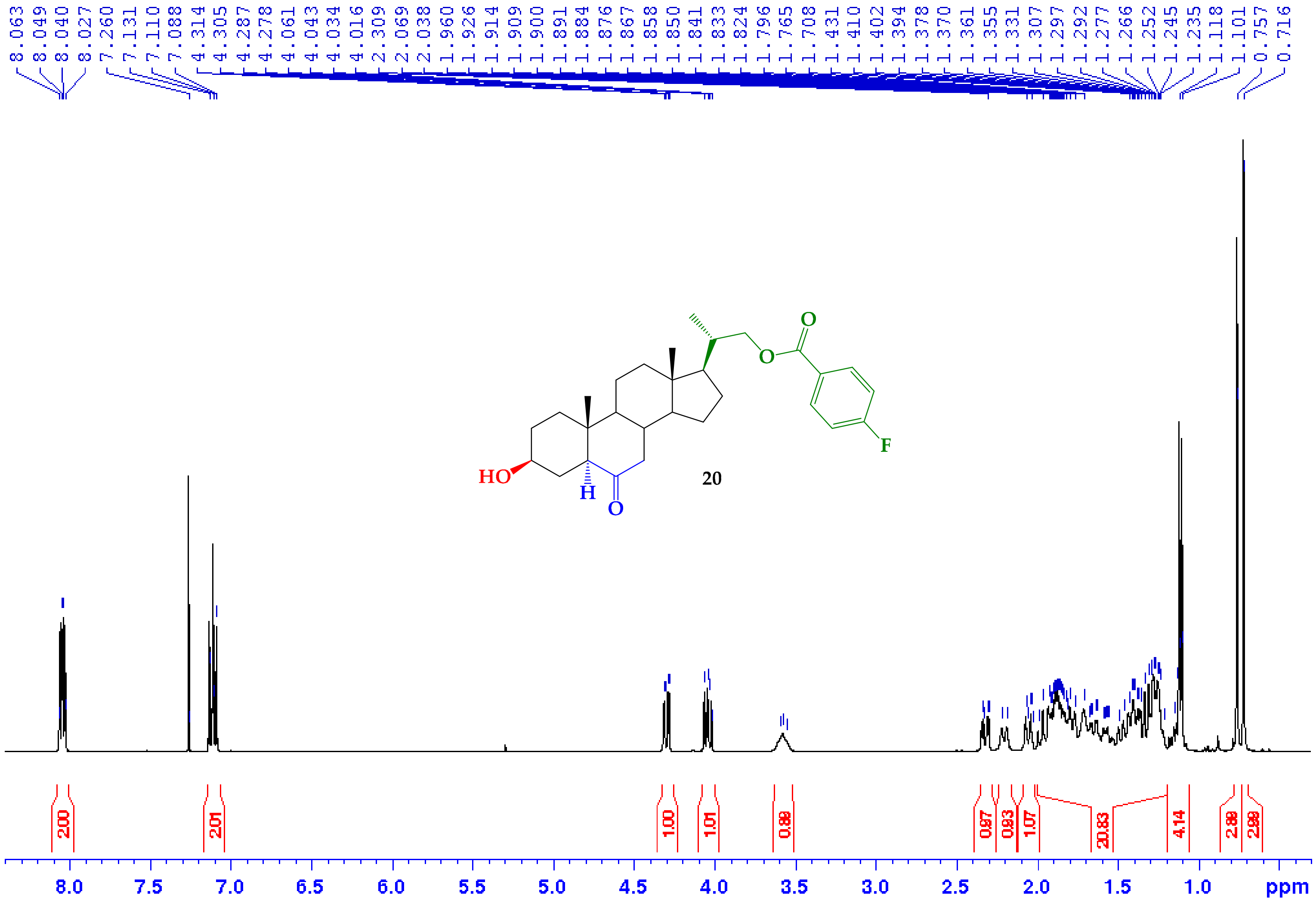

**Figure S86**. $^{1}$H NMR spectrum of 3β-hydroxy-5α-cholan-6-oxo-23,24-dinor-22-(4-fluoro)benzoate-22-yl (**20**).

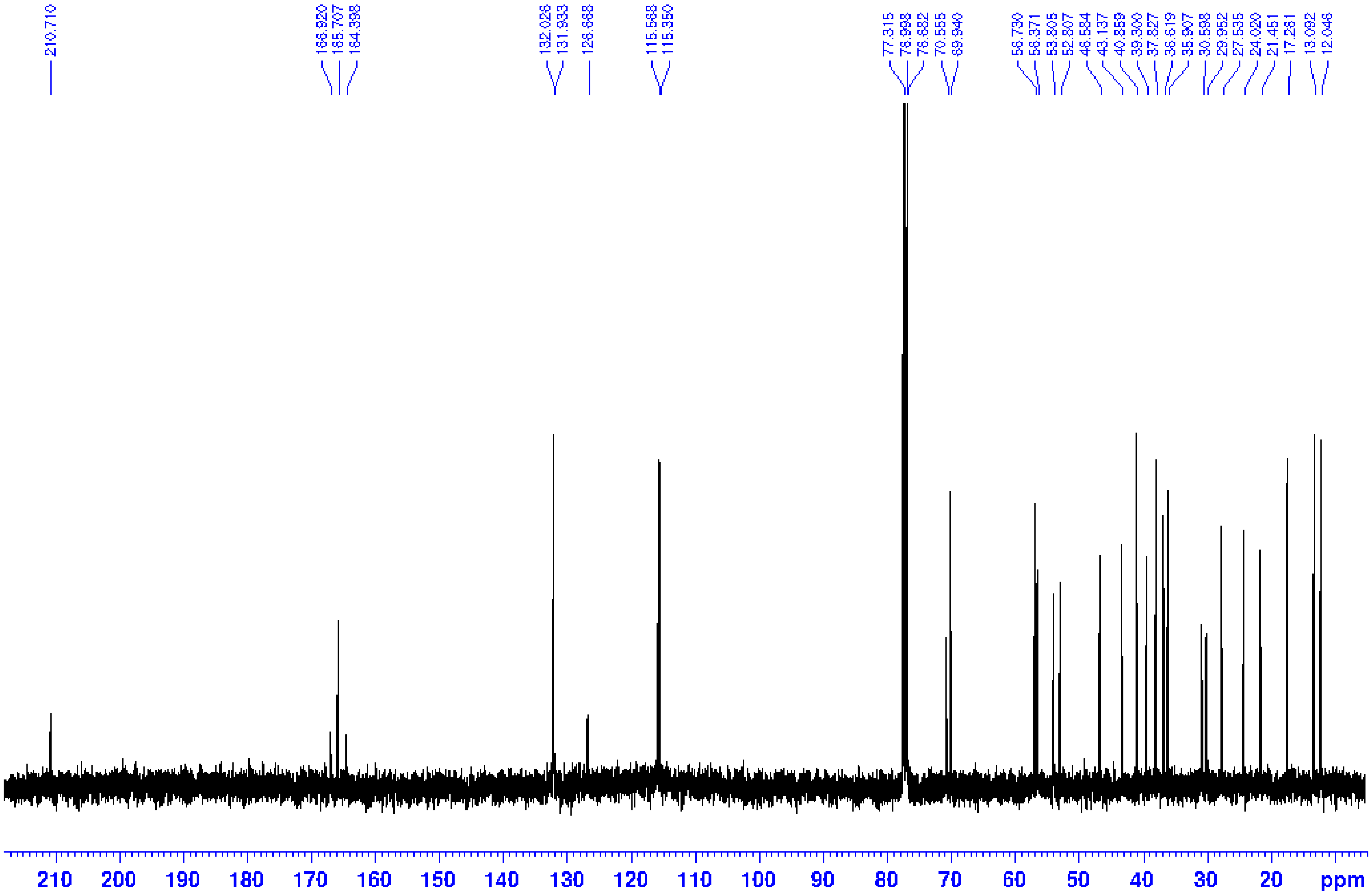


**Figure S87**. $^{13}$C NMR spectrum of 3β-hydroxy-5α-cholan-6-oxo-23,24-dinor-22-(4-fluoro)benzoate-22-yl (**20**).

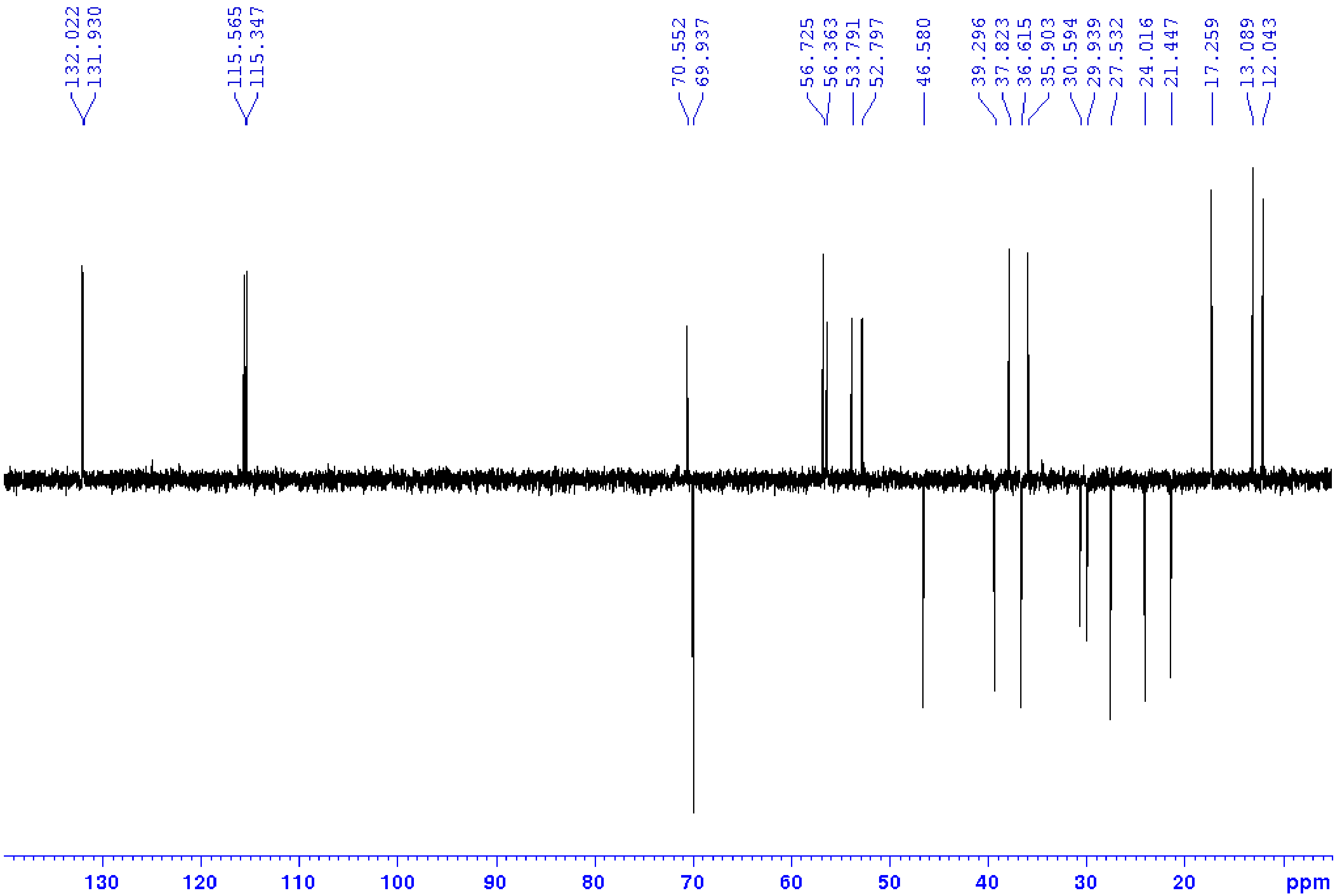


**Figure S88**. $^{13}$C DEPT-135 NMR spectrum of 3β-hydroxy-5α-cholan-6-oxo-23,24-dinor-22-(4-fluoro)benzoate-22-yl (**20**).

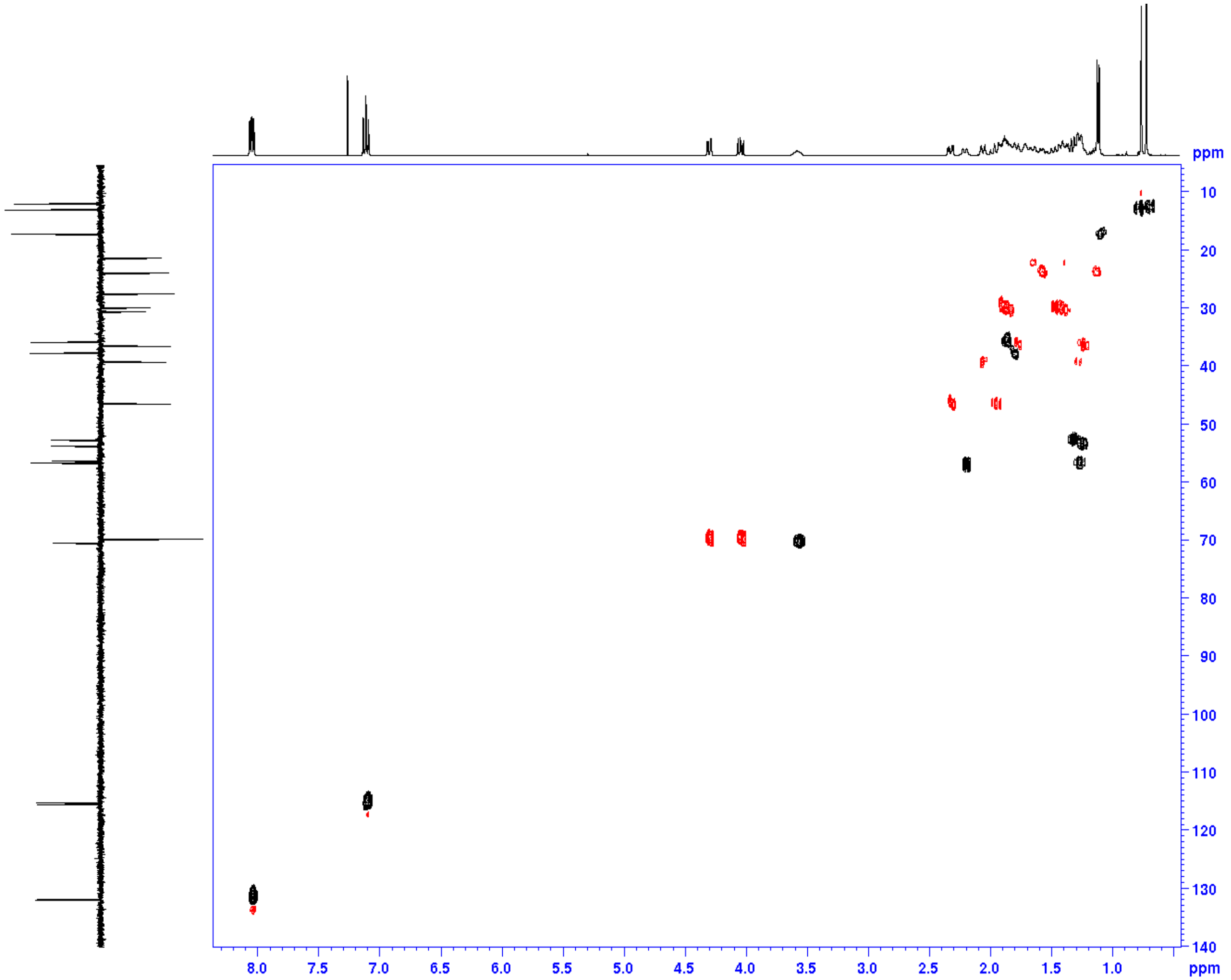


**Figure S89**. 2D HSQC NMR spectrum of 3β-hydroxy-5α-cholan-6-oxo-23,24-dinor-22-(4-fluoro)benzoate-22-yl (**20**).

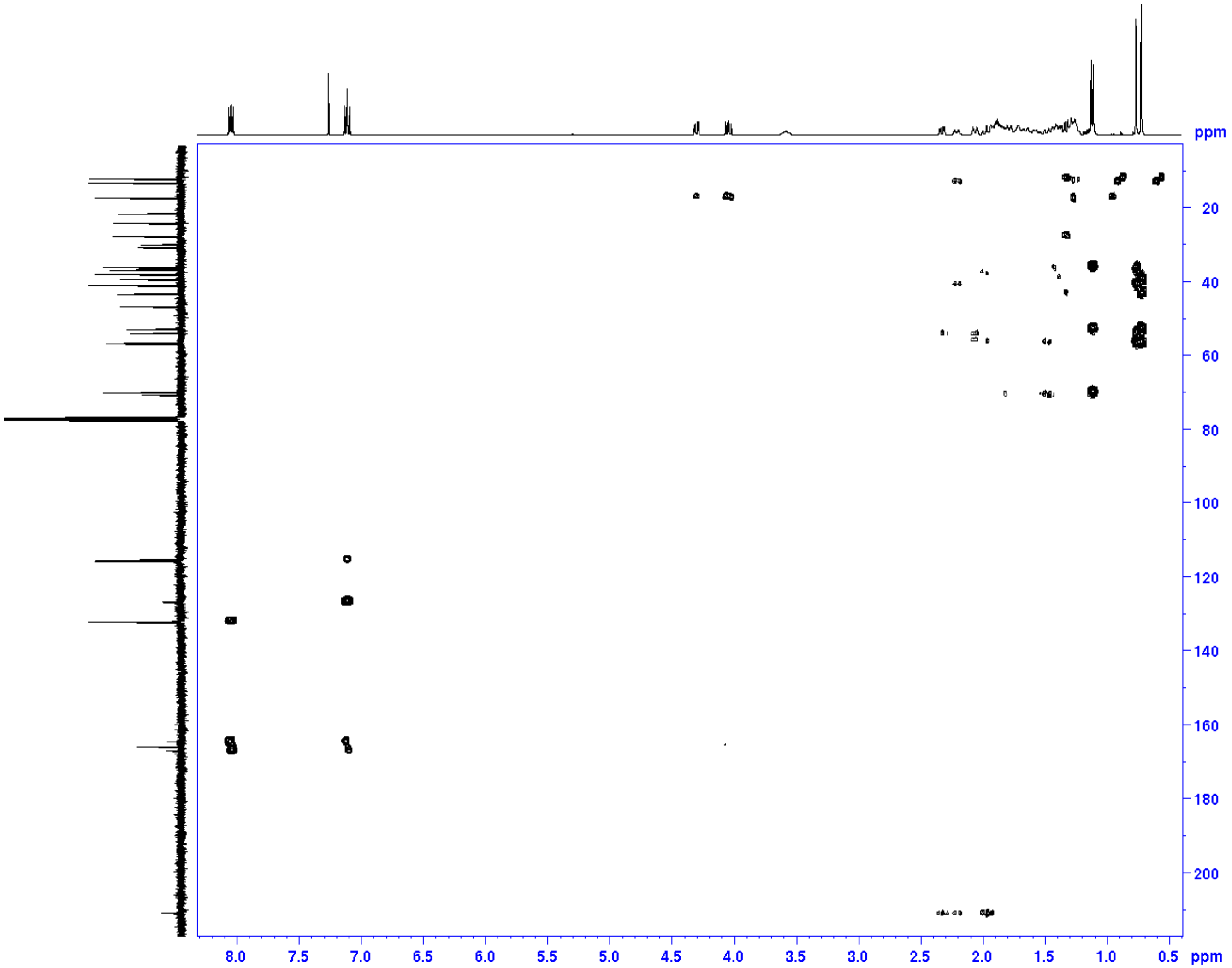


**Figure S90**. 2D HMBC NMR spectrum of 3β-hydroxy-5α-cholan-6-oxo-23,24-dinor-22-(4-fluoro)benzoate-22-yl (**20**).

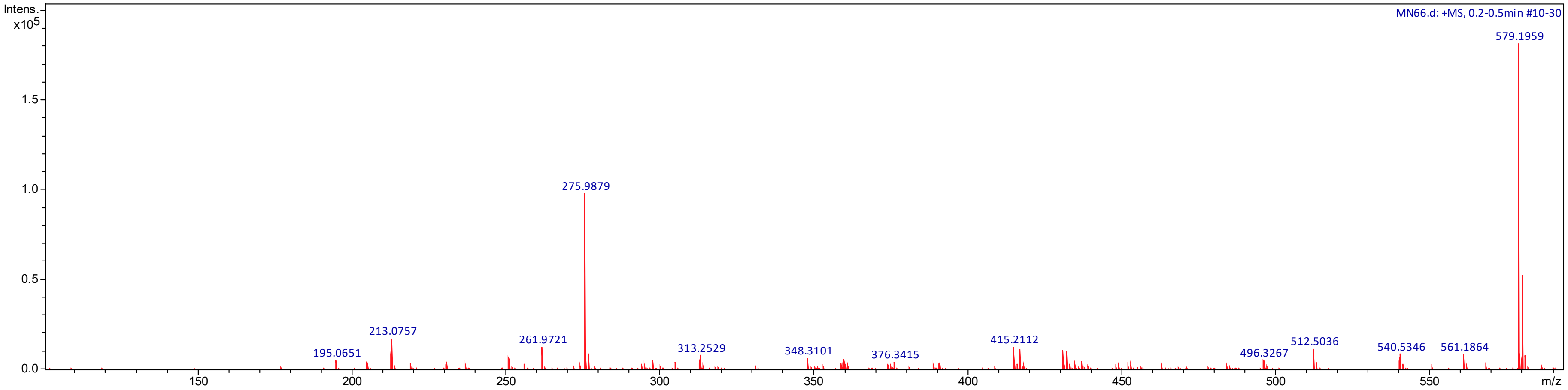


**Figure S91**. HRSM spectrum of 3β-hydroxy-5α-cholan-6-oxo-23,24-dinor-22-(4-iodine)benzoate-22-yl (**21**).

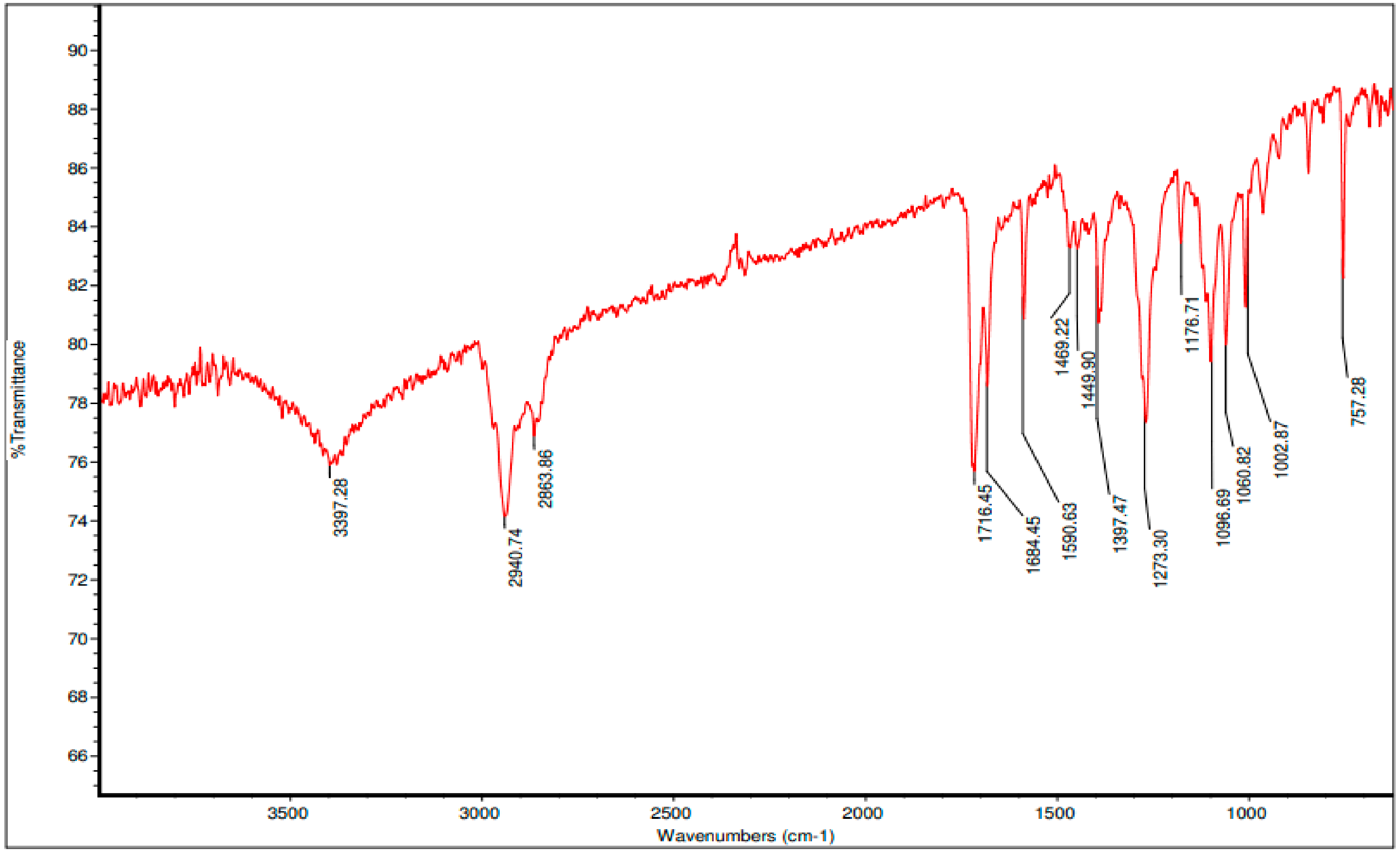


**Figure S92**. IR spectrum of 3β-hydroxy-5α-cholan-6-oxo-23,24-dinor-22-(4-iodine)benzoate-22-yl (**21**).

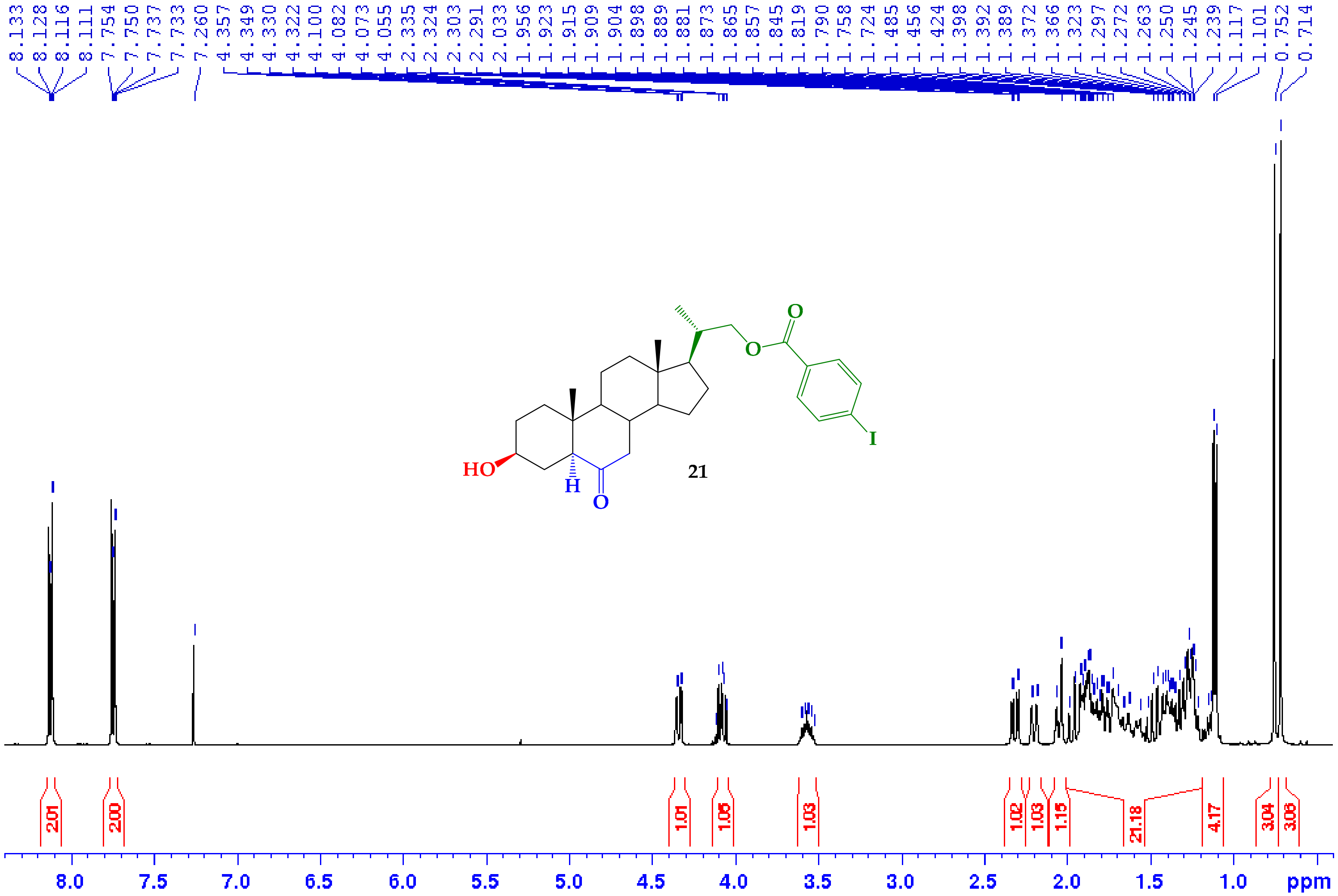

**Figure S93**. $^{1}$H NMR spectrum of 3β-hydroxy-5α-cholan-6-oxo-23,24-dinor-22-(4-iodine)benzoate-22-yl (**21**).

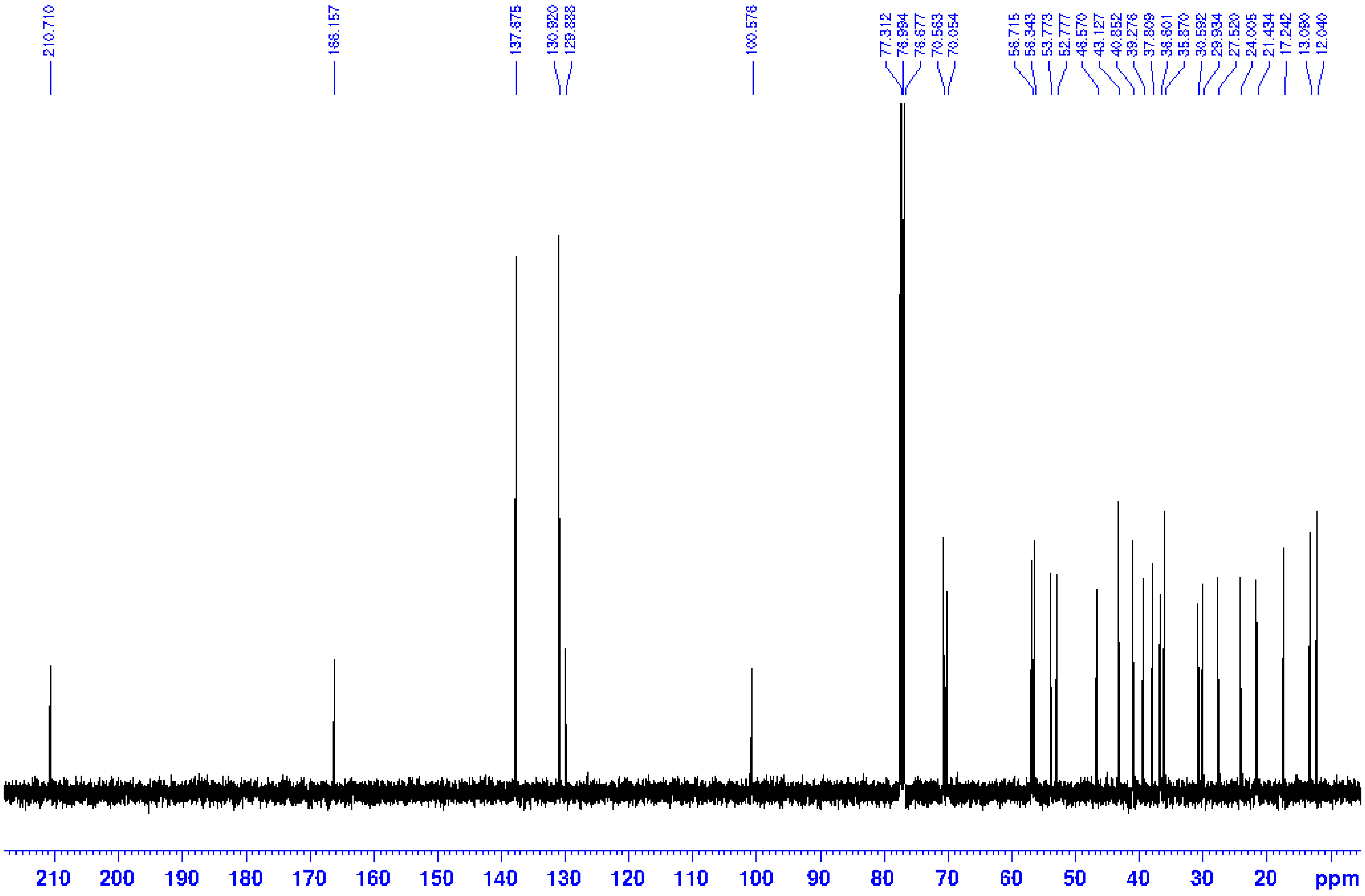


**Figure 94.** $^{13}$C NMR spectrum of 3β-hydroxy-5α-cholan-6-oxo-23,24-dinor-22-(4-iodine)benzoate-22-yl (**21**).

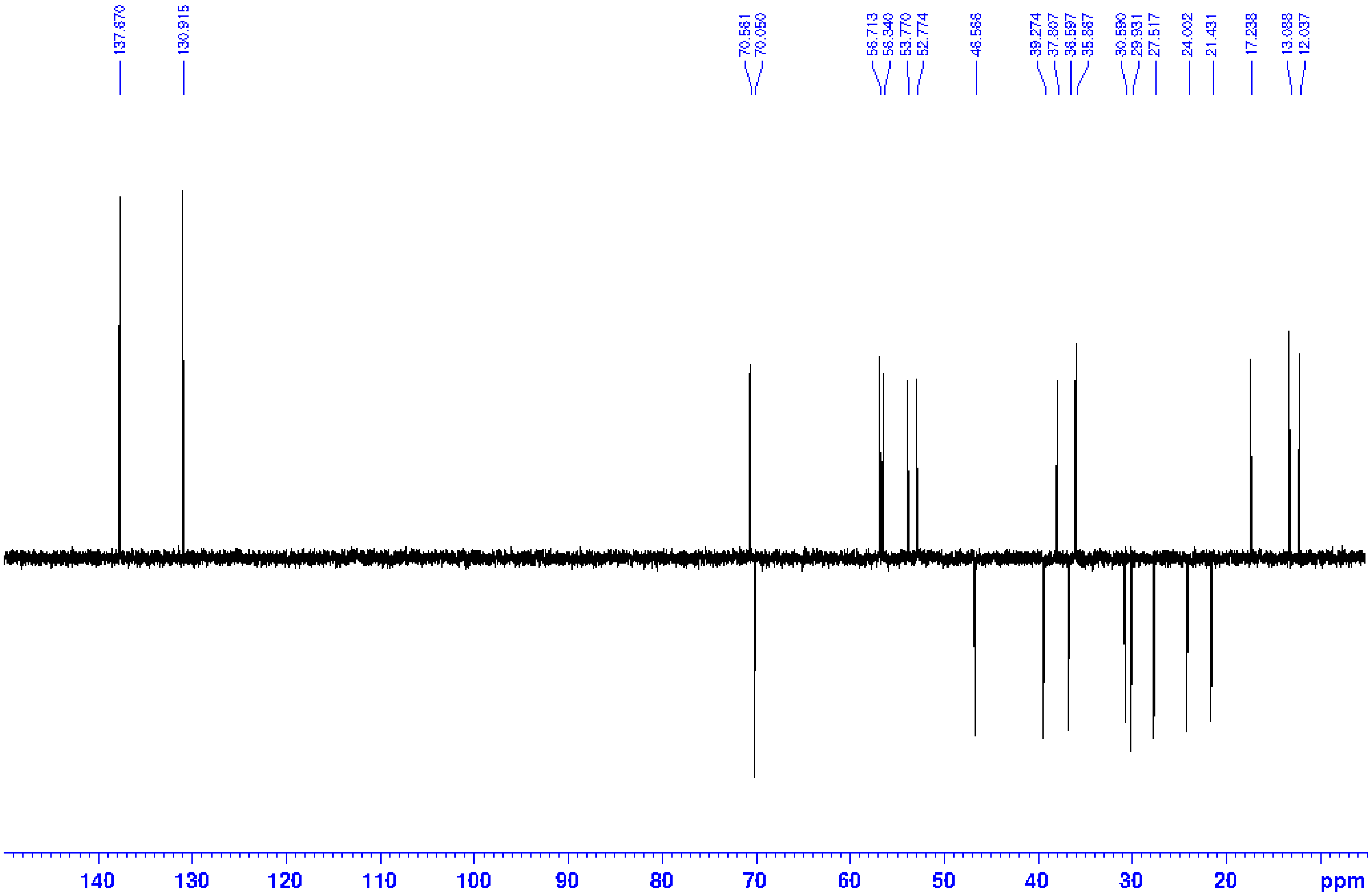


**Figure 95.** $^{13}C$ DEPT-135 NMR spectrum of 3β-hydroxy-5α-cholan-6-oxo-23,24-dinor-22-(4-iodine)benzoate-22-yl (**21**).

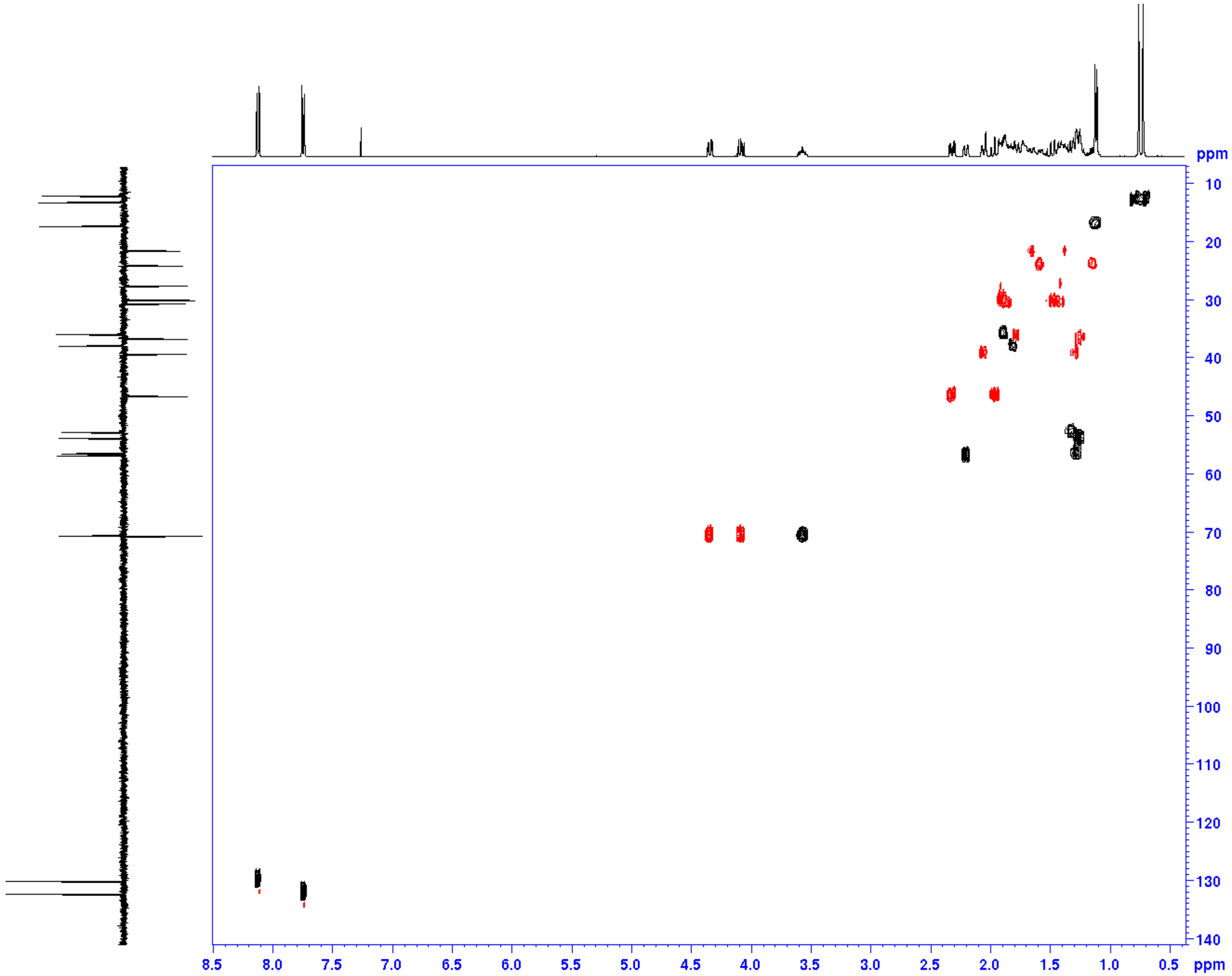


**Figure 96.** 2D HSQC NMR spectrum of 3β-hydroxy-5α-cholan-6-oxo-23,24-dinor-22-(4-iodine)benzoate-22-yl (**21**).

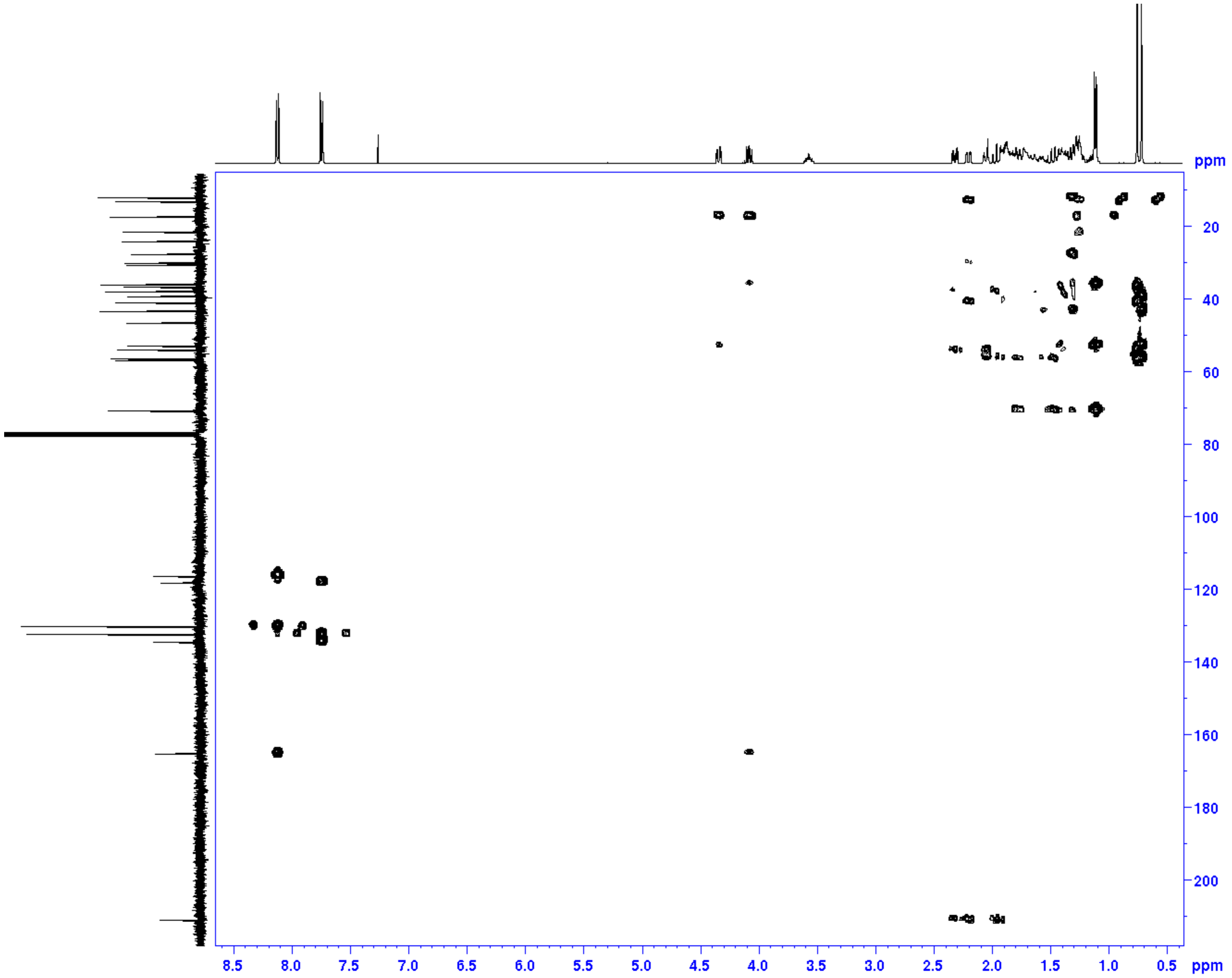


**Figure 97.** 2D HMBC NMR spectrum of 3β-hydroxy-5α-cholan-6-oxo-23,24-dinor-22-(4-iodine)benzoate-22-yl (**21**).

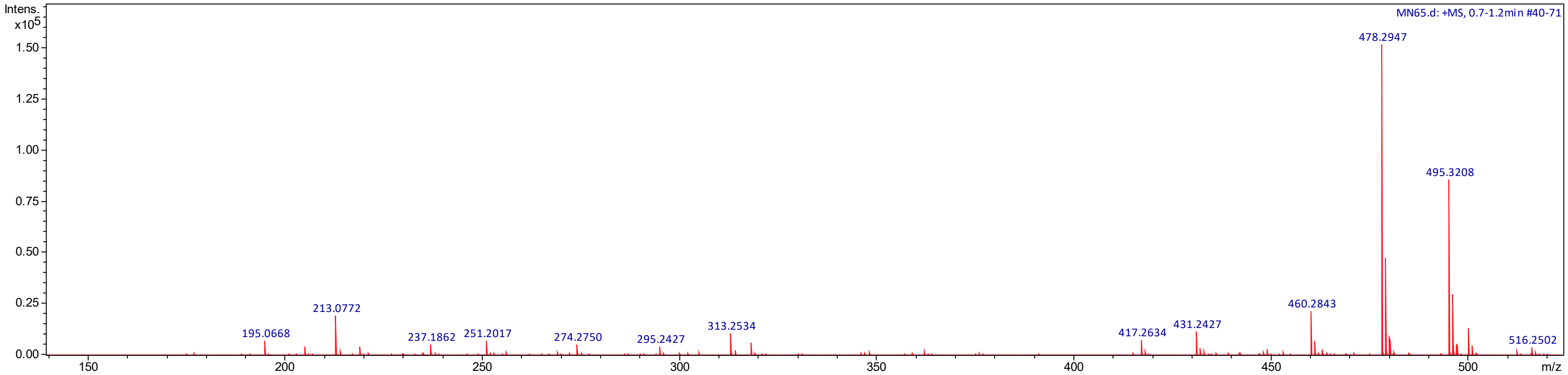


**Figure S98**. HRSM spectrum of 3β-hydroxy-5α-cholan-6-oxo-23,24-dinor-22-(4-cyan)benzoate-22-yl (**22**).

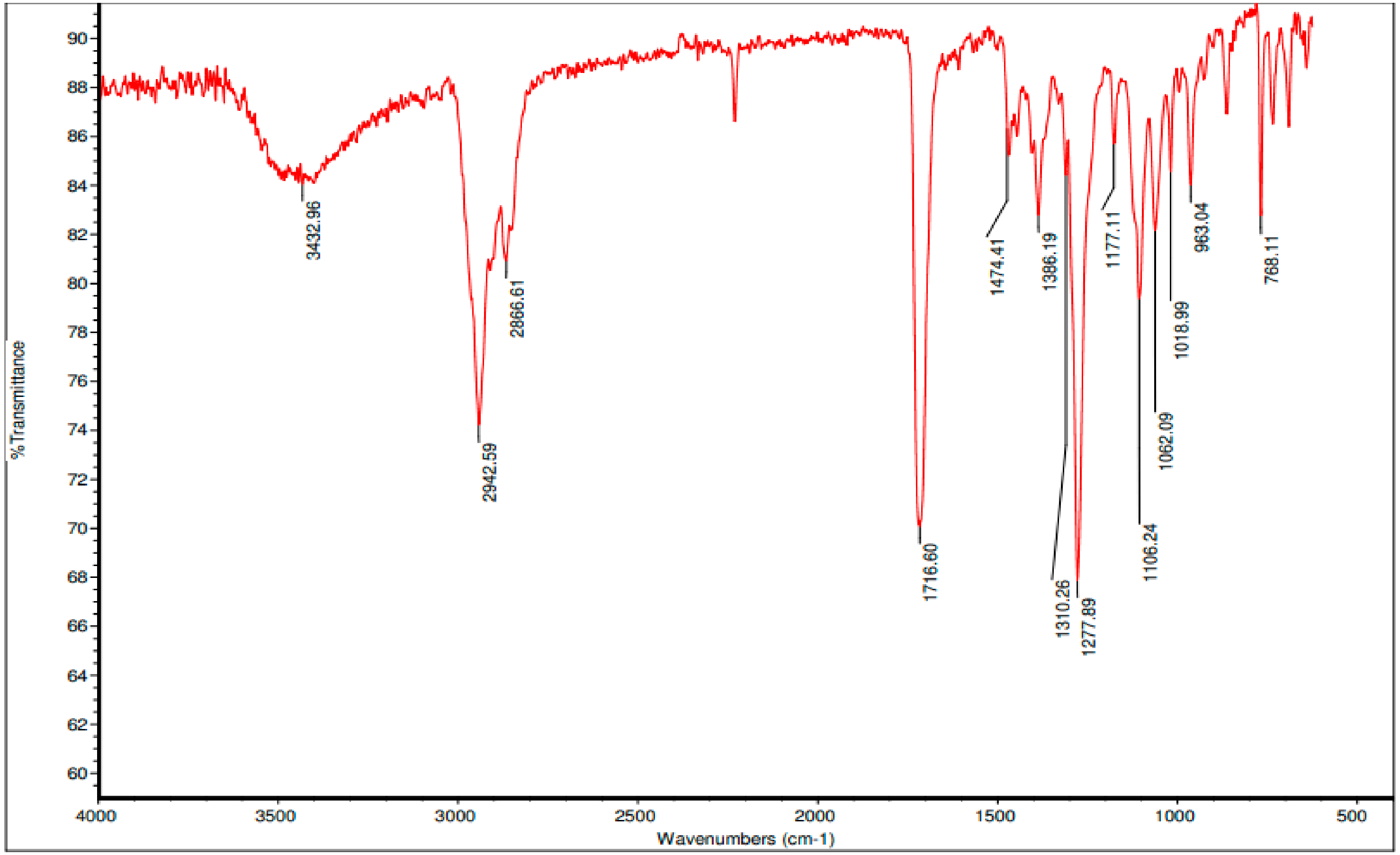


**Figure S99**. IR spectrum of 3β-hydroxy-5α-cholan-6-oxo-23,24-dinor-22-(4-cyan)benzoate-22-yl (**22**).

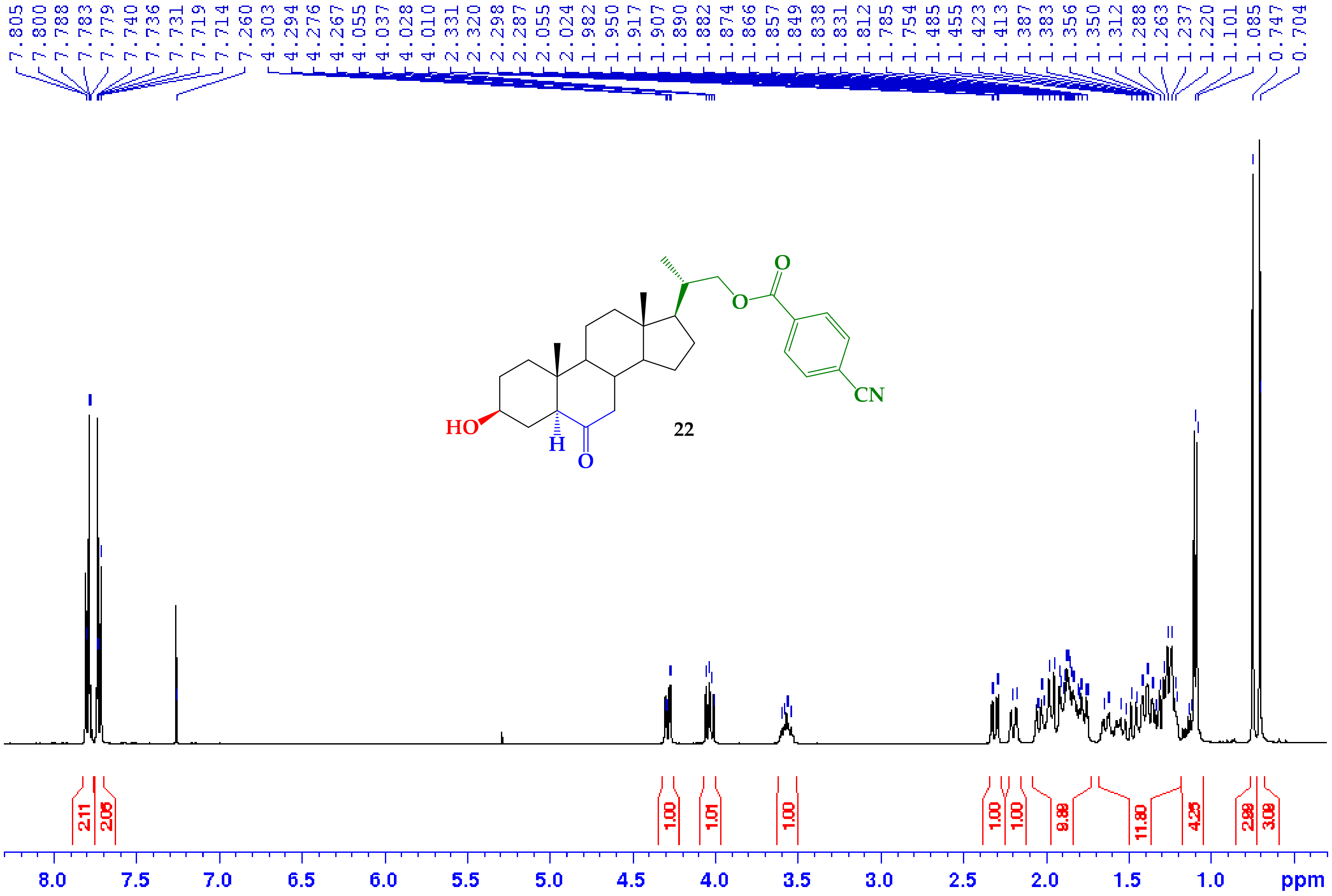

**Figure S100**. $^{1}$H NMR spectrum of 3β-hydroxy-5α-cholan-6-oxo-23,24-dinor-22-(4-cyan)benzoate-22-yl (**22**).

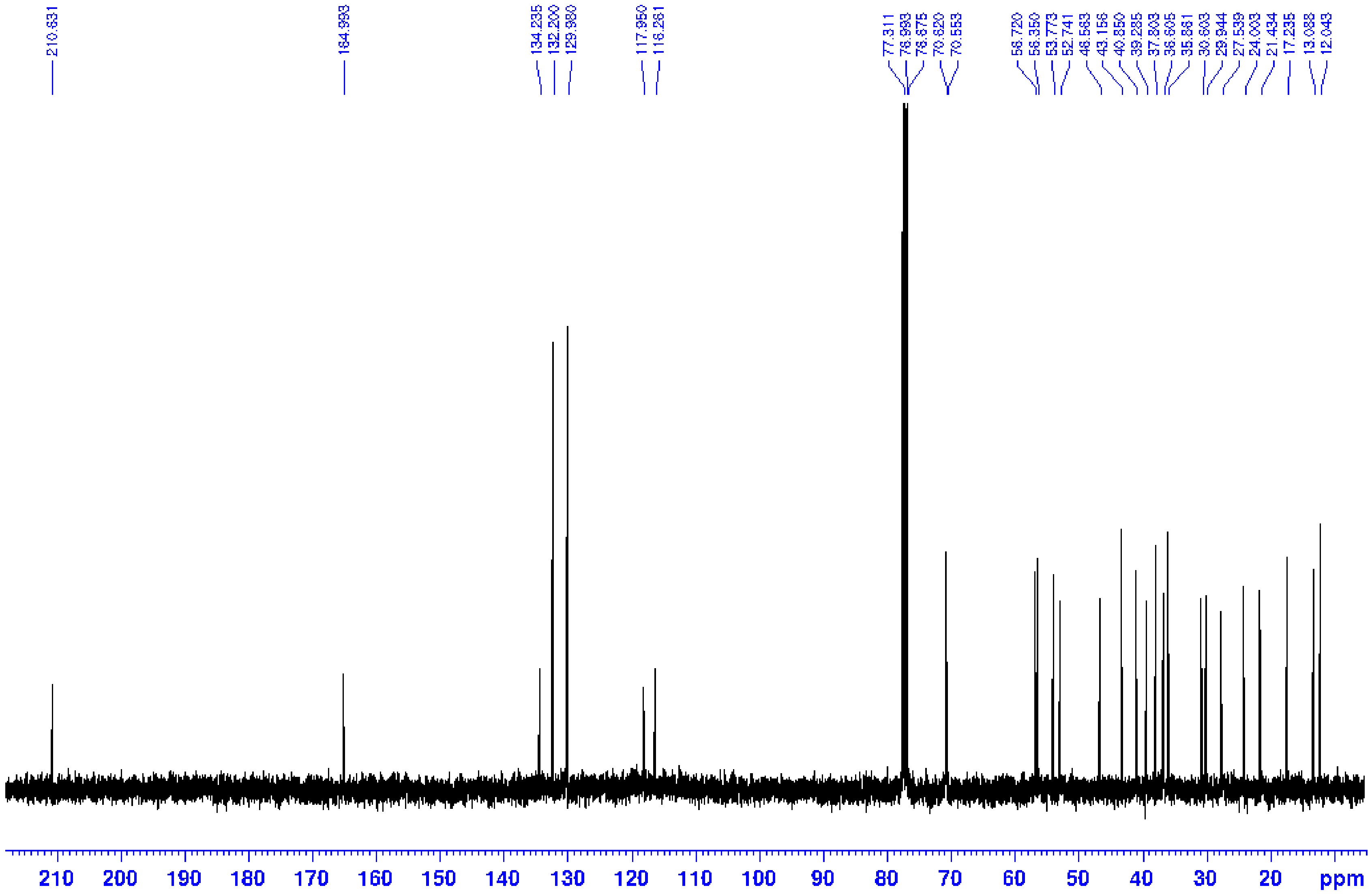


**Figure S101**. $^{13}$C NMR spectrum of 3β-hydroxy-5α-cholan-6-oxo-23,24-dinor-22-(4-cyan)benzoate-22-yl (**22**).

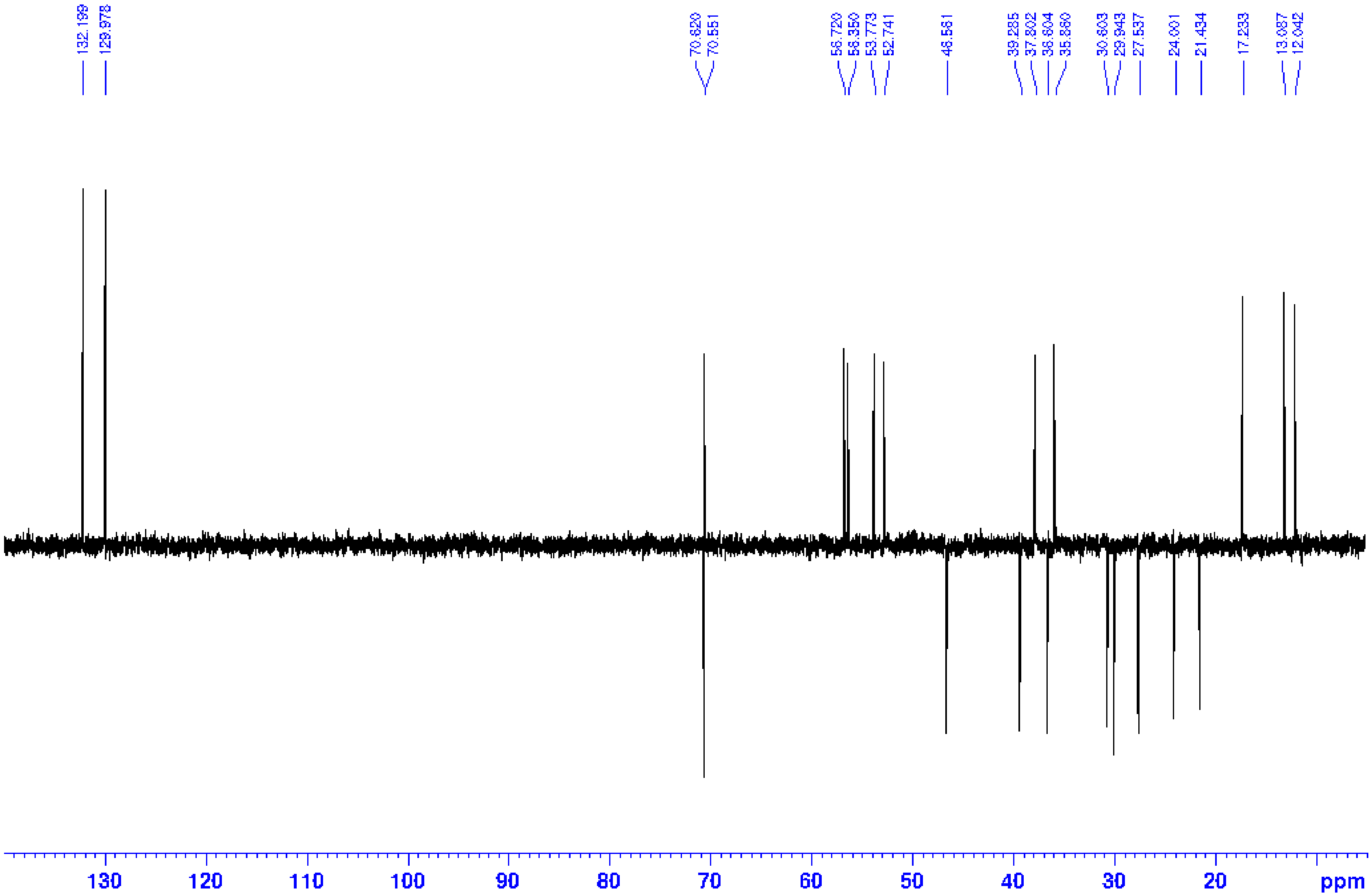


**Figure S102**. $^{13}C$ DEPT-135 NMR spectrum of 3β-hydroxy-5α-cholan-6-oxo-23,24-dinor-22-(4-cyan)benzoate-22-yl (**22**).

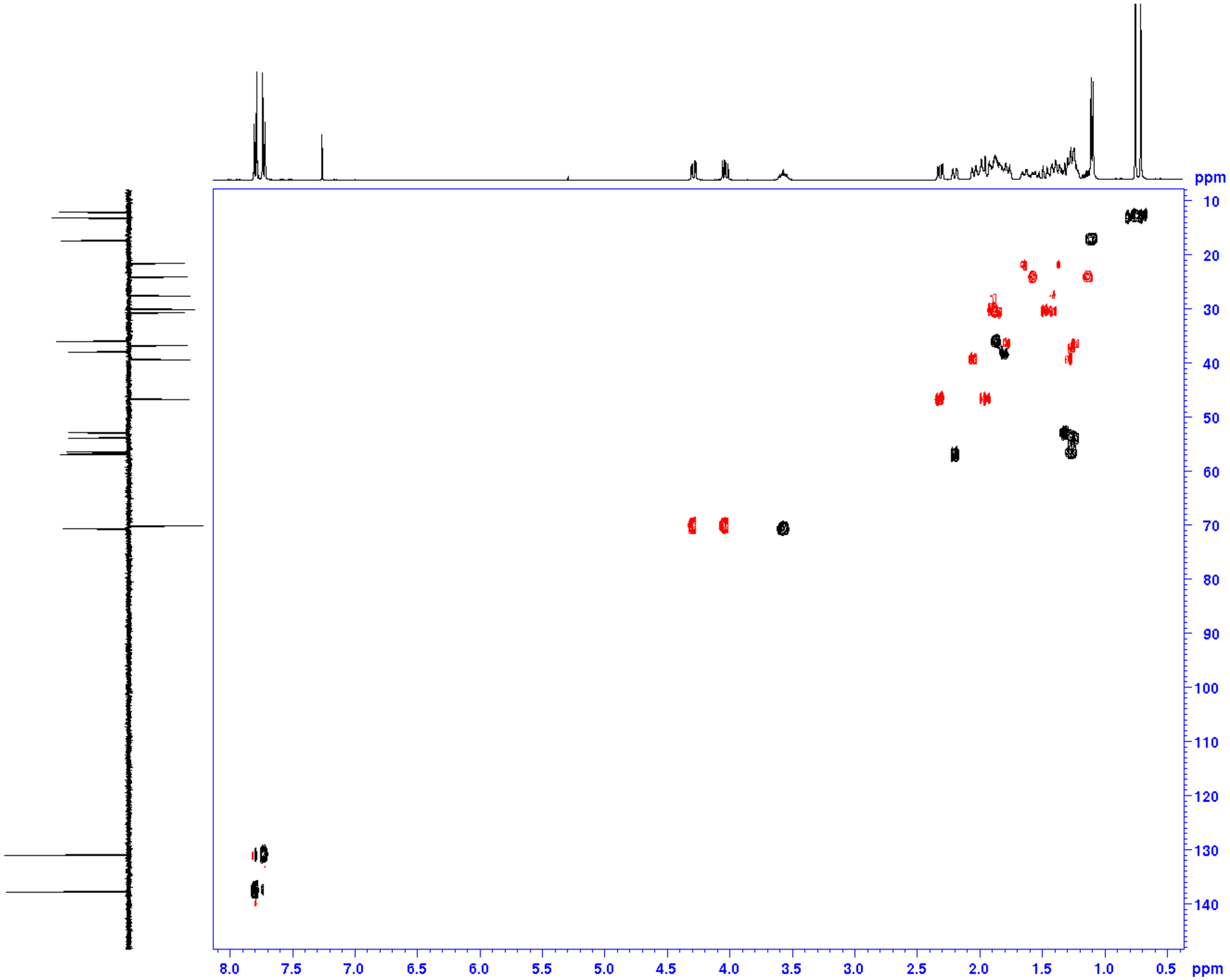


**Figure S103**. 2D HSQC NMR spectrum of 3β-hydroxy-5α-cholan-6-oxo-23,24-dinor-22-(4-cyan)benzoate-22-yl (**22**).

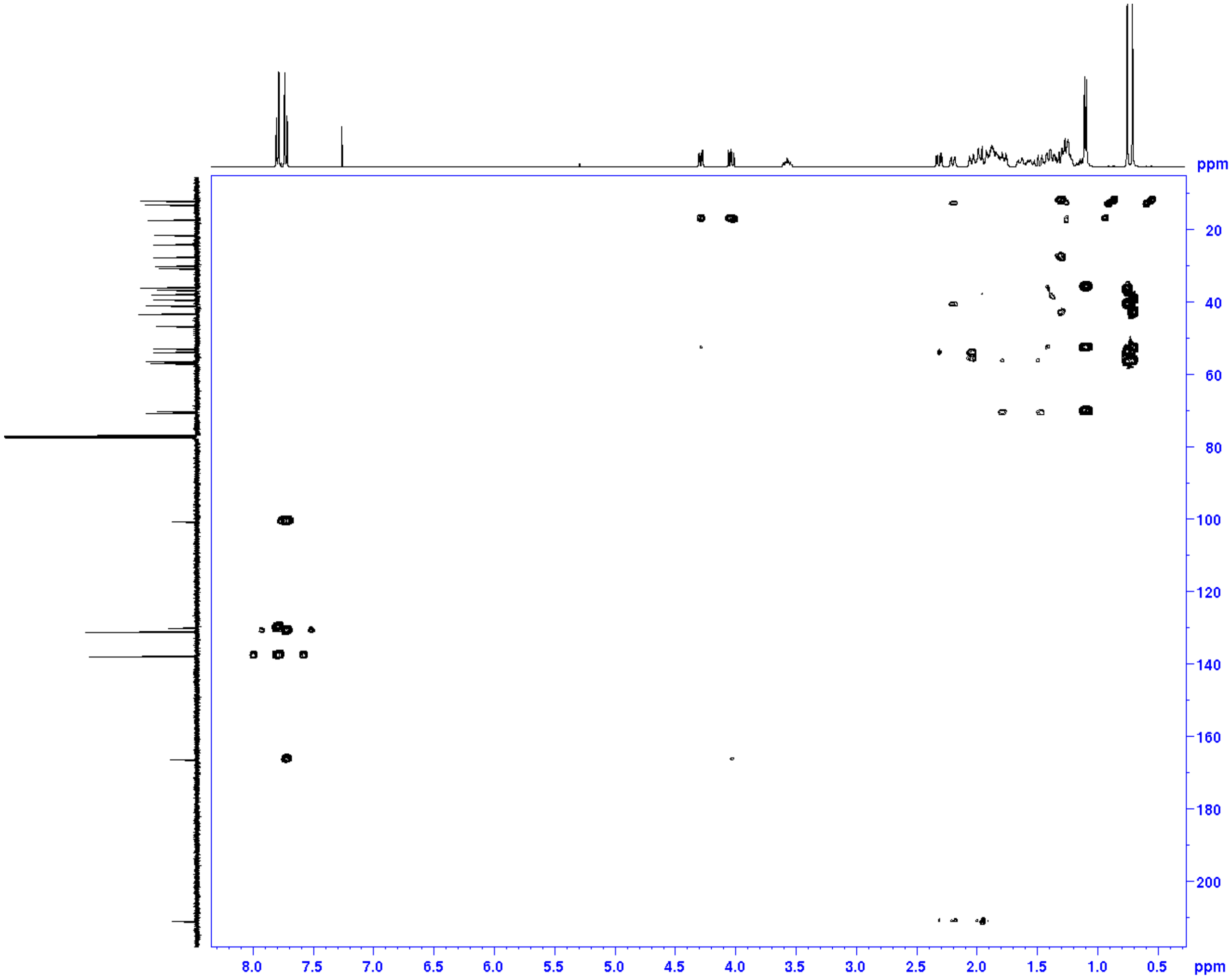


**Figure S104**. 2D HMBC NMR spectrum of 3β-hydroxy-5α-cholan-6-oxo-23,24-dinor-22-(4-cyan)benzoate-22-yl (**22**).

**Table S1:** Rice-lamina assays using the second leaf lamina joints (Angle Opening, Degrees) of excised leaf segments treated with BRs analogs (**1**, **15**-**22** and **31**) at different concentrations. Brassinolide was used as positive control at the same concentrations.

| | **RLIT (Angle Opening, Degrees)** | | |
|---|---|---|---|
| **Compounds** | **1 x 10 $^{-8}$ M** | **1 x 10 $^{-7}$ M** | **1 x 10 $^{-6}$ M** |
| 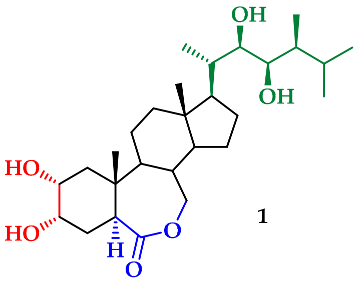 **1** | 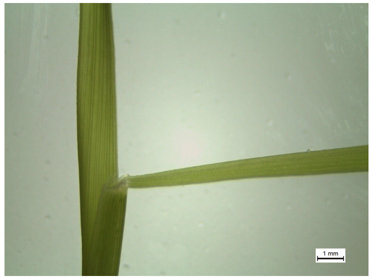 | 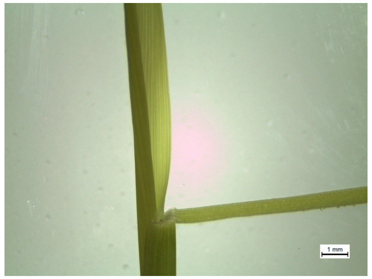 | 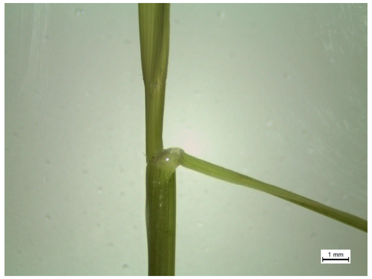 |
| 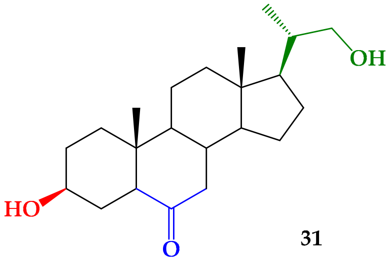 **31** | 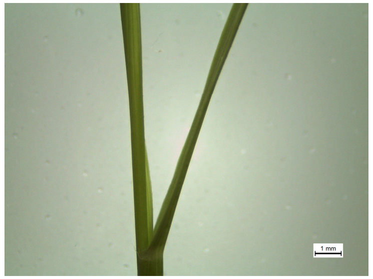 | 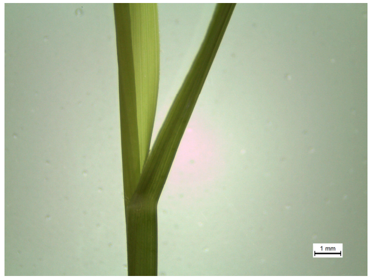 | 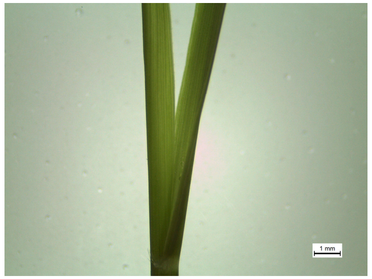 |
| 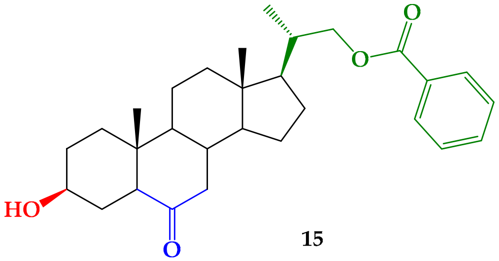 **15** | 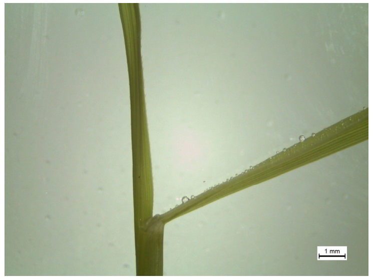 | 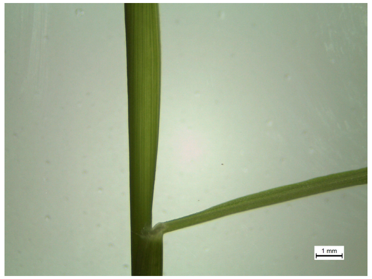 | 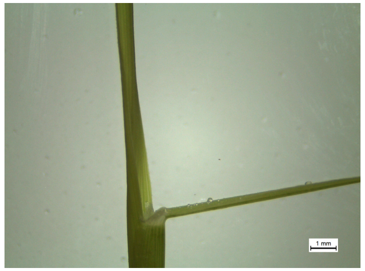 |

| | | | |
|---|---|---|---|
| 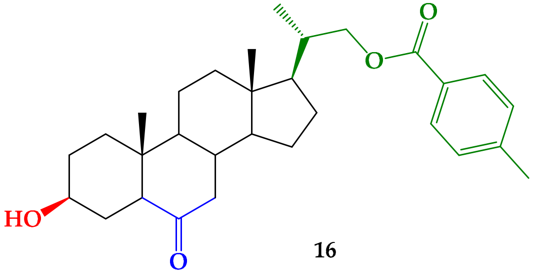 **16** | 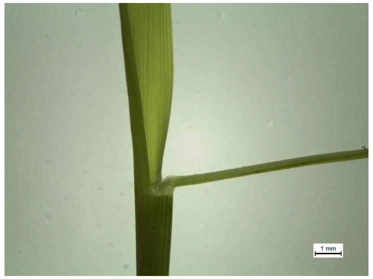 | 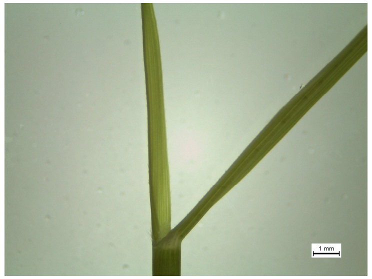 | 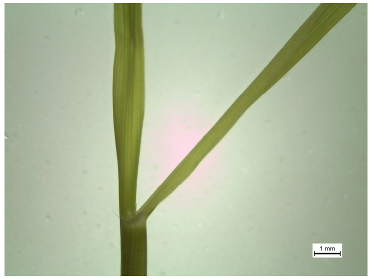 |
| 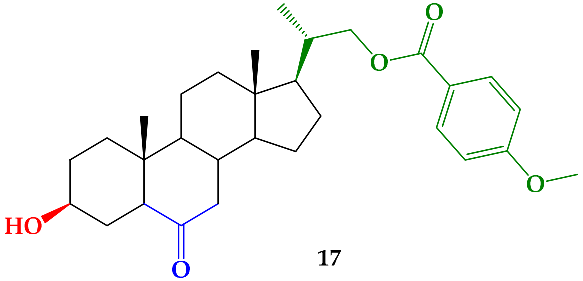 **17** | 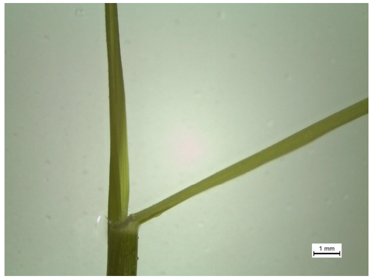 | 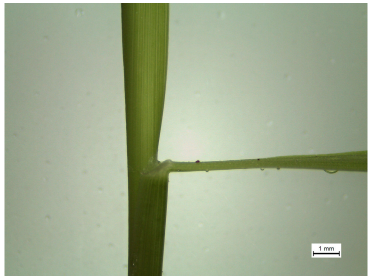 | 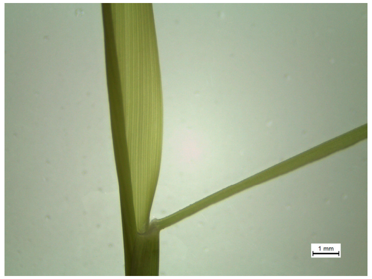 |
| 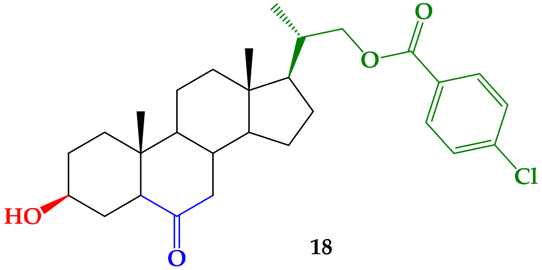 **18** | 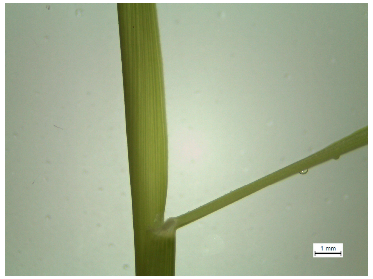 | 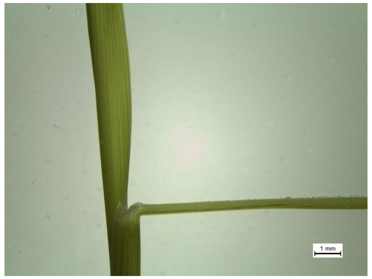 | 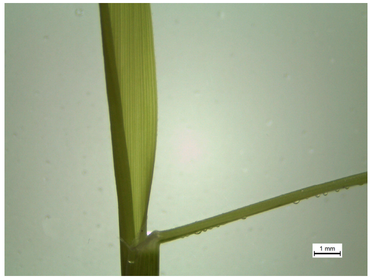 |

| | | | |
|---|---|---|---|
| 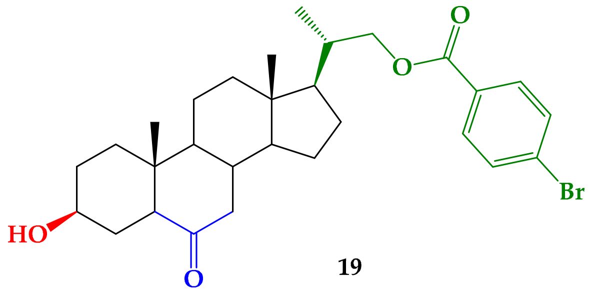 **19** | 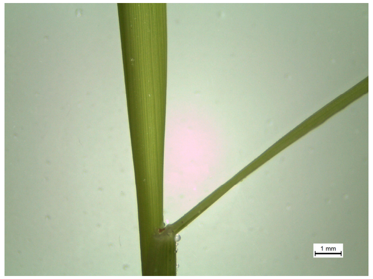 | 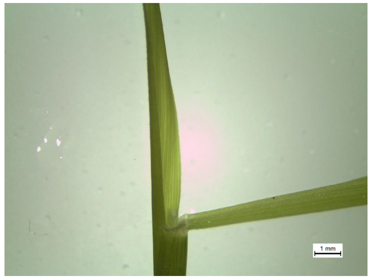 | 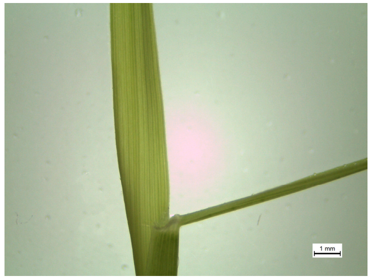 |
| 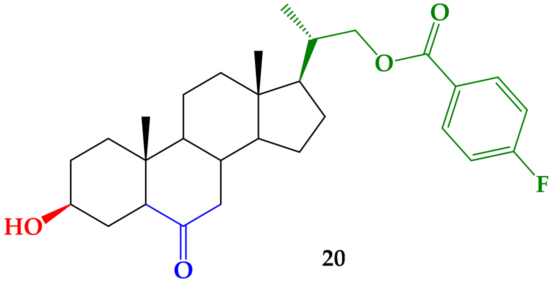 **20** | 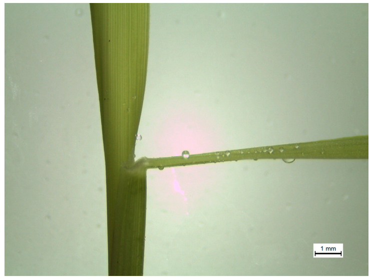 | 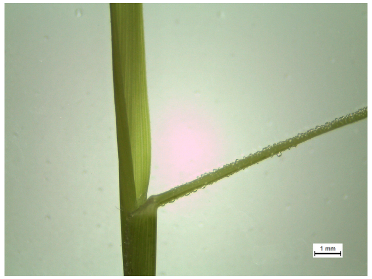 | 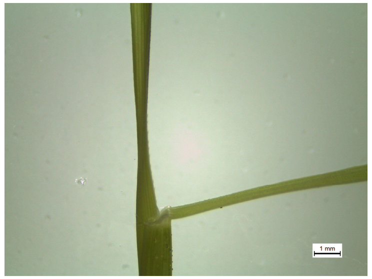 |
| 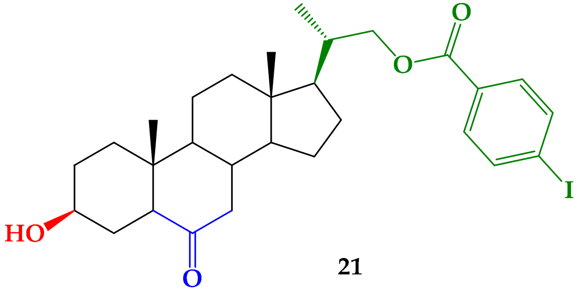 **21** | 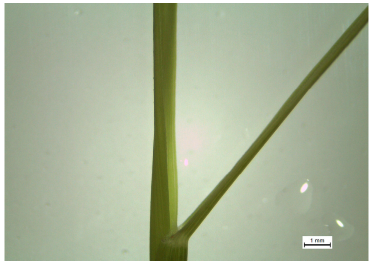 | 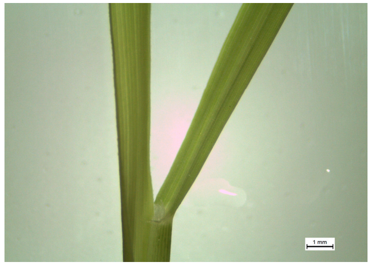 | 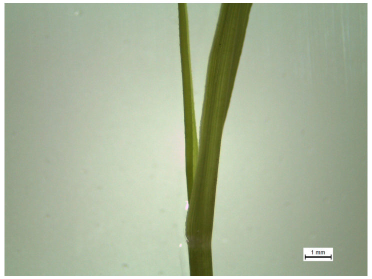 |

| | | | |
|---|---|---|---|
| 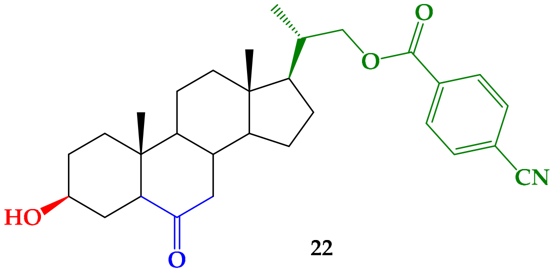  **22** | 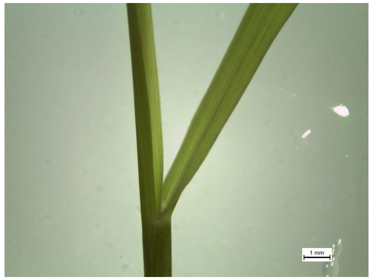 | 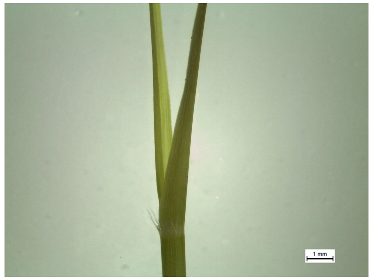 | 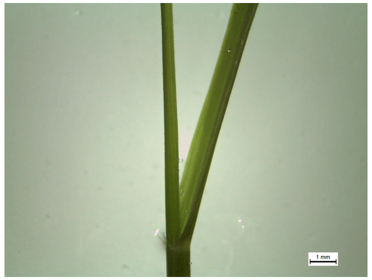 |
| C (-) | 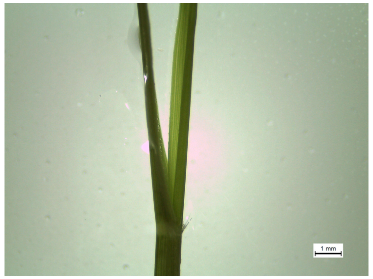 | | |

**Table S2:** Analysis of docked brassinolide (1) and synthetic analogs (1, 31 and 15-22). ΔEb: Binding Energy in kcal/mol. Hydrogen bonds are represented in yellow segmented lines. π-π stacking are represented in green (turquoise) segmented lines. Visualization of the docked poses was performed using Discovery Studio Visualizer (BIOVIA, San Diego, CA, USA).

| **Com-pounds** | **binding modes** | **ΔEb Glide Score** | **Hydrogen bond and π-π stacking interactions** |
|---|---|---|---|

| | | | |
|---|---|---|---|
| **15** | 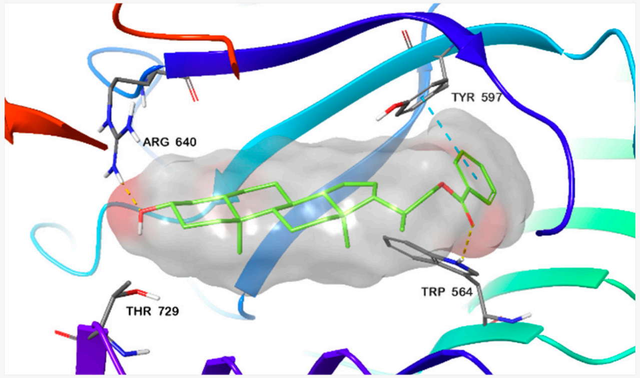 | **-11.96** | **Arg640, Trp 564, Tyr 597.** |
| **16** | 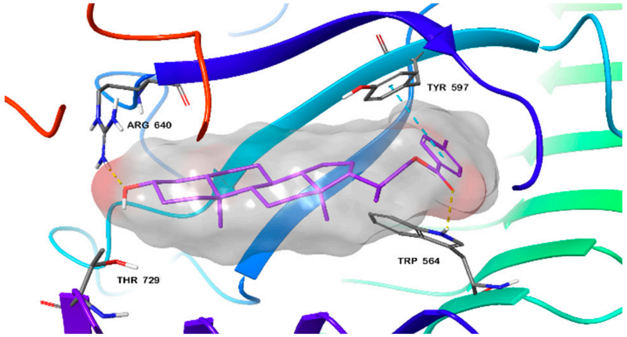 | **-12.01** | **Arg640, Trp 564, Tyr 597.** |
| **17** | 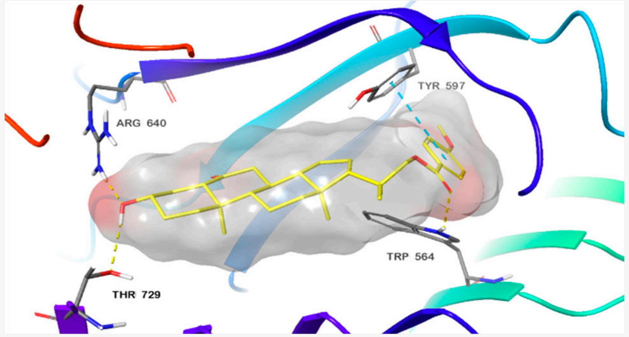 | **-11.96** | **Arg640, Thr 729, Trp 564, Tyr 597.** |

| | | | |
|---|---|---|---|
| **18** | 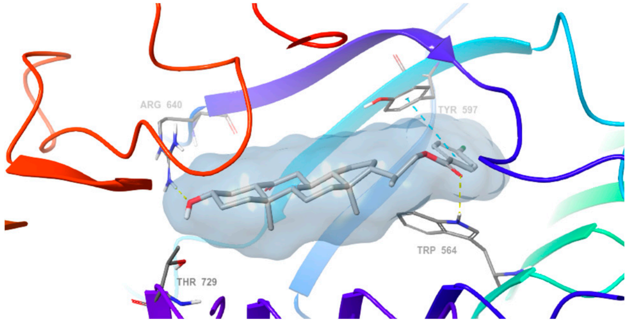 | **-12.03** | **Arg640, Thr 729, Trp 564, Tyr 597.** |
| **19** | 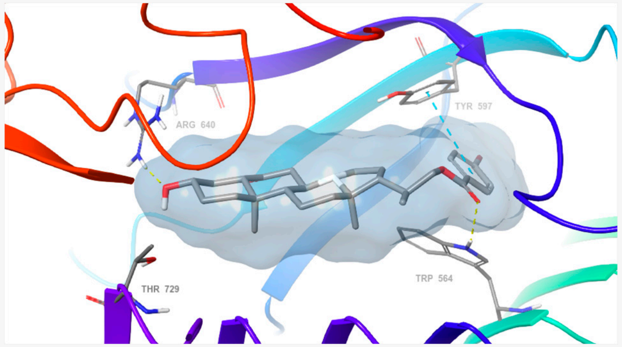 | **-11.96** | **Arg640, Thr 729, Trp 564, Tyr 597.** |
| **20** | 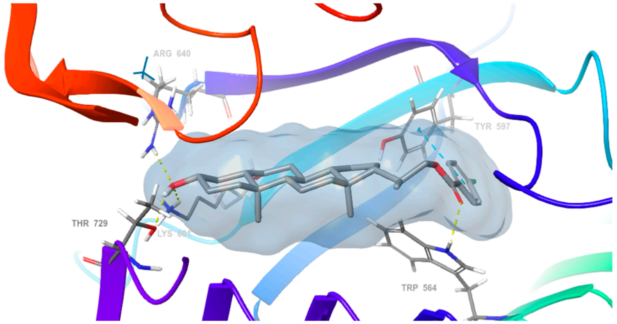 | **-12.24** | **Arg640, Thr 729, Trp 564, Tyr 597, Lys 601.** |

| | | | |
|---|---|---|---|
| **21** | 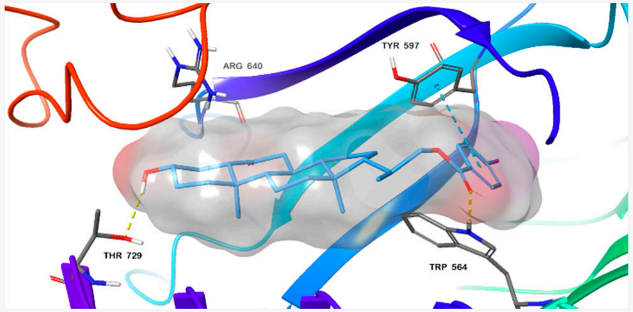 | **-10.70** | **Thr 729, Trp 564, Tyr 597.** |
| **22** | 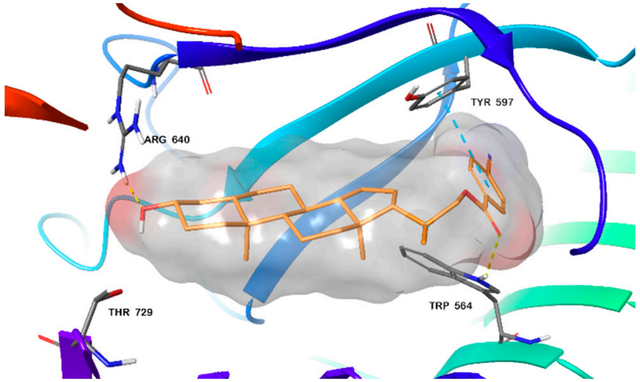 | **-13.17** | **Arg640, Trp 564, Tyr 597.** |
| **31** | 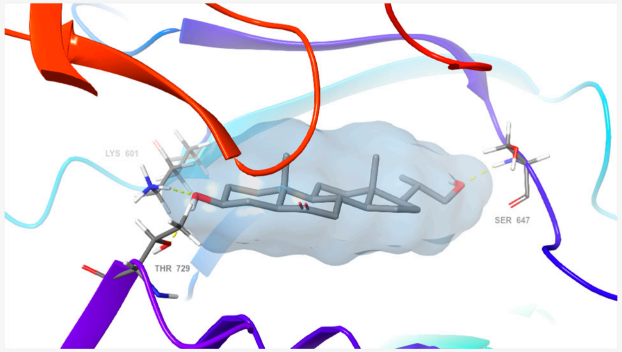 | **-9.67** | **Thr 729, Lys 601, Ser 647.** |

**Disclaimer/Publisher's Note:** The statements, opinions and data contained in all publications are solely those of the individual author(s) and contributor(s) and not of MDPI and/or the editor(s). MDPI and/or the editor(s) disclaim responsibility for any injury to people or property resulting from any ideas, methods, instructions or products referred to in the content.
